# Pyrimidopteridine‐catalyzed Photo‐mediated Hydroacetoxylation

**DOI:** 10.1002/chem.202201761

**Published:** 2022-08-24

**Authors:** Andranik Petrosyan, Luisa Zach, Tobias Taeufer, T. S. Mayer, Jabor Rabeah, Jola Pospech

**Affiliations:** ^1^ Leibniz Institute for Catalysis Albert-Einstein-Str. 29a 18059 Rostock Germany

**Keywords:** C−O bond formation, hydroacetoxylation, photoredox catalysis, pyrimidopteridines

## Abstract

Herein we report a photo‐mediated formal addition of carboxylic acids to activated alkenes catalyzed by a pyrimidopteridine photoredox catalyst. The decarboxylation of aliphatic carboxylic acids upon single‐electron oxidation is countered in the presence of electron‐rich alkenes and a hydroacetoxylation is observed. Mechanistic proposals have been made based on CV measurements, competitive Stern‐Volmer quenching and EPR experiments. Evidence that tetra‐*N*‐substituted pyrimidopteridines function as dual photoredox and hydrogen atom transfer catalyst was supported by spectroscopic means.

## Graphical abstract

Pyrimidopteridine organic photoredox catalysts are applied in a formal addition of carboxylic acids to activated alkenes as a dual photoredox and hydrogen atom transfer catalysts. Electron‐rich alkenes counter the decarboxylation of carboxylic acids upon single‐electron oxidation and a selective hydroacetoxylation is observed.

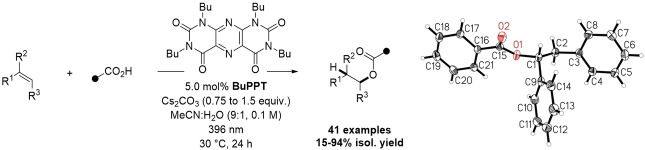

## Introduction

**Scheme 1:**
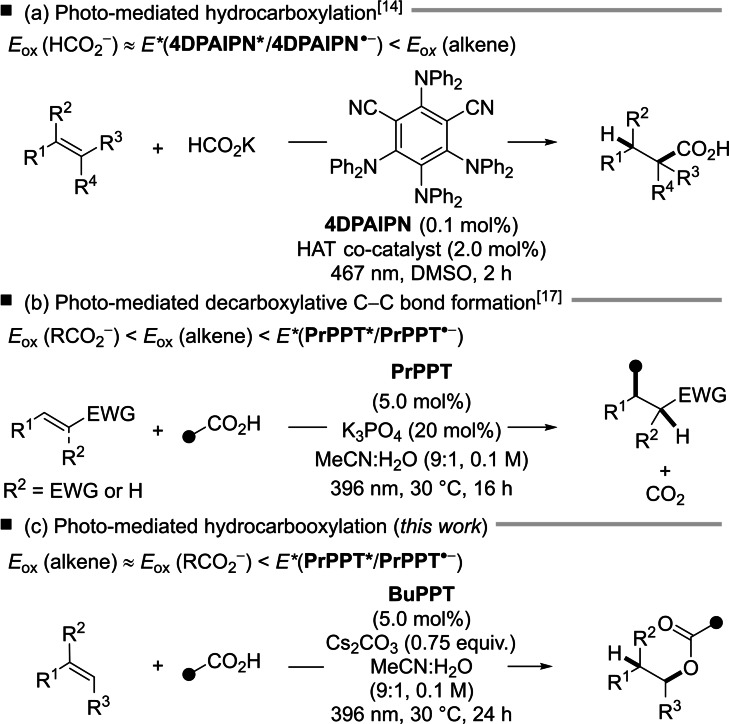
Photo‐mediated (a) C−C bond, (b) decarboxylative C−C bond and (c) C−O bond formation.

Alkene functionalization represents a tool for the rapid and atom‐economic build‐up of structural complexity.^[1]^ In recent years, photochemical approaches have yielded a considerable number of diverse hydro‐ and difunctionalizations of olefins.^[2]^ Most strategies are reliant on the electrophilic activation of olefins and interception by a nucleophilic coupling partner to afford functionalized products.^[3]^ Alternatively, the corresponding nucleophile is activated through proton‐coupled electron transfer (PCET) and reacts with electron‐rich double bonds.^[4]^ Either scenario typically requires a hydrogen atom transfer (HAT) co‐catalyst to convert the *C*‐centered radical intermediates. Carboxylic acids are attractive starting materials for hydrofunctionalization reactions.^[5],[6]^ Organic photoredox catalysts have been particularly widely used in this context.^[7]^ Photo‐mediated C−O bond formations with carboxylic acids catalyzed by acridinium Photoredox catalysts and a HAT co‐catalyst have previously been reported by the groups of Lei and Nicewicz.^[8]^ In 2021, the group of Wickens reported a photo‐induced hydrocarboxylation through the delivery of formate across activated C=C double bonds (Scheme 1a).^[9]^ Our group has recently introduced organic pyrimidopteridine photoredox catalysts and exemplified their applicability in photo‐mediated hydrofunctionalization reactions in the absence of additional HAT catalysts.^[10]^ We have utilized carboxylic acids as starting materials in photo‐mediated pyrimidopteridine‐catalyzed decarboxylative C−C bond formation which proceeds via oxidation of carboxylic acids, decarboxylation and interception of the corresponding alkyl radical with electron‐deficient alkenes (Scheme 1b).^[11]^ In contrast, the pyrimidopteridine‐catalyzed hydroamination of stilbenes proceeds via alkene oxidation.^[12]^ In the course of our study on the photo‐mediated decarboxylative C−C bond formation we have noticed that in some occasions, the decarboxylation of aliphatic carboxylic acids is suppressed. Instead, a hydroacetoxylation product was isolated (Scheme 1c). In this article, we present our findings on the reactivity‐controlling aspects of the formal addition of carboxylic acids to activated alkenes catalyzed by organic pyrimidopteridine photoredox catalysts in the absence of additional HAT catalysts.

## Results and Discussion

The reaction conditions were optimized using (*E*)‐stilbene (**1 a**) (*E* ^ox^_1/2_=+1.53 V vs. SCE in MeCN)^[12]^ and benzoic acid (**2 a**) (*E* ^ox^_1/2_=+0.94 V vs. SCE in MeCN for BzO^−^). Benzoic acids are generally more robust towards undesired photo‐mediated decarboxylations which proceed only at UV−B irradiation in the presence of suitable acceptors.^[13]^ Tetrabutyl pyrimidopteridine (E*_red_ =+2.10 V vs. SCE in MeCN)^[10b]^ was identified as optimal photoredox catalyst under irradiation at 396 nm in MeCN:H_2_O (9 : 1). A competitive hydration as described by Lei and co‐workers was not observed.[^8d^, ^8e^] In the course of the optimization a strong cesium effect was evident.^[14]^ Cesium carbonate yielded the desired product **3 a** in up to 89 % isolated yield, whereas other bases resulted in varying yields between 20–40 %.^[15]^ In the absence of base, catalyst or light, no conversion of the starting materials was observed. The use of sub‐stoichiometric amounts of base diminished the reaction rate.

Next, we turned our attention to the reaction scope. The formal esterification of (hetero)aromatic and alkenyl carboxylic acids in the presence of (*E*)‐stilbene (**1 a**) was accomplished in 18 examples (Table 1). The corresponding α‐phenyl phenylethylesters **3** were obtained in synthetically useful yields of up to 94 % for benzoic acid derivatives. Heteroaromatic carboxylic acids were converted in varying yields. Alkenylic carboxylic acids yielded the products 45–61 % isolated yields. The 1,2‐diphenylethyl perillic acid ester (**3 r**) was isolated as a 1:1 mixture of diastereomers.

**Table 1:**
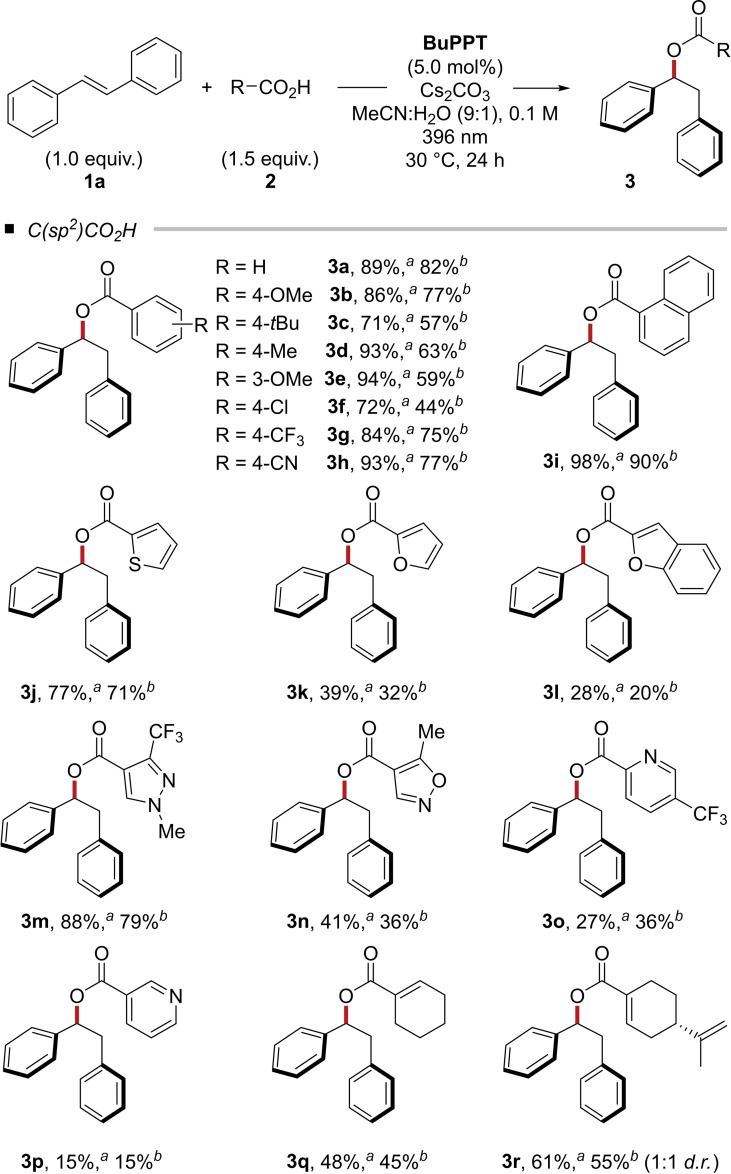
Scope of (hetero)aromatic and alkenylic carboxylic acids in the photo‐mediated hydroacetoxylation of (*E*)‐stilbene (**1 a**).^*a*^

^a^Reaction conditions: **1** (0.5 mmol), **2** (0.75 mmol), Cs_2_CO_3_ (0.75 mmol), **BuPPT** (5.0 mol %), MeCN:H_2_O (9 : 1, 5.0 mL), 30 °C, 24 h. Isolated yields are shown. ^b^Cs_2_CO_3_ (0.36 mmol) was used.

The decarboxylation of aliphatic carboxylic acids upon single‐electron oxidation is a well‐studied and rapid process.^[16]^ Nevertheless, under the applied reaction conditions, only minor amounts of protodecarboxylation products (<5 %) were observed. Several structurally divers aliphatic carboxylic acids were successfully converted. Linear and branched aliphatic carboxylic acids were converted to the corresponding products in 13–79 % isolated yield (Table 2). Noteworthy, the highest yield of 79 % was obtained from cyclopropylcarboxylic acid (**2 e**) which in case of single‐electron oxidation of the carboxylic acid would result in rapid decarboxylation and opening of the cyclopropyl ring. Similarly, α‐chiral (*S*)‐2,2‐dimethylcyclopropane‐1‐carboxylic acid yielded ester **5 m** as 1 : 1 mixture of diastereomers in 55 % yield using 0.75 equiv. of base within 24 h. Surprisingly, other cycloalkyl carboxylic acids yielded the products in considerably lower yields (**5 f**–**5 k**). Levulinic acid bearing a ketone functionality was converted to 1,2‐diphenylethyl 4‐oxopentanoate (**5 o**) in up to 59 % isolated yield. The phenol moieties of the plasticizer diphenolic acid can serve as radical scavenger and had to be acetate protected prior to the reaction to form diphenolic ester **5 p**.

**Table 2:**
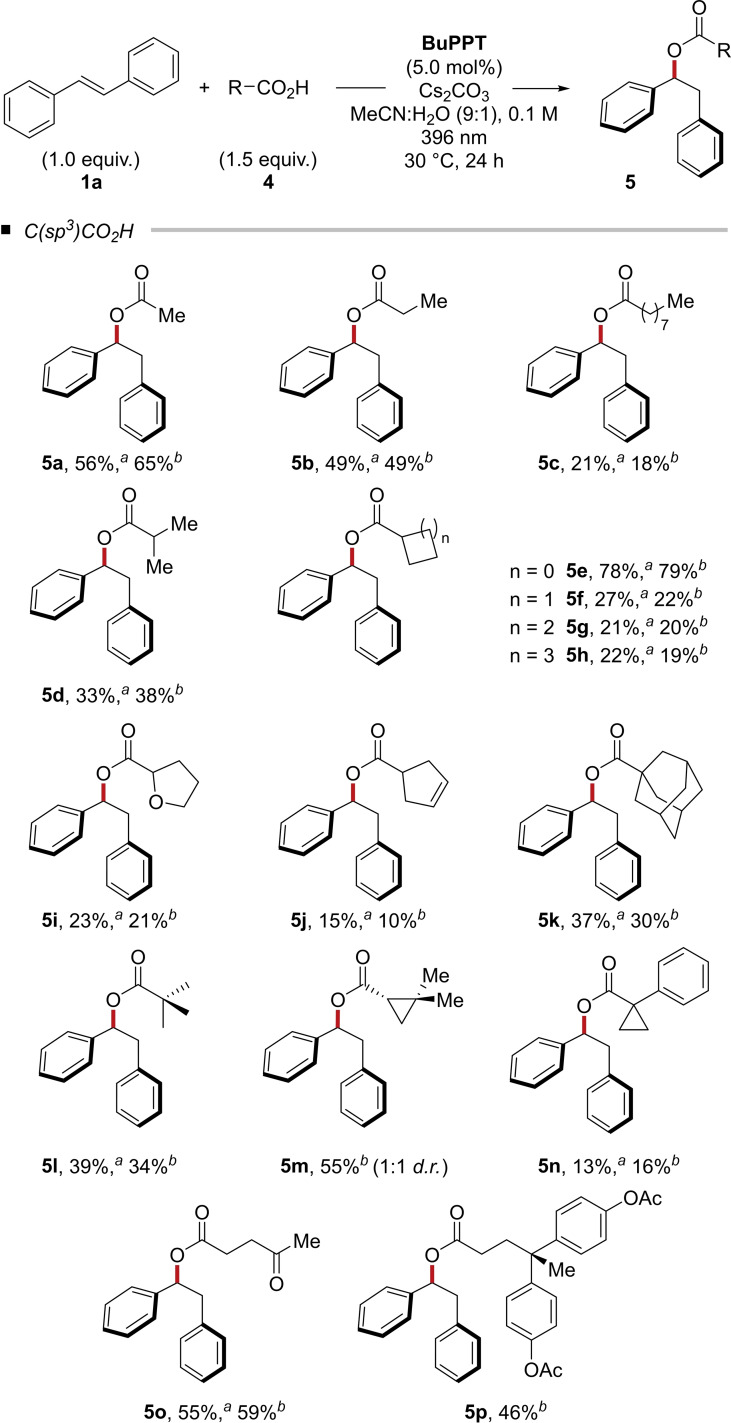
Scope of aliphatic carboxylic acids in the photo‐mediated hydroacetoxylation of (*E*)‐stilbene (**1 a**).^*a*^

^a^Reaction conditions: **1** (0.5 mmol), **4** (0.75 mmol), Cs_2_CO_3_ (0.75 mmol), **BuPPT** (5.0 mol %), MeCN:H_2_O (9 : 1, 5.0 mL), 30 °C, 24 h. Isolated yields are shown. ^b^Cs_2_CO_3_ (0.36 mmol) was used.

In the following, we turned our attention to the alkene scope (Table 3). The implementation of an anisyl group into a molecular scaffold results in a drastic decrease of the innate oxidation potentials (*E* ^ox^_1/2_=+2.00 V for **1 b** versus +1.55 V for **1 c** vs. SCE in MeCN).^[17]^ Thus, amongst others, vinylanisols serve as especially suitable substrates for the photo‐mediated hydrooxylation yielding up to 90 % isolated yield of the corresponding benzoate (Table 3). Furthermore, (*E*)‐1‐styryl‐4‐(trifluoromethyl) benzene (**1 f**, *E* ^ox^_1/2_=+1.69 V vs. SCE in MeCN)^[12]^ was successfully converted, yielding 73 % of the benzoylated product **7 f** as a 1 : 2 mixture of diastereomers. Estrone pentan‐2‐yl benzoate (**7 g**) was isolated in 64 % yield.

**Table 3:**
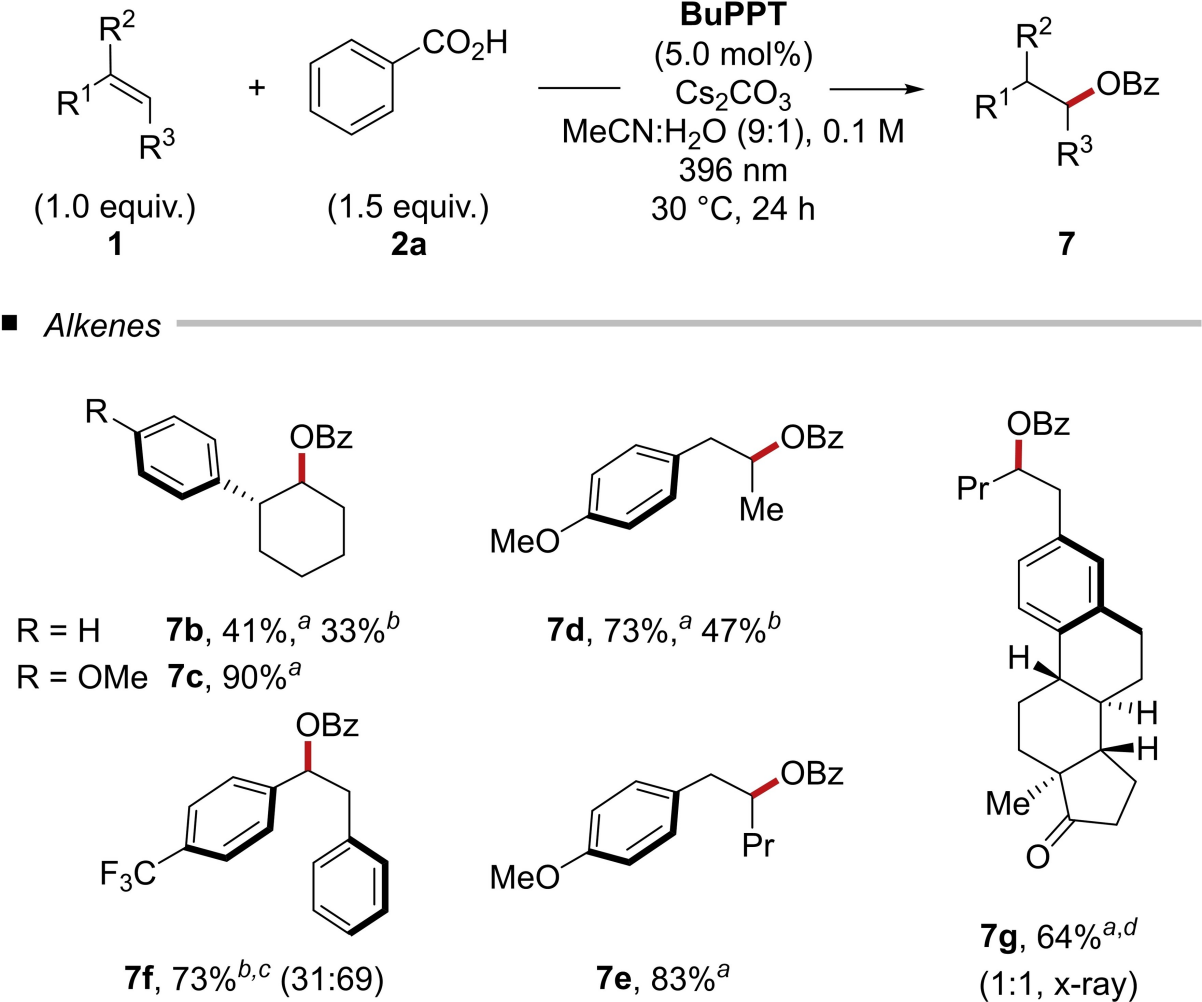
Scope of alkenes in the photo‐mediated hydroacetoxylation with benzoic acid (**2 a**).^*a*^

^a^Reaction conditions: **1** (0.5 mmol), **2** (0.75 mmol), Cs_2_CO_3_ (0.75 mmol), **BuPPT** (5.0 mol %), MeCN:H_2_O (9 : 1, 5 mL), 30 °C, 24 h. Isolated yields are shown. ^b^Cs_2_CO_3_ (0.75 mmol) was used. ^c^Major isomer is shown. ^d^EtOAc (10 % v/v) was added.

**Scheme 2:**
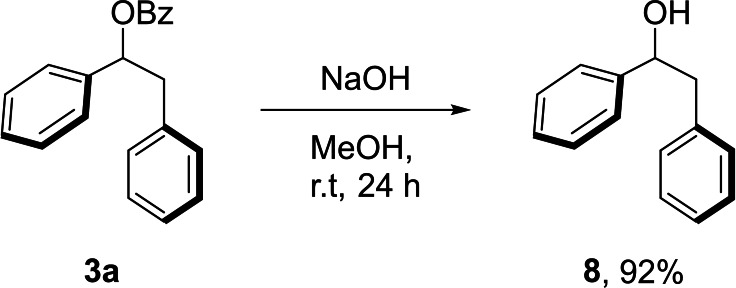
Cleavage of the benzoyl protecting group.

The benzoyl group in 1,2‐diphenylethyl benzoate (**3 a**) was efficiently saponified under basic reaction conditions. The corresponding free alcohol **8** was obtained in 92 % isolated yield after an aqueous work‐up (Scheme 2).

**Figure 1:**
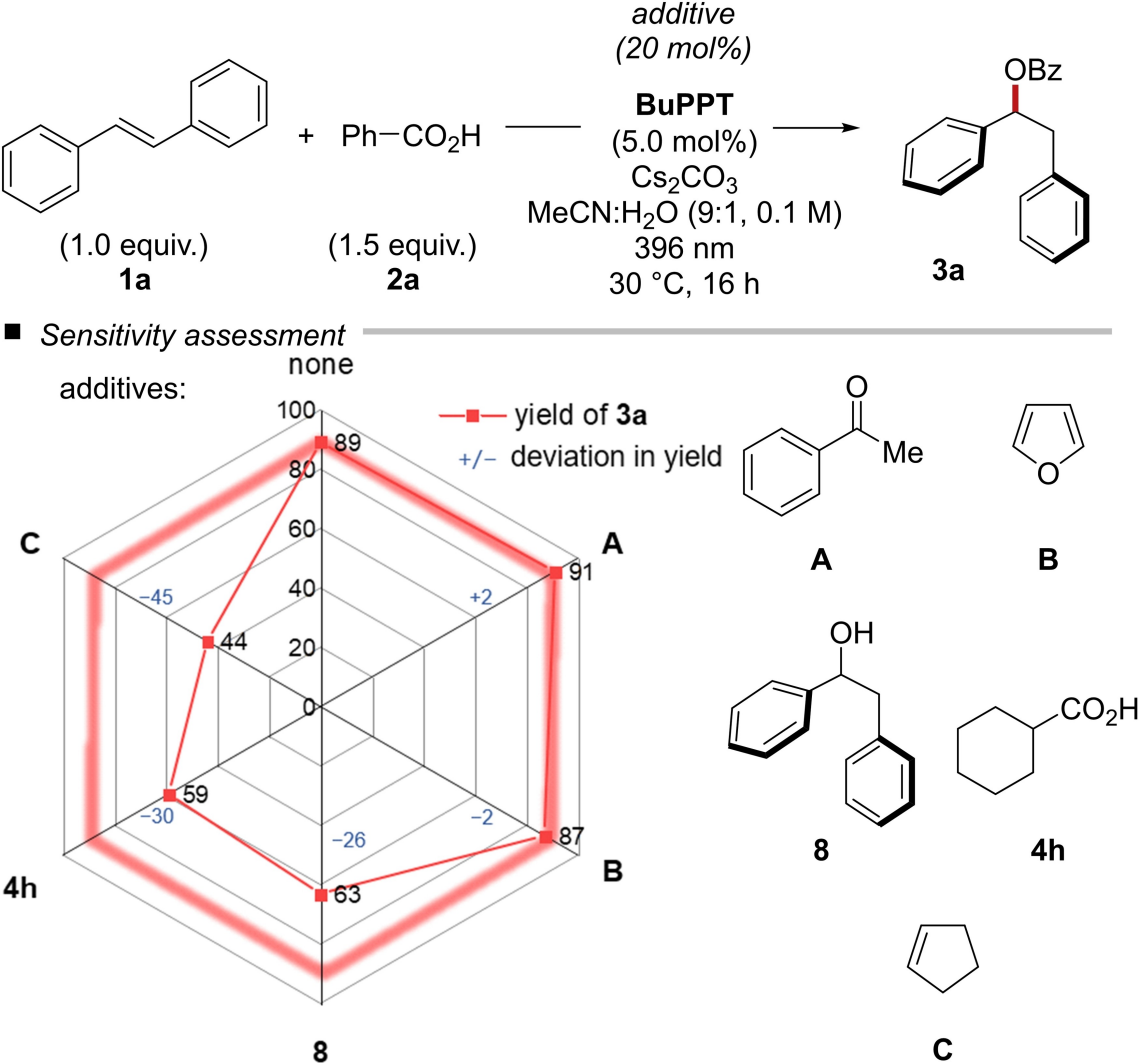
Sensitivity assessment of the photo‐mediated hydroacetoxylation of (*E*)‐stilbene (1a) with benzoic acid (2a).

Next, we performed mechanistic experiments. To investigate the cause of the reduced yield of some aliphatic carboxylic acids, we performed long‐term experiments over 72 h or successive addition of carboxylic acid in two portions. Neither protodecarboxylation nor saponification of the esters were pronounced reaction pathways. However, small amounts (<10 %) of hydroacylation products were formed in some cases.^[18]^ At this point, we have to assume a unknown catalyst deactivation pathway. A sensitivity assessment was performed by selective addition of potential quenchers (Figure 1). The addition of acetophenone and furan ($E_{1/2}^{ox}$ =+1.70 V vs. SCE in MeCN)^[19]^ resulted in no reduction in yield. In the presence of sub‐stoichiometric amounts of alcohol **8** or cyclohexylcarboxylic acid **4 h**, the desired product was obtained in diminished yields of 63 % and 59 % respectively. Thus, the nature of the hydrogen atom donor appears to affect the reaction outcome. Cyclopentene ($E_{1/2}^{ox}$ =+2.32 V vs. SCE in MeCN)^[20]^ had a surprisingly big effect on the isolated product yield which was diminished to 44 %.

**Figure 2:**
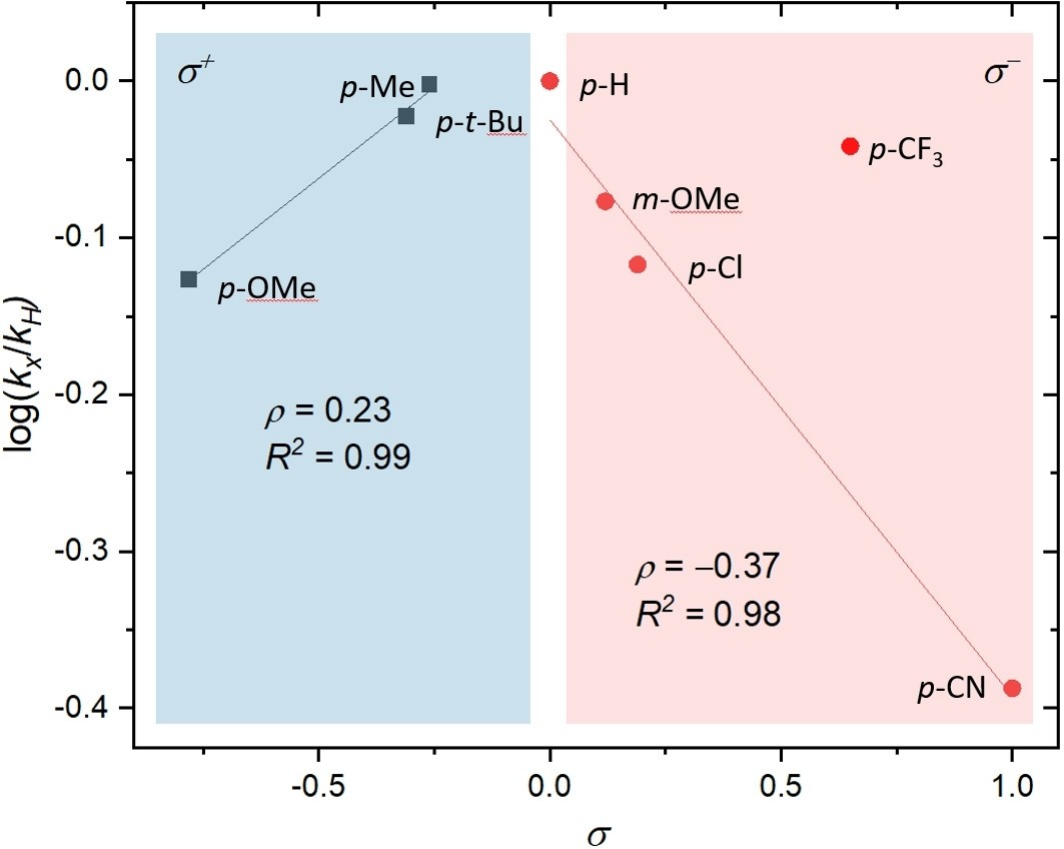
Hammett plot for the photo‐mediated hydroacetoxylation of (*E*)‐stilbene (1a) with benzoic acids 2a–h.

Furthermore, a Hammett plot analysis was developed from intermolecular competition experiments between benzoic acid (**2 a**) and substituted benzoic acids **2 b**–**2 h** at low conversion. The experiments result in a broken, linear Hammett relationship between substrates with electron‐donating (best correlated with *σ* ^+^) and electron‐withdrawing groups (best correlated with *σ* ^−^) with a break at X=H (Figure 2).^[21]^ This observation suggests a change of the rate‐determining step depending on the electronic nature of the substrate. Benzoic acids with *σ* values near zero produce nearly equal mixtures of products. As *σ* ^+^ becomes more negative the relative rate of the reaction decreases. The positive slope (*ρ=*+0.23) suggests the build‐up of a negative charge in the rate‐determining step, for example, the deprotonation of the benzoic acid. Likewise, as *σ* ^−^ values become more positive, the relative rate of the reaction decreases. In this scenario the negative slope (*ρ*=−0.37) suggest the build‐up of a positive charge in the rate‐determining step, for example, the nucleophilic attack of benzoate at a radical cation. *Para*‐Trifluoromethyl benzoic acid (**2 g**, *σ* ^−^= 0.74) is a notable outlier to this trend.

**Scheme 3:**
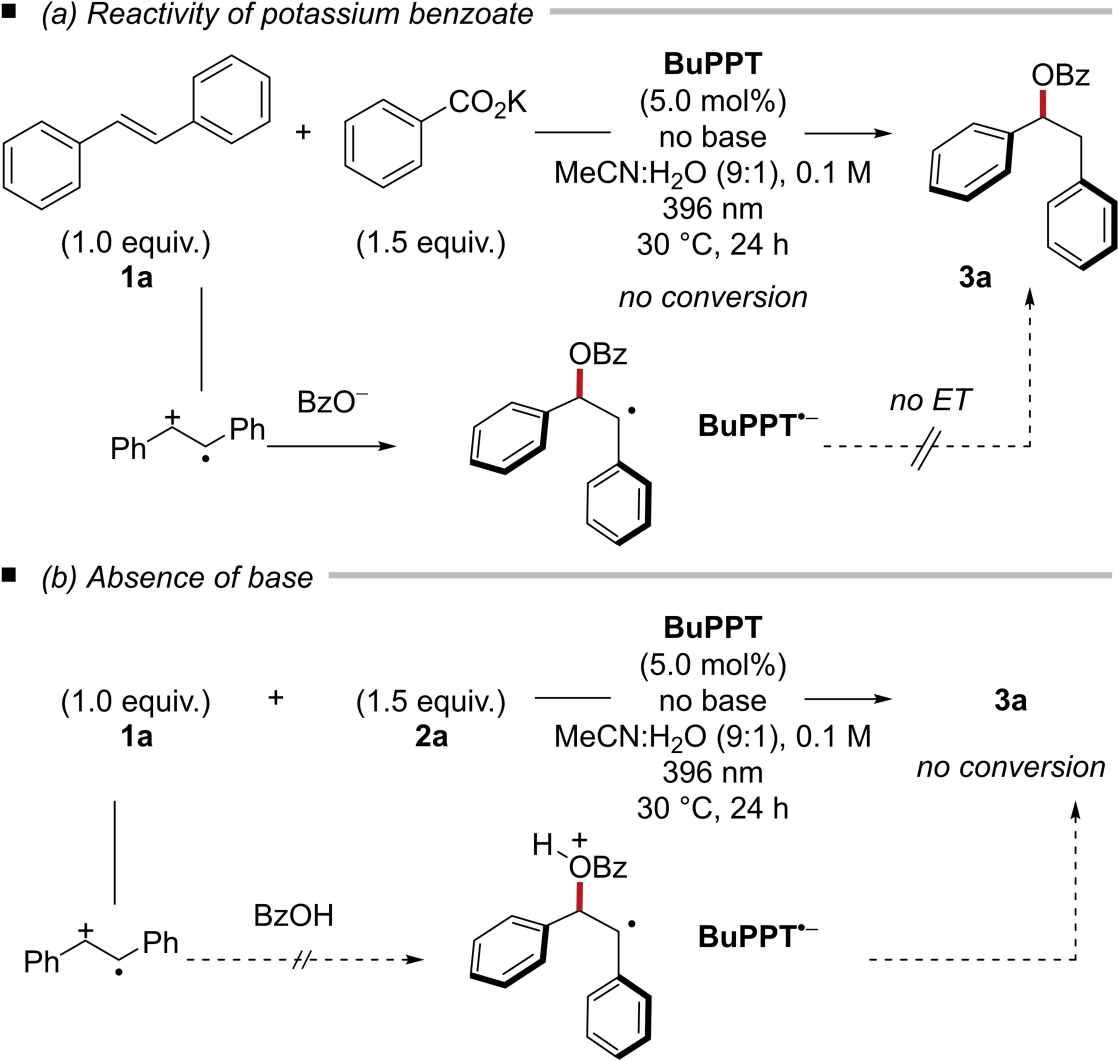
Mechanistic experiments.

Competitive Stern‐Volmer experiments revealed that (*E*)‐stilbene (*K*_SV_=58.2 m ^−1^ for **1 a**) is a significantly better quencher for the excited state of catalyst BuPPT* as compared to benzoate (*K*_SV_=19.2 m ^−1^ for tetrabutylammonium benzoate (**2 a**⋅**TBA**)). Thus, an initial single‐electron oxidation of the alkene can be anticipated. When potassium benzoate is used in the absence of additional base, no product formation was observed (Scheme 3a). Since the oxidation of the (*E*)‐stilbene (**1 a**) by the excited state catalyst is not affected in this scenario, it can be assumed that the benzoate attacks the generated carbocation yielding the neutral benzylic radical and the radical anion of the catalyst **PPT**⋅^**−**^. However, the subsequent reduction of a *C*‐centered benzyl radical (*E* ^red^_1/2_=−1.60 V vs. SCE in MeCN for ethylenebenzene)^[22]^ is thermodynamically unfavorable (*E* ^red^_1/2_(**BuPPT**/**BuPPT**⋅^**−**^)=−1.21 V vs. SCE in MeCN)^[10b]^ and can be ruled out due to the lack of conversion. Likewise, no conversion was observed under standard reaction conditions but in the absence of a base, demonstrating the poor nucleophilicity of carboxylic acids (Scheme 3b). These observations are in accordance with the broken linear free energy relationship.

As evidenced by EPR spectroscopy, the pyrimidopteridine radical anion **PPT**⋅^**−**^ is protonated to **PPTH**⋅ in the presence of benzoic acid.^[23]^ This species is deemed essential for the turn‐over of the catalytic cycle and product formation by means of hydrogen atom transfer to the benzylic radical.

**Figure 3:**
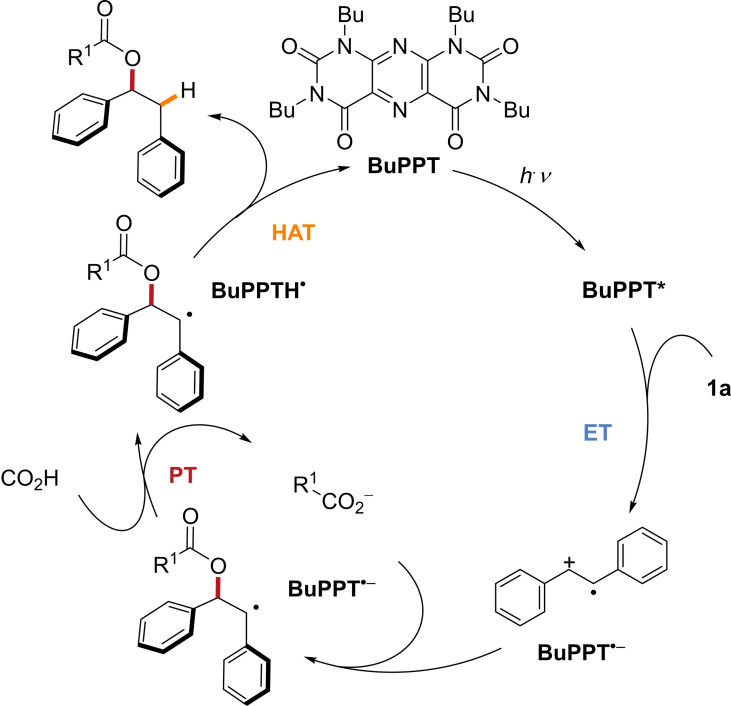
Proposed catalytic cycle.

A plausible catalytic cycle for the photo‐mediated hydroacetoxylation of (*E*)‐stilbene (**1 a**) with carboxylic acids is depicted in Figure 3. The reaction sequence commences with the excitation of the **BuPPT** catalyst at 396 nm.^[10b]^ Initial single‐electron transfer (SET) from (*E*)‐stilbene to the excited state catalyst results in the radical anion **BuPPT**⋅^**−**^and the (*E*)‐stilbene radical cation. The latter reacts with a carboxylate ion to form a benzylic *C‐*centered radical intermediate. Subsequent HAT from the **BuPPTH**⋅ to the C‐centered radical regenerates the photoredox catalyst and leads to the formation of the hydroacetoxylation product.

## Conclusion

In summary, we have disclosed a protocol for the photo‐mediated hydroacetoxylation of various alkenes with a broad range of carboxylic acids. We have demonstrated that the C−O bond formation outcompetes the photo‐mediated decarboxylation of aliphatic carboxylic acids under the optimized reaction conditions. A Hammett‐plot analysis revealed a broken linear free‐energy suggesting a change of the rate‐determining step depending on the electronic nature of the substrates. Furthermore, mechanistic in conjunction with luminescence quenching experiments and EPR spectroscopic data allowed us to propose a catalytic cycle underscoring the dual role of pyrimidopteridines as photoredox and hydrogen atom transfer catalysts.

## Experimental Section

**General procedure**: In a typical experiment, a 10 mL Schlenk flask equipped with a magnetic stir bar was charged with the alkene (0.5 mmol, 1.0 equiv.), the corresponding carboxylic acid [if solid] (0.75 mmol, 1.5 equiv.), BuPPT (5.0 mol%), Cs_2_CO_3_ (0.75 mmol, 1.5 equiv.), and was sealed with a rubber septum. The flask was then repeatedly evacuated and purged with argon. The corresponding carboxylic acid [if liquid] (0.75 mmol, 1.5 equiv.), acetonitrile (4.5 mL) and water (0.5 mL) were subsequently added under inert atmosphere. The reaction mixture was stirred for 24 h under irradiation at 396 nm. After the indicated time, aqueous K_2_CO_3_ solution (10 wt%, 15 mL) and brine (15 mL) were added, and the reaction mixture was extracted with dichloromethane (4×15 mL). The combined organic layers were washed with brine (15 mL) and dried over anhydrous Na_2_SO_4_, filtered and concentrated under reduced pressure. The residue was purified by column chromatography on silica gel using mixture of *n*‐pentane and ethyl acetate as eluent.

**Synthesis of 1,2‐diphenylethyl benzoate (3 a)**: Compound 3a was prepared following the general procedure. Purification by column chromatography (*n*‐pentane:EtOAc=50:1→20 : 1) yielded the title compound **3 a** (135 mg, 0.45 mmol, 89 %) as a colorless solid. **R**_***f***_=0.28 (*n*‐pentane/EtOAc=40 : 1, UV); **m.p**.=67–68 °C; ^**1**^ **H NMR** (300 MHz, Chloroform‐*d*) δ 8.06–8.02 (m, 2H), 7.54–7.48 (m, 1H), 7.43–7.11 (m, 12H), 6.18 (dd, *J*=7.6, 6.0 Hz, 1H), 3.35 (dd, *J*=13.8, 7.6 Hz, 1H), 3.18 (dd, *J*=13.8, 6.0 Hz, 1H); ^**13**^ **C NMR** (75 MHz, Chloroform‐*d*) δ 165.7, 140.2, 137.0, 133.0, 130.4, 129.7, 128.5, 128.4, 128.3, 128.1, 126.7, 126.6, 77.4, 43.3; **MS** (EI): *m*/*z* (relative intensity) 211 (26), 180 (18), 165 (5), 105 (100), 91 (5), 77 (23); **HRMS** (ESI‐TOF, *m*/*z*): calcd. for C_21_H_18_O_2_ [M+Na^+^] 325.1204; found 325.1210; **IR** (ATR, neat, cm^−1^): 3060 (w), 3027 (w), 2942 (w), 2864 (w), 1703 (m), 1600 (w), 1494 (w), 1451 (w), 1268 (m), 1100 (m), 1070 (m), 1026 (m), 974 (m), 911 (w), 801 (w), 757 (m), 714 (s), 695 (s), 613 (m), 595 (w), 555 (s), 497 (m).

## Conflict of interest

The authors declare no conflict of interest.

1

## Supporting information

As a service to our authors and readers, this journal provides supporting information supplied by the authors. Such materials are peer reviewed and may be re‐organized for online delivery, but are not copy‐edited or typeset. Technical support issues arising from supporting information (other than missing files) should be addressed to the authors.

Supporting Information

## Data Availability

The data that support the findings of this study are available in the supplementary material of this article.
