# Supplementary material for: Pyrimidopteridine‐catalyzed Photo‐mediated Hydroacetoxylation
Source: Chemistry. 2022 Aug 24;28(57):e202201761. doi: 10.1002/chem.202201761 (PMC9804165; doi:10.1002/chem.202201761)
Supplement: Supplementary file 1 — Supporting Information [file CHEM-28-0-s001.pdf]

Chemistry–A European Journal

Supporting Information

Pyrimidopteridine-catalyzed Photo-mediated Hydroacetoxylation

Andranik Petrosyan, Luisa Zach, Tobias Taeufer, T. S. Mayer, Jabor Rabeah, and Jola Pospech*

Contents

General Remarks 2

Reaction Set-up 3

Experimental Section... 4

Optimization of Reaction Conditions 4

General Procedures 7

Scope of benzoic and alkenyl carboxylic acids... 8

Scope of alkyl carboxylic acids 21

Scope of alkenes... 33

Deprotection of 1,2-diphenylethylbenzoate (7a)... 39

Crystallographic data.. 41

Stern-Volmer Experiments... 43

Competitive Stern-Volmer Experiments... 46

EPR Spectroscopy... 50

Reaction Monitoring.. 51

Free-energy relationship (Hammett-plot analysis) 52

Mechanistic experiments.. 54

Reactivity of potassium benzoate... 54

Reactivity in the absence of base 54

Long-time experiments 55

Sensitivity assessment.. 56

NMR Spectra 57

References 107

General Remarks

Unless otherwise noted, all reactions were carried out under an inert atmosphere. All chemicals were purchased from commercial suppliers and used as received. Pyrimidopteridinetetraone *N*-oxides (PPTNO) and pyrimidopteridinetetraones (PPT) were synthesized according to procedures previously reported by our group.[1, 2] Dry dichloromethane (CH_2Cl_2), acetonitrile (MeCN), hexanes and tertrahydrofuran (THF) was obtained by passing commercially available anhydrous, oxygen-free HPLC-grade solvents through activated alumina columns using an MBRAUN solvent purification system. Hexafluoroisopropanol (HFIP) was distilled under inert atmosphere and stored over molecular sieves. Analytical thin-layer chromatography was performed on Merck silica gel 60 F254 aluminium plates. Visualization was accomplished with UV light and/or potassium permanganate ($KMnO_4$) or cerium ammonium molybdate stain (CAM). Retention factor (R_f) values were measured using a 5 × 2 cm TLC plate in a developing chamber containing the solvent system described. Flash column chromatography was performed using Silicycle SiliaFlash® P60 (SiO_2, 40-63 μm particle size, 230-400 mesh). ^{1}H and ^{13}C NMR spectra were recorded on Bruker 300 & 400 (400 MHz, ^{1}H; 100 MHz, ^{13}C). Spectra are referenced to residual chloroform (δ = 7.26 ppm, ^{1}H; 77.16 ppm, ^{13}C), residual dimethyl sulfoxide (δ = 2.50 ppm, ^{1}H; 39.5 ppm, ^{13}C), residual methanol (δ = 3.31 ppm, ^{1}H; 49.0 ppm, ^{13}C), or residual benzene (δ = 7.16 ppm, ^{1}H; 128.06 ppm, ^{13}C). Chemical shifts are reported in parts per million (ppm). Multiplicities are indicated by s (singlet), d (doublet), t (triplet), q (quartet), m (multiplet), and br (broad). Coupling constants *J* are reported in Hertz (Hz). Mass spectrometry (MS) was performed by the LIKAT analytic department. Electron Impact (EI^+) spectra were performed at 70 eV using methane as the carrier gas, with time-of-flight (TOF) mass analyzer. Electrospray ionization (ESI^+) spectra were performed using a time-of-flight (TOF) mass analyzer. Data are reported in the form of m/z (intensity relative to the base peak = 100). High-resolution mass was obtained by using Waters Q-TOF Ultima ESI and Agilent 6230 ESI TOF LC/MS spectrometers. Infrared spectra were measured neat on a Perkin-Elmer spectrum BX FT-IR spectrometer. Peaks are reported in cm^{-1} with indicated relative intensities: s (strong, 0–33% T); m (medium, 34–66% T), w (weak, 67–100% T), and br (broad). The emission spectrum of the LED was recorded using an Avantes Sensline Avaspec-ULS TEC Spectrometer. Melting points of solid products were measured on a Buchi B-540 melting point apparatus and are uncorrected.

Reaction Set-up

Unless otherwise stated, all reactions were performed on a 0.5 mmol scale and a molarity of 0.1 M under inert atmosphere. The reactions were irradiated using 2 x 30 W in 5.7 and 10.4 cm distance (Figure S1). The emission spectra of the light source and the superimposed emission spectra of the light source and the absorption spectra of the **PPT** and **PPTNO** catalyst are shown in Figure S2.

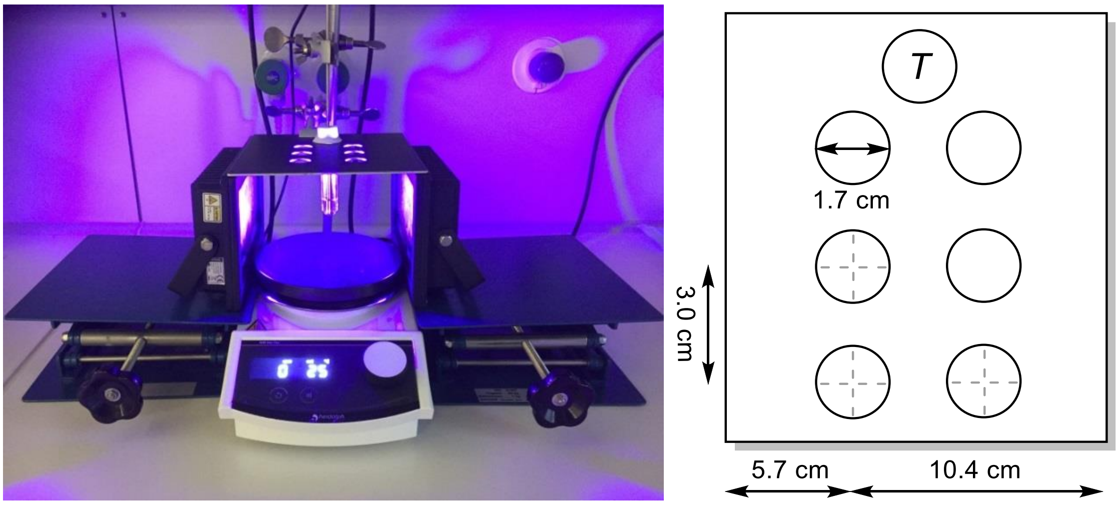

Figure S1: Reaction set-up.

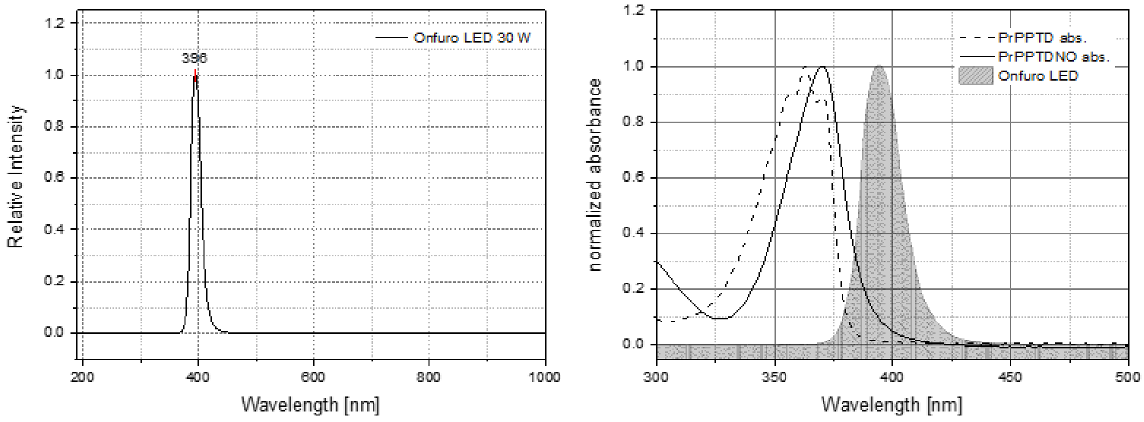

Figure S2: Emission spectrum of the utilized LED-lamp ONFURO IP66 (30 W) (left). Absorption spectra of PrPPTNO and PrPPT and emission spectra of ONFURO IP66 LED.

Experimental Section

Optimization of Reaction Conditions

Table S 1. Screening of bases.[a]

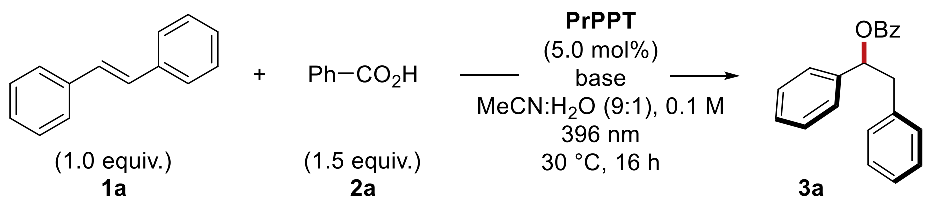

entry	base	equiv.	**2a**:base ratio	yield [%][a]
1	K_3PO_4	0.2	7.5:1	33
2	K_3PO_4	1.0	1.5:1	33
3	K_3PO_4	1.5	1:1	43
4	K_3PO_4	2.0	1:1.3	32
5	K_3PO_4	3.0	1:2	10
6	CsF	1.5	1:1	24
7	Cs_2CO_3	1.5	1:1	(70)
8	Cs_2CO_3	1.5	1:1	(89)[b,c]
9	Cs_2CO_3	0.75	2:1	(81)[b,c]
10	Cs_2CO_3	0.5	3:1	(59)[b,c]
11	Cs_2CO_3	0.25	6:1	(39)[b,c]
12	K_2HPO_4	1.5	1:1	43
13	K_2CO_3	1.5	1:1	44
14	Na_2CO_3	1.5	1:1	47
15	$NaHCO_3$	1.5	1:1	36
16	LiOAc	1.5	1:1	20
17	NaOMe	1.5	1:1	32
18	NaOH	1.5	1:1	12

[a] Yields were determined by calibrated GC using biphenyl as internal standard. Isolated yields in paranthese; [b] BuPPT (5.0 mol%) was used as catalyst; [c] 24 h reaction time.

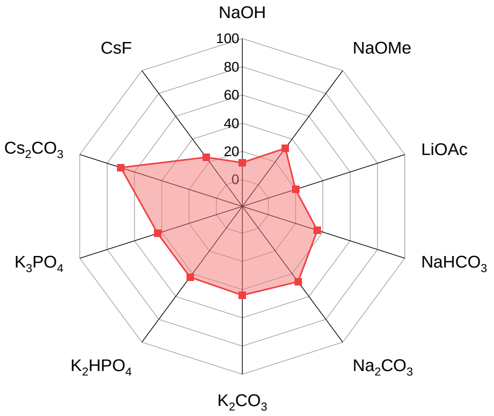

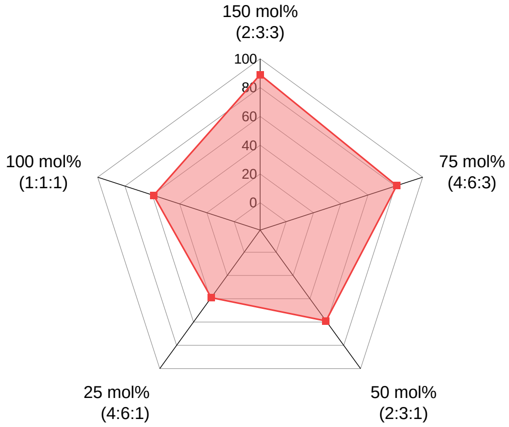

Table S 2. Solvent Screening.[a]

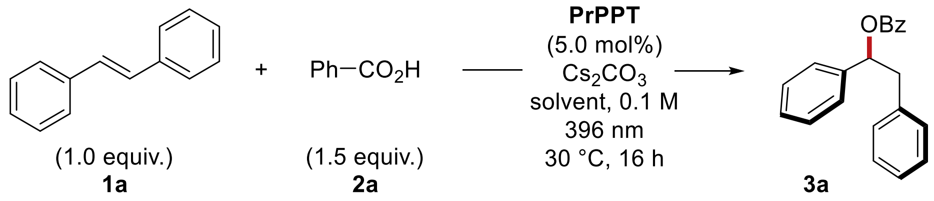

entry	solvent	ratio	molarity [M]	yield [%][a]
1	PhMe	-	0.1	0
2	PhMe:H_2O	-	0.1	0
3	CH_2Cl_2	-	0.1	0
4	dioxane	-	0.1	0
5	acetone	-	0.1	0
6	DMSO	-	0.1	0
7	MeCN:H_2O	9:1	0.1	(70)
8	MeCN:MeOH	9:1	0.1	0
9	MeCN:*i*-PrOH	9:1	0.1	0
10	MeCN:HFIP	9:1	0.1	(64)
11	PrCN:H_2O	9:1	0.1	4
12	PhCN:H_2O	9:1	0.1	11

[a] Cs_2CO_3 (1.5 equiv.) was used as base. Yields were determined by calibrated GC using biphenyl as internal standard. Isolated yields in paranthese.

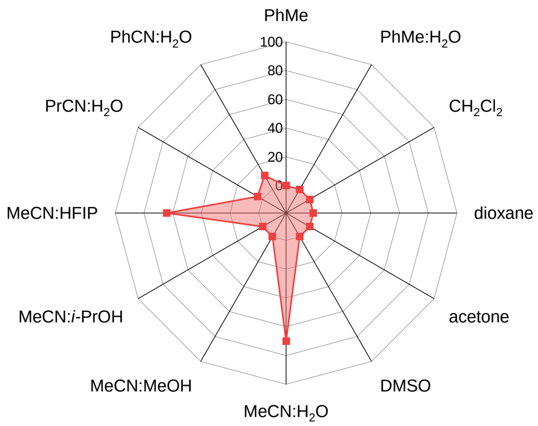

Table S 3. Catalyst Screening.[a]

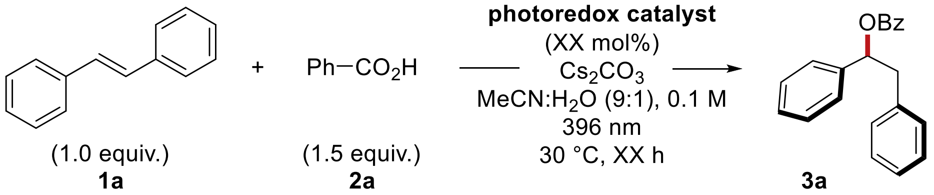

entry	photoredox catalyst	[mol%]	time [h]	yield [%][a]
1	MePPTNO	5.0	16	5
2	MePPT	5.0	16	12
3	PrPPTNO	5.0	16	(62)
4	PrPPT	5.0	16	(70)
5	BuPPTNO	5.0	16	(68)
6	BuPPT	5.0	16	(80)
7	PhPPTNO	5.0	16	2
8	PrPPT	2.5	16	55
9	PrPPT	1.0	16	42
10	PrPPT	5.0	24	(83)
11	PrPPT	2.5	24	58
12	PrPPT	5.0	48	(90)
13	BuPPT	5.0	24	(89)

[a] Yields were determined by calibrated GC using biphenyl as internal standard. Isolated yields in paranthese.

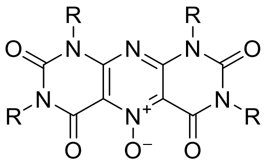

R = Me **MePPTNO**
Pr **PrPPTNO**
Bu **BuPPTNO**
Ph **PhPPTNO**

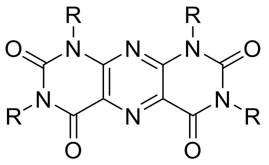

R = Me **MePPT**
Pr **PrPPT**
Bu **BuPPT**
Ph **PhPPT**

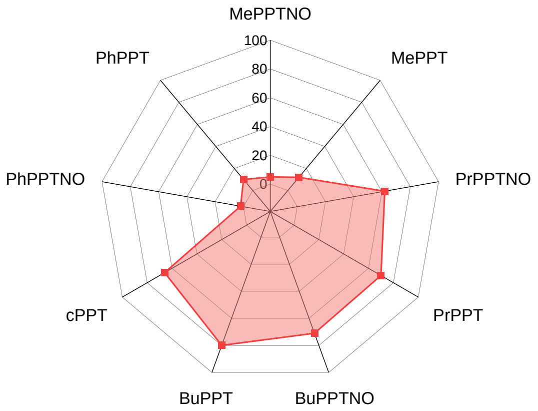

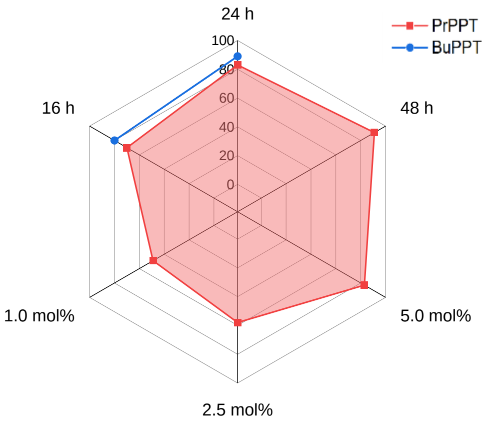

Table S 4. Control experiments.[a]

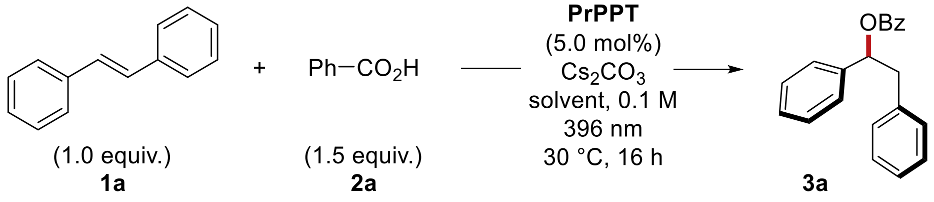

entry	deviation from optimized reaction conditions	yield [%][a]
1	no photocatalyst	<5
2	in the dark	<5
3	no base	<5

[a] **1a** (1.0.equiv.), **2a**(1.5 equiv.), Cs_2CO_3 (1.5 equiv.), PrPPT (5.0 mol%), 16 h. Yields were determined by calibrated GC using biphenyl as internal standard.

General Procedures

General procedure A. In a typical experiment, a 10 mL Schlenk flask equipped with a magnetic stir bar was filled with *E*-stilbene (**1a**) (0.5 mmol, 1.0 equiv.), corresponding carboxylic acid [if solid] (0.75 mmol, 1.5 equiv.), BuPPT (5.0 mol%), Cs_2CO_3 (0.75 mmol, 1.5 equiv.), and sealed with a rubber septum. The flask was then evacuated and purged with argon for 3 times. Corresponding carboxylic acid [if liquid] (0.75 mmol, 1.5 equiv.), acetonitrile (4.5 mL) and water (0.5 mL) were subsequently added under inert atmosphere. The reaction mixture was stirred for 24 h under irradiation at 396 nm. Subsequently, aqueous K_2CO_3 solution (10wt%, 15 mL) and brine (15 mL) were added, and the reaction mixture was extracted with dichloromethane (4 x 15 mL). The combined organic layers were washed with brine (15 mL) and dried over anhydrous Na_2SO_4, filtered and concentrated under reduced pressure. The residue was purified by column chromatography on silica gel using mixture of *n*-pentane and ethyl acetate as eluent.

General procedure B. A 10 mL Schlenk flask equipped with a magnetic stir bar was filled with benzoic acid (**2a**) (0.75 mmol, 1.5 equiv.), corresponding alkene [if solid] (0.5 mmol, 1.0 equiv.), BuPPT (5.0 mol%), Cs_2CO_3 (0.75 mmol, 1.5 equiv.), and sealed with a rubber septum. The flask was then evacuated and purged with argon for 3 times. Corresponding alkene [if liquid] (0.5 mmol, 1.0 equiv.), acetonitrile (4.5 mL) and water (0.5 mL) were subsequently added under inert atmosphere. The reaction mixture was stirred for 24 h under irradiation at 396 nm. Subsequently, aqueous K_2CO_3 solution (10wt%, 15 mL) and brine (15 mL) were added, and the reaction mixture was extracted with dichloromethane (4 x 15 mL). The combined organic layers were washed with brine (15 mL) and dried over anhydrous Na_2SO_4, filtered and concentrated under reduced pressure. The residue was purified by column chromatography on silica gel using mixture of *n*-pentane and ethyl acetate as eluent.

Scope of benzoic and alkenyl carboxylic acids

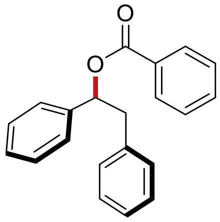

3a

Synthesis of 1,2-diphenylethyl benzoate (3a):

Compound **3a** was prepared following general procedure A. Purification by column chromatography (*n*-pentane:EtOAc = 50:1 → 20:1) yielded the title compound **3a** (135 mg, 0.45 mmol, 89%) as a colorless solid.

R$_f$ = 0.28 (*n*-pentane/EtOAc = 40:1, UV)

m.p. = 67–68 °C

^{1}H NMR (300 MHz, Chloroform-*d*) δ 8.06 – 8.02 (m, 2H), 7.54 – 7.48 (m, 1H), 7.43 – 7.11 (m, 12H), 6.18 (dd, *J* = 7.6, 6.0 Hz, 1H), 3.35 (dd, *J* = 13.8, 7.6 Hz, 1H), 3.18 (dd, *J* = 13.8, 6.0 Hz, 1H).

^{13}C NMR (75 MHz, Chloroform-*d*) δ 165.7, 140.2, 137.0, 133.0, 130.4, 129.7, 128.5, 128.4, 128.3, 128.1, 126.7, 126.6, 77.4, 43.3.

MS (EI): *m/z* (relative intensity) 211 (26), 180 (18), 165 (5), 105 (100), 91 (5), 77 (23).

HRMS (ESI-TOF, *m/z*): calcd. for $C_{21}H_{18}O_2$ [M+Na$^+$] 325.1204; found 325.1210.

IR (ATR, neat, cm^{-1}): 3060 (w), 3027 (w), 2942 (w), 2864 (w), 1703 (m), 1600 (w), 1494 (w), 1451 (w), 1268 (m), 1100 (m), 1070 (m), 1026 (m), 974 (m), 911 (w), 801 (w), 757 (m), 714 (s), 695 (s), 613 (m), 595 (w), 555 (s), 497 (m).

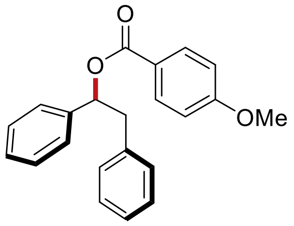

3b

Synthesis of 1,2-diphenylethyl 4-methoxybenzoate (3b):

Compound **3b** was prepared following general procedure A. Purification by column chromatography (*n*-pentane:EtOAc = 80:1 → 20:1) yielded the title compound **3b** (142.9 mg, 0.43 mmol, 86%) as a colorless solid.

R$_f$ = 0.42 (*n*-pentane/EtOAc = 15:1, UV)

m.p. = 85–87 °C

^{1}H NMR (300 MHz, Chloroform-*d*) δ 8.00 – 7.95 (m, 2H), 7.33 – 7.08 (m, 10H), 6.88 – 6.83 (m, 2H), 6.14 (dd, *J* = 7.6, 6.0 Hz, 1H), 3.75 (s, 3H), 3.31 (dd, *J* = 13.7, 7.6 Hz, 1H), 3.15 (dd, *J* = 13.7, 6.0 Hz, 1H).

^{13}C NMR (75 MHz, Chloroform-*d*) δ 165.4, 163.4, 140.3, 137.1, 131.7, 129.7, 128.4, 128.3, 127.9, 126.6, 126.5, 122.8, 113.7, 76.9, 55.4, 43.3.

MS (EI): *m*/*z* (relative intensity) 241 (8), 180 (8), 135 (100), 92 (5), 77 (8).

HRMS (ESI-TOF, *m/z*): calcd. for $C_{22}H_{20}O_3$ [M+Na$^+$] 355.1309; found 355.1307.

IR (ATR, neat, cm^{-1}): 3062 (w), 3030 (w), 2938 (w), 2860 (w), 1703 (s), 1604 (w), 1509 (w), 1453 (w), 1421 (w), 1265 (s), 1167 (m), 1113 (m), 1098 (s), 1025 (m), 976 (m), 911 (w), 851 (m), 759 (m), 744 (m), 699 (s), 627 (w), 583 (m), 551 (m), 509 (m).

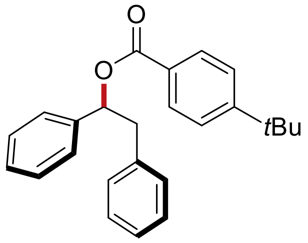

3c

Synthesis of 1,2-diphenylethyl 4-(tert-butyl)benzoate (3c):

Compound **3c** was prepared following general procedure A. Purification by column chromatography (*n*-pentane:EtOAc = 150:1 → 40:1) yielded the title compound **3c** (127 mg, 0.36 mmol, 71%) as a colorless solid.

R$_f$ = 0.44 (*n*-pentane/EtOAc = 40:1, UV)

m.p. = 74–75 °C

^{1}H NMR (300 MHz, Chloroform-*d*): δ 8.01 – 7.95 (m, 2H), 7.47 – 7.42 (m, 2H), 7.36 – 7.14 (m, 10H), 6.19 – 6.14 (m, 1H), 3.38 – 3.30 (m, 1H), 3.21 – 3.14 (m, 1H), 1.33 – 1.32 (m, 9H).

^{13}C NMR (75 MHz, Chloroform-*d*): δ 165.8, 156.7, 140.4, 137.1, 129.7, 129.6, 128.5, 128.4, 128.0, 127.7, 126.7, 126.6, 125.5, 77.2, 43.4, 35.2, 31.2.

MS (EI): *m*/*z* (relative intensity) 267 (8), 180 (9), 178 (8), 165 (7), 161 (100), 146 (13), 118 (20), 115 (9), 103 (8), 91 (59), 77 (10), 65 (8).

HRMS (ESI-TOF, *m*/*z*): calcd. for $C_{25}H_{26}O_2$ $[M+Na]^+$ 381.1830; found 381.1820.

IR (ATR, neat, cm^{-1}): 3029 (w), 2953 (w), 2862 (w), 1707 (m), 1607 (w), 1494 (w), 1453 (w), 1409 (w), 1363 (w), 1344 (w), 1267 (s), 1206 (w), 1187 (m), 1112 (m), 1096 (s), 1015 (m), 970 (m), 922 (w), 851 (m), 774 (m), 763 (m), 746 (m), 722 (w), 697 (s), 616 (w), 555 (m), 542 (m), 514 (m).

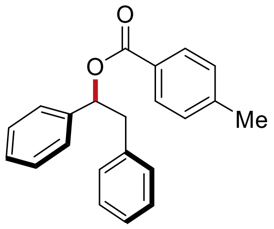

3d

Synthesis of 1,2-diphenylethyl 4-methylbenzoate (3d):

Compound **3d** was prepared following general procedure A. Purification by column chromatography (*n*-pentane:EtOAc = 150:1 → 40:1) yielded the title compound **3d** (147 mg, 0.47 mmol, 93%) as a colorless solid.

$\mathbf{R}_f$ = 0.53 (*n*-pentane/EtOAc = 40:1, UV)

m.p. = 79–80 °C

^{1}H NMR (400 MHz, Chloroform-*d*): δ 7.93 (d, *J* = 8.1 Hz, 2H), 7.35 – 7.12 (m, 12H), 6.18 – 6.15 (m, 1H), 3.34 (dd, *J* = 13.7, 7.6 Hz, 1H), 3.18 (dd, *J* = 13.7, 6.0 Hz, 1H), 2.39 (s, 3H).

^{13}C NMR (100 MHz, Chloroform-*d*): δ 165.8, 143.7, 140.3, 137.1, 129.8, 129.7, 129.2, 128.5, 128.3, 128.0, 127.7, 126.7, 126.6, 77.2, 43.3, 21.8.

MS (EI): *m*/*z* (relative intensity) 226 (6), 225 (54), 180 (37), 165 (24), 152 (8), 119 (99), 103 (12), 91 (100), 77 (22), 65 (51), 63 (11), 51 (9), 39 (8).

HRMS (ESI-TOF, *m*/*z*): calcd. for $C_{22}H_{20}O_2$ $[M+Na]^+$ 339.1360; found 339.1361.

IR (ATR, neat, cm^{-1}): 3029 (w), 2943 (w), 2919 (w), 2854 (w), 1712 (s), 1607 (w), 1494 (w), 1453 (w), 1361 (w), 1247 (s), 1207 (w), 1178 (m), 1090 (s), 1017 (m), 983 (m), 913 (w), 839 (m), 778 (w), 745 (s), 697 (s), 631 (w), 593 (w), 550 (s), 510 (m), 473 (m).

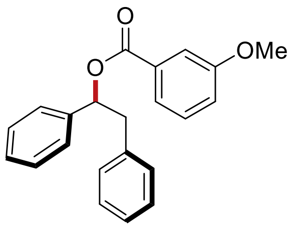

3e

Synthesis of 1,2-diphenylethyl 3-methoxybenzoate (3e):

Compound **3e** was prepared following general procedure A. Purification by column chromatography (*n*-pentane:EtOAc = 80:1 → 20:1) yielded the title compound **3e** (156 mg, 0.47 mmol, 94%) as a colorless oil.

R$_f$ = 0.26 (*n*-pentane/EtOAc = 40:1, UV)

^{1}H NMR (400 MHz, Chloroform-*d*): δ 7.67 – 7.64 (m, 1H), 7.55 (dd, *J* = 2.6, 1.5 Hz, 1H), 7.36 – 7.14 (m, 11H), 7.09 (ddd, *J* = 8.3, 2.7, 1.0 Hz, 1H), 6.17 (dd, *J* = 7.7, 6.0 Hz, 1H), 3.84 (s, 3H), 3.35 (dd, *J* = 13.8, 7.7 Hz, 1H), 3.20 (dd, *J* = 13.8, 6.0 Hz, 1H).

^{13}C NMR (100 MHz, Chloroform-*d*): δ 165.7, 159.6, 140.2, 137.0, 131.8, 129.7, 129.5, 128.5, 128.4, 128.1, 126.7, 126.6, 122.1, 119.5, 114.3, 77.5, 55.6, 43.3.

MS (EI): *m*/*z* (relative intensity) 241 (25), 180 (23), 178 (18), 165 (17), 152 (8), 135 (100), 107 (34), 103 (9), 91 (73), 77 (47), 65 (15), 51 (7).

HRMS (ESI-TOF, *m*/*z*): calcd. for $C_{22}H_{20}O_3$ [M+Na]$^+$ 355.1309; found 355.1301.

IR (ATR, neat, cm^{-1}): 3030 (w), 2940 (w), 2835 (w), 1715 (s), 1585 (m), 1487 (m), 1453 (m), 1431 (m), 1320 (w), 1271 (s), 1217 (s), 1180 (m), 1099 (m), 1073 (m), 1038 (s), 983 (m), 922 (w), 875 (w), 797 (w), 751 (s), 696 (s), 542 (m).

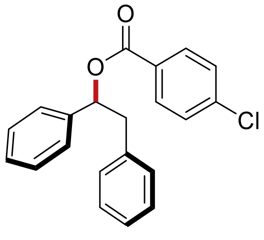

3f

Synthesis of 1,2-diphenylethyl 4-chlorobenzoate (3f):

Compound **3f** was prepared following general procedure A. Purification by column chromatography (*n*-pentane:EtOAc = 100:1 → 40:1) yielded the title compound **3f** (121 mg, 0.36 mmol, 72%) as a colorless solid.

R$_f$ = 0.22 (*n*-pentane/EtOAc = 40:1, UV)

m.p. = 98–100 °C

f^{1}H NMR (300 MHz, Chloroform-*d*) δ 7.99 – 7.95 (m, 2H), 7.42 – 7.38 (m, 2H), 7.36 – 7.18 (m, 8H), 7.16 – 7.11 (m, 2H), 6.17 (dd, *J* = 7.7, 6.0 Hz, 1H), 3.35 (dd, *J* = 13.8, 7.7 Hz, 1H), 3.20 (dd, *J* = 13.8, 6.0 Hz, 1H).

^{13}C NMR (75 MHz, Chloroform-*d*) δ 164.8, 139.9, 139.5, 136.9, 131.1, 129.7, 128.9, 128.8, 128.5, 128.4, 128.2, 126.7, 126.6, 77.7, 43.2.

MS (EI): *m/z* (relative intensity) 245 (24), 180 (24), 165 (13), 152 (5), 139 (100), 111 (37), 103 (7), 91 (42), 75 (17), 65 (12), 51 (6).

HRMS (ESI-TOF, *m/z*): calcd. for $C_{21}H_{17}ClO_2$ [M+Na$^+$] 359.0814; found 359.0810.

IR (ATR, neat, cm^{-1}): 3029 (w), 2916 (w), 2852 (w), 1716 (s), 1586 (w), 1487 (w), 1454 (w), 1400 (w), 1283 (w), 1254 (s), 1169 (w), 1090 (s), 1012 (m), 975 (m), 846 (m), 779 (w), 757 (s), 724 (m), 698 (s), 616 (w), 556 (s), 524 (m), 507 (s), 476 (m).

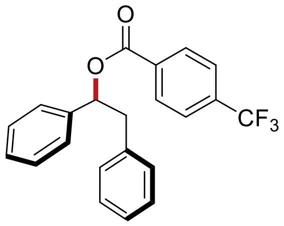

3g

Synthesis of 1,2-diphenylethyl 4-(trifluoromethyl) benzoate (3g):

Compound **3g** was prepared following general procedure A. Purification by column chromatography (*n*-pentane:EtOAc = 150:1 → 80:1) yielded the title compound **3g** (156 mg, 0.42 mmol, 84%) as a colorless solid.

R$_f$ = 0.25 (*n*-pentane/EtOAc = 40:1, UV)

m.p. = 54–56 °C

^{1}H NMR (300 MHz, Chloroform-*d*) δ 8.11 – 8.08 (m, 2H), 7.65 – 7.62 (m, 2H), 7.35 – 7.09 (m, 10H), 6.18 (dd, *J* = 7.8, 5.9 Hz, 1H), 3.33 (dd, *J* = 13.8, 7.8 Hz, 1H), 3.18 (dd, *J* = 13.8, 5.9 Hz, 1H).

^{13}C NMR (75 MHz, Chloroform-*d*) δ 164.5, 139.8, 136.8, 134.5 (q, *J* = 32.5 Hz, C–F), 133.7, 130.1, 129.6, 128.6, 128.4, 128.3, 126.8, 126.6, 125.5 (q, *J* = 3.8 Hz, C–F), 123.7 (q, *J* = 272.6 Hz, C–F), 78.0, 43.2.

^{19}F NMR (376 MHz, Chloroform-*d*) δ -63.1.

MS (EI): *m/z* (relative intensity) 279 (28), 180 (31), 173 (100), 165 (6), 145 (44), 91 (9), 77 (6).

HRMS (ESI-TOF, *m/z*): calcd. for $C_{22}H_{17}F_3O_2$ [M+Na$^+$] 393.1078; found 393.1080.

IR (ATR, neat, cm^{-1}): 3029 (w), 2928 (w), 2854 (w), 1721 (s), 1586 (w), 1496 (w), 1454 (w), 1411 (w), 1325 (s), 1267 (s), 1169 (s), 1114 (s), 1100 (s), 1064 (s), 1018 (s), 974 (m), 950 (m), 912 (w), 861 (s), 772 (m), 757 (m), 744 (m), 697 (s), 666 (w), 626 (w), 588 (w), 553 (s), 513 (m), 477 (m), 419 (w).

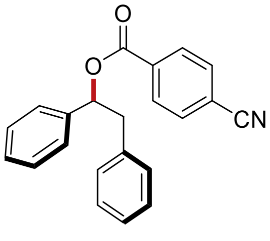

3h

Synthesis of 1,2-diphenylethyl 4-cyanobenzoate (3h):

Compound **3h** was prepared following general procedure A. Purification by column chromatography (*n*-pentane:EtOAc = 60:1 → 15:1) yielded the title compound **3h** (152 mg, 0.47 mmol, 93%) as a colorless solid.

R$_f$ = 0.18 (*n*-pentane/EtOAc = 40:1, UV)

m.p. = 114–116 °C

^{1}H NMR (300 MHz, Chloroform-*d*): δ 8.12 – 8.09 (m, 2H), 7.75 – 7.71 (m, 2H), 7.36 – 7.30 (m, 5H), 7.27 – 7.12 (m, 5H), 6.22 – 6.17 (m, 1H), 3.36 (dd, *J* = 13.8, 7.9 Hz, 1H), 3.22 (dd, *J* = 13.8, 6.0 Hz, 1H).

^{13}C NMR (75 MHz, Chloroform-*d*): δ 164.1, 139.6, 136.7, 134.3, 132.3, 130.2, 129.6, 128.7, 128.5, 128.5, 126.9, 126.7, 118.1, 116.5, 78.4, 43.1.

MS (EI): *m*/*z* (relative intensity) 236 (169), 180 (21), 165 (10), 130 (100), 102 (39), 91 (38), 77 (14), 65 (14), 63 (5), 51 (11).

IR (ATR, neat, cm^{-1}): 3028 (w), 2923 (w), 2233 (w), 1715 (m), 1494 (w), 1454 (w), 1406 (w), 1346 (w), 1272 (s), 1176 (w), 1105 (s), 1023 (m), 963 (m), 943 (m), 920 (w), 855 (m), 762 (s), 741 (s), 697 (s), 641 (w), 615 (m), 593 (w), 558 (m), 542 (s), 508 (m).

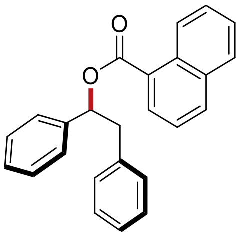

3i

Synthesis of 1,2-diphenylethyl 1-naphthoate (3i):

Compound **3i** was prepared following general procedure A. Purification by column chromatography (*n*-pentane:EtOAc = 200:1 → 150:1) yielded the title compound **3i** (173 mg, 0.49 mmol, 98%) as a colorless oil.

R$_f$ = 0.38 (*n*-pentane/EtOAc = 20:1, UV)

^{1}H NMR (300 MHz, Chloroform-*d*) δ 8.71 – 8.67 (m, 1H), 8.10 (dd, *J* = 7.3, 1.3 Hz, 1H), 7.92 – 7.89 (m, 1H), 7.78 – 7.75 (m, 1H), 7.49 – 7.35 (m, 5H), 7.32 – 7.11 (m, 8H), 6.29 (dd, *J* = 7.9, 5.9 Hz, 1H), 3.35 (dd, *J* = 13.8, 7.9 Hz, 1H), 3.21 (dd, *J* = 13.8, 5.9 Hz, 1H).

^{13}C NMR (75 MHz, Chloroform-*d*) δ 166.7, 140.3, 137.1, 133.8, 133.3, 131.4, 130.0, 129.7, 128.5, 128.5, 128.4, 128.1, 127.7, 127.4, 126.7, 126.7, 126.2, 125.9, 124.5, 77.5, 43.4.

MS (EI): *m*/*z* (relative intensity) 352 (1) [M$^+$], 261 (6), 178 (10), 165 (12), 155 (100), 127 (89), 103 (8), 91 (67), 77 (20), 65 (12), 51 (7).

HRMS (ESI-TOF, *m*/*z*): calcd. for $C_{25}H_{20}O_2$ [M+Na$^+$] 375.1355; found 375.1363.

IR (ATR, neat, cm^{-1}): 3060 (w), 3029 (w), 2920 (w), 1710 (s), 1592 (w), 1509 (w), 1495 (w), 1453 (w), 1345 (w), 1275 (m), 1236 (s), 1192 (s), 1129 (s), 1072 (m), 1030 (m), 1001 (s), 910 (w), 812 (w), 778 (s), 754 (m), 695 (s), 656 (m), 541 (m), 506 (m).

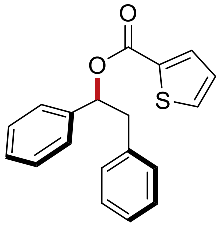

3j

Synthesis of 1,2-diphenylethyl thiophene-2-carboxylate (3j):

Compound **3j** was prepared following general procedure A. Purification by column chromatography (*n*-pentane:EtOAc = 80:1 → 40:1) yielded the title compound **3j** (119 mg, 0.39 mmol, 77%) as a colorless oil.

R$_f$ = 0.35 (*n*-pentane/EtOAc = 20:1, UV)

^{1}H NMR (300 MHz, Chloroform-*d*) δ 7.75 (dd, *J* = 3.7, 1.3 Hz, 1H), 7.46 (dd, *J* = 4.9, 1.3 Hz, 1H), 7.33 – 7.11 (m, 10H), 7.01 (dd, *J* = 4.9, 3.7 Hz, 1H), 6.10 (dd, *J* = 7.8, 5.8 Hz, 1H), 3.30 (dd, *J* = 13.8, 7.8 Hz, 1H), 3.13 (dd, *J* = 13.8, 5.8 Hz, 1H).

^{13}C NMR (75 MHz, Chloroform-*d*) δ 161.3, 139.9, 136.9, 133.9, 133.5, 132.5, 129.7, 128.5, 128.3, 128.1, 127.8, 126.7, 126.6, 77.5, 43.2.

MS (EI): *m*/*z* (relative intensity) 217 (17), 180 (14), 165 (12), 111 (100), 103 (7), 91 (36), 83 (10), 77 (13), 65 (12), 51 (6), 39 (15).

HRMS (ESI-TOF, *m*/*z*): calcd. for $C_{19}H_{16}O_2S$ [M+Na$^+$] 331.0763; found 331.0770.

IR (ATR, neat, cm^{-1}): 3063 (w), 3029 (w), 2921 (w), 1703 (s), 1603 (w), 1524 (w), 1495 (w), 1453 (w), 1415 (m), 1359 (m), 1252 (s), 1222 (m), 1089 (s), 1072 (s), 1031 (m), 983 (m), 910 (w), 860 (m), 812 (w), 745 (s), 721 (s), 695 (s), 542 (m), 506 (m).

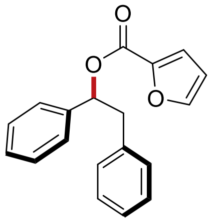

3k

Synthesis of 1,2-diphenylethyl furan-2-carboxylate (3k):

Compound **3k** was prepared following general procedure A. Purification by column chromatography (*n*-pentane:EtOAc = 150:1 → 40:1) yielded the title compound **3k** (57.0 mg, 0.20 mmol, 39%) as a colorless solid.

R$_f$ = 0.44 (*n*-pentane/EtOAc = 15:1, UV)

m.p. = 68–70 °C

^{1}H NMR (300 MHz, Chloroform-*d*) δ 7.54 (dd, *J* = 1.8, 0.9 Hz, 1H), 7.34 – 7.16 (m, 9H), 7.15 – 7.10 (m, 2H), 6.46 (dd, *J* = 3.5, 1.8 Hz, 1H), 6.15 (dd, *J* = 7.7, 6.1 Hz, 1H), 3.33 (dd, *J* = 13.8, 7.7 Hz, 1H), 3.16 (dd, *J* = 13.8, 6.1 Hz, 1H).

^{13}C NMR (75 MHz, Chloroform-*d*) δ 157.9, 146.5, 144.8, 139.8, 136.8, 129.7, 128.5, 128.4, 128.2, 126.7, 118.0, 111.9, 77.3, 43.1.

MS (EI): *m/z* (relative intensity) 201 (35), 180 (23), 165 (7), 95 (100), 91 (7), 77 (6).

HRMS (ESI-TOF, *m/z*): calcd. for $C_{19}H_{16}O_3$ [M+Na$^+$] 315.0992; found 315.0996.

IR (ATR, neat, cm^{-1}): 3120 (w), 3032 (w), 2904 (w), 1715 (s), 1602 (w), 1567 (w), 1494 (w), 1471 (m), 1455 (m), 1396 (m), 1319 (w), 1288 (s), 1237 (m), 1176 (s), 1116 (s), 1076 (m), 1046 (m), 1020 (m), 994 (m), 934 (m), 915 (w), 884 (w), 813 (w), 755 (s), 695 (s), 607 (m), 596 (m), 572 (m), 544 (s), 523 (m).

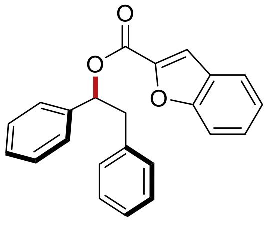

3l

Synthesis of 1,2-diphenylethyl benzofuran-2-carboxylate (3l):

Compound **3l** was prepared following general procedure A. Purification by column chromatography (*n*-pentane:EtOAc = 80:1 → 40:1) yielded the title compound **3l** (47.9 mg, 0.14 mmol, 28%) as a colorless solid.

$\mathbf{R_f}$ = 0.31 (*n*-pentane/EtOAc = 20:1, UV)

m.p. = 85–87 °C

^{1}H NMR (300 MHz, Chloroform-*d*) δ 7.68 – 7.64 (m, 1H), 7.59 – 7.55 (m, 1H), 7.52 (d, *J* = 1.0 Hz, 1H), 7.45 – 7.42 (m, 1H), 7.40 – 7.28 (m, 6H), 7.26 – 7.14 (m, 5H), 6.22 (dd, *J* = 7.7, 6.0 Hz, 1H), 3.38 (dd, *J* = 13.8, 7.7 Hz, 1H), 3.21 (dd, *J* = 13.8, 6.0 Hz, 1H).

^{13}C NMR (75 MHz, Chloroform-*d*) δ 158.8, 155.9, 145.7, 139.6, 136.7, 129.7, 128.6, 128.4, 128.3, 127.7, 127.0, 126.8, 126.8, 123.9, 122.9, 114.0, 112.5, 77.8, 43.1.

MS (EI): *m*/*z* (relative intensity) 251 (16), 180 (18), 165 (11), 145 (100), 103 (8), 91 (38), 89 (48), 77 (11), 63 (14).

HRMS (ESI-TOF, *m*/*z*): calcd. for $C_{23}H_{18}O_3$ [M+Na$^+$] 365.1148; found 365.1153.

IR (ATR, neat, cm^{-1}): 3062 (w), 3029 (w), 2931 (w), 1725 (m), 1561 (m), 1495 (w), 1446 (w), 1353 (w), 1299 (m), 1259 (w), 1208 (m), 1174 (s), 1145 (m), 1093 (m), 1050 (w), 1030 (w), 978 (m), 885 (w), 833 (w), 781 (w), 744 (s), 697 (s), 631 (m), 548 (s), 498 (m).

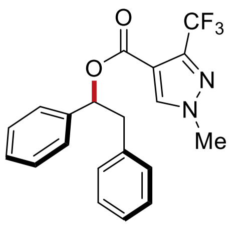

3m

Synthesis of 1,2-diphenylethyl 1-methyl-3-(trifluoromethyl)-1*H*-pyrazole-4-carboxylate (3m):

Compound **3m** was prepared following general procedure A. Purification by column chromatography (*n*-pentane:EtOAc = 40:1 → 5:1) yielded the title compound **3m** (165 mg, 0.44 mmol, 88%) as a colorless solid.

$\mathbf{R_f}$ = 0.24 (*n*-pentane/EtOAc = 5:1, $KMnO_4$)

m.p. = 95–97 °C

^{1}H NMR (400 MHz, Chloroform-*d*) δ 7.91 (s, 1H), 7.33 – 7.17 (m, 8H), 7.12 – 7.09 (m, 2H), 6.12 (dd, *J* = 7.7, 6.4 Hz, 1H), 3.91 (s, 3H), 3.32 (dd, *J* = 13.8, 7.7 Hz, 1H), 3.13 (dd, *J* = 13.8, 6.4 Hz, 1H).

^{13}**C NMR** (75 MHz, Chloroform-*d*) δ 159.9, 141.3 (q, *J* = 38.3 Hz, C–F), 139.7, 136.8, 136.7, 129.5, 128.4, 128.3, 128.2, 126.7, 126.6, 120.5 (q, *J* = 269.6 Hz, C–F), 113.2, 77.7, 43.0, 39.7.

^{19}**F NMR** (282 MHz, Chloroform-*d*) δ -61.4.

MS (EI): *m/z* (relative intensity) 283 (54), 177 (100), 165 (37), 155 (6), 152 (11), 149 (9), 130 (6), 115 (6), 110 (5), 103 (24), 91 (53), 77 (44), 65 (41), 51 (21), 43 (14), 39 (9).

HRMS (ESI-TOF, *m/z*): calcd. for $C_{20}H_{17}F_3N_2O_2$ [M+Na$^+$] 397.1139; found 397.1133.

IR (ATR, neat, cm^{-1}): 3138 (w), 3065 (w), 3026 (w), 2938 (w), 2852 (w), 1717 (m), 1543 (m), 1495 (w), 1453 (w), 1315 (m), 1298 (s), 1199 (m), 1170 (s), 1136 (s), 1054 (s), 991 (m), 914 (w), 890 (w), 781 (m), 757 (m), 696 (s), 628 (w), 572 (w), 545 (s), 433 (w).

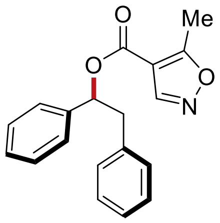

3n

Synthesis of 1,2-diphenylethyl 5-methylisoxazole-4-carboxylate (3n):

Compound **3n** was prepared following general procedure A. Purification by column chromatography (*n*-pentane:EtOAc = 70:1 → 20:1) yielded the title compound **3n** (63.0 mg, 0.21 mmol, 41%) as a colorless oil.

R*f* = 0.25 (*n*-pentane/EtOAc = 20:1, $KMnO_4$)

^{1}**H NMR** (300 MHz, Chloroform-*d*) δ 8.46 (q, *J* = 0.6, 1H), 7.34 – 7.18 (m, 8H), 7.12 – 7.08 (m, 2H), 6.12 (dd, *J* = 7.9, 6.0 Hz, 1H), 3.29 (dd, *J* = 13.8, 7.9 Hz, 1H), 3.17 (dd, *J* = 13.8, 6.0 Hz, 1H), 2.62 (d, *J* = 0.6, 3H).

^{13}**C NMR** (75 MHz, Chloroform-*d*) δ 174.4, 160.8, 150.2, 139.6, 136.7, 129.5, 128.6, 128.4, 128.3, 126.8, 126.6, 109.6, 77.4, 43.1, 12.7.

MS (EI): *m/z* (relative intensity) 216 (27), 180 (34), 165 (25), 152 (8), 110 (100), 103 (17), 91 (57), 77 (30), 65 (22), 51 (15), 43 (45), 39 (8).

HRMS (ESI-TOF, *m/z*): calcd. for $C_{19}H_{17}NO_3$ [M+H$^+$] 308.1287; found 308.1294.

IR (ATR, neat, cm^{-1}): 3064 (w), 3031 (w), 2924 (w), 1718 (s), 1612 (m), 1486 (m), 1454 (w), 1415 (m), 1394 (m), 1367 (w), 1271 (m), 1228 (s), 1155 (w), 1094 (s), 979 (m), 931 (m), 848 (w), 757 (s), 696 (s), 621 (w), 572 (w), 545 (m), 523 (m).

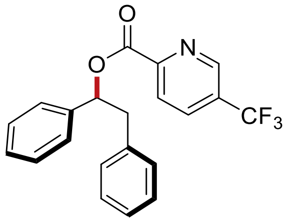

3o

Synthesis of 1,2-diphenylethyl 5-(trifluoromethyl)picolinate (3o):

Compound **3o** was prepared following general procedure B. Purification by column chromatography (*n*-pentane:EtOAc = 60:1 → 20:1) yielded the title compound **3o** (66.8 mg, 0.18 mmol, 36%) as a colorless solid.

$\mathbf{R}_f$ = 0.21 (*n*-pentane/EtOAc = 20:1, UV)

m.p. = 116–117 °C

^{1}H NMR (400 MHz, Chloroform-*d*) δ 9.02 (s, 1H), 8.17 (d, *J* = 8.2 Hz, 1H), 8.06 (dd, *J* = 8.2, 1.7 Hz, 1H), 7.42 – 7.39 (m, z2H), 7.36 – 7.28 (m, 3H), 7.25 – 7.15 (m, 5H), 6.27 (dd, *J* = 7.8, 6.2 Hz, 1H), 3.46 (dd, *J* = 13.8, 7.8 Hz, 1H), 3.26 (dd, *J* = 13.8, 6.2 Hz, 1H).

^{13}C NMR (75 MHz, Chloroform-*d*) δ 163.2, 151.3, 147.0 (q, *J* = 3.0 Hz, C–F), 139.3, 136.7, 134.4 (q, *J* = 3.4 Hz, C–F), 129.7, 129.3 (q, *J* = 33.0 Hz, C–F), 128.6, 128.5, 126.9, 126.8, 124.8, 123.1 (q, *J* = 273.3 Hz, C–F), 79.0, 42.9.

^{19}F NMR (282 MHz, Chloroform-*d*) δ -62.7.

MS (EI): *m/z* (relative intensity) 280 (99), 236 (10), 180 (47), 174 (100), 165 (29), 152 (11), 146 (99), 126 (35), 105 (17), 91 (67), 77 (30), 69 (7), 65 (21), 51 (12).

HRMS (ESI-TOF, *m/z*): calcd. for $C_{21}H_{16}F_3NO_2$ [M+Na$^+$] 394.1030; found 394.1024.

IR (ATR, neat, cm^{-1}): 3088 (w), 3031 (w), 2969 (w), 1736 (m), 1601 (w), 1578 (w), 1495 (w), 1456 (w), 1391 (w), 1325 (s), 1277 (m), 1242 (m), 1144 (s), 1117 (s), 1075 (s), 1013 (m), 964 (m), 882 (m), 802 (w), 777 (w), 759 (w), 746 (m), 696 (s), 615 (w), 554 (s), 512 (w), 474 (w).

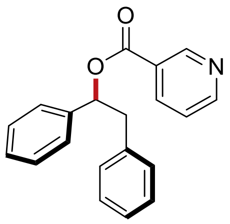

3p

Synthesis of 1,2-diphenylethyl nicotinate (3p):

Compound **3p** was prepared following general procedure A. Purification by column chromatography (*n*-pentane:EtOAc = 40:1 → 5:1) yielded the title compound **3p** (23.1 mg, 0.08 mmol, 15%) as a colorless solid.

R*f* = 0.29 (*n*-pentane/EtOAc = 4:1, $KMnO_4$)

m.p. = 81–83 °C

^{1}H NMR (300 MHz, Chloroform-*d*) δ 9.24 (dd, *J* = 2.1, 0.7 Hz, 1H), 8.76 (dd, *J* = 4.9, 1.7 Hz, 1H), 8.28 – 8.24 (m, 1H), 7.39 – 7.29 (m, 6H), 7.27 – 7.14 (m, 5H), 6.20 (dd, *J* = 7.9, 5.8 Hz, 1H), 3.36 (dd, *J* = 13.8, 7.9 Hz, 1H), 3.22 (dd, *J* = 13.8, 5.8 Hz, 1H).

^{13}C NMR (75 MHz, Chloroform-*d*) δ 164.4, 153.4, 150.9, 139.7, 137.3, 136.8, 129.7, 128.6, 128.5, 128.4, 126.8, 126.7, 126.4, 123.5, 78.0, 43.2.

MS (EI): *m*/*z* (relative intensity) 212 (23), 180 (24), 165 (15), 106 (100), 91 (64), 78 (59), 65 (19), 51 (38), 39 (6).

HRMS (ESI-TOF, *m*/*z*): calcd. for $C_{20}H_{17}NO_2$ [M+H$^+$] 304.1337; found 304.1335.

IR (ATR, neat, cm^{-1}): 3065 (w), 3020 (w), 2956 (w), 2867 (w), 1717 (s), 1591 (m), 1495 (w), 1454 (w), 1425 (w), 1342 (w), 1326 (w), 1271 (s), 1133 (m), 1108 (s), 1024 (s), 965 (m), 952 (m), 923 (m), 827 (w), 784 (w), 763 (m), 736 (s), 696 (s), 616 (m), 554 (s), 511 (m), 425 (w).

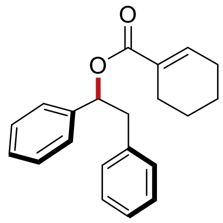

3q

Synthesis of 1,2-diphenylethyl cyclohex-1-ene-1-carboxylate (3q):

Compound **3q** was prepared following general procedure A. Purification by column chromatography (*n*-pentane:EtOAc = 100:1 → 80:1) yielded the title compound **3q** (73.5 mg, 0.24 mmol, 48%) as a colorless solid.

R*f* = 0.31 (*n*-pentane/EtOAc = 20:1, $KMnO_4$)

m.p. = 68–69 °C

^{1}H NMR (300 MHz, Chloroform-*d*) δ 7.32 – 7.14 (m, 8H), 7.10 – 7.05 (m, 2H), 7.02 – 6.99 (m, 1H), 5.98 (dd, *J* = 7.4, 6.2 Hz, 1H), 3.23 (dd, *J* = 13.7, 7.4 Hz, 1H), 3.07 (dd, *J* = 13.7, 6.2 Hz, 1H), 2.27 – 2.13 (m, 4H), 1.67 – 1.52 (m, 4H).

^{13}C NMR (75 MHz, Chloroform-*d*) δ 166.7, 140.5, 140.0, 137.2, 130.5, 129.7, 128.4, 128.2, 127.9, 126.6, 126.5, 76.5, 43.3, 25.9, 24.2, 22.1, 21.5.

MS (EI): *m*/*z* (relative intensity) 215 (26), 180 (23), 165 (20), 152 (5), 109 (100), 103 (11), 91 (41), 81 (33), 77 (22), 65 (13), 53 (12), 39 (7).

HRMS (ESI-TOF, *m*/*z*): calcd. for $C_{21}H_{22}O_2$ [M+Na$^+$] 329.1517; found 329.1513.

IR (ATR, neat, cm^{-1}): 3031 (w), 2926 (w), 2857 (w), 1707 (s), 1647 (m), 1494 (w), 1453 (w), 1418 (w), 1367 (w), 1272 (m), 1232 (s), 1089 (m), 1074 (m), 1039 (m), 984 (m), 952 (w), 921 (m), 784 (w), 743 (m), 696 (s), 626 (w), 617 (w), 593 (w), 559 (m), 540 (m), 509 (m).

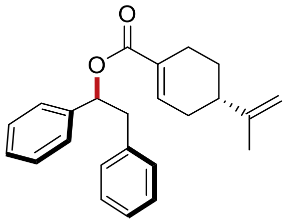

3r

Synthesis of 1,2-diphenylethyl (4S)-4-(prop-1-en-2-yl)cyclohex-1-ene-1-carboxylate (3r):

Compound **3r** was prepared following general procedure A. Purification by column chromatography (*n*-pentane:EtOAc = 150:1 → 60:1) yielded the title compound **3r** (106 mg, 0.31 mmol, 61%) as a colorless oil.

R$_f$ = 0.34 (*n*-pentane/EtOAc = 20:1, $KMnO_4$)

^{1}H NMR (400 MHz, Chloroform-*d*) δ 7.31 – 7.15 (m, 8H), 7.09 – 7.07 (m, 2H), 7.03 – 7.00 (m, 1H), 5.99 (ddd, *J* = 7.7, 6.1, 2.1 Hz, 1H), 4.75 (pent, *J* = 1.6 Hz, 1H), 4.71 – 4.70 (m, 1H), 3.22 (ddd, *J* = 13.7, 7.7, 1.1 Hz, 1H), 3.08 (ddd, *J* = 13.7, 6.1, 1.5 Hz, 1H), 2.50 – 2.41 (m, 1H), 2.35 – 2.02 (m, 4H), 1.89 – 1.83 (m, 1H), 1.73 (t, *J* = 1.1 Hz, 3H), 1.48 – 1.37 (m, 1H).

^{13}C NMR (100 MHz, Chloroform-*d*) δ 166.4, 166.4, 148.9, 140.5, 139.4, 137.1, 130.2, 129.7, 128.4, 128.2, 127.9, 126.6, 109.3, 76.6, 43.3, 40.1, 31.2, 27.1, 24.6, 20.8.

MS (EI): *m*/*z* (relative intensity) 255 (42), 180 (43), 165 (35), 149 (99), 131 (6), 121 (18), 105 (30), 91 (100), 77 (55), 65 (25), 53 (43), 39 (12).

HRMS (ESI-TOF, *m*/*z*): calcd. for $C_{24}H_{26}O_2$ [M+Na$^+$] 369.1830; found 369.1831.

IR (ATR, neat, cm^{-1}): 3062 (w), 3030 (w), 2920 (w), 1706 (s), 1648 (w), 1604 (w), 1495 (w), 1453 (w), 1435 (w), 1381 (w), 1240 (s), 1197 (m), 1074 (s), 1043 (m), 1004 (w), 924 (w), 888 (m), 757 (m), 739 (m), 696 (s), 542 (m).

Scope of alkyl carboxylic acids

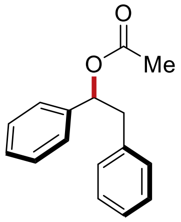

5a

Synthesis of 1,2-diphenylethyl acetate (5a):

Compound **5a** was prepared following general procedure B. Purification by column chromatography (*n*-pentane:EtOAc = 80:1 → 60:1) yielded the title compound **5a** (78.3 mg, 0.33 mmol, 65%) as a colorless oil.

R*f* = 0.24 (*n*-pentane/EtOAc = 40:1, $KMnO_4$)

^{1}H NMR (300 MHz, Chloroform-*d*) δ 7.34 – 7.15 (m, 8H), 7.11 – 7.07 (m, 2H), 5.94 (dd, *J* = 7.9, 6.0 Hz, 1H), 3.19 (dd, *J* = 13.7, 7.9 Hz, 1H), 3.04 (dd, *J* = 13.7, 6.0 Hz, 1H), 1.99 (s, 3H).

^{13}C NMR (75 MHz, Chloroform-*d*) δ 170.1, 140.1, 137.1, 129.6, 128.4, 128.3, 128.0, 126.7, 126.6, 76.7, 43.1, 21.2.

MS (EI): *m*/*z* (relative intensity) 180 (92), 165 (27), 152 (14), 149 (92), 115 (7), 107 (100), 103 (17), 91 (99), 77 (72), 65 (50), 51 (31), 43 (99), 39 (15).

HRMS (ESI-TOF, *m*/*z*): calcd. for $C_{16}H_{16}O_2$ [M+Na$^+$] 263.1047; found 263.1053.

IR (ATR, neat, cm^{-1}): 3064 (w), 3031 (w), 2926 (w), 1735 (m), 1604 (w), 1496 (w), 1454 (w), 1371 (w), 1230 (s), 1092 (w), 1074 (w), 1020 (m), 976 (w), 942 (w), 758 (m), 696 (s), 601 (w), 541 (m), 503 (w).

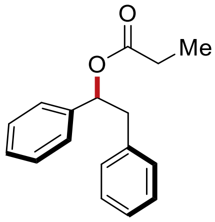

5b

Synthesis of 1,2-diphenylethyl propionate (5b):

Compound **5b** was prepared following general procedure A. Purification by column chromatography (*n*-pentane:EtOAc = 200:1 → 60:1) yielded the title compound **5b** (62.7 mg, 0.25 mmol, 49%) as a colorless oil.

R*f* = 0.32 (*n*-pentane/EtOAc = 20:1, $KMnO_4$)

^{1}H NMR (400 MHz, Chloroform-*d*) δ 7.31 – 7.14 (m, 8H), 7.10 – 7.07 (m, 2H), 5.95 (dd, *J* = 8.0, 5.9 Hz, 1H), 3.18 (dd, *J* = 13.7, 8.0 Hz, 1H), 3.04 (dd, *J* = 13.7, 5.9 Hz, 1H), 2.28 (q, *J* = 7.6 Hz, 2H), 1.05 (t, *J* = 7.6 Hz, 3H).

^{13}C NMR (100 MHz, Chloroform-*d*) δ 173.5, 140.3, 137.1, 129.6, 128.4, 128.3, 128.0, 126.6, 126.6, 76.4, 43.1, 27.8, 9.1.

MS (EI): *m*/*z* (relative intensity) 180 (45), 163 (63), 107 (13), 91 (17), 77 (15), 65 (6), 57 (100).

HRMS (ESI-TOF, *m*/*z*): calcd. for $C_{17}H_{18}O_2$ [M+Na$^+$] 277.1204; found 277.1210.

IR (ATR, neat, cm^{-1}): 3063 (w), 3030 (w), 2981 (w), 2941 (w), 1734 (s), 1604 (w), 1496 (w), 1454 (w), 1356 (w), 1272 (w), 1171 (s), 1078 (m), 1030 (w), 1002 (m), 911 (w), 806 (w), 757 (m), 696 (s), 639 (w), 540 (m), 507 (w).

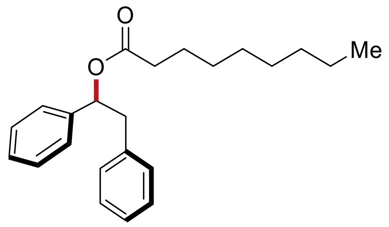

5c

Synthesis of 1,2-diphenylethyl nonanoate (5c):

Compound **5c** was prepared following general procedure A. Purification by column chromatography (*n*-pentane:EtOAc = 200:1 → 80:1) yielded the title compound **5c** (35.5 mg, 0.11 mmol, 21%) as a colorless oil.

R*f* = 0.43 (*n*-pentane/EtOAc = 20:1, $KMnO_4$)

^{1}H NMR (300 MHz, Chloroform-*d*) δ 7.34 – 7.16 (m, 8H), 7.12 – 7.08 (m, 2H), 5.96 (dd, *J* = 8.1, 6.0 Hz, 1H), 3.19 (dd, *J* = 13.7, 8.1 Hz, 1H), 3.05 (dd, *J* = 13.7, 6.0 Hz, 1H), 2.26 (t, *J* = 7.4 Hz, 2H), 1.53 (pent, *J* = 7.4 Hz, 2H), 1.34 – 1.15 (m, 10H), 0.88 (t, *J* = 6.8 Hz, 3H).

^{13}C NMR (75 MHz, Chloroform-*d*) δ 173.0, 140.4, 137.2, 129.6, 128.4, 128.3, 128.0, 126.7, 126.6, 76.4, 43.2, 34.6, 31.9, 29.3, 29.2, 29.2, 25.0, 22.8, 14.2.

MS (EI): *m*/*z* (relative intensity) 210 (8), 192 (100), 178 (14), 165 (13), 131 (9), 115 (24), 103 (9), 91 (8), 71 (7), 57 (11), 43 (14).

HRMS (ESI-TOF, *m*/*z*): calcd. for $C_{23}H_{30}O_2$ [M+Na$^+$] 361.2138; found 361.2141.

IR (ATR, neat, cm^{-1}): 3031 (w), 2924 (m), 2854 (w), 1735 (m), 1604 (w), 1496 (w), 1454 (w), 1376 (w), 1242 (w), 1156 (m), 1106 (w), 1074 (w), 1048 (w), 1030 (w), 990 (w), 757 (m), 696 (s), 639 (w), 541 (m).

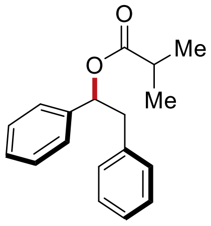

5d

Synthesis of 1,2-diphenylethyl isobutyrate (5d):

Compound **5d** was prepared following general procedure B. Purification by column chromatography (*n*-pentane:EtOAc = 200:1 → 60:1) yielded the title compound **5d** (51.0 mg, 0.19 mmol, 38%) as a colorless oil.

$\mathbf{R}_f$ = 0.32 (*n*-pentane/EtOAc = 20:1, $KMnO_4$)

^{1}H NMR (400 MHz, Chloroform-*d*) δ 7.33 – 7.16 (m, 8H), 7.13 – 7.09 (m, 2H), 5.95 (dd, *J* = 8.2, 5.7 Hz, 1H), 3.17 (dd, *J* = 13.8, 8.2 Hz, 1H), 3.06 (dd, *J* = 13.8, 5.7 Hz, 1H), 2.50 (hept, *J* = 7.0 Hz, 1H), 1.08 (d, *J* = 7.0, 3H), 1.07 (d, *J* = 7.0, 3H).

^{13}C NMR (100 MHz, Chloroform-*d*) δ 176.1, 140.5, 137.2, 129.7, 128.5, 128.3, 127.9, 126.6, 126.5, 76.3, 43.3, 34.2, 19.0, 18.9.

MS (EI): *m/z* (relative intensity) 180 (57), 177 (65), 165 (42), 152 (16), 115 (8), 107 (11), 103 (31), 91 (99), 77 (63), 71 (100), 65 (45), 51 (24), 43 (99), 39 (28).

HRMS (ESI-TOF, *m/z*): calcd. for $C_{18}H_{20}O_2$ [M+Na$^+$] 291.1360; found 291.1364.

IR (ATR, neat, cm^{-1}): 3064 (w), 3031 (w), 2973 (w), 2933 (w), 2874 (w), 1731 (s), 1604 (w), 1496 (w), 1468 (w), 1454 (w), 1386 (w), 1340 (w), 1252 (w), 1188 (m), 1149 (s), 1069 (m), 1030 (w), 989 (w), 841 (w), 756 (m), 696 (s), 639 (w), 541 (m).

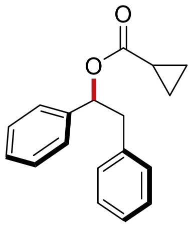

5e

Synthesis of 1,2-diphenylethyl cyclopropanecarboxylate (5e):

Compound **5e** was prepared following general procedure B. Purification by column chromatography (*n*-pentane:EtOAc = 150:1 → 60:1) yielded the title compound **5e** (105.2 mg, 0.40 mmol, 79%) as a colorless solidifying oil.

R*f* = 0.37 (*n*-pentane/EtOAc = 20:1, $KMnO_4$)

m.p. = 40–42 °C

^{1}H NMR (300 MHz, Chloroform-*d*) δ 7.29 – 7.16 (m, 8H), 7.09 – 7.05 (m, 2H), 5.93 (dd, *J* = 7.7, 6.1 Hz, 1H), 3.18 (dd, *J* = 13.7, 7.7 Hz, 1H), 3.04 (dd, *J* = 13.7, 6.1 Hz, 1H), 1.59 (tt, *J* = 8.0, 4.7 Hz, 1H), 0.96 – 0.85 (m, 2H), 0.83 – 0.71 (m, 2H).

^{13}C NMR (75 MHz, Chloroform-*d*) δ 173.9, 140.2, 137.0, 129.6, 128.4, 128.2, 127.9, 126.6, 126.6, 76.5, 43.1, 13.2, 8.4, 8.4.

MS (EI): *m*/*z* (relative intensity) 180 (22), 175 (26), 165 (14), 103 (10), 91 (41), 77 (21), 69 (100), 65 (18), 51 (10), 41 (35).

HRMS (ESI-TOF, *m*/*z*): calcd. for $C_{18}H_{18}O_2$ [M+Na$^+$] 289.1199; found 289.1201.

IR (ATR, neat, cm^{-1}): 3063 (w), 3030 (w), 2924 (w), 1723 (s), 1604 (w), 1496 (w), 1454 (w), 1391 (m), 1360 (w), 1263 (m), 1163 (s), 1100 (w), 1064 (m), 1028 (m), 991 (w), 949 (w), 906 (w), 855 (w), 823 (w), 757 (m), 744 (m), 696 (s), 541 (m), 504 (w).

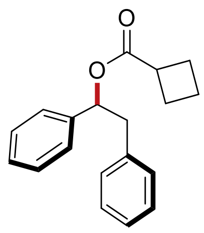

5f

Synthesis of 1,2-diphenylethyl cyclobutanecarboxylate (5f):

Compound **5f** was prepared following general procedure A. Purification by column chromatography (*n*-pentane:EtOAc = 150:1 →80:1) yielded the title compound **5f** (37.8 mg, 0.14 mmol, 27%) as a colorless oil.

R*f* = 0.36 (*n*-pentane/EtOAc = 20:1, $KMnO_4$)

^{1}H NMR (300 MHz, Chloroform-*d*) δ 7.32 – 7.18 (m, 8H), 7.13 – 7.09 (m, 2H), 5.95 (dd, *J* = 8.2, 5.8 Hz, 1H), 3.21 – 3.02 (m, 3H), 2.24 – 2.07 (m, 4H), 1.99 – 1.77 (m, 2H).

^{13}C NMR (75 MHz, Chloroform-*d*) δ 174.5, 140.5, 137.2, 129.7, 128.5, 128.3, 128.0, 126.6, 126.6, 76.3, 43.2, 38.3, 25.3, 25.0, 18.5.

MS (EI): *m/z* (relative intensity) 189 (27), 180 (23), 165 (17), 152 (6), 103 (13), 91 (44), 83 (66), 77 (22), 65 (17), 55 (100), 39 (11), 29 (8).

HRMS (ESI-TOF, *m/z*): calcd. for $C_{19}H_{20}O_2$ [M+Na$^+$] 303.1355; found 303.1364.

IR (ATR, neat, cm^{-1}): 3086 (w), 3064 (w), 3030 (w), 2986 (w), 2946 (w), 2866 (w), 1726 (s), 1604 (w), 1496 (w), 1454 (w), 1355 (w), 1249 (m), 1162 (s), 1108 (w), 1053 (m), 1030 (m), 987 (w), 911 (w), 756 (m), 696 (s), 637 (w), 540 (m).

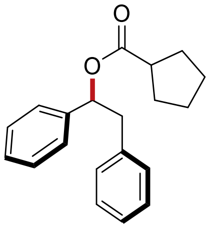

5g

Synthesis of 1,2-diphenylethyl cyclopentanecarboxylate (**5g**):

Compound **5g** was prepared following general procedure A. Purification by column chromatography (*n*-pentane:EtOAc = 150:1 → 80:1) yielded the title compound **5g** (30.9 mg, 0.11 mmol, 21%) as a colorless oil.

R$_f$ = 0.33 (*n*-pentane/EtOAc = 40:1, $KMnO_4$)

^{1}H NMR (300 MHz, Chloroform-*d*) δ 7.32 – 7.19 (m, 8H), 7.13 – 7.09 (m, 2H), 5.95 (dd, *J* = 8.2, 5.8 Hz, 1H), 3.17 (dd, *J* = 13.8, 8.2 Hz, 1H), 3.06 (dd, *J* = 13.8, 5.8 Hz, 1H), 2.76 – 2.65 (m, 1H), 1.86 – 1.46 (m, 8H).

^{13}C NMR (75 MHz, Chloroform-*d*) δ 175.8, 140.6, 137.3, 129.7, 128.5, 128.3, 127.9, 126.6, 126.5, 76.3, 44.0, 43.3, 30.0, 29.8, 25.9, 25.8.

MS (EI): *m/z* (relative intensity) 203 (28), 178 (23), 165 (27), 152 (7), 105 (11), 103 (18), 97 (53), 91 (73), 77 (21), 69 (100), 65 (27), 41 (46).

HRMS (ESI-TOF, *m/z*): calcd. for $C_{20}H_{22}O_2$ [M+Na$^+$] 317.1512; found 317.1519.

IR (ATR, neat, cm^{-1}): 3030 (w), 2954 (w), 2870 (w), 1728 (s), 1604 (w), 1496 (w), 1453 (w), 1372 (w), 1253 (w), 1146 (s), 1075 (w), 1047 (w), 993 (m), 909 (w), 756 (m), 696 (s), 543 (m).

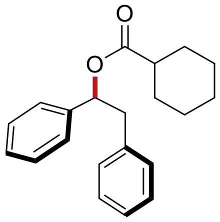

5h

Synthesis of 1,2-diphenylethyl cyclohexanecarboxylate (5h):

Compound **5h** was prepared following general procedure A. Purification by column chromatography (*n*-pentane:EtOAc = 150:1 → 80:1) yielded the title compound **5h** (33.9 mg, 0.11 mmol, 22%) as a colorless oil.

$\mathbf{R}_f$ = 0.33 (*n*-pentane/EtOAc = 40:1, $KMnO_4$)

^{1}**H NMR** (300 MHz, Chloroform-*d*) δ 7.31 – 7.18 (m, 8H), 7.12 – 7.08 (m, 2H), 5.95 (dd, *J* = 8.1, 5.8 Hz, 1H), 3.17 (dd, *J* = 13.8, 8.1 Hz, 1H), 3.05 (dd, *J* = 13.8, 5.8 Hz, 1H), 2.31 – 2.21 (m, 1H), 1.87 – 1.77 (m, 2H), 1.73 – 1.65 (m, 2H), 1.64 – 1.57 (m, 1H), 1.41 – 1.14 (m, 5H).

^{13}**C NMR** (75 MHz, Chloroform-*d*) δ 175.1, 140.5, 137.2, 129.7, 128.4, 128.3, 128.0, 126.6, 126.5, 76.1, 43.3, 43.3, 29.0, 28.9, 25.9, 25.5, 25.5.

MS (EI): *m/z* (relative intensity) 217 (27), 179 (36), 165 (35), 152 (8), 111 (55), 103 (20), 91 (81), 83 (100), 77 (29), 65 (23), 55 (60), 51 (10), 41 (31), 29 (6).

HRMS (ESI-TOF, *m/z*): calcd. for $C_{21}H_{24}O_2$ [M+Na$^+$] 331.1668; found 331.1676.

IR (ATR, neat, cm^{-1}): 3030 (w), 2929 (m), 2854 (w), 1729 (s), 1604 (w), 1496 (w), 1452 (m), 1377 (w), 1310 (w), 1245 (m), 1163 (s), 1129 (m), 1074 (w), 1030 (m), 895 (w), 846 (w), 755 (m), 696 (s), 542 (m).

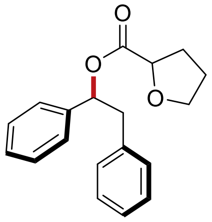

5i

Synthesis of 1,2-diphenylethyl tetrahydrofuran-2-carboxylate (5i):

Compound **5i** was prepared following general procedure A. Purification by column chromatography (*n*-pentane:EtOAc = 70:1 → 10:1) yielded the title compound **5i** (34.1 mg, 0.12 mmol, 23%) as a colorless oil.

$\mathbf{R}_f$ = 0.45 (*n*-pentane/EtOAc = 10:1, $KMnO_4$)

^{1}H NMR (300 MHz, Chloroform-*d*) δ 7.32 – 7.19 (m, 8H), 7.17 – 7.09 (m, 2H), 6.07 – 5.98 (m, 1H), 4.45 – 4.38 (m, 1H), 3.98 – 3.82 (m, 2H), 3.25 – 3.17 (m, 1H), 3.14 – 3.06 (m, 1H), 2.24 – 2.04 (m, 1H), 1.87 – 1.64 (m, 3H).

^{13}C NMR (75 MHz, Chloroform-*d*) δ 172.6, 172.6, 139.9, 139.8, 137.0, 136.9, 129.6, 129.5, 128.5, 128.4, 128.2, 128.1, 126.7, 126.7, 126.6, 77.0, 76.8, 69.4, 69.3, 43.1, 42.9, 30.1, 25.2, 25.1.

MS (EI): *m/z* (relative intensity) 181 (39), 165 (23), 152 (6), 103 (17), 91 (29), 77 (21), 71 (100), 65 (18), 51 (8), 43 (32), 39 (15), 29 (6).

HRMS (ESI-TOF, *m/z*): calcd. for $C_{19}H_{20}O_3$ [M+Na$^+$] 319.1304; found 319.1299.

IR (ATR, neat, cm^{-1}): 3063 (w), 3030 (w), 2952 (w), 2876 (w), 1745 (m), 1604 (w), 1496 (w), 1454 (w), 1270 (w), 1169 (m), 1083 (s), 986 (m), 926 (w), 757 (m), 696 (s), 541 (m).

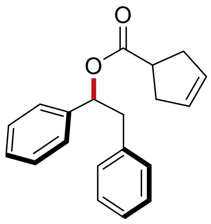

5j

Synthesis of 1,2-diphenylethyl cyclopent-3-ene-1-carboxylate (5j):

Compound **5j** was prepared following general procedure A. Purification by column chromatography (*n*-pentane:EtOAc = 150:1 → 60:1) yielded the title compound **5j** (21.9 mg, 0.08 mmol, 15%) as a colorless oil.

R$_f$ = 0.33 (*n*-pentane/EtOAc = 20:1, $KMnO_4$)

^{1}H NMR (300 MHz, Chloroform-*d*) δ 7.33 – 7.19 (m, 8H), 7.14 – 7.10 (m, 2H), 5.97 (dd, *J* = 8.3, 5.8 Hz, 1H), 5.64 – 5.58 (m, 2H), 3.19 (dd, *J* = 13.8, 8.3 Hz, 1H), 3.14 – 3.03 (m, 2H), 2.63 – 2.43 (m, 4H).

^{13}C NMR (75 MHz, Chloroform-*d*) δ 175.2, 140.4, 137.2, 129.7, 129.1, 129.0, 128.5, 128.3, 128.0, 126.7, 126.6, 76.6, 43.2, 41.8, 36.3, 36.1.

MS (EI): *m/z* (relative intensity) 201 (26), 180 (32), 165 (26), 152 (7), 103 (21), 95 (43), 91 (59), 77 (28), 67 (100), 51 (13), 39 (26).

HRMS (ESI-TOF, *m/z*): calcd. for $C_{20}H_{20}O_2$ [M+Na$^+$] 315.1355; found 315.1361.

IR (ATR, neat, cm^{-1}): 3061 (w), 3030 (w), 2923 (w), 2855 (w), 1730 (s), 1604 (w), 1496 (w), 1453 (w), 1345 (w), 1256 (w), 1189 (m), 1154 (s), 1073 (w), 1001 (m), 756 (m), 696 (s), 541 (m).

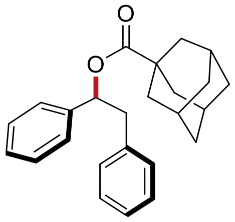

5k

Synthesis of 1,2-diphenylethyl (1s,3s)-adamantane-1-carboxylate (5k):

Compound **5k** was prepared following general procedure A. Purification by column chromatography (*n*-pentane:EtOAc = 80:1 → 60:1) yielded the title compound **5k** (66.7 mg, 0.19 mmol, 37%) as a colorless solid.

R$_f$ = 0.30 (*n*-pentane/EtOAc = 40:1, $KMnO_4$)

m.p. = 69–71 °C

^{1}H NMR (400 MHz, Chloroform-*d*) δ 7.32 – 7.16 (m, 8H), 7.12 – 7.09 (m, 2H), 5.92 (dd, *J* = 8.2, 5.6 Hz, 1H), 3.15 (dd, *J* = 13.8, 8.2 Hz, 1H), 3.05 (dd, *J* = 13.8, 5.6 Hz, 1H), 1.99 – 1.96 (m, 3H), 1.81 – 1.80 (m, 6H), 1.73 – 1.63 (m, 6H).

^{13}C NMR (100 MHz, Chloroform-*d*) δ 176.6, 140.8, 137.2, 129.7, 128.4, 128.2, 127.8, 126.6, 126.3, 75.9, 43.4, 40.8, 38.8, 36.6, 28.0.

MS (EI): *m/z* (relative intensity) 269 (21), 180 (52), 163 (42), 135 (100), 107 (24), 103 (25), 91 (99), 79 (50), 65 (19), 55 (7), 53 (5), 41 (8).

HRMS (ESI-TOF, *m/z*): calcd. for $C_{25}H_{28}O_2$ [M+Na$^+$] 383.1986; found 383.1984.

IR (ATR, neat, cm^{-1}): 3028 (w), 2895 (m), 2852 (m), 1709 (s), 1603 (w), 1493 (w), 1453 (m), 1345 (w), 1323 (w), 1269 (w), 1225 (s), 1182 (m), 1104 (w), 1070 (s), 1026 (m), 983 (m), 963 (m), 915 (w), 899 (w), 813 (w), 759 (s), 740 (s), 695 (s), 613 (w), 585 (w), 548 (s), 517 (m), 443 (w).

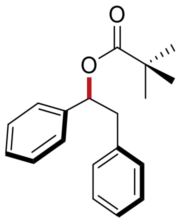

5l

Synthesis of 1,2-diphenylethyl pivalate (5l):

Compound **5l** was prepared following general procedure A. Purification by column chromatography (*n*-pentane:EtOAc = 150:1 → 40:1) yielded the title compound **5l** (55.1 mg, 0.20 mmol, 39%) as a colorless oil.

R$_f$ = 0.22 (*n*-pentane/EtOAc = 40:1, $KMnO_4$)

^{1}H NMR (300 MHz, Chloroform-*d*) δ 7.33 – 7.18 (m, 8H), 7.14 – 7.09 (m, 2H), 5.92 (dd, *J* = 8.3, 5.6 Hz, 1H), 3.15 (dd, *J* = 13.8, 8.3 Hz, 1H), 3.06 (dd, *J* = 13.8, 5.6 Hz, 1H), 1.11 (s, 9H).

^{13}C NMR (75 MHz, Chloroform-*d*) δ 177.5, 140.7, 137.2, 129.7, 128.5, 128.3, 127.9, 126.6, 126.3, 76.3, 43.4, 38.8, 27.1.

MS (EI): *m/z* (relative intensity) 191 (57), 180 (67), 165 (48), 152 (13), 115 (7), 107 (61), 103 (37), 91 (78), 85 (61), 77 (48), 65 (29), 57 (100), 51 (16), 41 (61), 29 (18).

HRMS (ESI-TOF, *m/z*): calcd. for $C_{19}H_{22}O_2$ [M+Na$^+$] 305.1517; found 305.1520.

IR (ATR, neat, cm^{-1}): 3064 (w), 3031 (w), 2971 (w), 2931 (w), 2871 (w), 1727 (s), 1604 (w), 1496 (w), 1479 (w), 1454 (w), 1396 (w), 1366 (w), 1280 (m), 1145 (s), 1074 (w), 1031 (m), 991 (w), 912 (w), 756 (m), 696 (s), 638 (w), 543 (m), 525 (w).

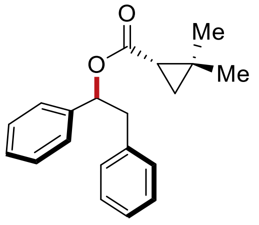

5m

Synthesis of 1,2-diphenylethyl (1*S*)-2,2-dimethylcyclopropane-1-carboxylate (5m):

Compound **5m** was prepared following general procedure B. Purification by column chromatography (*n*-pentane:EtOAc = 150:1 → 80:1) yielded the title compound **5m** (81.0 mg, 0.28 mmol, 55%) as a colorless oil.

R$_f$ = 0.30 (*n*-pentane/EtOAc = 40:1, $KMnO_4$)

^{1}H NMR (300 MHz, Chloroform-*d*) δ 7.33 – 7.18 (m, 8H), 7.16 – 7.06 (m, 2H), 6.01 – 5.91 (m, 1H), 3.26 – 3.17 (m, 1H), 3.09 – 3.02 (m, 1H), 1.57 – 1.48 (m, 1H), 1.13 (d, *J* = 2.2 Hz, 3H), 1.04 – 0.95 (m, 4H), 0.83 – 0.76 (m, 1H).

^{13}C NMR (75 MHz, Chloroform-*d*) δ 172.1, 172.0, 140.6, 140.4, 137.3, 137.2, 129.7, 128.4, 128.3, 128.3, 128.3, 128.0, 127.8, 126.8, 126.6, 126.6, 126.5, 76.7, 76.4, 43.3, 43.2, 27.2, 27.1, 27.1, 23.3, 23.3, 22.1, 18.8, 18.7.

MS (EI): *m/z* (relative intensity) 203 (19), 181 (48), 165 (37), 152 (7), 103 (26), 97 (100), 91 (68), 77 (29), 65 (23), 51 (14), 41 (46).

HRMS (ESI-TOF, *m/z*): calcd. for $C_{20}H_{22}O_2$ [M+Na$^+$] 317.1512; found 317.1518.

IR (ATR, neat, cm^{-1}): 3064 (w), 3031 (w), 2950 (w), 2872 (w), 1722 (s), 1604 (w), 1496 (w), 1454 (w), 1396 (m), 1376 (w), 1309 (w), 1266 (m), 1154 (s), 1120 (m), 1082 (m), 1031 (w), 993 (w), 910 (w), 829 (w), 756 (m), 696 (s), 541 (m).

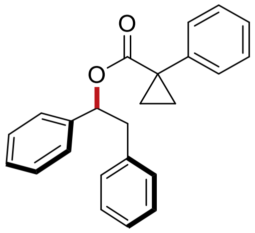

5n

Synthesis of 1,2-diphenylethyl 1-phenylcyclopropane-1-carboxylate (5n):

Compound **5n** was prepared following general procedure B. Purification by column chromatography (*n*-pentane:EtOAc = 150:1 → 60:1) yielded the title compound **5n** (27.9 mg, 0.08 mmol, 16%) as a colorless oil.

$\mathbf{R}_f$ = 0.31 (*n*-pentane/EtOAc = 20:1, $KMnO_4$)

^{1}H NMR (300 MHz, Chloroform-*d*) δ 7.36 – 7.23 (m, 8H), 7.21 – 7.16 (m, 3H), 7.11 – 7.07 (m, 2H), 6.92 – 6.87 (m, 2H), 5.88 – 5.83 (m, 1H), 2.96 – 2.93 (m, 2H), 1.54 – 1.42 (m, 2H), 1.19 – 1.07 (m, 2H).

^{13}C NMR (75 MHz, Chloroform-*d*) δ 173.6, 140.5, 139.6, 137.0, 130.8, 129.8, 128.4, 128.2, 127.8, 127.2, 126.5, 126.2, 77.2, 43.4, 29.4, 16.7, 16.3.

MS (EI): *m*/*z* (relative intensity) 251 (15), 180 (18), 165 (18), 145 (53), 117 (100), 103 (18), 91 (90), 77 (22), 65 (18), 51 (9), 39 (6).

HRMS (ESI-TOF, *m*/*z*): calcd. for $C_{24}H_{22}O_2$ [M+Na$^+$] 365.1512; found 365.1517.

IR (ATR, neat, cm^{-1}): 3086 (w), 3060 (w), 3029 (w), 2921 (w), 1719 (m), 1603 (w), 1496 (w), 1453 (w), 1280 (m), 1165 (s), 1088 (m), 1027 (w), 995 (w), 952 (w), 753 (m), 695 (s), 640 (w), 546 (m).

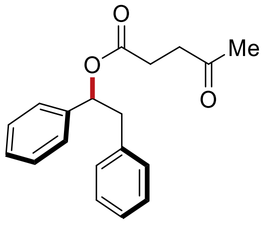

5o

Synthesis of 1,2-diphenylethyl 4-oxopentanoate (5o):

Compound **5o** was prepared following general procedure B. Purification by column chromatography (*n*-pentane:EtOAc = 30:1 → 5:1) yielded the title compound **5o** (87.4 mg, 0.30 mmol, 59%) as a yellow oil.

R$_f$ = 0.41 (*n*-pentane/EtOAc = 5:1, UV)

^{1}H NMR (300 MHz, Chloroform-*d*) δ 7.32 – 7.17 (m, 8H), 7.10 – 7.05 (m, 2H), 5.92 (dd, *J* = 7.7, 6.2 Hz, 1H), 3.19 (dd, *J* = 13.7, 7.7 Hz, 1H), 3.05 (dd, *J* = 13.7, 6.2 Hz, 1H), 2.66 – 2.60 (m, 2H), 2.58 – 2.52 (m, 2H), 2.11 (s, 3H).

^{13}C NMR (75 MHz, Chloroform-*d*) δ 206.5, 171.9, 139.9, 136.9, 129.6, 128.4, 128.3, 128.0, 126.6, 126.6, 77.0, 43.0, 37.9, 29.8, 28.2.

MS (EI): *m*/*z* (relative intensity) 209 (35), 192 (100), 178 (21), 165 (21), 152 (8), 131 (7), 115 (32), 103 (15), 99 (73), 91 (16), 77 (9), 71 (9), 43 (60).

HRMS (ESI-TOF, *m*/*z*): calcd. for $C_{19}H_{20}O_3$ [M+Na$^+$] 319.1309; found 319.1304.

IR (ATR, neat, cm^{-1}): 3063 (w), 3030 (w), 2921 (w), 2855 (w), 1717 (s), 1603 (w), 1495 (w), 1454 (w), 1407 (w), 1359 (m), 1206 (m), 1152 (s), 1073 (w), 1049 (w), 1029 (w), 986 (w), 759 (m), 698 (s), 666 (w), 541 (m), 447 (w).

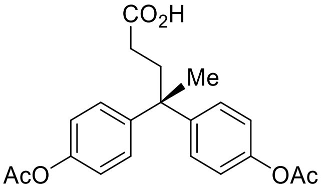

4p

Synthesis of 4,4-bis(4-acetoxyphenyl) pentanoic acid (4p):

To a suspension of diphenolic acid (1.5 g, 5.25 mmol) in acetic anhydride (3 mL) were added 6 drops of concentrated sulfuric acid and the reaction mixture was heated at 80 °C for 2 h. The reaction mixture was allowed to cool to ambient temperature and brine (20 mL) was added. The crude reaction mixture was extracted with dichloromethane (3 x 30 mL). The combined organic phases were washed with brine (20 mL) and dried over Na_2SO_4, filtered, and concentrated under reduced pressure. The resulting crude

product was purified by column chromatography (CH_2Cl_2:EtOAc = 7:1 → 3:1) to yield the title compound **4p** (1.68 g, 86 %) as a colorless solid.

R$_f$ = 0.35 (*n*-pentane/EtOAc = 1:1, UV)

m.p. = 152–154 °C

^{1}H NMR (400 MHz, Chloroform-*d*) δ 10.33 (brs, 1H), 7.21 – 7.17 (m, 4H), 7.02 – 6.98 (m, 4H), 2.46 – 2.41 (m, 2H), 2.28 (s, 6H), 2.17 – 2.13 (m, 2H), 1.61 (s, 3H).

^{13}C NMR (100 MHz, Chloroform-*d*) δ 179.7, 169.6, 148.9, 145.7, 128.4, 121.3, 45.3, 36.3, 30.0, 27.8, 21.3.

MS (EI): *m*/*z* (relative intensity) 370 (15) [M$^+$], 328 (17), 297 (79), 271 (9), 255 (94), 213 (100), 169 (8), 119 (16), 99 (8), 43 (51).

HRMS (ESI-TOF, *m*/*z*): calcd. for $C_{21}H_{22}O_6$ [M+Na$^+$] 393.1308; found 393.1319.

IR (ATR, neat, cm^{-1}): 3040 (w), 2980 (w), 2941 (w), 1751 (m), 1706 (m), 1501 (m), 1428 (w), 1368 (m), 1315 (w), 1197 (s), 1165 (s), 1007 (m), 912 (m), 850 (m), 797 (m), 660 (w), 610 (w), 594 (w), 564 (m), 519 (w).

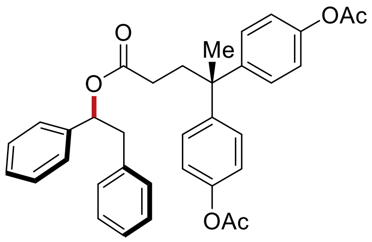

5p

Synthesis of (5-(1,2-diphenylethoxy)-5-oxopentane-2,2-diyl)bis(4,1-phenylene) diacetate (5p):

Compound **5p** was prepared following general procedure B. Purification by column chromatography (*n*-pentane:EtOAc = 20:1 → 4:1) yielded the title compound **5p** (124.5 mg, 0.23 mmol, 46%) as a colorless oil.

R$_f$ = 0.24 (*n*-pentane/EtOAc = 5:1, $KMnO_4$)

^{1}H NMR (300 MHz, Chloroform-*d*) δ 7.34 – 7.18 (m, 8H), 7.17 – 7.06 (m, 6H), 7.02 – 6.96 (m, 4H), 5.93 (dd, *J* = 8.1, 6.0 Hz, 1H), 3.15 (dd, *J* = 13.8, 8.1 Hz, 1H), 3.03 (dd, *J* = 13.8, 6.0 Hz, 1H), 2.32 – 2.26 (m, 8H), 2.08 – 2.03 (m, 2H), 1.54 (s, 3H).

^{13}C NMR (75 MHz, Chloroform-*d*) δ 172.8, 169.6, 148.9, 145.9, 140.1, 137.1, 129.6, 128.5, 128.3, 128.1, 126.7, 121.2, 76.7, 45.3, 43.1, 36.5, 30.5, 27.7, 21.3.

MS (EI): *m*/*z* (relative intensity) 353 (100), 311 (37), 297 (16), 269 (18), 255 (36), 213 (62), 181 (18), 165 (14), 121 (36), 103 (11), 91 (38), 43 (53).

HRMS (ESI-TOF, *m*/*z*): calcd. for $C_{35}H_{34}O_6$ [M+Na$^+$] 573.2247; found 573.2256.

IR (ATR, neat, cm^{-1}): 3032 (w), 2970 (w), 1754 (m), 1733 (m), 1603 (w), 1503 (m), 1454 (w), 1368 (m), 1294 (w), 1195 (s), 1167 (s), 1075 (w), 1046 (w), 1014 (m), 909 (s), 847 (m), 759 (m), 729 (s), 698 (s), 664 (w), 594 (w), 541 (m), 512 (w).

Scope of alkenes

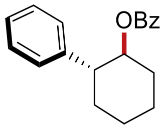

7b

Synthesis of 2-phenylcyclohexyl benzoate (7b):

Compound **7b** was prepared following general procedure A. Purification by column chromatography (*n*-pentane:EtOAc = 150:1 → 60:1) yielded the title compound **7b** (57.5 mg, 0.21 mmol, 41%) as a colorless solid.

R*f* = 0.47 (*n*-pentane/EtOAc = 20:1, $KMnO_4$)

m.p. = 44–46 °C

^{1}H NMR (300 MHz, Chloroform-*d*) δ 8.02 – 7.97 (m, 2H), 7.56 – 7.50 (m, 1H), 7.45 – 7.39 (m, 2H), 7.29 – 7.25 (m, 2H), 7.23 – 7.18 (m, 2H), 7.15 – 7.09 (m, 1H), 5.44 – 5.41 (m, 1H), 2.94 – 2.87 (m, 1H), 2.33 – 2.16 (m, 2H), 2.03 – 1.95 (m, 1H), 1.91 – 1.83 (m, 1H), 1.79 – 1.44 (m, 4H).

^{13}C NMR (75 MHz, Chloroform-*d*) δ 165.7, 143.3, 132.8, 131.0, 129.5, 128.4, 128.3, 127.8, 126.5, 74.0, 46.9, 31.0, 26.5, 26.2, 20.5.

MS (EI): *m*/*z* (relative intensity) 158 (40), 129 (9), 115 (15), 105 (100), 91 (36), 77 (72), 65 (6), 51 (17).

HRMS (ESI-TOF, *m*/*z*): calcd. for $C_{19}H_{20}O_2$ [M+Na$^+$] 303.1360; found 303.1364.

IR (ATR, neat, cm^{-1}): 3063 (w), 2934 (m), 2860 (w), 1706 (s), 1600 (w), 1496 (w), 1448 (m), 1368 (w), 1344 (w), 1312 (w), 1272 (s), 1173 (m), 1098 (s), 1069 (m), 1024 (m), 940 (m), 884 (m), 809 (w), 757 (m), 716 (s), 699 (s), 546 (m), 478 (w).

Synthesis of 4'-methoxy-2,3,4,5-tetrahydro-1,1'-biphenyl (6c):

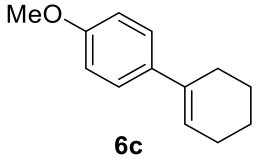

6c

Magnesium (267.8 mg, 11 mmol, 1.1 equiv.) was transferred to a 50 ml round-bottom-Schlenk-flask and 10 ml dry THF was added. A solution of 1-bromo-4-methoxybenzene (1.87 g, 10.0 mmol, 1.0 eq.) in 10 ml dry THF was added dropwise to the suspension at room temperature. After 2 h, the magnesium was mainly consumed, and cyclohexanone (992.0 mg, 10 mmol, 1 eq.) was added dropwise. The reaction was quenched with water (100 ml) and extracted with DCM (3x20 ml). The solvent was evaporated to a volume of 20 ml. *p*-Toluene sulfonic acid (190 mg, 1.00 mmol, 0.1 eq.) and anhydrous sodium sulfate was added. The solution was stirred over night at room temperature. Finally, the solvent was evaporated under reduced pressure and the residue was purified by column chromatography with pure *n*-pentane, yielding the title compound **6c** (1.16 g, 6.2 mmol, 62%) as a colorless solid.

R_f = 0.40 (*n*-pentane, UV)

^{1}H NMR (300 MHz, $CDCl_3$) δ 7.38 – 7.31 (m, 1H), 6.91 – 6.83 (m, 1H), 6.11 – 6.01 (m, 1H), 3.82 (s, 1H), 2.47 – 2.35 (m, 1H), 2.27 – 2.16 (m, 1H), 1.88 – 1.74 (m, 1H), 1.73 – 1.62 (m, 1H).

^{13}C NMR (75 MHz, $CDCl_3$) δ 158.5, 136.0, 135.4, 126.0, 123.2, 113.7, 55.4, 27.6, 26.0, 23.2, 22.3.

The analytical data is in accordance with those reported in the literature.[3]

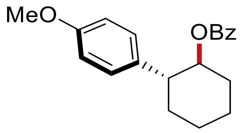

7c

Synthesis of (*trans*-1,2)-2-(4-methoxyphenyl)cyclohexyl benzoate (7c):

Compound **7c** was prepared following general procedure 1. Purification by column chromatography (*n*-pentane:EtOAc = 50:1 → 30:1) yielded the title compound **7c** (139.7 mg, 0.45 mmol, 90%) as a colorless solid.

R_f = 0.15 (*n*-pentane/EtOAc = 20:1, UV)

m.p. = 71–73 °C

^{1}H NMR (400 MHz, $CDCl_3$) δ 8.09 – 7.89 (m, 2H), 7.59 – 7.52 (m, 1H), 7.48 – 7.36 (m, 2H), 7.24 – 7.16 (m, 2H), 6.84 – 6.63 (m, 2H), 5.48 – 5.32 (m, 1H), 3.72 (s, 3H), 2.92 – 2.80 (m, 1H), 2.31 – 2.13 (m, 2H), 2.07 – 1.93 (m, 1H), 1.85 (dq, *J* = 12.8, 3.5 Hz, 1H), 1.79 – 1.42 (m, 4H).

^{13}**C NMR** (101 MHz, $CDCl_3$) δ 165.7, 158.1, 135.5, 132.8, 131.1, 129.6, 128.7, 128.4, 113.7, 74.2, 55.2, 46.1, 30.9, 26.8, 26.2, 20.5.

MS (EI): *m/z* (relative intensity) 310 (4), 205 (2), 188 (100), 160 (12), 121 (72), 105 (34), 77 (22).

HRMS (ESI-TOF, *m/z*): calcd. for $C_{20}H_{22}O_3$ [M+Na$^+$] 333.1461; found 333.1464.

IR (ATR, neat, cm^{-1}): 2938(w), 2925(w), 1707(s), 1511(m), 1452(w), 1438(w), 1315(w), 1270(s), 1259(s), 1242(s), 1177(m), 1111(m), 1092(w), 1070(m), 1051(w), 1035(m), 1027(m), 1000(w), 944(w), 930(w), 885(w), 828(m), 814(m), 790(w), 713(s), 687(w), 593(w), 543(m), 529(w), 457(w).

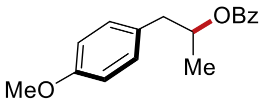

7d

Synthesis of 1-(4-methoxyphenyl)propan-2-yl benzoate (7d):

Compound **7d** was prepared following general procedure A. Purification by column chromatography (*n*-pentane:EtOAc = 50:1) yielded the title compound **7d** (98.7 mg, 0.37 mmol, 73%) as a colorless oil.

R$_f$ = 0.34 (*n*-pentane/EtOAc = 20:1, UV)

^{1}**H NMR** (400 MHz, Chloroform-*d*) δ 8.04 – 8.01 (m, 2H), 7.56 – 7.53 (m, 1H), 7.46 – 7.41 (m, 2H), 7.19 – 7.16 (m, 2H), 6.85 – 6.82 (m, 2H), 5.33 (sext, *J* = 6.4 Hz, 1H), 3.78 (s, 3H), 3.02 (dd, *J* = 13.8, 6.4 Hz, 1H), 2.85 (dd, *J* = 13.8, 6.4 Hz, 1H), 1.34 (d, *J* = 6.2 Hz, 3H).

^{13}**C NMR** (100 MHz, Chloroform-*d*) δ 166.2, 158.4, 132.9, 130.9, 130.6, 129.7, 129.6, 128.4, 113.9, 72.4, 55.3, 41.5, 19.5.

MS (EI): *m/z* (relative intensity) 148 (100), 133 (7), 121 (73), 105 (82), 91 (18), 77 (95), 65 (6), 51 (26).

HRMS (ESI-TOF, *m/z*): calcd. for $C_{17}H_{18}O_3$ [M+Na$^+$] 293.1148; found 293.1153.

IR (ATR, neat, cm^{-1}): 2933(w), 1711(s), 1611(w), 1584(w), 1511(s), 1451(m), 1380(w), 1354(w), 1314(w), 1270(s), 1246(s), 1218(m), 1176(m), 1108(s), 1069(m), 1053(m), 1026(s), 935(w), 918(w), 831(w), 808(m), 755(w), 710(s), 687(w), 674(w), 571(w).

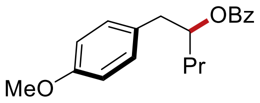

7e

Synthesis of 1-(4-methoxyphenyl)pentan-2-yl benzoate (7e):

Compound **7e** was prepared following general procedure A. Purification by column chromatography (*n*-pentane:EtOAc = 50:1) yielded the title compound **7e** (124 mg, 0.42 mmol, 83%) as a colorless oil.

R$_f$ = 0.24 (*n*-pentane/EtOAc = 20:1, UV)

^{1}H NMR (400 MHz, $CDCl_3$) δ 8.17 – 7.92 (m, 2H), 7.67 – 7.51 (m, 1H), 7.52 – 7.38 (m, 2H), 7.23 – 7.11 (m, 2H), 6.89 – 6.76 (m, 2H), 5.30 (dtd, *J* = 7.7, 6.3, 4.8 Hz, 1H), 3.78 (s, 3H), 2.98 (dd, *J* = 13.9, 6.3 Hz, 1H), 2.88 (dd, *J* = 13.9, 6.3 Hz, 1H), 1.73 – 1.57 (m, 2H), 1.52 – 1.33 (m, 2H), 0.91 (t, *J* = 7.3 Hz, 3H).

^{13}C NMR (101 MHz, $CDCl_3$) δ 166.3, 158.3, 132.9, 130.8, 130.6, 129.7, 129.7, 128.4, 113.9, 77.4, 75.6, 55.3, 39.8, 35.6, 18.8, 14.1.

MS (EI): *m*/*z* (relative intensity) 176 (96), 147 (100), 121 (47), 105 (69), 91 (8), 77 (37), 51 (6).

HRMS (ESI-TOF, *m*/*z*): calcd. for $C_{19}H_{22}O_3$ [M+Na$^+$] 321.1461; found 321.1469.

IR (ATR, neat, cm^{-1}): 2957(w), 1711(s), 1612(w), 1511(m), 1464(w), 1450(w), 1314(w), 1268(s), 1244(s), 1175(m), 1108(s), 1069(m), 1035(m), 1026(m), 985(w), 947(w), 833(w), 807(w), 708(s), 687(w), 674(w), 517(w).

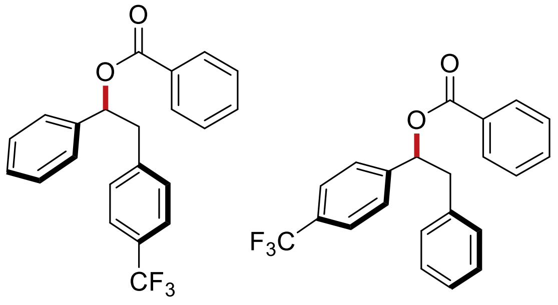

7f **7f'**

Synthesis of 2-phenyl-1-(4-(trifluoromethyl)phenyl)ethyl benzoate (7f) and 1-phenyl-2-(4-(trifluoromethyl)phenyl)ethyl benzoate (7f'):

Compounds **7f** and **7f'** were prepared following general procedure A. Purification by column chromatography (*n*-pentane:EtOAc = 50:1) yielded the title compound **7f** and **7f'** (135 mg, 0.37 mmol, 73%) as a colorless solid as a 69:31 mixture of regioisomers.

7f:

R$_f$ = 0.32 (*n*-pentane/EtOAc = 20:1, UV)

^{1}H NMR (300 MHz, $CDCl_3$) δ 8.1 – 8.0 (m, 2H), 7.6 – 7.5 (m, 1H), 7.5 – 7.4 (m, 4H), 7.4 – 7.3 (m, 5H), 7.3 – 7.2 (m, 1H), 7.2 – 7.2 (m, 1H), 6.2 (dd, *J* = 7.5, 6.0 Hz, 1H), 3.4 (dd, *J* = 13.8, 7.4 Hz, 1H), 3.3 (dd, *J* = 13.8, 6.0 Hz, 1H), 1.3 (s, 2H).

^{13}C NMR (75 MHz, $CDCl_3$) δ 165.7, 141.1, 139.6, 133.3, 130.3 (q, *J* = 33.2 Hz), 130.2, 130.1, 129.7, 128.7, 128.6, 128.4, 126.6, 125.3 (q, *J* = 3.8 Hz), 124.3 (q, *J* = 271.7 Hz), 76.8, 43.0.

^{13}C NMR (126 MHz, $CDCl_3$) δ 165.6, 141.0, 139.5, 133.1, 130.1, 129.9, 129.6, 129.0 (d, J = 32.4 Hz), 128.5, 128.5, 128.2, 126.5, 125.2 (q, J = 3.8 Hz), 124.2 (q, J = 271.7 Hz), 76.8, 42.9.

^{19}F NMR (282 MHz, $CDCl_3$) δ −62.43.

MS (EI): m/z (relative intensity) 248 (4), 229 (5), 211 (22), 178 (7), 159 (7), 151 (3), 105 (100), 77 (28), 51 (6).

HRMS (ESI-TOF, m/z): calcd. for $C_{22}H_{17}O_2F_3$ [M+Na$^+$] 393.1078; found 393.1083.

IR (ATR, neat, cm^{-1}): 2923(w), 1705(w), 1450(w), 1340(w), 1318(s), 1270(m), 1159(s), 1128(s), 1106(s), 1065(s), 1048(m), 1026(m), 1018(m), 990(m), 955(w), 935(w), 923(w), 888(w), 868(w), 858(w), 822(m), 800(w), 769(m), 753(w), 733(w), 706(s), 688(s), 671(w), 651(m), 617(w), 596(m), 537(s), 462(w), 426(w), 415(w).

7f':

R$_f$ = 0.32 (n-pentane/EtOAc = 20:1, UV)

^{1}H NMR (300 MHz, $CDCl_3$) δ 8.2 – 8.0 (m, 2H), 7.7 – 7.5 (m, 3H), 7.5 – 7.4 (m, 4H), 7.3 – 7.2 (m, 3H), 7.2 – 7.1 (m, 2H), 6.2 (dd, J = 7.4, 6.2 Hz, 1H), 3.4 (dd, J = 13.8, 7.4 Hz, 1H), 3.2 (dd, J = 13.8, 6.2 Hz, 1H).

^{13}C NMR (126 MHz, $CDCl_3$) δ 165.7, 144.2, 136.3, 133.4, 130.3 (q, J = 32.5 Hz), 130.1, 129.8, 129.7, 128.6, 128.6, 127.0, 126.9, 125.6 (q, J = 3.8 Hz), 124.2 (d, J = 272.3 Hz), 76.7, 43.2.

^{19}F NMR (282 MHz, $CDCl_3$) δ −62.58.

MS (EI): m/z (relative intensity) 248 (43), 178 (7), 151 (3), 105 (100), 91 (8), 77 (27), 51 (5).

HRMS (ESI-TOF, m/z): calcd. for $C_{22}H_{17}O_2F_3$ [M+Na$^+$] 393.1078; found 393.1080.

IR (ATR, neat, cm^{-1}): 2923(w), 1705(s), 1451(w), 1318(s), 1272(s), 1259(m), 1192(w), 1160(s), 1132(s), 1106(s), 1065(s), 1046(m), 1018(m), 997(m), 933(w), 883(w), 862(w), 852(w), 834(s), 808(w), 763(w), 747(m), 740(m), 709(s), 702(s), 685(s), 660(m), 607(m), 568(w), 531(s), 491(w), 453(w), 421(w).

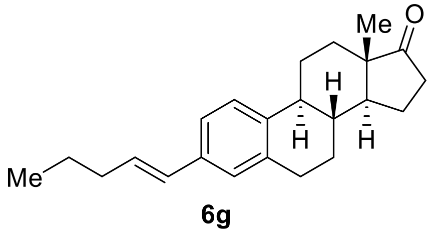

6g

Synthesis of 3-pent-2'-enyl-estra-1,3,5(10)-trien-17-on (6g):

Compound **6g** was prepared following modified procedure.[4] Estrone-triflate (805 mg, 2.00 mmol, 1.0 equiv.), 1-pentenylboronic acid (450 mg, 4.00 mmol, 2.0 equiv.), palladium(II) acetate (43 mg, 10 mol%), triphenylphosphine (65 mg, 13 mol%) and cesium carbonate (1.95 g, 6.00 mmol, 3.0 equiv.) was added to a microwave vial. Afterwards the vial was sealed, evacuated, and filled with argon. Dry tetrahydrofuran (3.60 mL) and distilled degassed water (0.4 mL) were added and the mixture was stirred at 85 °C for 16 h. The reaction was quenched by the addition of water and exposure to air. The crude product was extracted with dichloromethane, dried over Na_2SO_4, concentrated under reduced pressure and purified by column chromatography. The title compound **6g** (582 mg, 1.80 mmol, 90%). was obtained as a colorless crystalline solid

R$_f$ = 0.59 (*n*-pentane/EtOAc = 10:1, UV)

^{1}H NMR (300 MHz, $CDCl_3$) δ 7.25 – 7.19 (m, 1H), 7.15 (dd, *J* = 8.1, 1.9 Hz, 1H), 7.08 (d, *J* = 1.8 Hz, 1H), 6.33 (dt, *J* = 15.8, 1.3 Hz, 1H), 6.18 (dt, *J* = 15.8, 6.7 Hz, 1H), 2.91 (dd, *J* = 8.9, 4.2 Hz, 2H), 2.59 – 2.23 (m, 3H), 2.23 – 1.88 (m, 6H), 1.71 – 1.36 (m, 9H), 0.95 (t, *J* = 7.4 Hz, 3H), 0.91 (s, 3H).

^{13}C NMR (75 MHz, $CDCl_3$) δ 221.1, 138.5, 136.6, 135.7, 130.5, 129.7, 126.6, 125.6, 123.5, 50.6, 48.1, 44.5, 38.3, 36.0, 35.3, 31.7, 29.5, 26.7, 25.9, 22.7, 21.7, 14.0, 13.8.

HRMS (ESI-TOF, *m/z*): calcd. for $C_{23}H_{30}O$ [M+H$^+$] 323.2375; found 323.2378.

IR (ATR, neat, cm^{-1}): 2953 (w), 2928 (m), 2871 (w), 1730 (s), 1494 (w), 1463 (w), 1450 (w), 1438 (w), 1402 (w), 1370 (w), 1338 (w), 1258 (w), 1223 (w), 1187 (w), 1166 (w), 1113 (w), 1082 (m), 1048 (m), 1006 (w), 966 (s), 948 (w), 915 (w), 887 (w), 872 (w), 836 (w), 825 (m), 781 (m), 755 (w), 736 (w), 707 (w), 629 (w), 607 (w), 581 (w), 562 (w), 486 (w), 439 (m).

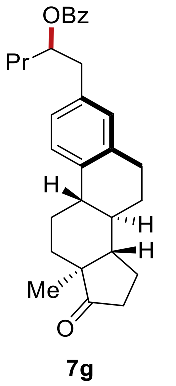

7g

Synthesis of estra-1,3,5(10)-trien-17-on-3-pentan-2'-yl benzoate (**7g**)**:**

Compound **7g** was prepared following general procedure A. Purification by column chromatography (*n*-pentane:EtOAc = 10:1) yielded the title compound **7g** (142 mg, 0.32 mmol, 64 %) as a colorless oil.

R$_f$ = 0.32 (*n*-pentane/EtOAc) = 10:1, UV)

m.p. = 48 – 52 °C

^{1}H NMR (300 MHz, $CDCl_3$) δ 8.02 – 7.88 (m, 2H), 7.54 – 7.43 (m, 1H), 7.39 – 7.29 (m, 2H), 7.12 (dt, *J* = 8.0, 1.3 Hz, 1H), 7.00 – 6.84 (m, 2H), 5.30 – 5.16 (m, 1H), 2.91 (dd, *J* = 13.7, 6.1 Hz, 1H), 2.83 – 2.68 (m, 3H), 2.43 (dd, *J* = 18.3, 8.3 Hz, 1H), 2.32 (dt, *J* = 11.8, 3.0 Hz, 1H), 2.27 – 2.14 (m, 1H), 2.13 – 1.82 (m, 4H), 1.71 – 1.13 (m, 10H), 0.88 – 0.80 (m, 6H).

^{13}C NMR (75 MHz, $CDCl_3$) δ 221.0, 166.2, 137.8, 136.4, 135.0, 132.8, 130.8, 130.2, 130.2, 129.6, 128.3, 127.0, 125.3, 75.5, 50.5, 48.0, 44.3, 40.1, 38.2, 35.9, 35.6, 31.6, 29.3, 26.5, 25.7, 21.6, 18.7, 14.0, 13.9.

MS (EI): *m*/*z* (relative intensity) 322 (100), 293 (4), 169 (3), 155 (4), 143 (4), 141 (4), 129 (14), 105 (49), 77 (10).

HRMS (ESI-TOF, *m*/*z*): calcd. for $C_{30}H_{36}O_3$ [M+Na$^+$] 467.2562; found 467.2556.

IR (ATR, neat, cm^{-1}): 2956(w), 2928(w), 2870(w), 1737(m), 1711(s), 1451(w), 1313(w), 1268(s), 1175(w), 1110(m), 1083(w), 1068(m), 1054(w), 1025(w), 1006(w), 946(w), 820(w), 710(s), 688(w), 675(w), 580(w), 442(w).

Deprotection of 1,2-diphenylethylbenzoate (7a)

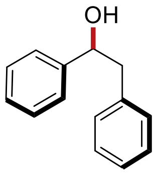

8

Synthesis of (S)-1,2-diphenylethan-1-ol (8):

A 50 mL flask equipped with a magnetic stir bar was filled with a solution of NaOH (20 mg, 0.5 mmol, 0.5 equiv.) in methanol (21 ml) and 1,2-diphenylethylbenzoate (**7a**) (302 mg, 1.0 mmol). The reaction mixture was stirred for 24 h at room temperature. Subsequently, the reaction mixture was concentrated under reduced pressure, the residue obtained was added with water and extracted with dichloromethane (4x15 ml). The combined organic layers were washed with brine, dried over anhydrous Na_2SO_4, filtered and evaporated to leave the title compound **8** (183 mg, 0.923 mmol, 92%) as a colorless solid.

R_f = 0,33 (*n*-pentane/EtOAc = 3:1, UV).

m.p. = 63 – 64 °C

^{1}H NMR (400 MHz, $CDCl_3$) δ = 7.4 – 7.2 (m, 10H), 4.9 (dd, *J* = 8.3, 5.0 Hz, 1H), 3.1 – 3.0 (m, 2H), 2.0 (brs, 1H).

^{13}C NMR (101 MHz, $CDCl_3$) δ = 143.9, 138.2, 129.6, 128.6, 128.5, 127.7, 126.7, 126.0, 75.5, 46.2.

MS (EI, 70 eV): *m/z* (relative intensity) 198 (1), 178 (5), 165 (3), 152 (2), 139 (1), 128 (1), 115 (1), 107 (86), 92 (100), 79 (65), 77 (43), 65 (12), 51 (8), 39 (4).

IR (ATR, neat, cm^{-1}): 3298(w), 3026(w), 2921(w), 2860(w), 1494(w), 1453(w), 1445(w), 1407(w), 1316(w), 1272(w), 1208(w), 1197(w), 1150(w), 1071(w), 1039(m), 1026(m), 1017(m), 952(w), 917(w), 778(w), 760(m), 741(m), 695(s), 616(m), 590(w), 551(s), 498(m), 435(w).

The analytical data is in accordance with those reported in the literature.[5]

Crystallographic data

Data were collected on a Bruker Kappa APEX II Duo diffractometer. The structures were solved by direct methods (SHELXS-97: Sheldrick, G. M. *Acta Cryst.* **2008**, *A64*, 112.) and refined by full-matrix least-squares procedures on F^2 (SHELXL-2018: Sheldrick, G. M. *Acta Cryst.* **2015**, *C71*, 3.). XP (Bruker AXS) was used for graphical representations. CCDC 2108421 (**3a**) and 2169089 (**7g**) contain the supplementary crystallographic data for this paper. These data are provided free of charge by the joint Cambridge Crystallographic Data Centre and Fachinformationszentrum Karlsruhe Access Structures service www.ccdc.cam.ac.uk/structures.

X-ray crystal structure analysis of 3a (CCDC 2108421):

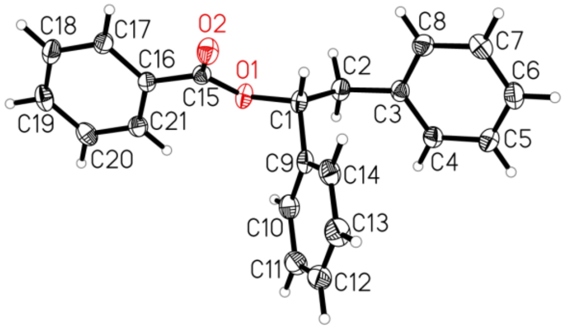

Figure S 1. ORTEP representation of **3a**. Displacement ellipsoids correspond to 50% probability.

Crystal data of **3a**: $C_{21}H_{18}O_2$, M = 302.35, monoclinic, space group $P2_1/c$, a = 11.7146(7), b = 5.7605(3), c = 23.7333(14) Å, β = 100.863(2)°, V = 1572.87(16) Å^3, T = 150(2) K, Z = 4, 39860 reflections measured, 4256 independent reflections (R_{int} = 0.0266), final R values ($I > 2\sigma(I)$): R_1 = 0.0403, wR_2 = 0.1050, final R values (all data): R_1 = 0.0462, wR_2 = 0.1105, 208 parameters, largest diff. peak/hole: 0.29/ -0.23 eÅ^{-3}.

X-ray crystal structure analysis of 7g (CCDC 2169089):

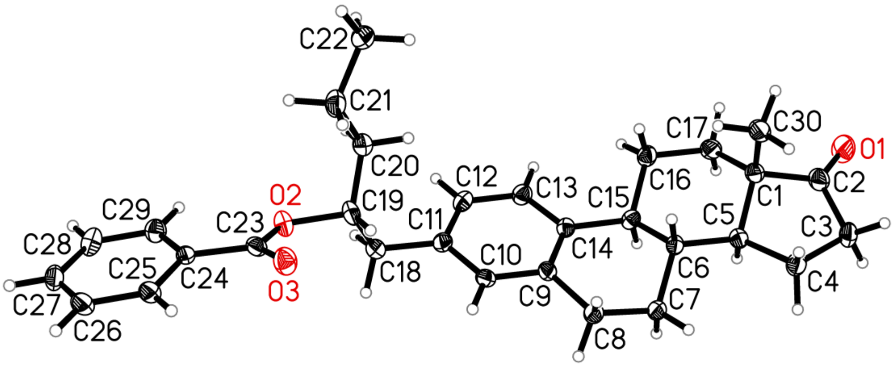

Figure S 2. ORTEP representation of **7g**. Displacement ellipsoids correspond to 30% probability.

Crystal data of **7g**: $C_{30}H_{36}O_3$, M = 444.59, orthorhombic, space group $P2_12_12_1$, a = 9.9361(6), b = 10.6474(6), c = 22.9826(13) Å, V = 2431.4(2) Å^3, T = 150(2) K, Z = 4, 30169 reflections measured, 4291 independent reflections (R_{int} = 0.0353), final R values ($I > 2\sigma(I)$): R_1 = 0.0537, wR_2 = 0.1371, final R values (all data): R_1 = 0.0542, wR_2 = 0.1374, 288 parameters, largest diff. peak/hole: 0.31/ -0.16 eÅ^{-3}.

Stern-Volmer Experiments

Stern-Volmer quenching experiments were run with freshly prepared solutions of **BuPPT** (0.02 mM in dry MeCN) at room temperature under an inert atmosphere. The solutions were degassed by the "freeze-pump-thaw" technique and flushed with argon directly before the measurement. The solutions were irradiated at 355 nm and luminescence was measured at 386 nm for (*E*)-stilbene (**1a**) or 390 nm for tetrabutylammonium benzoate (**2a·TBA**). The data shows that (*E*)-stilbene (**1a**: K_{SV} = 64.7 M^{-1}) is a better quencher for the excited state of **BuPPT** than tetrabutylammonium benzoate (**2a·TBA**: K_{SV} = 22.7 M^{-1}). For all tabular and graphical data, see Tables S1 and Figures S5 below.

Table S 5: Fluorescence quenching data of a solution of **BuPPT** by a solution of (*E*)-stilbene (**1a**).

I_x [a.u.]	$I_0/I_x - 1$ [a.u.]	Q [mM]
I_0 = 586.5649	0	0
I_1 = 512.6869	0.1441	2.8564
I_2 = 451.3364	0.2996	5.4530
I_3 = 402.9436	0.4557	7.8239
I_4 = 366.0566	0.6024	9.9972
I_5 = 333.4634	0.7590	11.9967
I_6 = 307.3798	0.9083	13.8423
I_7 = 285.4988	1.0545	15.5512
I_8 = 268.5372	1.1843	17.1381

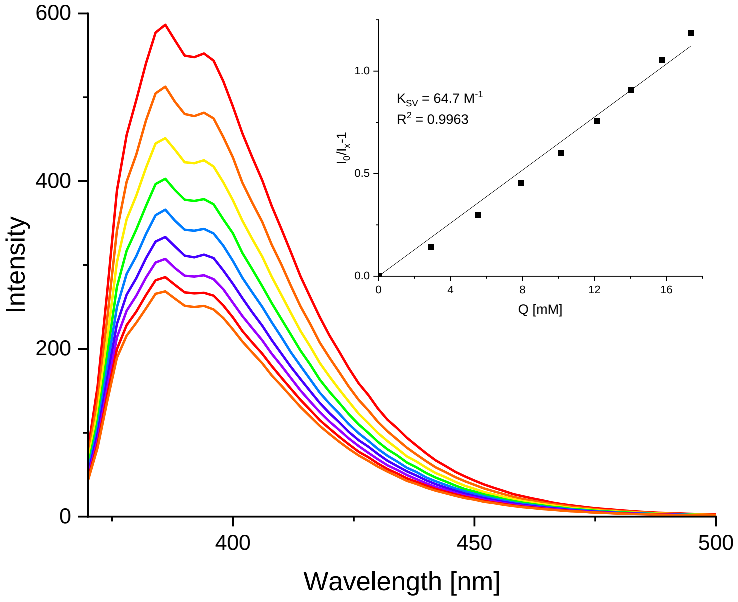

Figure S 3: Quenching of luminescence and Stern-Volmer plot (inset) of **BuPPT** with varying concentrations of (*E*)-stilbene (**1a**).

Table S 6: Fluorescence quenching data of a solution of **BuPPT** with a solution of tetrabutylammonium benzoate (**2a·TBA**).

I_x [a.u.]	$I_0/I_x - 1$ [a.u.]	Q [mM]
I_0 = 774.7062	0	0
I_1 = 736.4794	0.0519	2.9036
I_2 = 701.1320	0.1049	5.5432
I_3 = 667.1906	0.1611	7.9533
I_4 = 636.1788	0.2177	10.1625
I_5 = 601.4441	0.2881	12.1951
I_6 = 577.8432	0.3407	14.0712

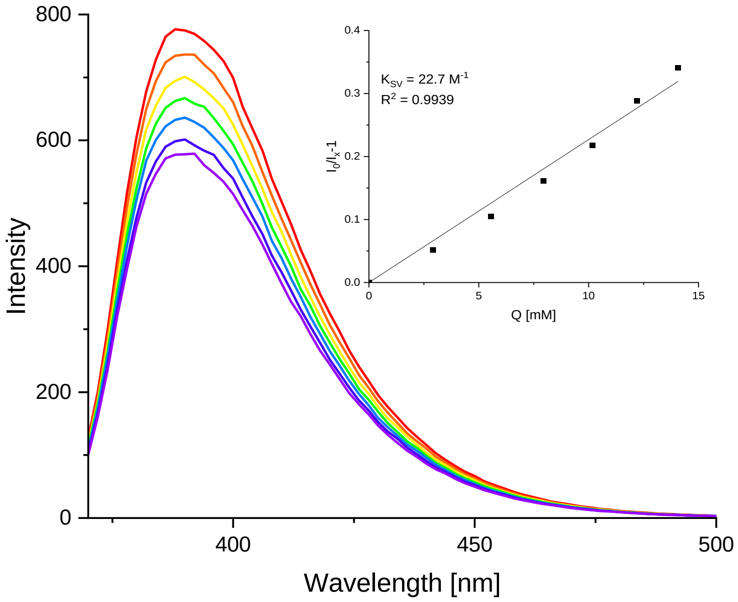

Figure S 4: Quenching of luminescence and Stern-Volmer plot (inset) of **BuPPT** with varying concentrations of tetrabutylammonium benzoate (**2a·TBA**).

Competitive Stern-Volmer Experiments

Stern-Volmer quenching experiments were run with freshly prepared solutions of **BuPPT** (0.02 mM in dry MeCN) at room temperature under an inert atmosphere. The solutions were degassed by “freeze-pump-thaw” technique and then flushed with argon directly before measurement. The solutions were irradiated at 355 nm and luminescence was measured at 388 nm. In this competitive experiment, the fluorescence quenching of a quencher up to a concentration of 10 mM was measured. Subsequently, the influence of the second quencher was examined. The data shows that in a competitive Stern-Volmer experiment *E*-stilbene (**1a**: K_{SV} = 58.2 M^{-1}) is a significantly better quencher for the excited state of **BuPPT** than tetrabutylammonium benzoate (**2a·TBA**: K_{SV} = 19.2 M^{-1}). For all tabular and graphical data, see Tables S7 and Figures S5 below.

Table S 7: Fluorescence quenching data from a solution of **BuPPT** first with (*E*)-stilbene (**1a**; Q_1) and then tetrabutylammonium benzoate (**2a·TBA**; Q_2).

I_x [a.u.]	Q_1: $I_0/I_x - 1$ [a.u.]	Q_2: $I_0/I_x - 1$ [a.u.]	Q_1 [mM]	Q_2 [mM]
I_0 = 793.7940	0	-	0	0
I_1 = 686.4799	0.1563	-	2.8902	0
I_2 = 605.6771	0.3106	-	5.5176	0
I_3 = 544.6657	0.4574	-	7.9165	0
I_4 = 496.2029	0.5997	0	10.1156	0
I_5 = 482.5254	0.6451	0.0283	9.7110	2.4390
I_6 = 469.7441	0.6898	0.0563	9.3374	4.6904
I_7 = 459.3331	0.7281	0.0803	8.9916	6.7750
I_8 = 446.5582	0.7776	0.1112	8.6705	8.7108
I_9 = 434.1786	0.8283	0.1429	8.3715	10.5130
I_{10} = 427.2868	0.8578	0.1613	8.0925	12.1951

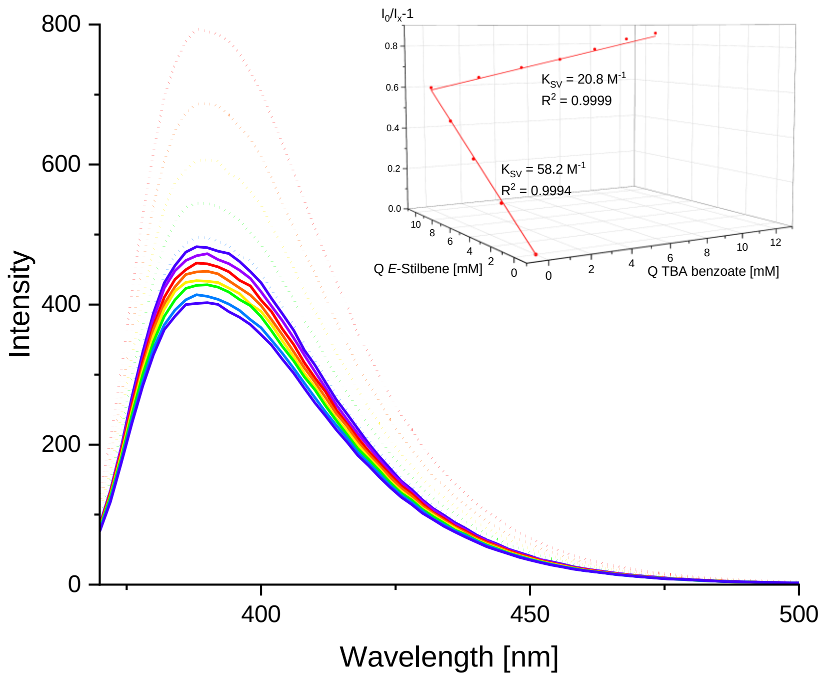

Figure S 5: Quenching of luminescence and Stern-Volmer plot (inset) of **BuPPT** variation of the concentration of (*E*)-stilbene (**1a**) (dotted lines) and subsequent variation of the concentration of tetrabutylammonium benzoate (**2a·TBA**) (solid lines).

Table S 8: Fluorescence quenching data from a solution of **BuPPT** first with tetrabutylammonium benzoate (**2a**·**TBA**; Q_1) and then (*E*)-stilbene (**1a**; Q_2).

I_x [a.u.]	Q_1: $I_0/I_x - 1$ [a.u.]	Q_2: $I_0/I_x - 1$ [a.u.]	Q_1 [mM]	Q_2 [mM]
I_0 = 778.5728	0	-	0	0
I_1 = 738.2601	0.0546	-	2.9036	0
I_2 = 706.2041	0.1025	-	5.5432	0
I_3 = 673.5037	0.1560	-	7.9533	0
I_4 = 651.1787	0.1956	0	10.1625	0
I_5 = 580.5770	0.3410	0.1216	9.7560	2.4277
I_6 = 531.5888	0.4646	0.2250	9.3808	4.6687
I_7 = 479.4892	0.6238	0.3581	9.0334	6.7437
I_8 = 444.3243	0.7523	0.4655	8.7108	8.6705
I_9 = 410.8751	0.8949	0.5849	8.4104	10.4644
I_{10} = 381.9334	1.0385	0.7050	8.1300	12.1387

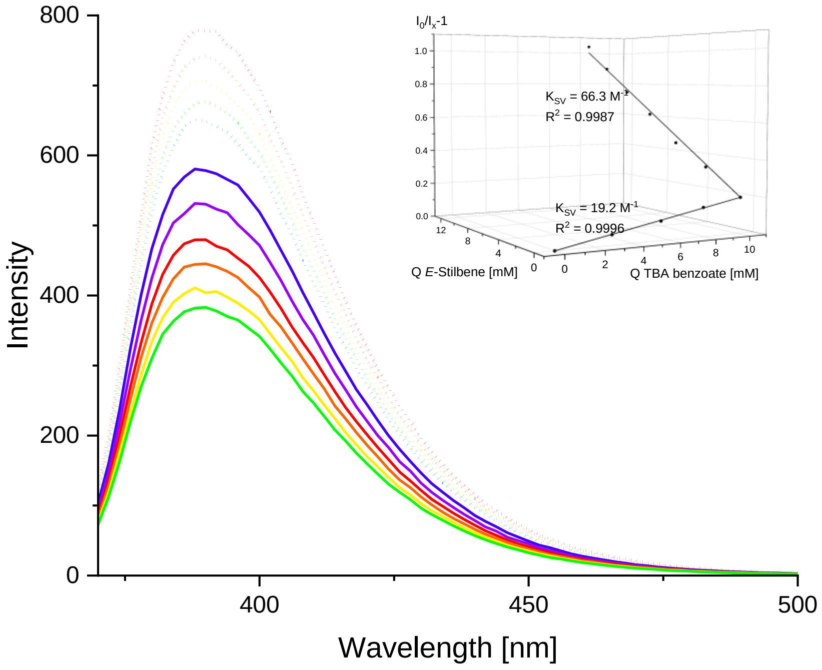

Figure S 6: Quenching of luminescence and Stern-Volmer plot (inset) of **BuPPT** first varying the concentration of tetrabutylammonium benzoate (**2a**·**TBA**) (dotted lines) and subsequent addition of (*E*)-stilbene (**1a**) (solid lines).

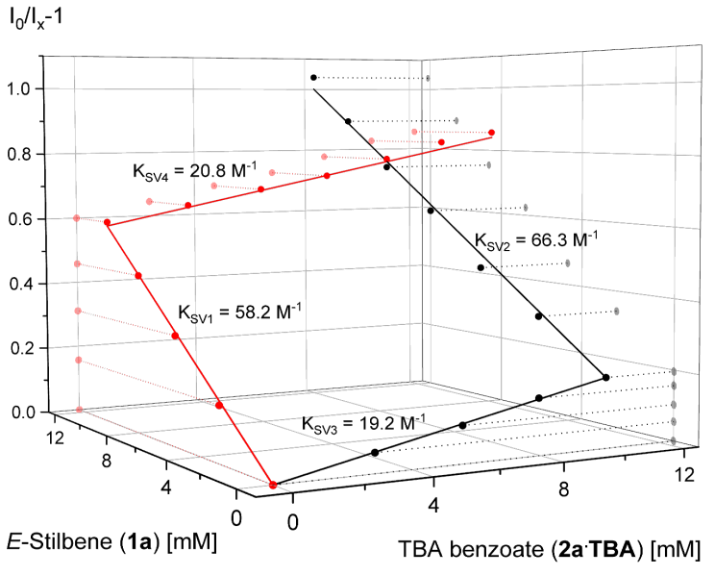

Figure S 7: Stern-Volmer plot of **BuPPT** first varying the concentration of tetrabutylammonium benzoate (**2a·TBA**) and subsequent addition of (*E*)-stilbene (**1a**) (black) and Stern-Volmer plot of **BuPPT** with variation of the concentration of (*E*)-stilbene (**1a**) and subsequent addition of tetrabutylammonium benzoate (**2a·TBA**) (red), respectively.

EPR Spectroscopy

EPR spectrum was recorded on an X-band Bruker EMX CW-micro EPR spectrometer equipped with an ER4119HS high-sensitivity resonator using a microwave power of Ca 6.9 mW, modulation frequency of 100 kHz and modulation amplitude 1 G. The experimental spectrum was simulated with EasySpin program.[6]

The EPR spectrum was recorded at 20 °C after irradiating a home-made EPR quartz flat-cell (ID 0.5 mm) filled with benzoic acid (**2a**) solution (0.1 M in MeCN, also containing 5.0 mol% BuPPT and 0.33 equiv. K_3PO_4) under Ar at 396 nm.

The EPR spectrum exhibited a characteristic multiple-lines signal at g = 2.005 with coupling constant A_{N1} = 7.84 G and A_{N2} = 3.72 and G A_H = 7.45 G (Figure S8). The spectrum was simulated considering a hyperfine splitting from two nonequivalent nitrogen and one hydrogen atom corresponding to the formation of a **PPTH•** radical anion.[7]

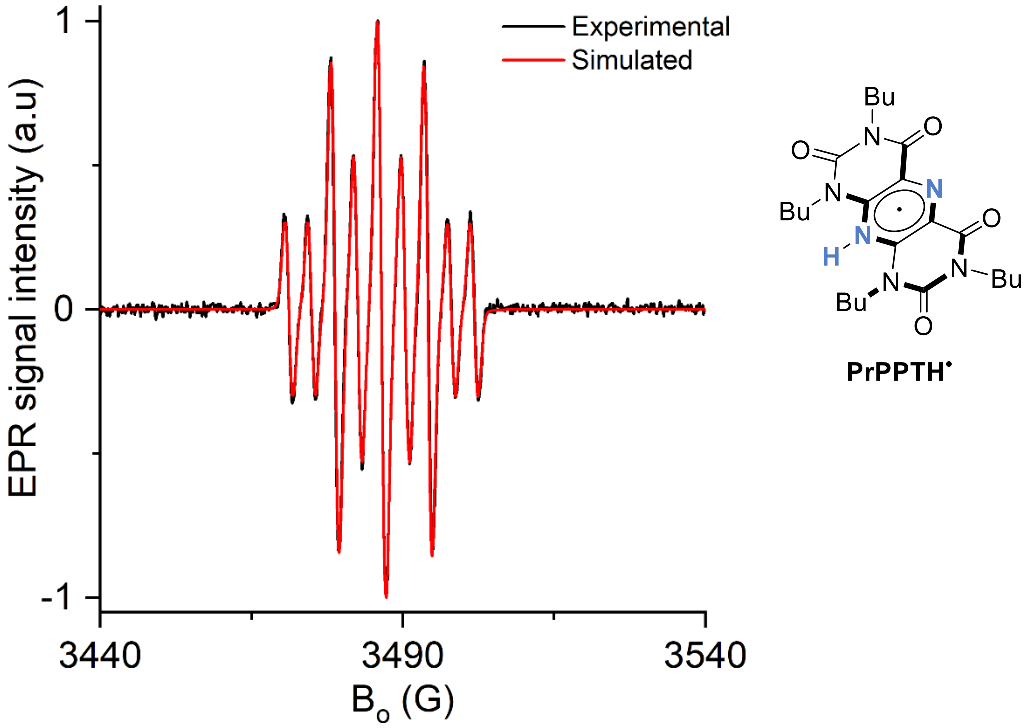

Figure S8. EPR spectrum of benzoic acid (**2a**, 0.1 M in MeCN), **BuPPT** (5.0 mol%) and K_3PO_4 (0.33 equiv.), irradiated at 396 nm at 20 °C for 60 min (black experimental and red simulated).

Reaction Monitoring

The reaction monitoring was performed under standard reaction conditions on a 0.5 mmol-scale with *trans*-stilbene (**1a**) and benzoic acid (**2a**) as model substrates. Following the general procedure A, seven samples were prepared and irradiated for 1, 2, 4, 6, 8, 16 and 24 h, respectively. After quenching of the reactions with air, the samples were analyzed by calibrated GC using biphenyl as internal standard. Initial rates were determined at conversions below 20% within the first 6 hours.

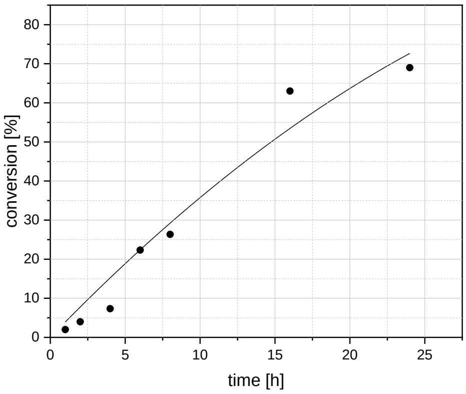

time [h]	conversion [%] run 1	run 2	run 3	mean
1	2	2	2	2
2	4	4	4	4
4	8	7	7	7.34
6	21	23	23	22.34
8	26	26	27	26.34
16	65	63	61	63
24	67	69	71	69

Figure S9: Conversion/time diagram of hydroacetoxylation product **3a**.

Free-energy relationship (Hammett-plot analysis)

The Hammett-plot was developed by carrying out competition experiments between benzoic acid derivatives (Scheme S 1) at low conversions (20 %) according to general procedure C. The selected benzoic acids bear electron-donating and electron-withdrawing substituents in para- and meta position. The relative rate of the reaction was determined in intramolecular competition experiments between benzoic acid and the substituted benzoic acid derivatives at low conversion.

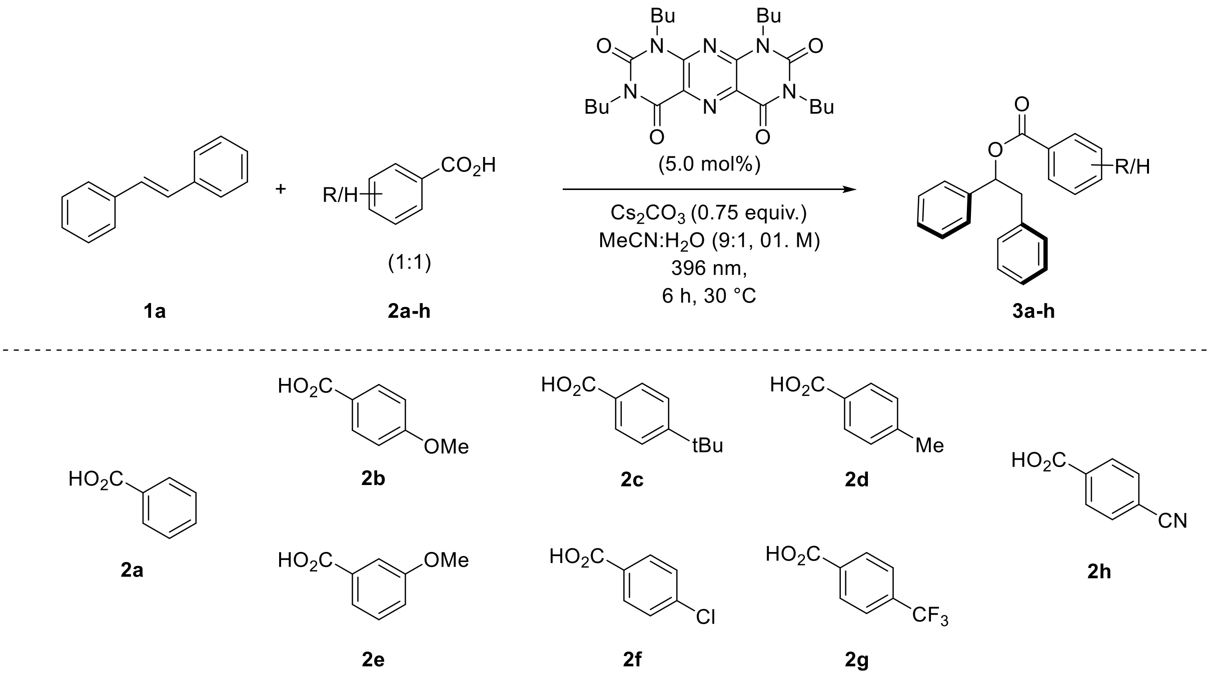

Scheme S 1. Benzoic acid derivatives (**2b-h**) used for competition experiments.

General procedure C. In a typical experiment, a 10 mL Schlenk flask equipped with a magnetic stir bar was filled with *trans*-stilbene (0.5 mmol, 1.0 equiv.), benzoic acid (0.375 mmol, 0.75 equiv.), the competing benzoic acid derivative (0.75 mmol, 1.5 equiv.), BuPPT (5.0 mol%), Cs_2CO_3 (0.37 mmol, 0.75 equiv.), and sealed with a rubber septum. The flask was then evacuated and purged with argon for 3 times. Acetonitrile (4.5 mL) and water (0.5 mL) were subsequently added under inert atmosphere. The reaction mixture was stirred for 6 h under irradiation at 396 nm. Subsequently, aqueous K_2CO_3 solution (10wt%, 15 mL) and brine (15 mL) were added, and the reaction mixture was extracted with dichloromethane (4 x 15 mL). The combined organic layers were washed with brine (15 mL) and dried over anhydrous Na_2SO_4, filtered and concentrated under reduced pressure. The samples were then analyzed by calibrated GC using biphenyl as internal standard.

Table S 9: Data for the Hammett-plot analysis.

entry	experiment	conversion [%][a]	peak area (R)	peak area (H)	log (k_x/k_H)	σ	σ^+	σ^-
1	*p*-OMe:H	42	256473	343319	-0,127	-0,27	-0,78	-0,26
2	*t*-Bu:H	14	83961	84393	-0,002	-0,2	-0,26	-0,13
3	Me:H	25	142844	150525	-0,023	-0,17	-0,31	-0,17
4	*m*-OMe:H	44	175444	209341	-0,077	0,12	-	-
5	Cl:H	12	56518	73993	-0,117	0,23	0,11	0,19
6	CF_3:H	21	97706	107510	-0,042	0,54	0,61	0,65
7	CN:H	34	68820	167940	-0,387	0,66	0,66	1

[a] Conversions were determined by calibrated GC using biphenyl as internal standard as sum of both products.

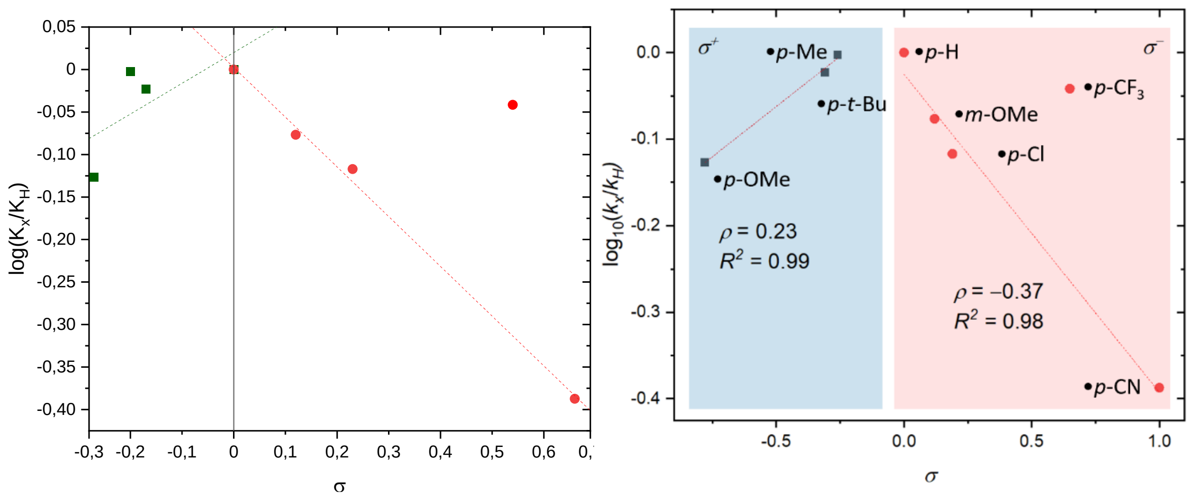

Figure S 9: Correlation of σ-values (left) and σ^+/σ^--values (right) with the respective product ratios of the competition experiments.

Mechanistic experiments

Reactivity of potassium benzoate

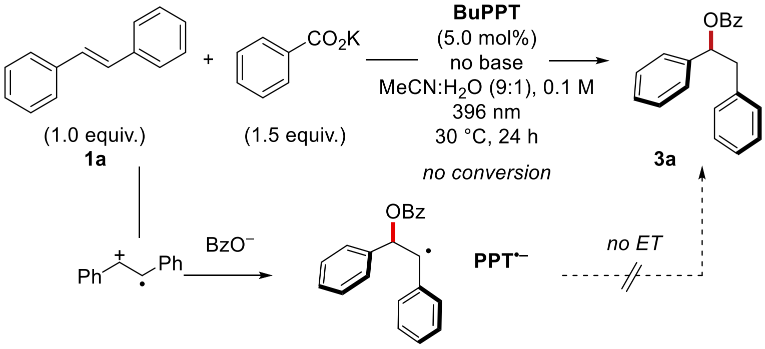

Scheme S 2. Reactivity of potassium benzoate.

In a preheated, dry, 10 mL Schlenk flask equipped with a magnetic stir bar, *trans*-stilbene (**1a**) (0.5 mmol, 1.0 equiv.), potassium benzoate (0.75 mmol, 1.5 equiv.) and **BuPPT** (5.0 mol%) were added under argon atmosphere. The flask was evacuated and purged with argon 3 times. Acetonitrile (4.5 mL) and water (0.5 mL) were subsequently added under inert atmosphere. The reaction mixture was stirred for 24 h under irradiation at 396 nm. Subsequently, the sample was analyzed by TLC and calibrated GC using biphenyl as internal standard. No conversion was observed. Consequently, an ET mechanism as the turnover-determining step can be excluded.

Reactivity in the absence of base

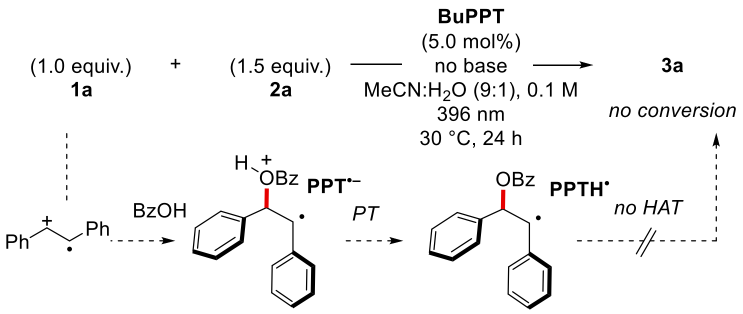

Scheme S 3. Model reaction without base

In a preheated, dry, 10 mL Schlenk flask equipped with a magnetic stir bar, *trans*-stilbene (**1a**) (0.5 mmol, 1.0 equiv.), benzoic acid (**2a**) (0.75 mmol, 1.5 equiv.) and **BuPPT** (5.0 mol%) were added under argon. The flask was then evacuated and purged with argon for 3 times. Acetonitrile (4.5 mL) and water (0.5 mL) were subsequently added under inert atmosphere. The reaction mixture was stirred for 24 h under irradiation at 396 nm. Subsequently the sample was analyzed by TLC and calibrated GC using biphenyl as internal standard, indicating that no product was formed. Therefore, a base-independent HAT as the turnover step can be excluded.

Long-time experiments

Reactions were set-up following general procedure A, using (*E*)-stilbene (**1a**) and cyclohexane carboxylic acid (**4h**) and adamantane carboxylic acid (**4h**), respectively. The reactions were irradiated at 396 nm for 72 h. The crude reaction mixture was filtered through a plug of Na_2SO_4 and a GC-MS chromatogram was recorded which is shown in Figure S 3 and Figure S 4, below.

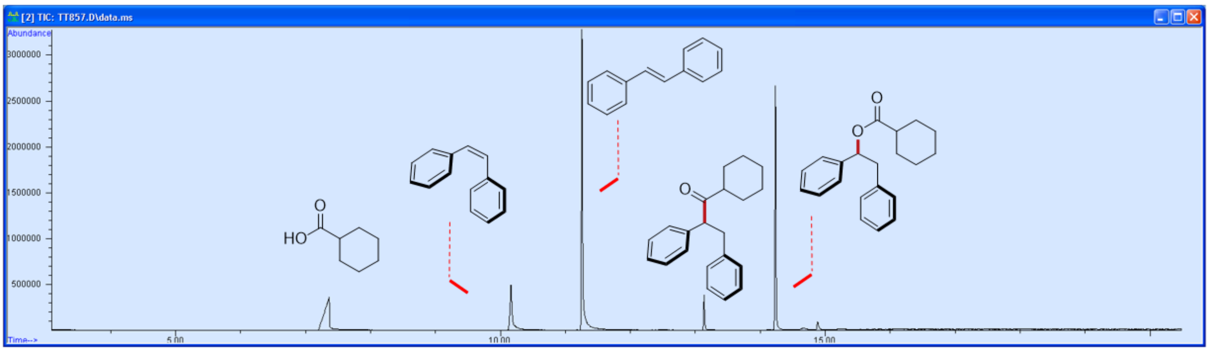

Figure S 3. GC-MS chromatogram of the crude reaction mixture of (*E*)-stilbene (**1a**) and cyclohexane carboxylic acid (**4h**) after irradiation for 72 h.

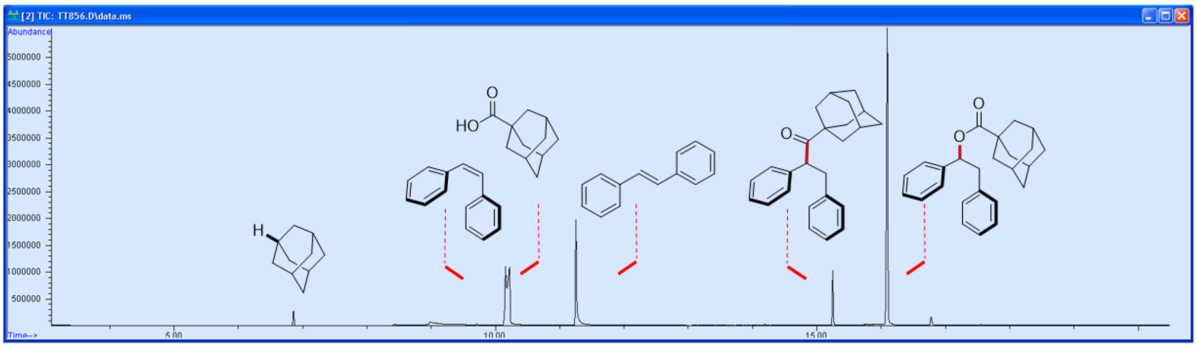

Figure S 4. GC-MS chromatogram of the crude reaction mixture of (*E*)-stilbene (**1a**) and adamantane carboxylic acid (**4k**) after irradiation for 72 h.

Sensitivity assessment

In a preheated, dry, 10 mL Schlenk flask equipped with a magnetic stir bar, *trans*-stilbene (**1a**) (0.5 mmol, 1.0 equiv.), benzoic acid (**2a**) (0.75 mmol, 1.5 equiv.), **BuPPT** (5.0 mol%) and the indicated additive (20 mol%) were added under argon atmosphere. The flask was evacuated and purged with argon 3 times. Acetonitrile (4.5 mL) and water (0.5 mL) were subsequently added under inert atmosphere. The reaction mixture was stirred for 24 h under irradiation at 396 nm. Subsequently, the crude reaction mixture was extracted and the product **3a** was isolated by flash chromatography.

Table S 10. Sensitivity assessment of the **BuPPT** catalyzed photo-hydroacetoxylation.

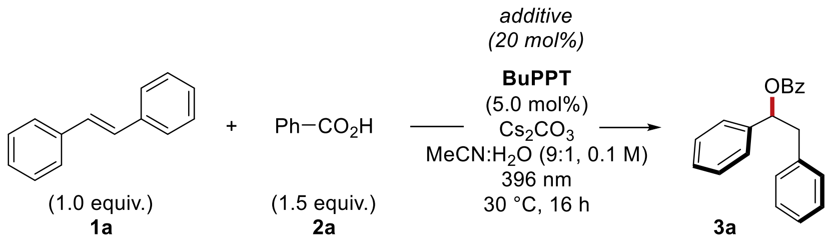

entry	additive	amount of additive [mol%]	yield [%][a]
1	none	-	89
2	acetophenone (**A**)	20	91
3	furan (**B**)	20	87
4	1,2-diphenylethan-1-ol (**8**)	20	63
5	cyclohexyl carboxylic acid (**4h**)	20	60

[a] Cs_2CO_3 (1.5 equiv.) was used as base.Isolated yields are shown.

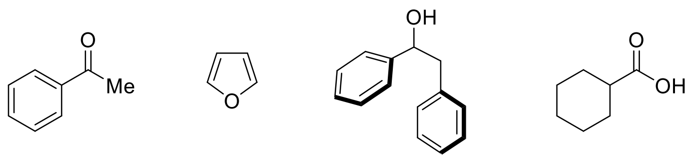

A **B** **8** **4h**

NMR Spectra

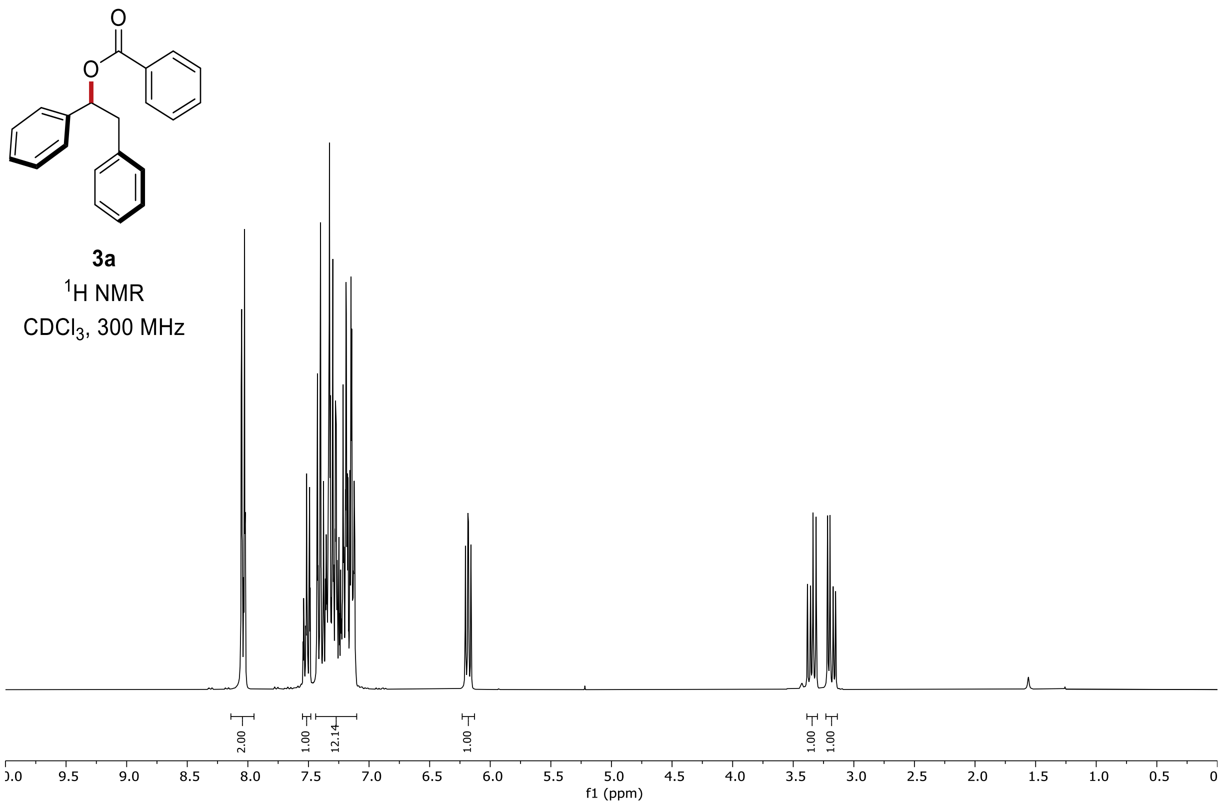
3a
^{1}H NMR
CDCl$_3$, 300 MHz
2.00
1.00
12.14
1.00
1.00
1.00
f1 (ppm)

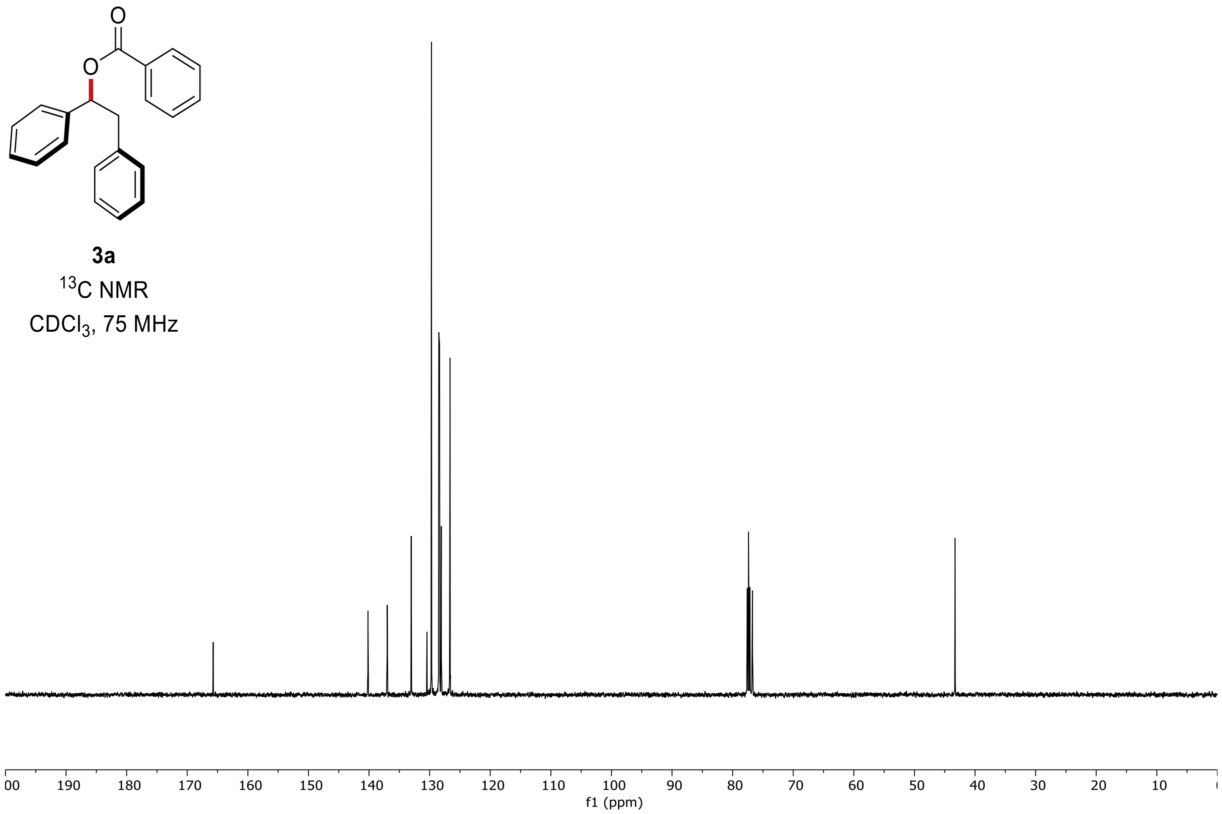
3a
^{13}C NMR
CDCl$_3$, 75 MHz
f1 (ppm)

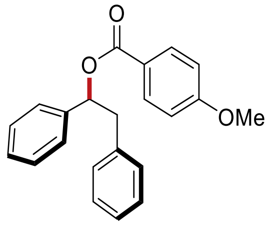

3b
^{1}H NMR
$CDCl_3$, 300 MHz

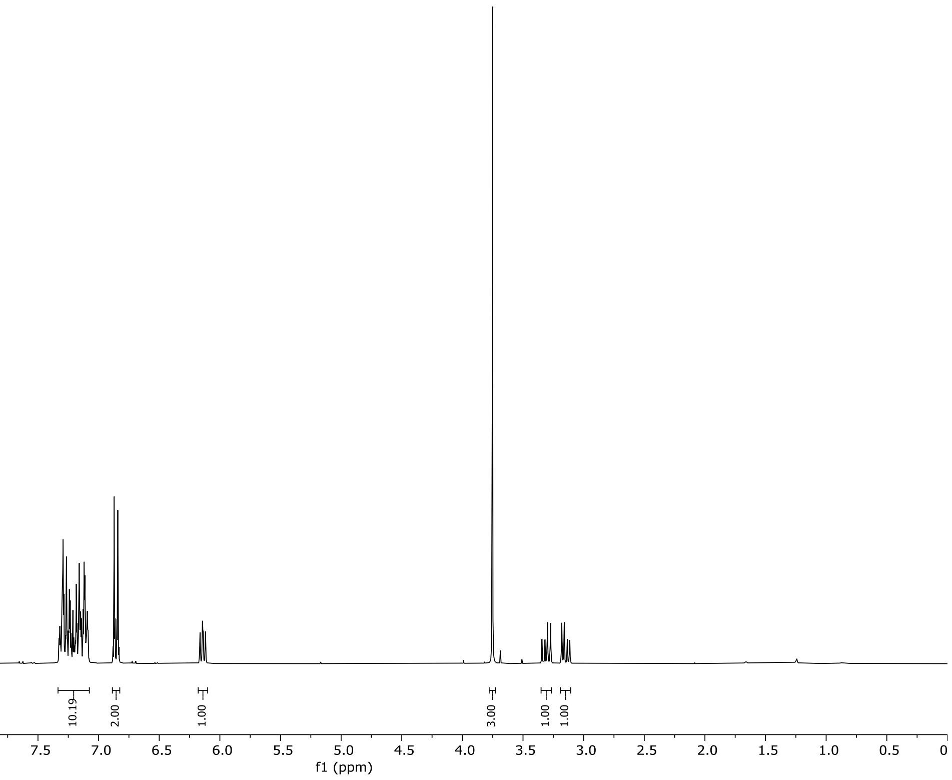

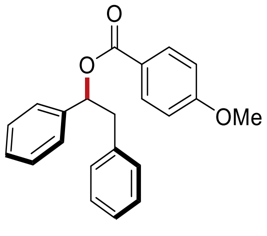

3b
^{13}C NMR
$CDCl_3$, 75 MHz

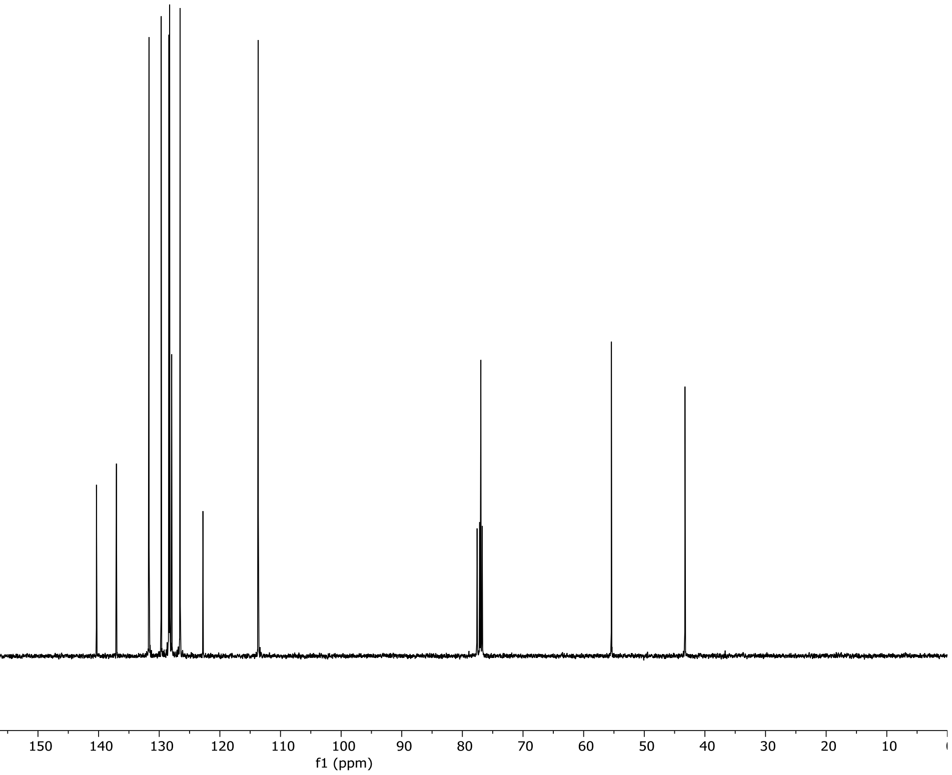

3c
^{1}H NMR
$CDCl_3$, 300 MHz

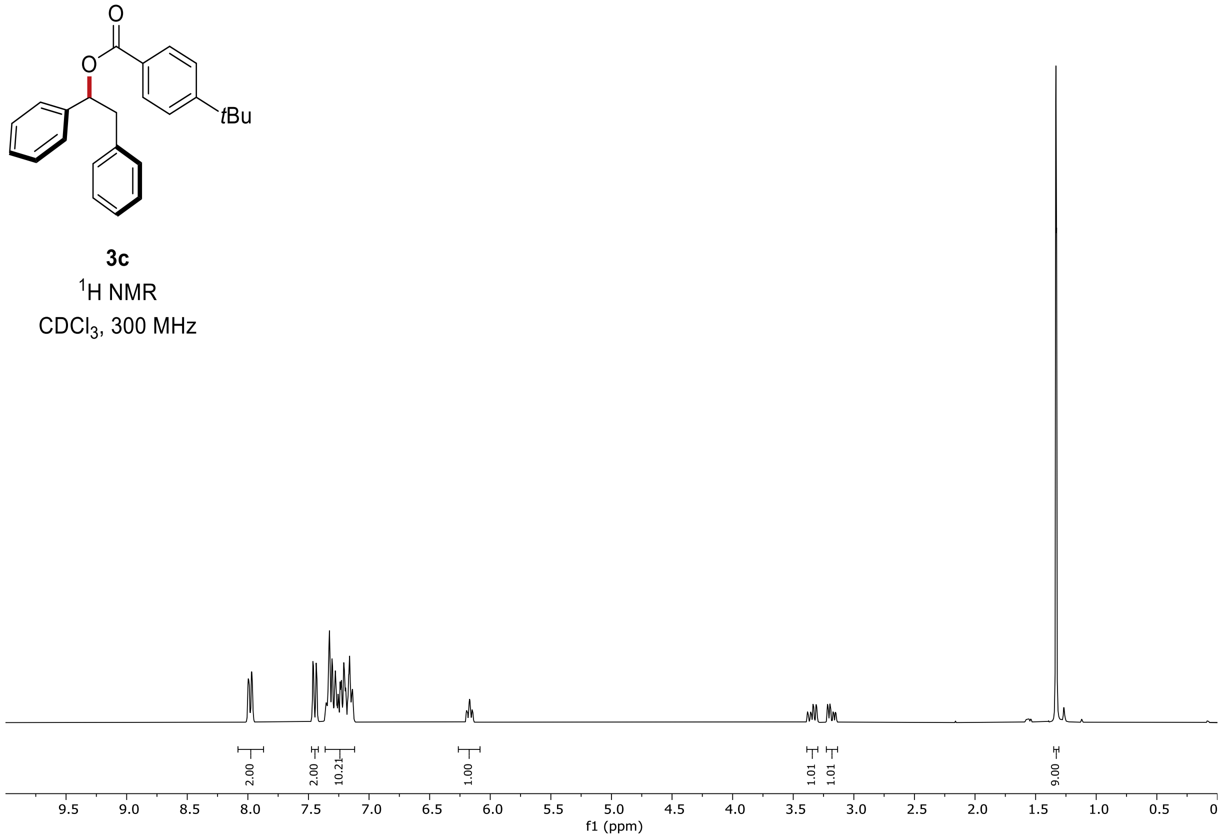

3c
^{13}C NMR
$CDCl_3$, 75 MHz

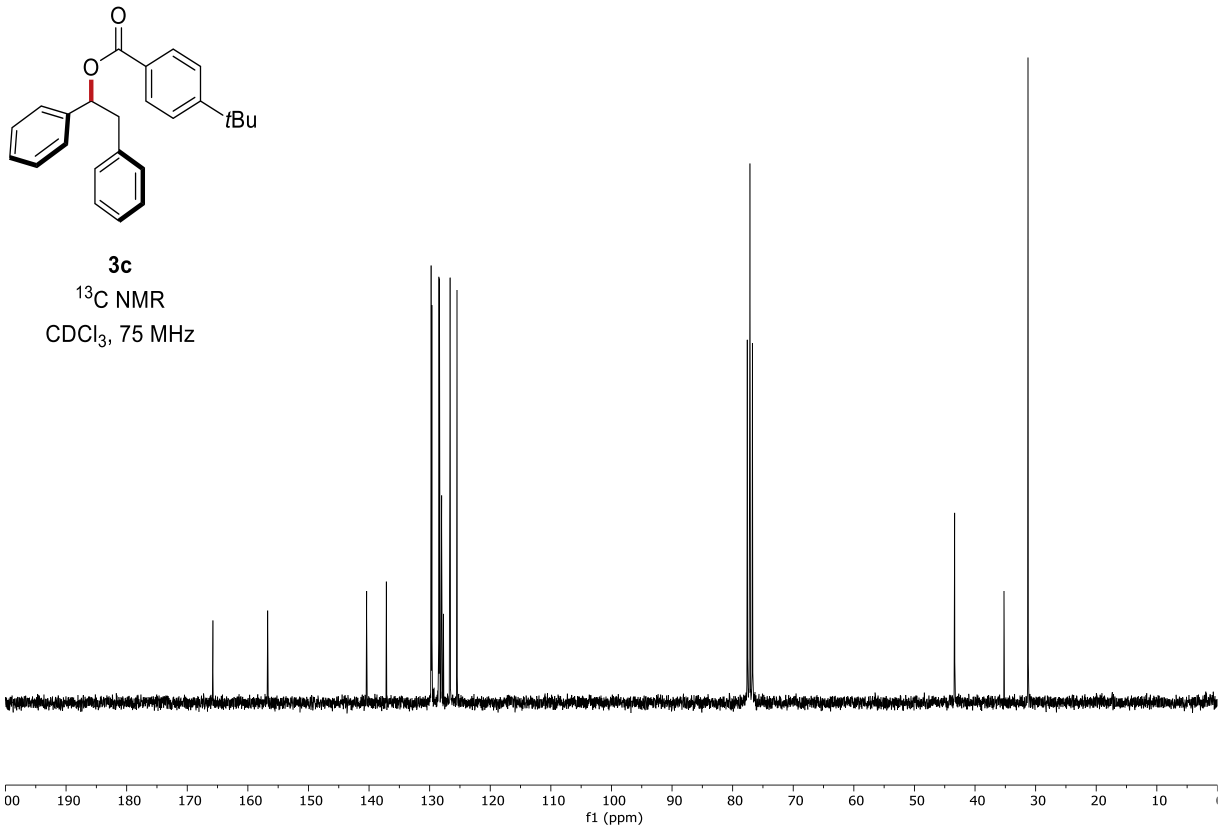

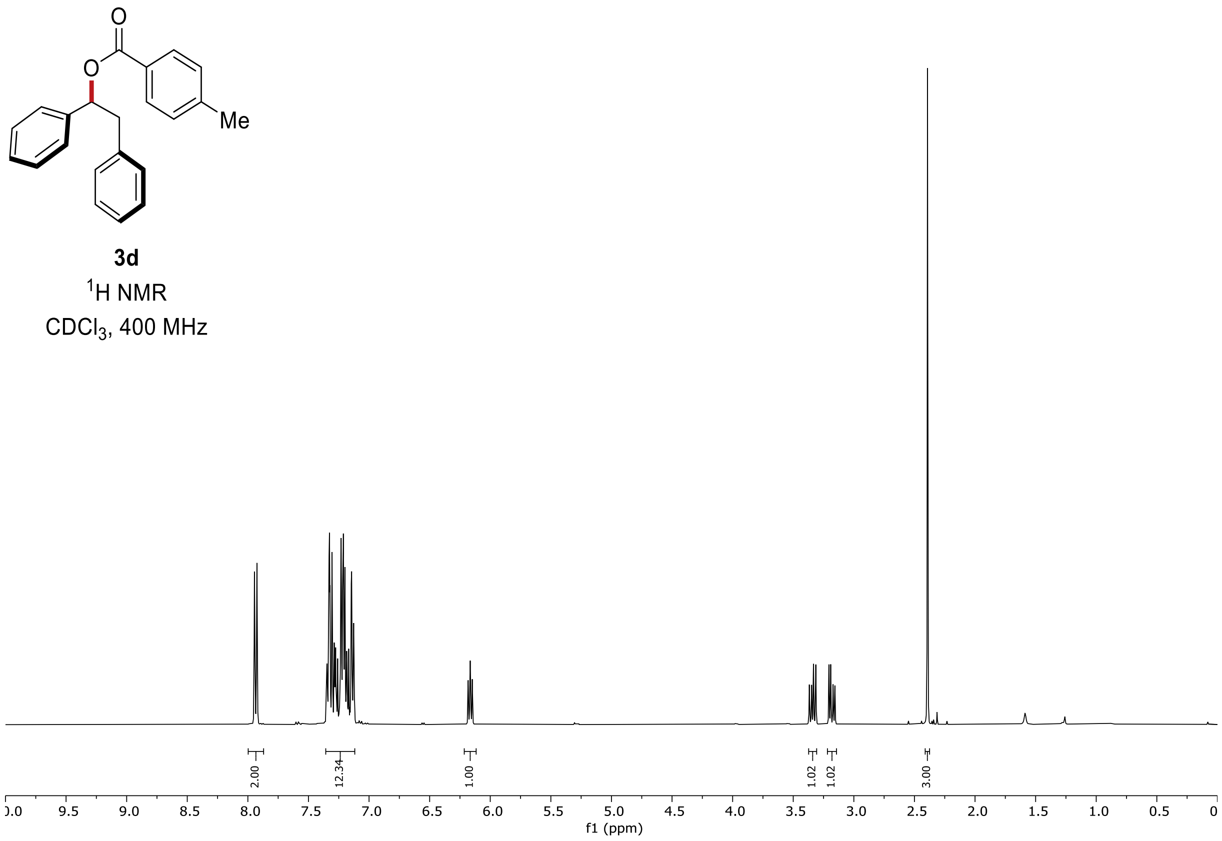
O
O
Me
3d
^{1}H NMR
CDCl$_3$, 400 MHz
2.00
12.34
1.00
1.02
1.02
3.00
f1 (ppm)

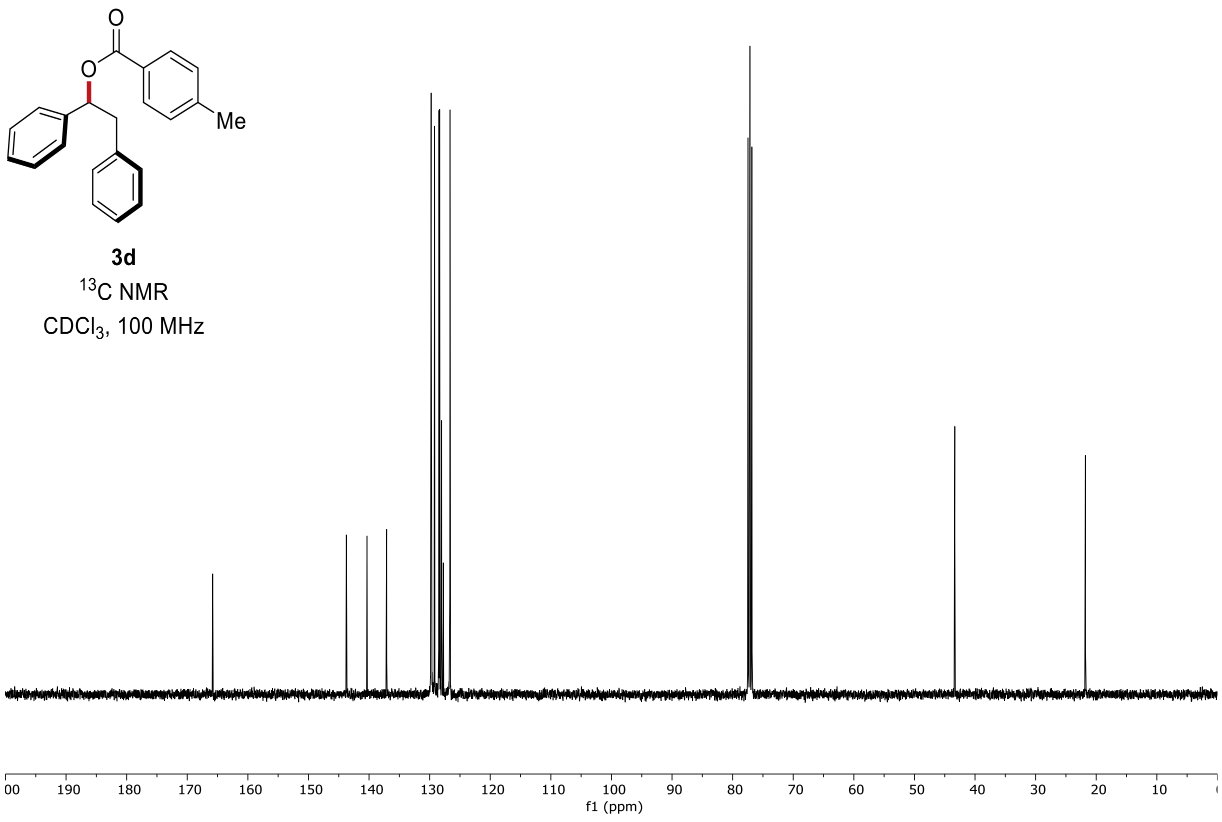
O
O
Me
3d
^{13}C NMR
CDCl$_3$, 100 MHz
f1 (ppm)

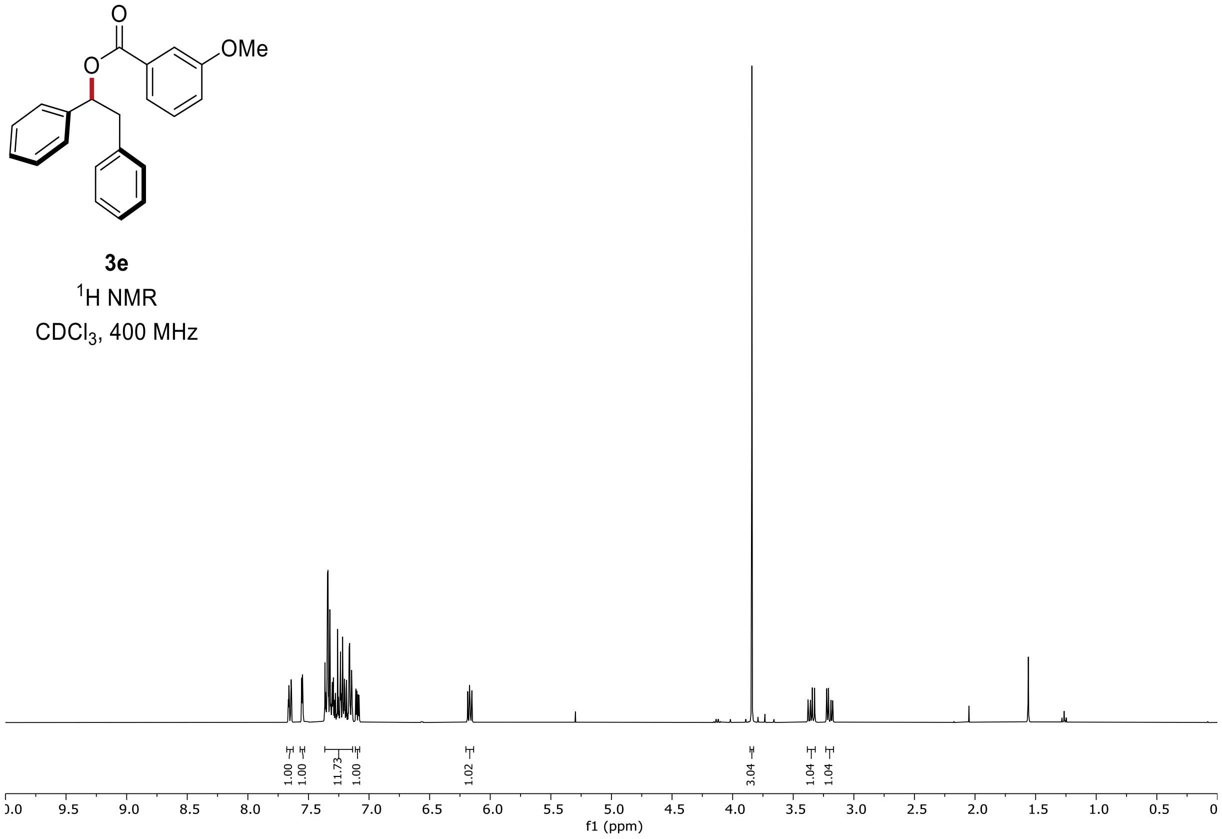

3e
^{1}H NMR
$CDCl_3$, 400 MHz

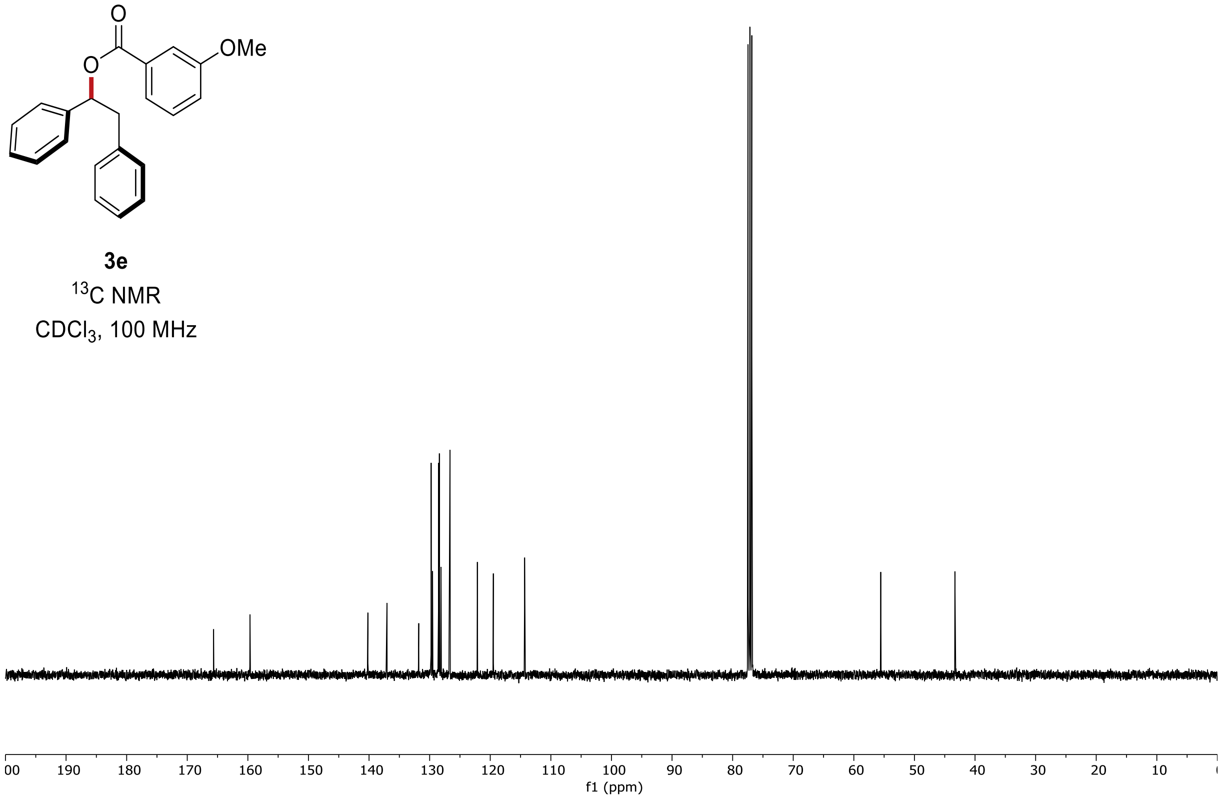

3e
^{13}C NMR
$CDCl_3$, 100 MHz

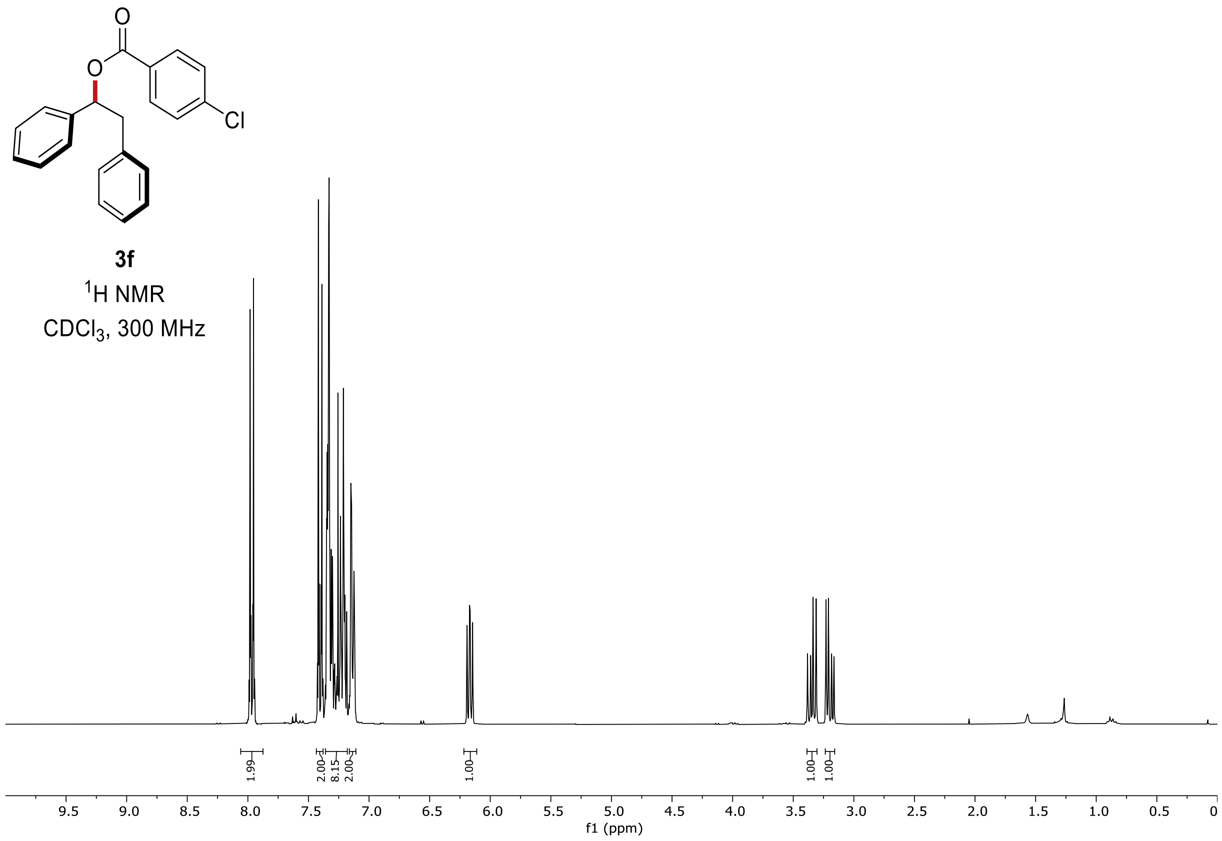

Cl
3f
^{1}H NMR
$CDCl_3$, 300 MHz
1.99
2.00
8.15
2.00
1.00
1.00
1.00
f1 (ppm)

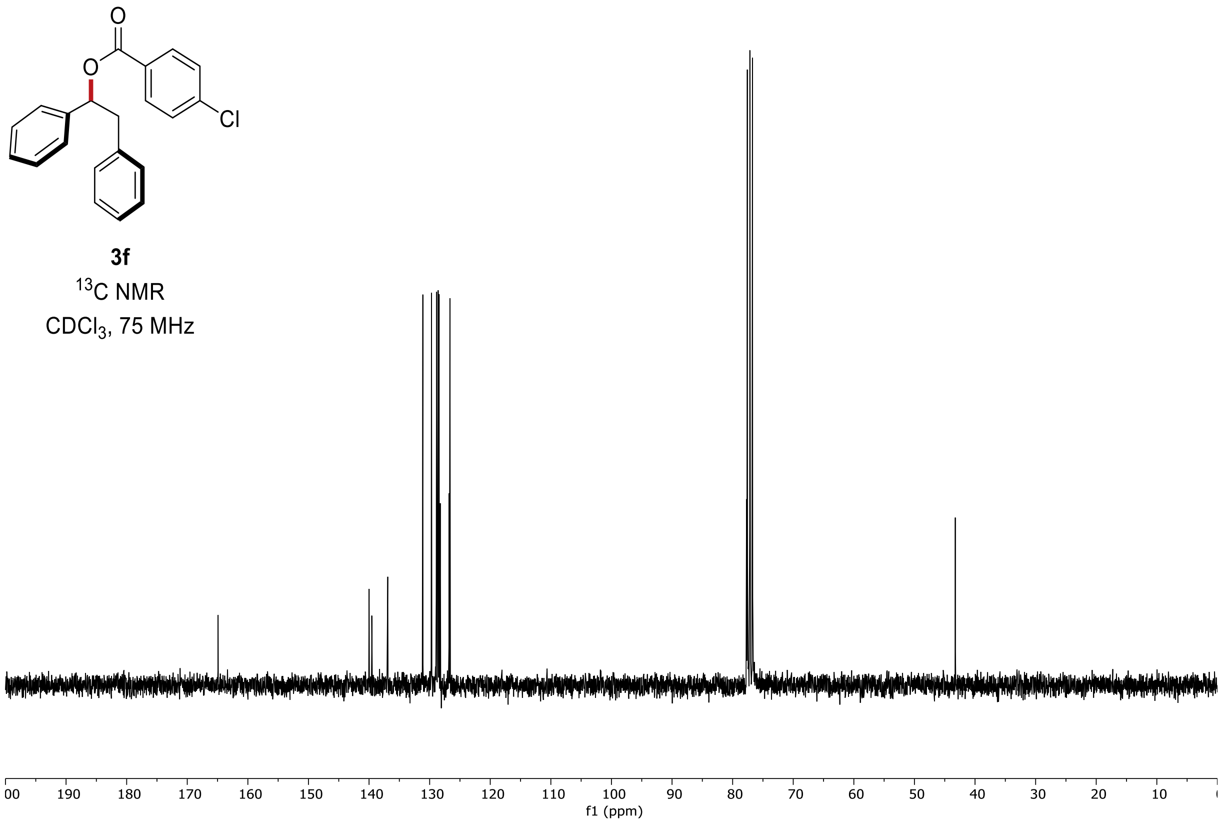

Cl
3f
^{13}C NMR
$CDCl_3$, 75 MHz
f1 (ppm)

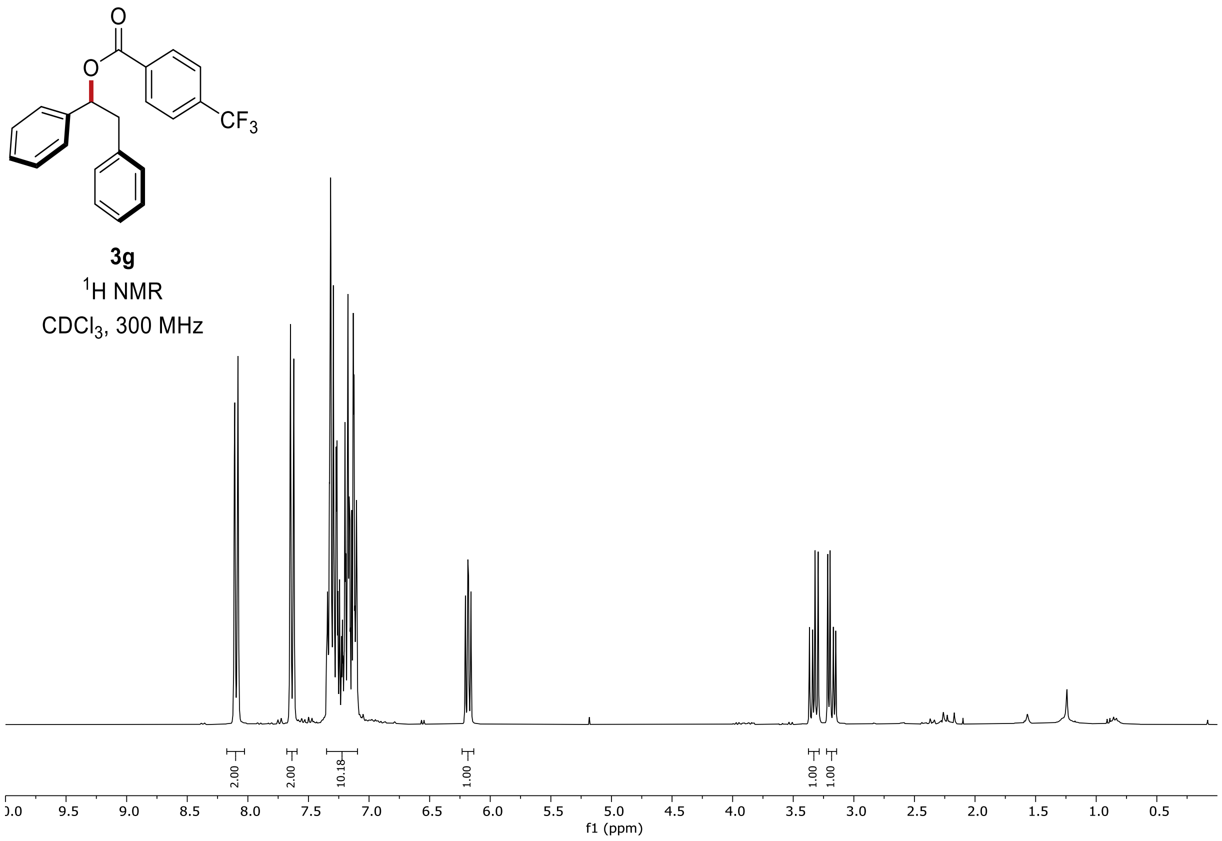
O
O
CF3
3g
1H NMR
CDCl3, 300 MHz
2.00
2.00
10.18
1.00
1.00
1.00
f1 (ppm)

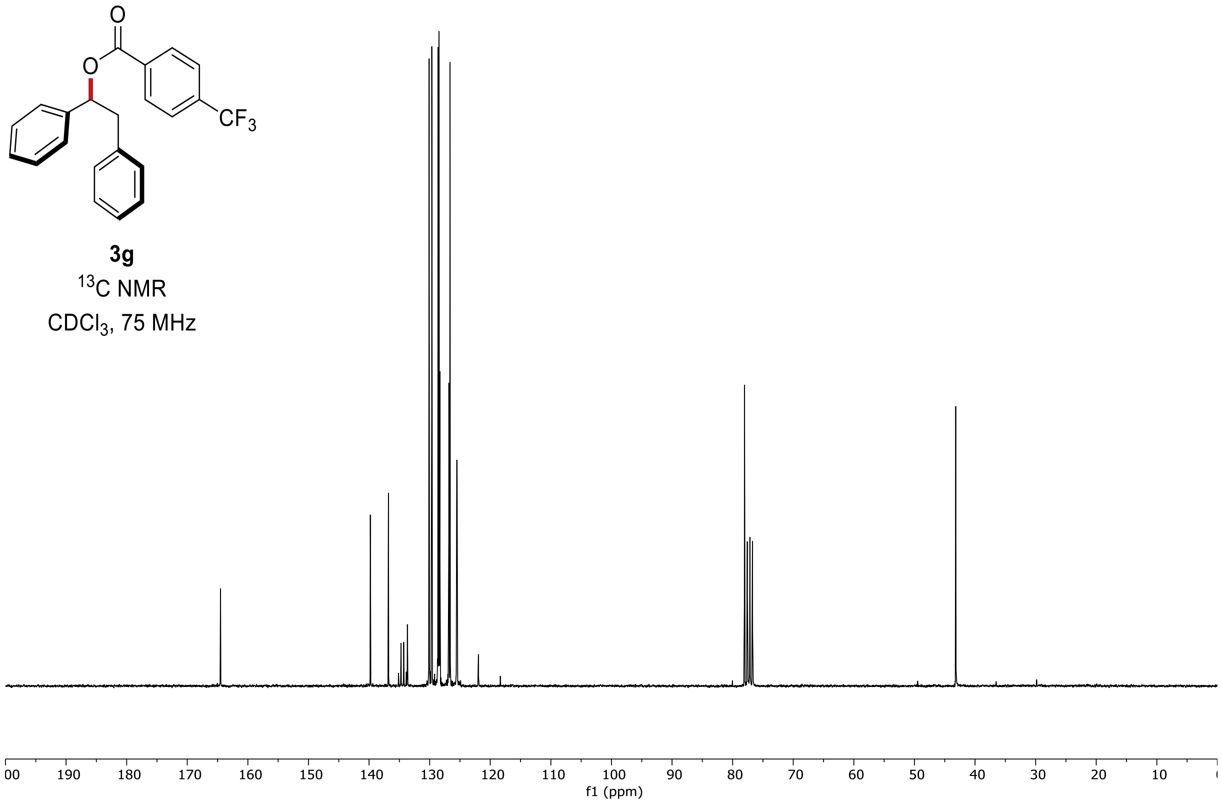
O
O
CF3
3g
13C NMR
CDCl3, 75 MHz
f1 (ppm)

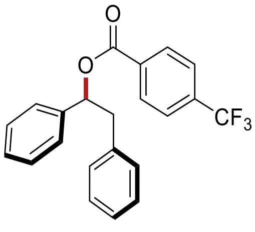

3g

^{19}F NMR

$CDCl_3$, 376 MHz

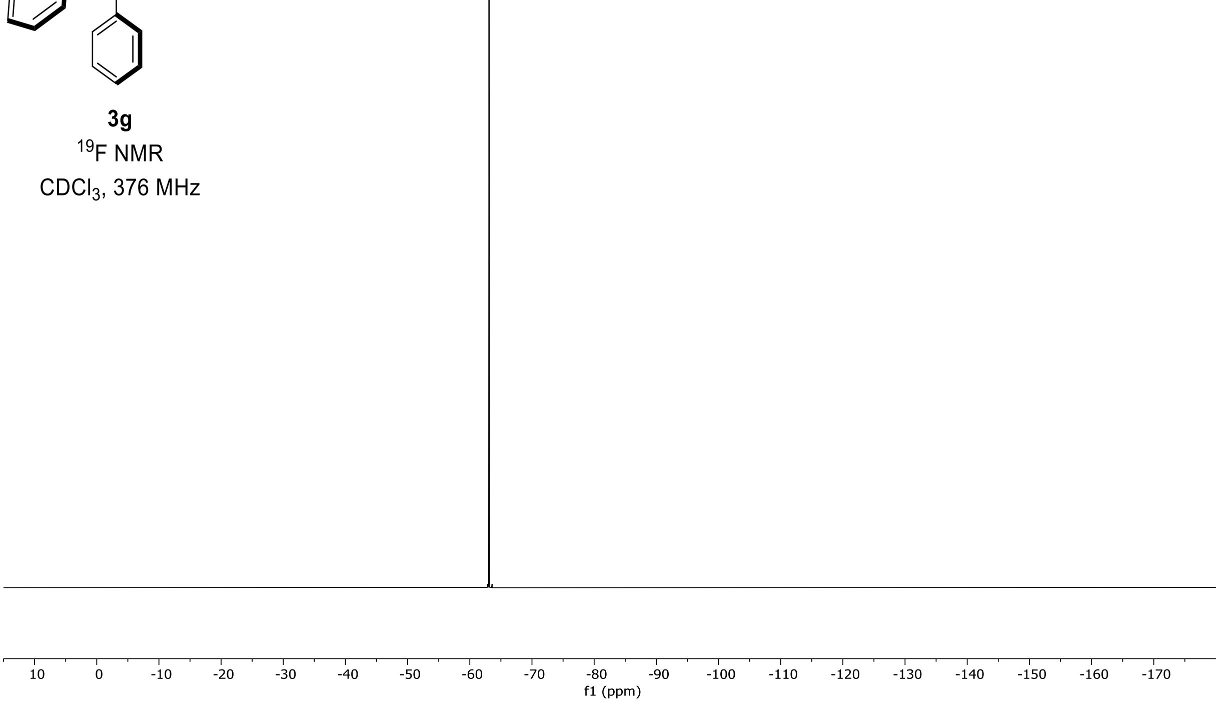

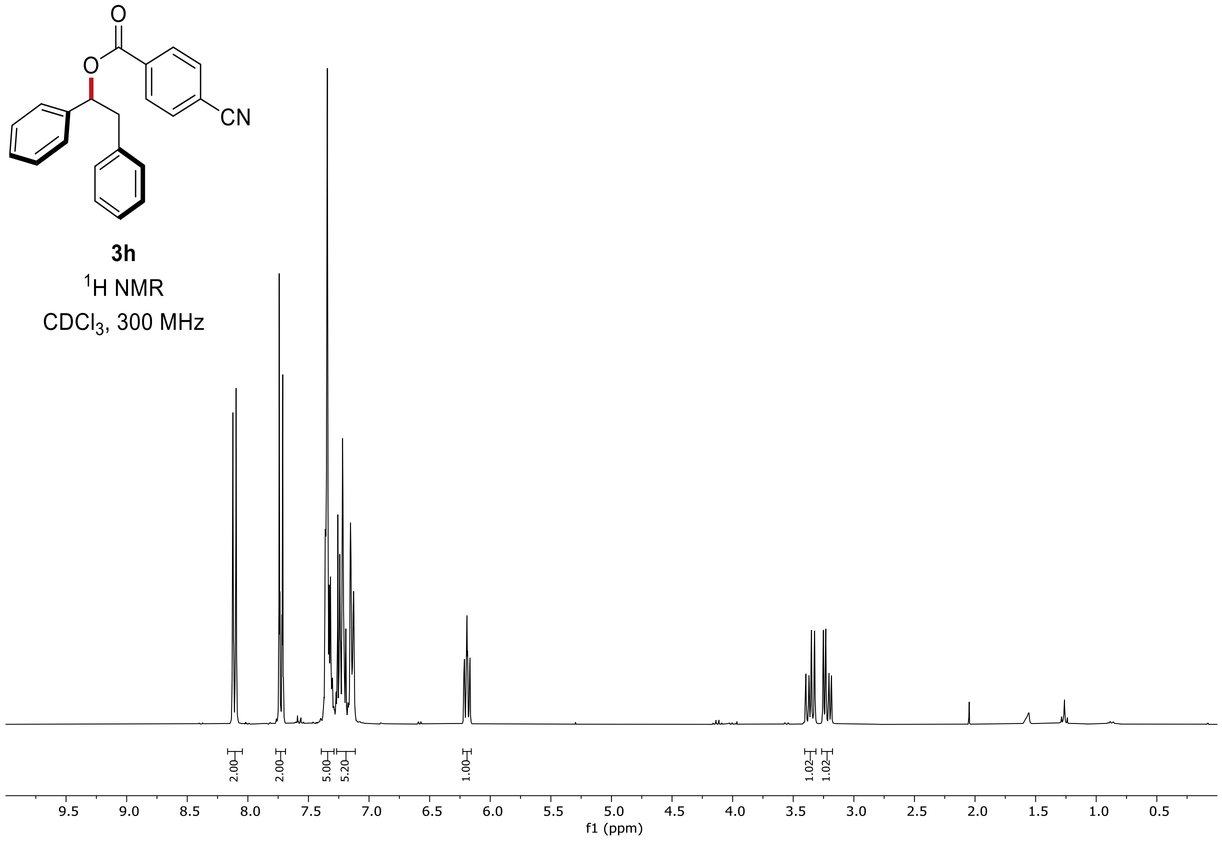
O
O
CN
3h
1H NMR
CDCl3, 300 MHz
2.00
2.00
5.00
5.20
1.00
1.02
1.02
9.5 9.0 8.5 8.0 7.5 7.0 6.5 6.0 5.5 5.0 4.5 4.0 3.5 3.0 2.5 2.0 1.5 1.0 0.5
f1 (ppm)

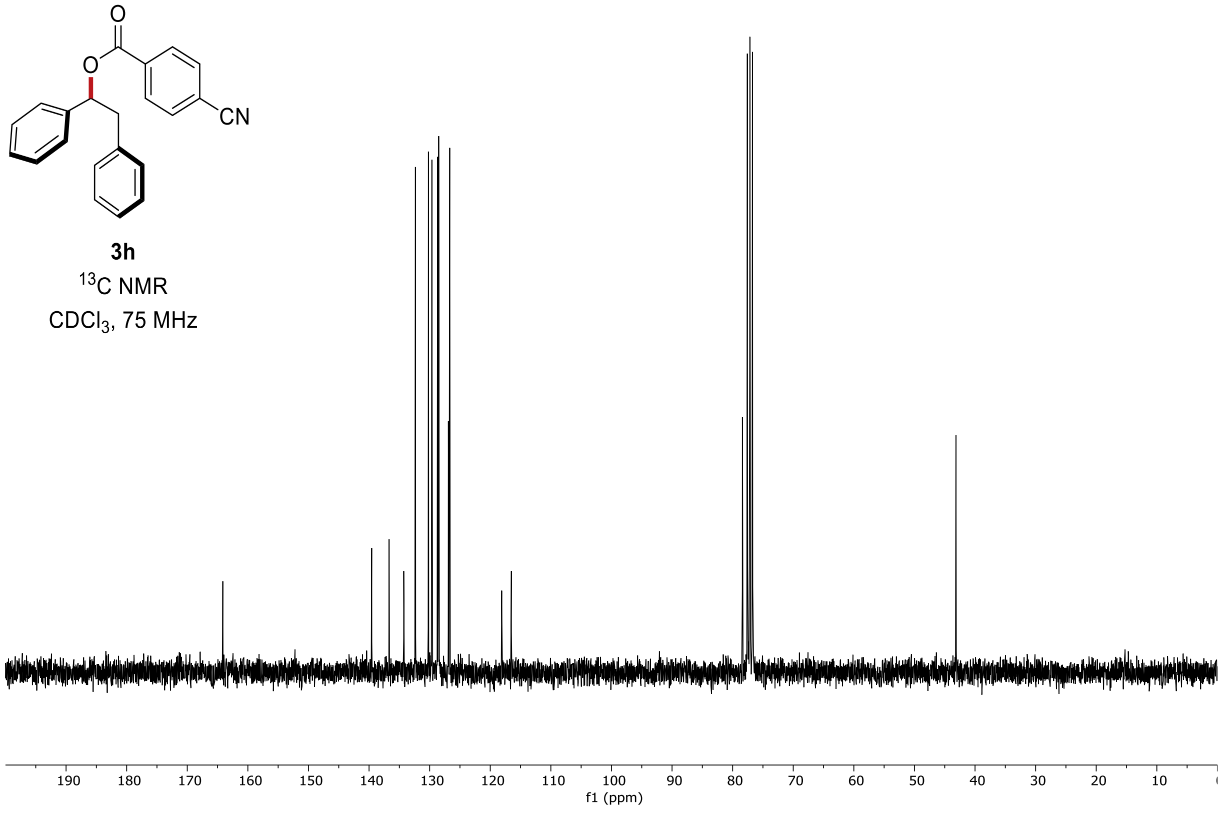
O
O
CN
3h
13C NMR
CDCl3, 75 MHz
190 180 170 160 150 140 130 120 110 100 90 80 70 60 50 40 30 20 10
f1 (ppm)

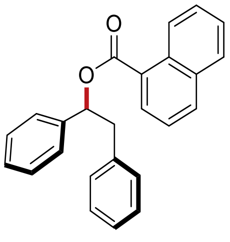

3i
^{1}H NMR
$CDCl_3$, 300 MHz

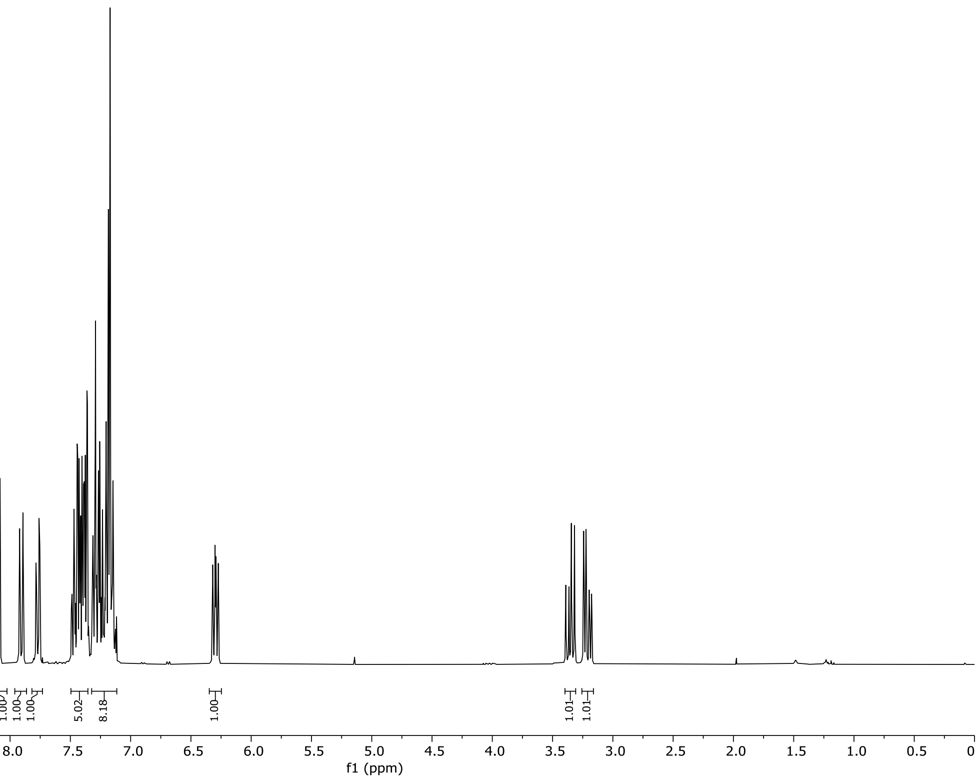

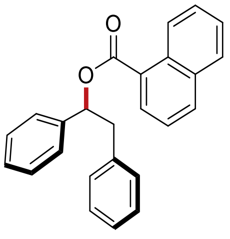

3i
^{13}C NMR
$CDCl_3$, 75 MHz

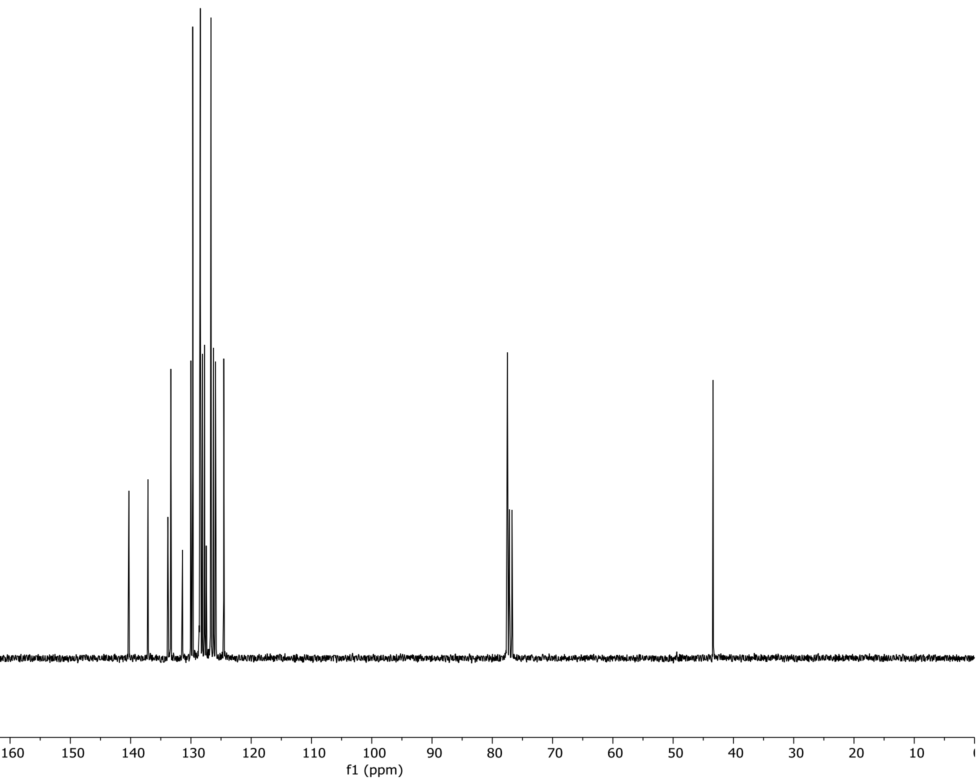

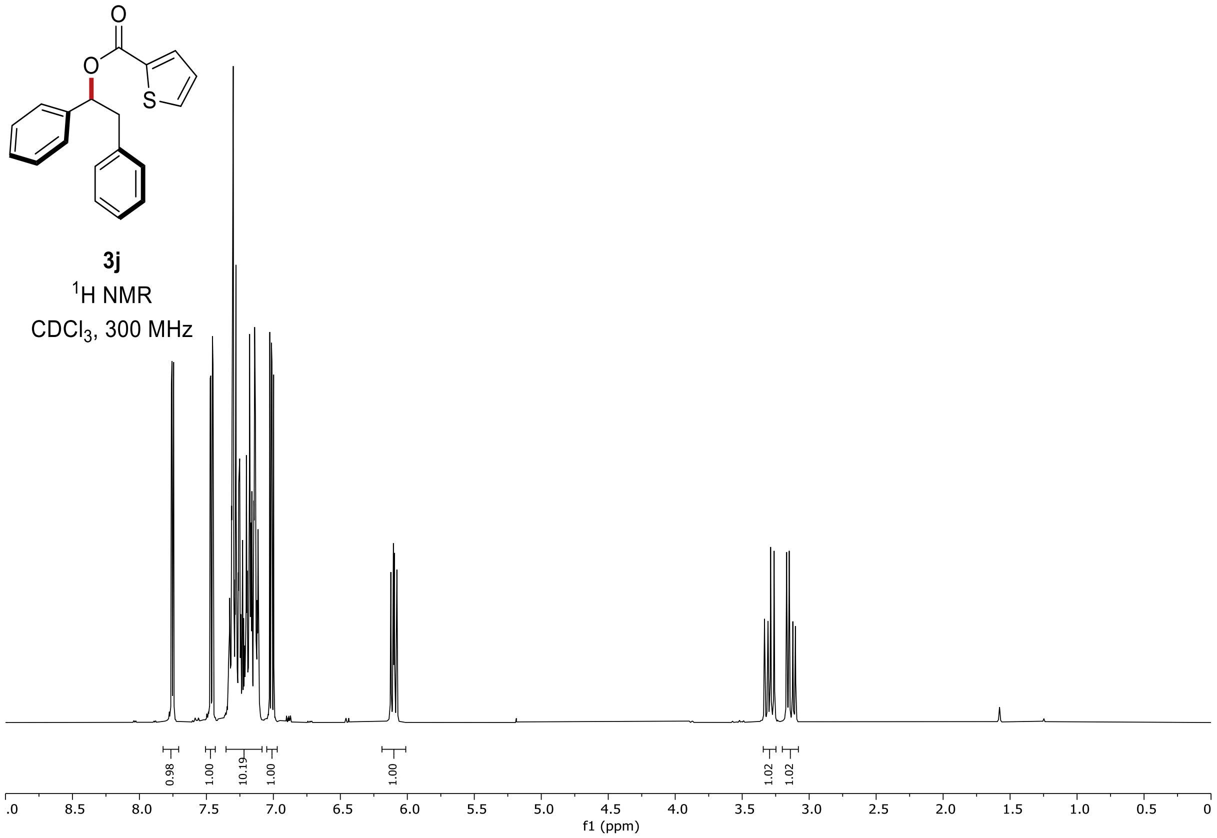

3j
^{1}H NMR
$CDCl_3$, 300 MHz

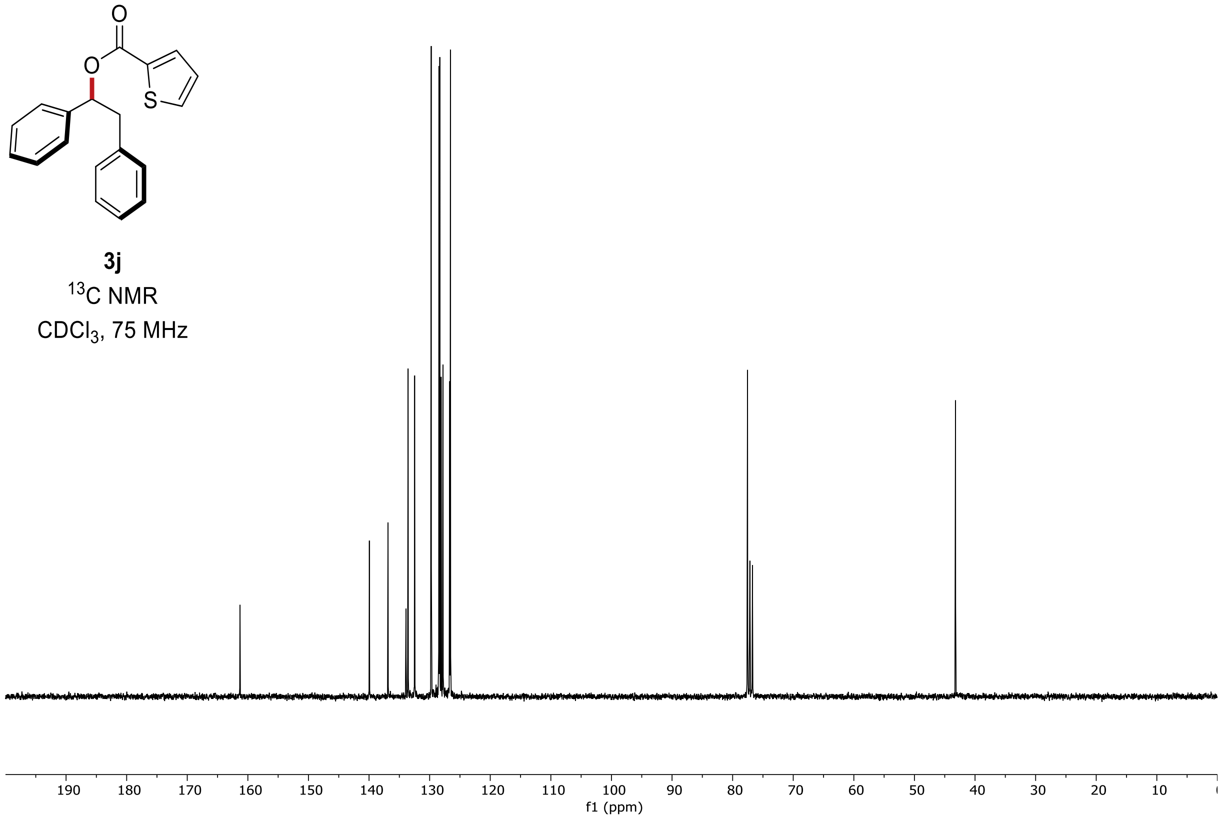

3j
^{13}C NMR
$CDCl_3$, 75 MHz

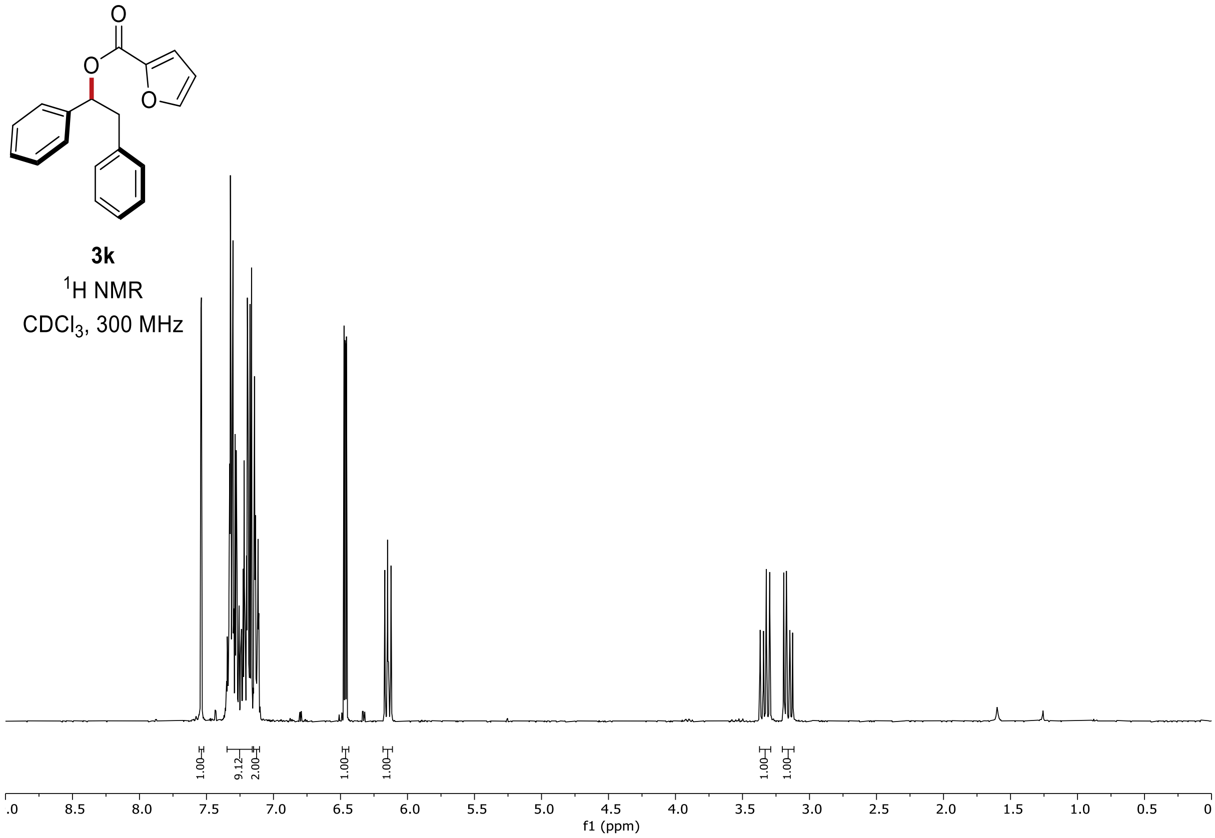
O
O
O
3k
1H NMR
CDCl3, 300 MHz
1.00
9.12
2.00
1.00
1.00
1.00
1.00
f1 (ppm)

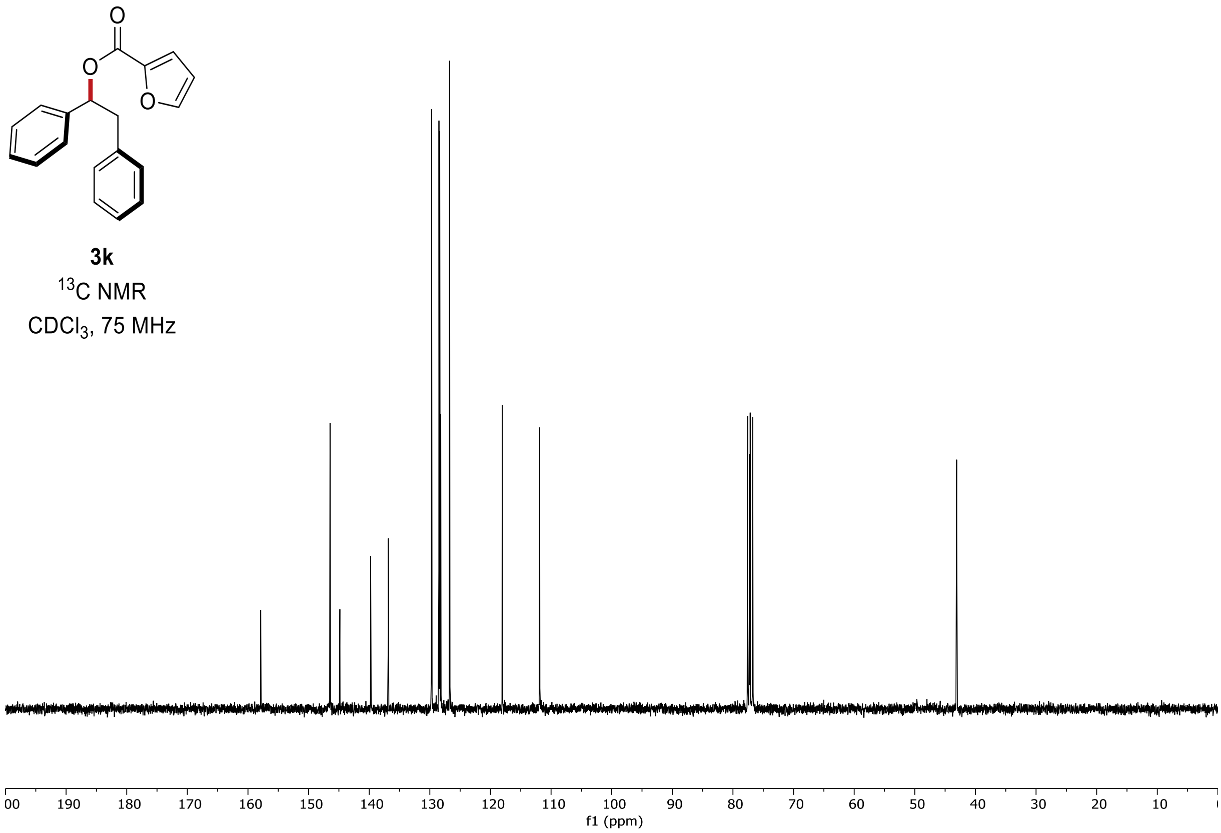
O
O
O
3k
13C NMR
CDCl3, 75 MHz
f1 (ppm)

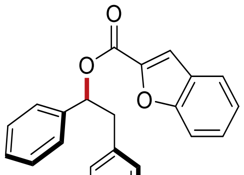

3l

^{1}H NMR

$CDCl_3$, 300 MHz

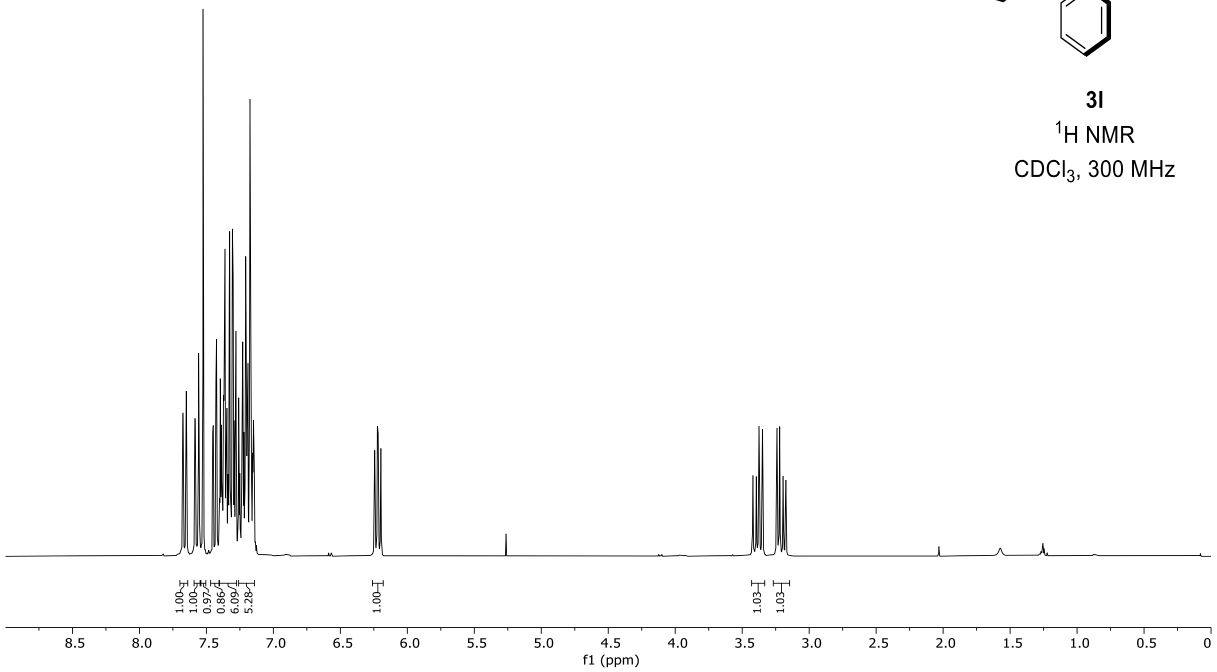

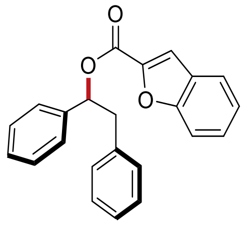

3l

^{13}C NMR

$CDCl_3$, 75 MHz

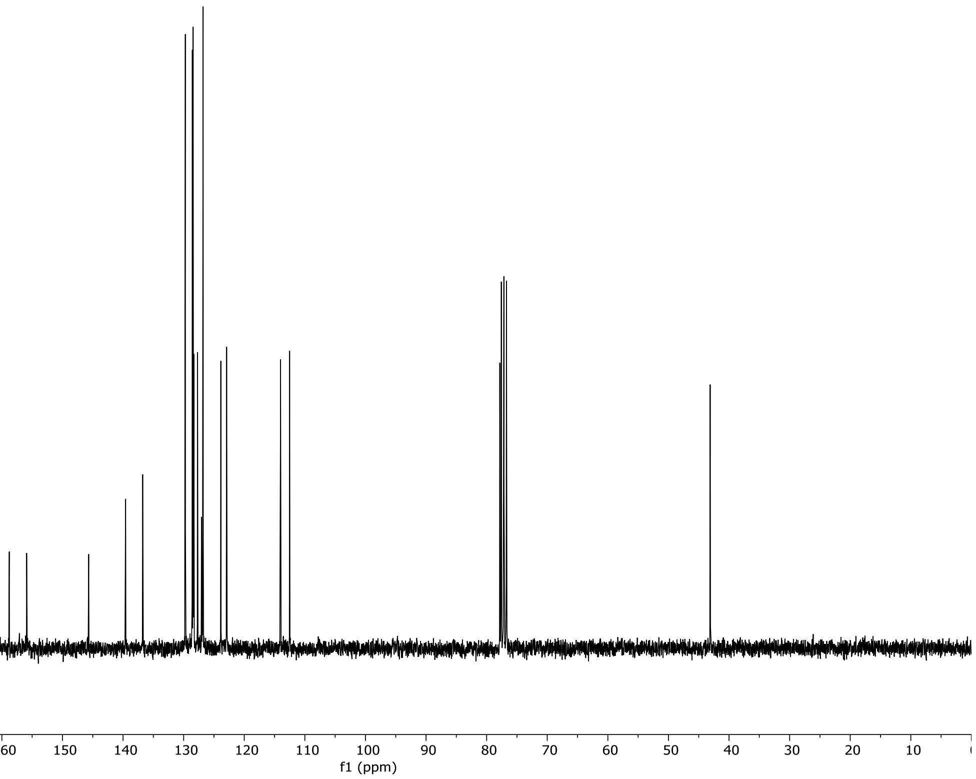

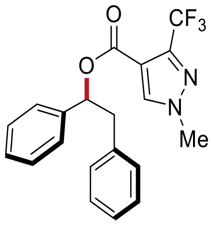

3m
^{1}H NMR
$CDCl_3$, 400 MHz

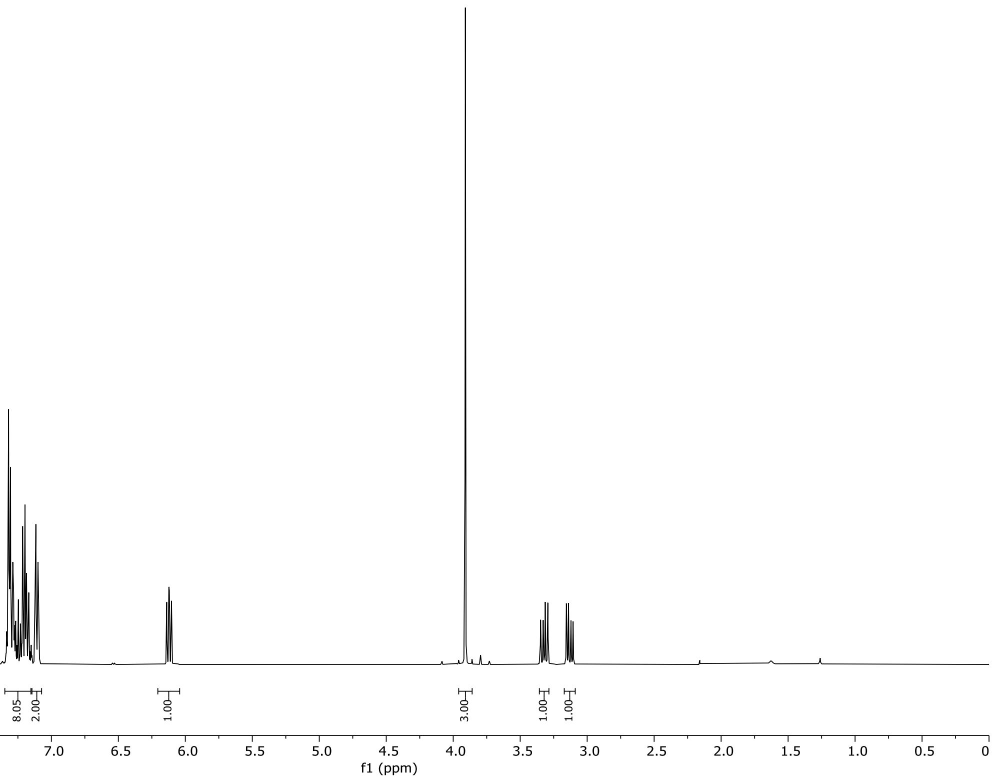

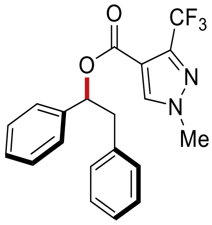

3m
^{13}C NMR
$CDCl_3$, 75 MHz

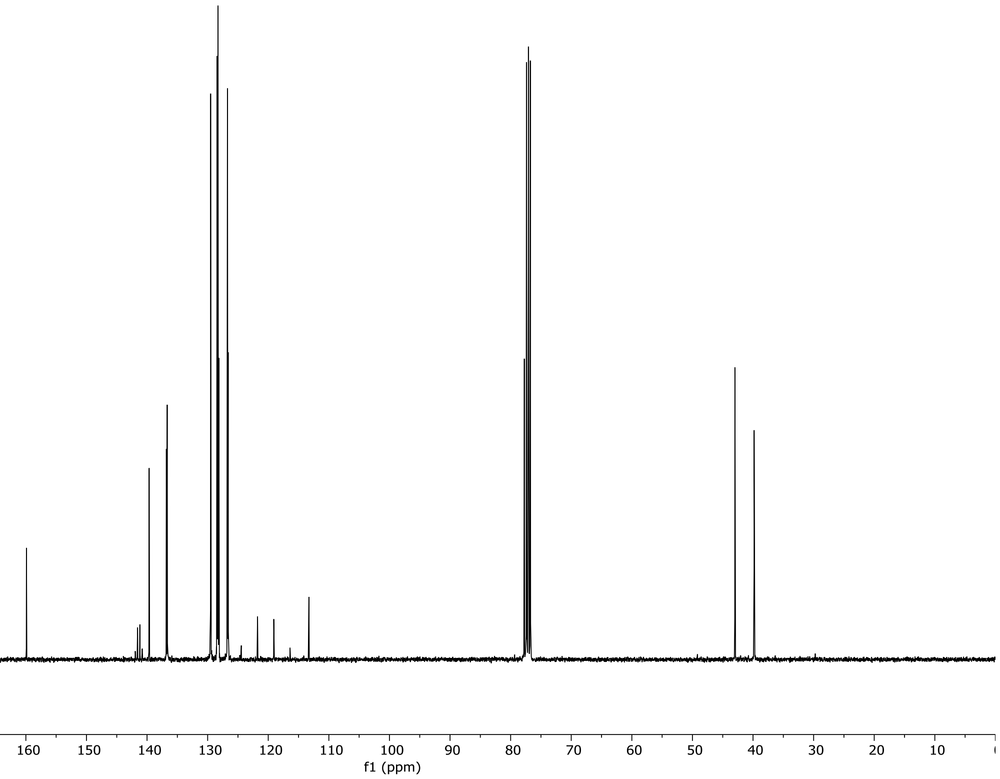

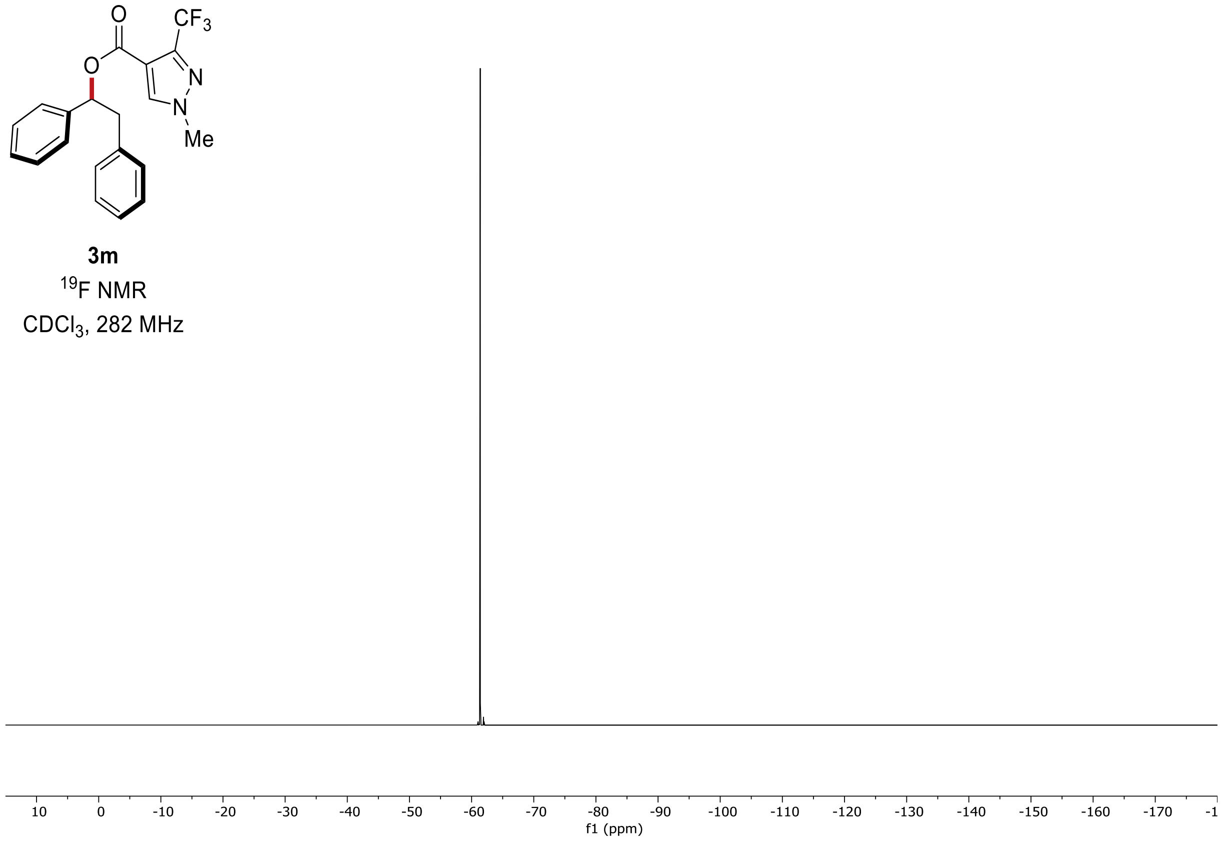

3m

^{19}F NMR

$CDCl_3$, 282 MHz

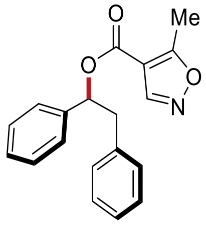

3n
^{1}H NMR
$CDCl_3$, 300 MHz

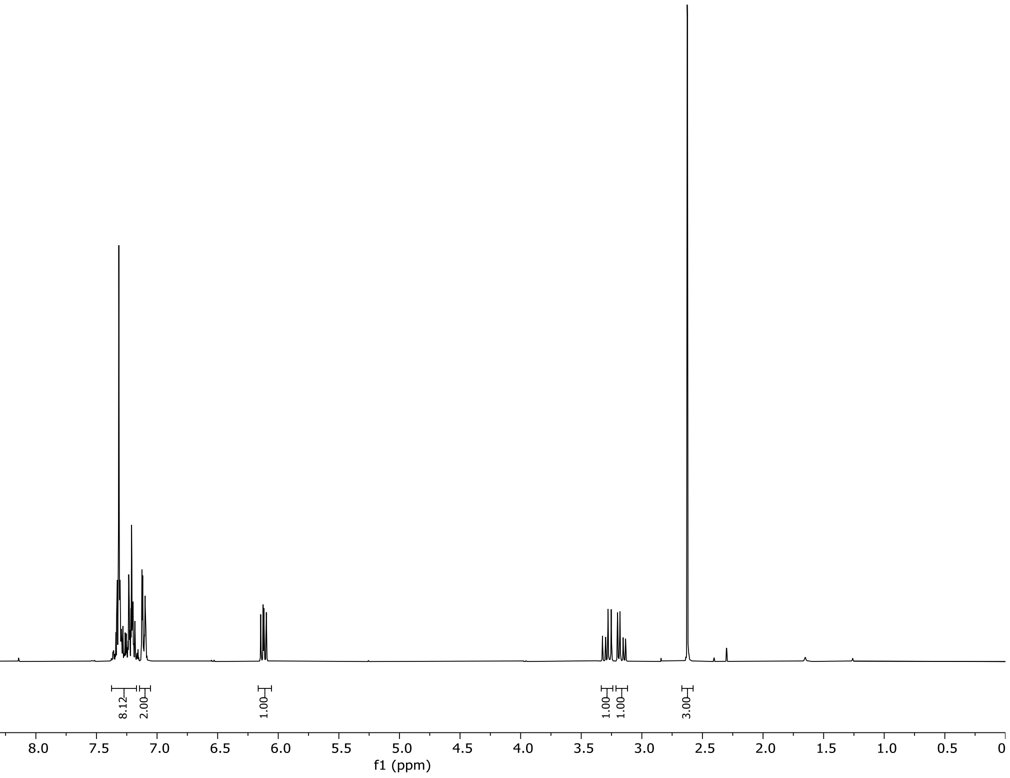

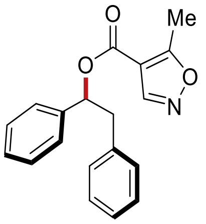

3n
^{13}C NMR
$CDCl_3$, 75 MHz

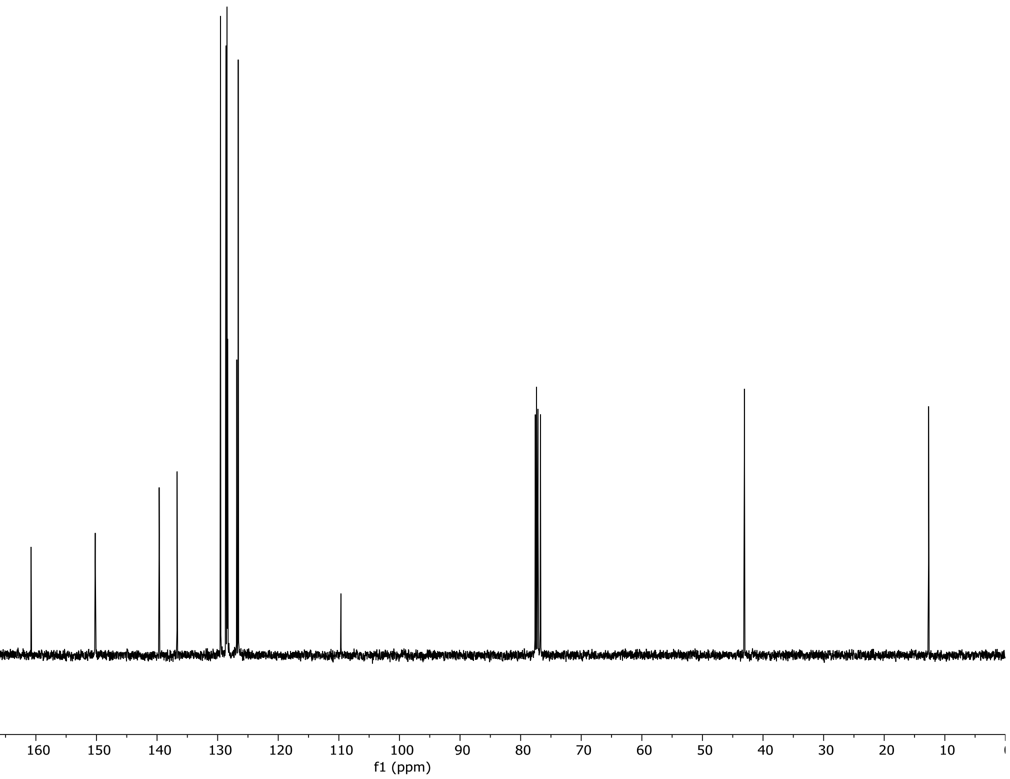

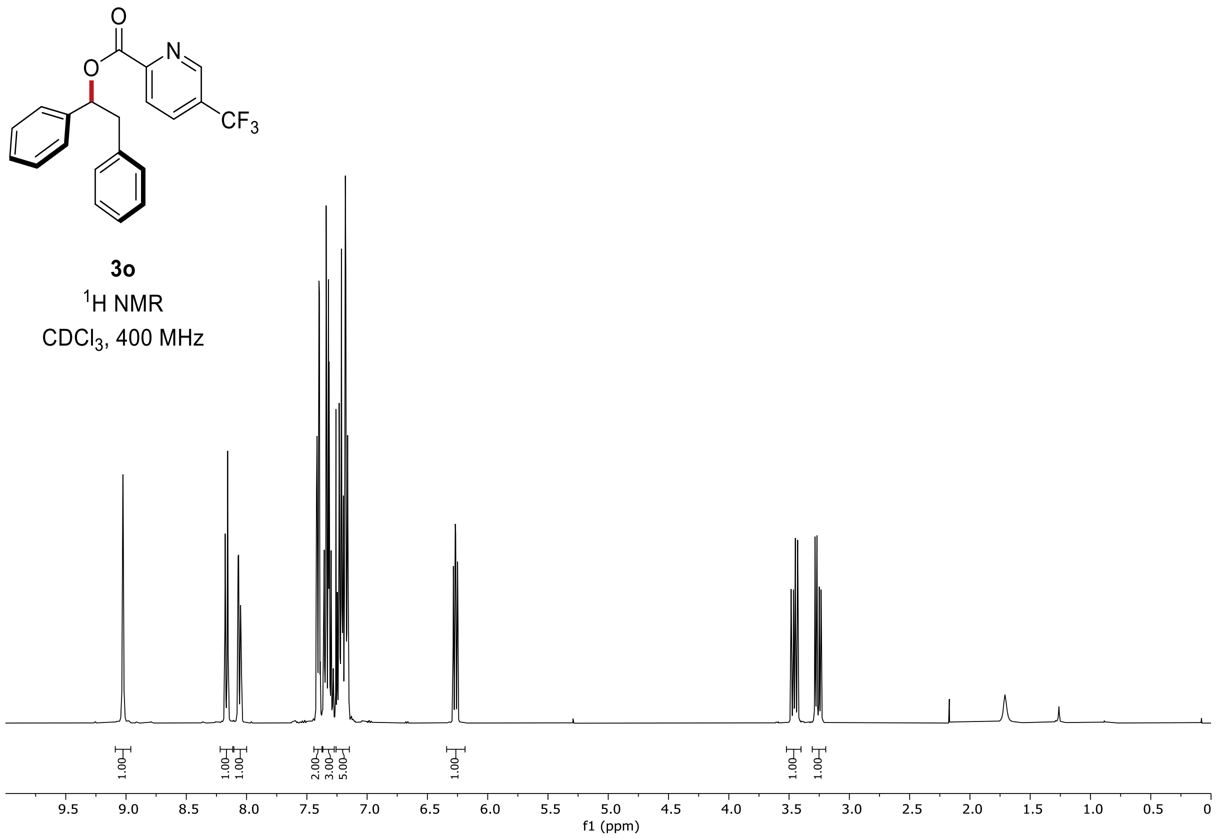

3o
1H NMR
CDCl3, 400 MHz
1.00
1.00
1.00
2.00
3.00
5.00
1.00
1.00
1.00
9.5
9.0
8.5
8.0
7.5
7.0
6.5
6.0
5.5
5.0
4.5
4.0
3.5
3.0
2.5
2.0
1.5
1.0
0.5
0
f1 (ppm)

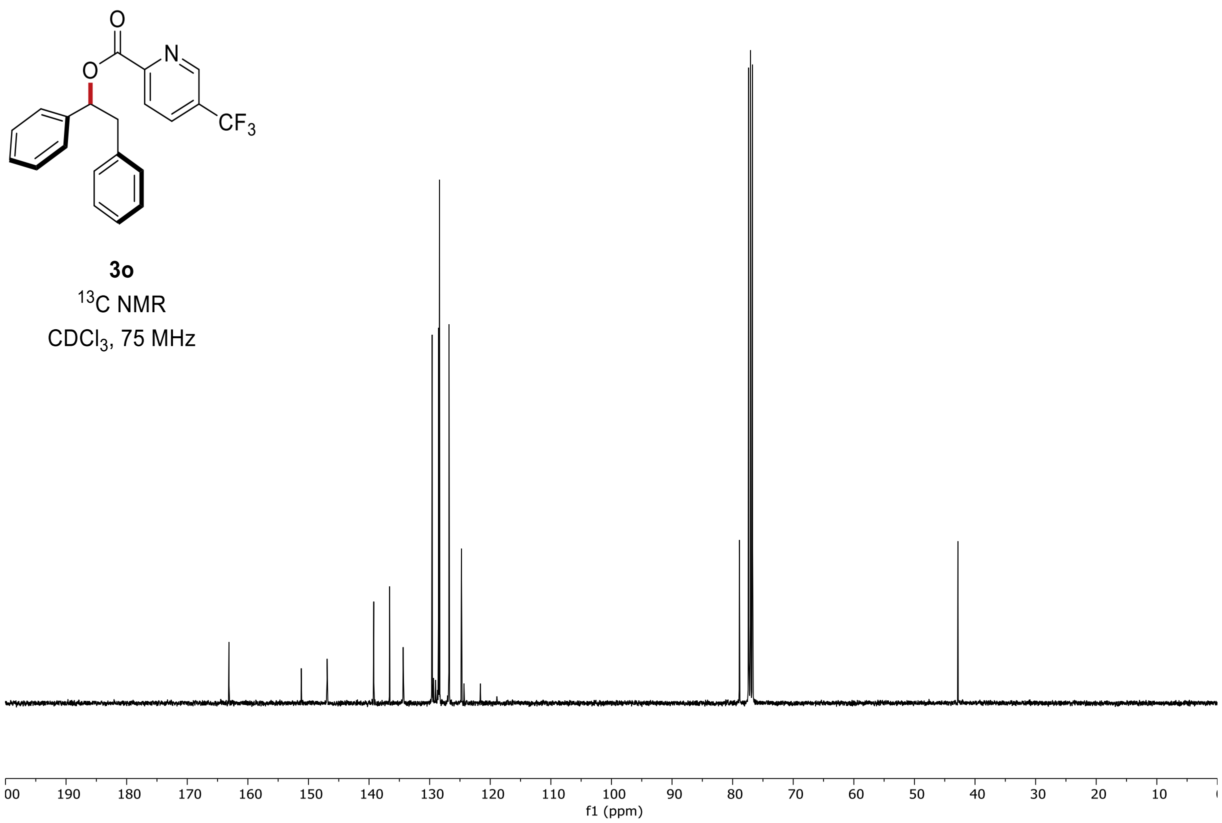

3o
13C NMR
CDCl3, 75 MHz
190
180
170
160
150
140
130
120
110
100
90
80
70
60
50
40
30
20
10
f1 (ppm)

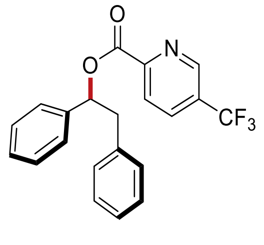

3o
^{19}F NMR
$CDCl_3$, 282 MHz

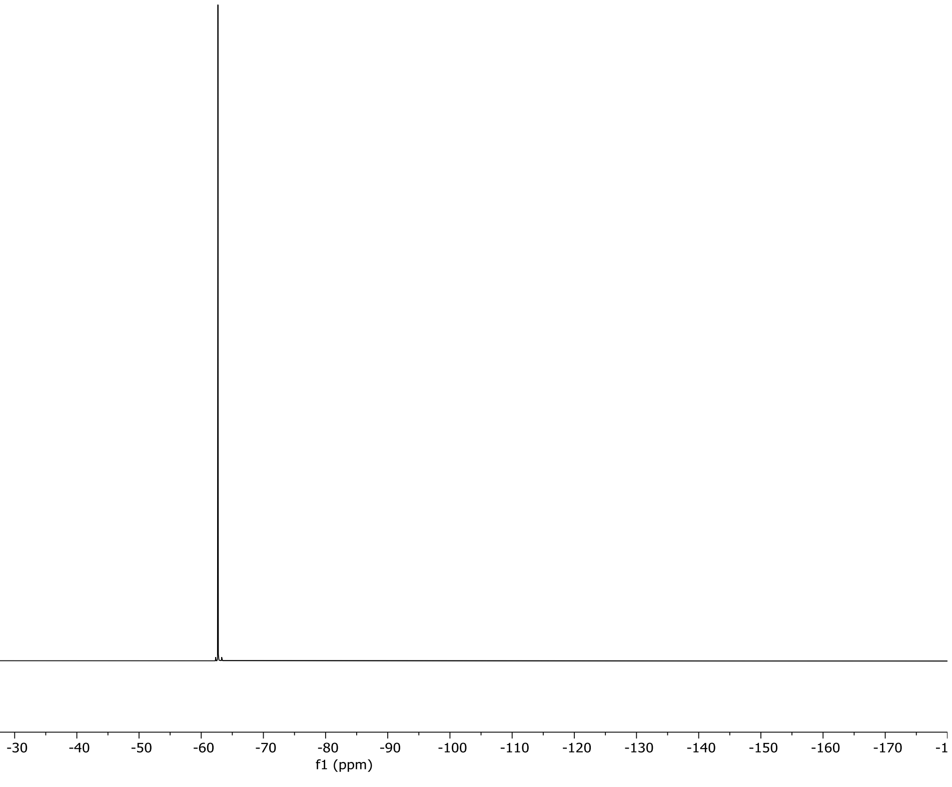

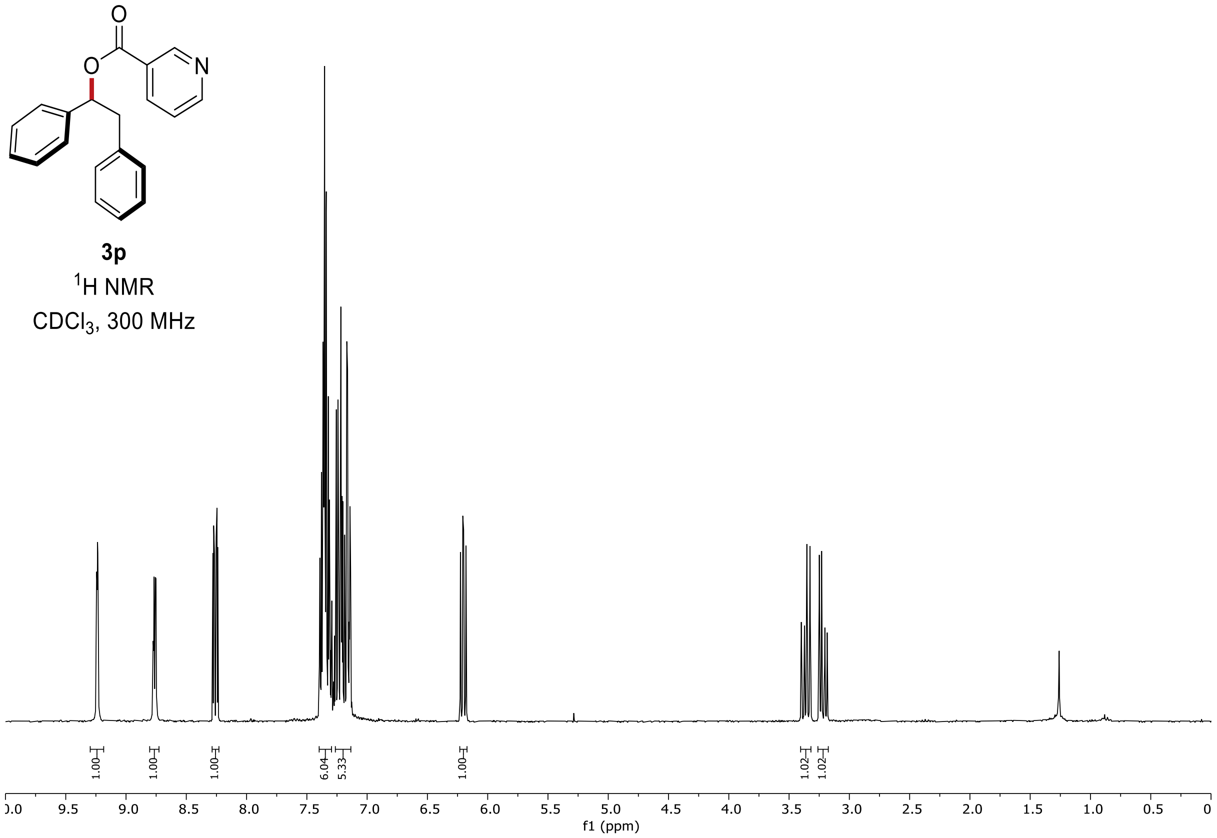

3p
^{1}H NMR
$CDCl_3$, 300 MHz

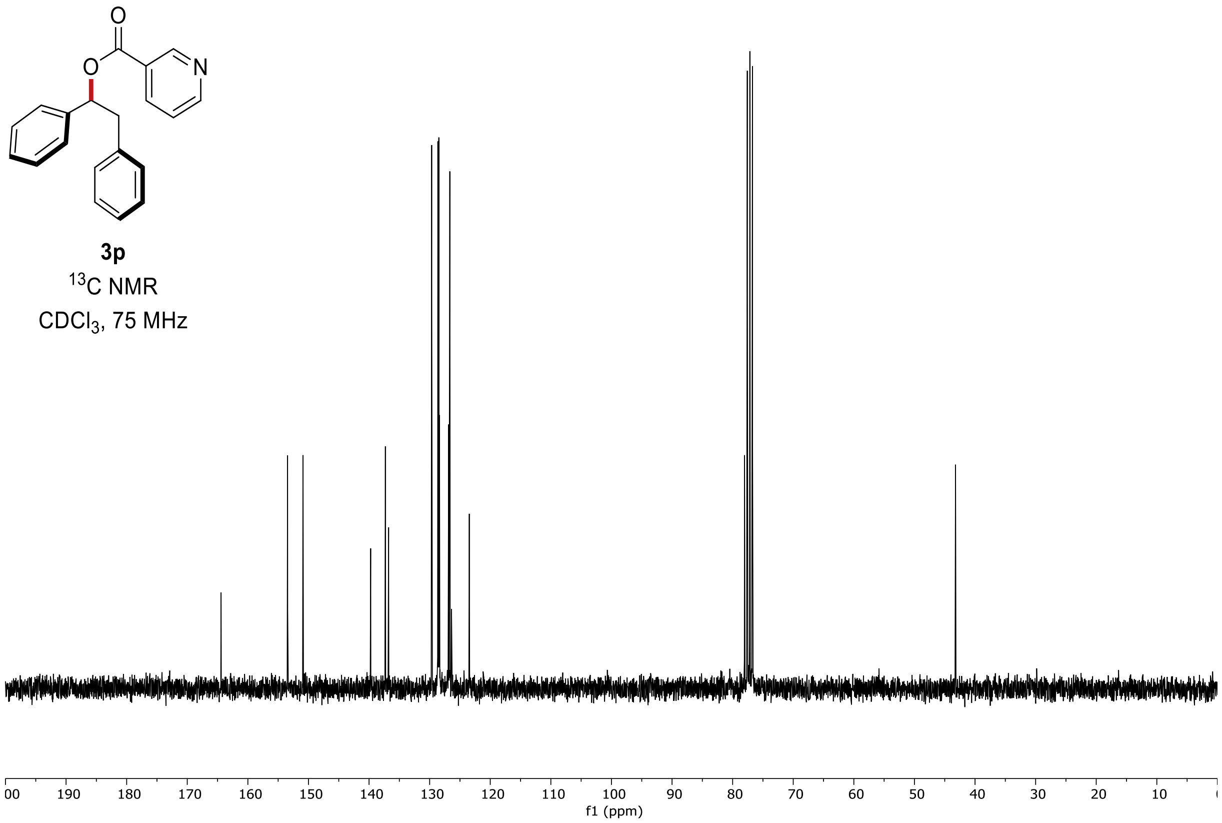

3p
^{13}C NMR
$CDCl_3$, 75 MHz

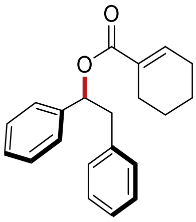

3q
^{1}H NMR
$CDCl_3$, 300 MHz

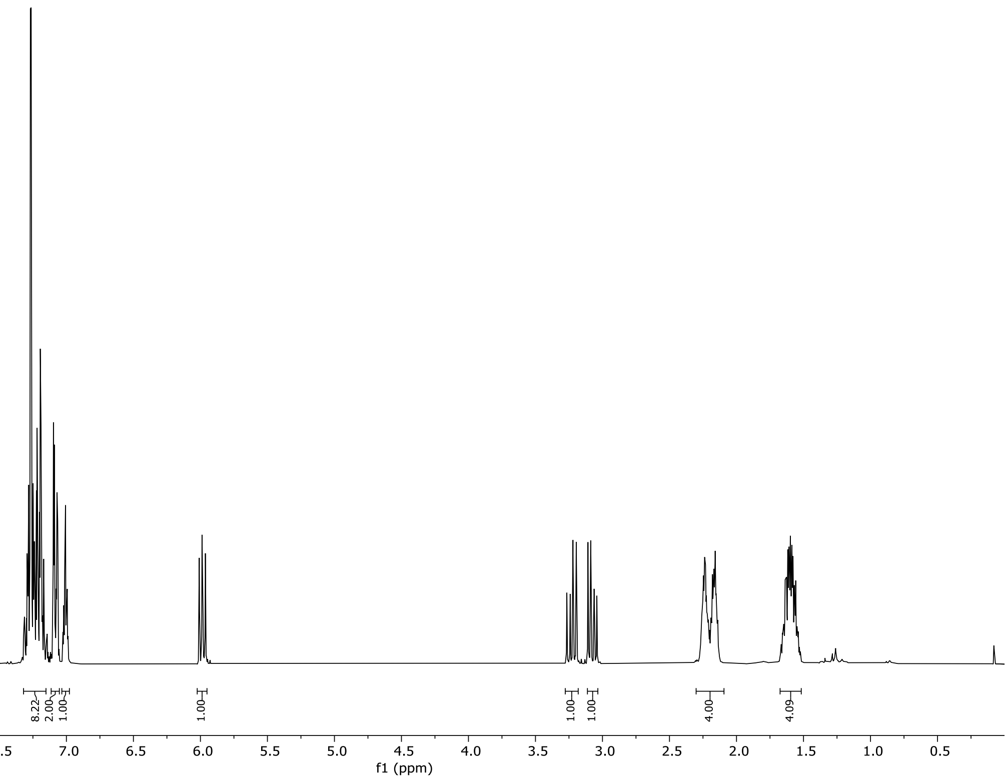

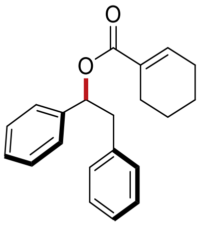

3q
^{13}C NMR
$CDCl_3$, 75 MHz

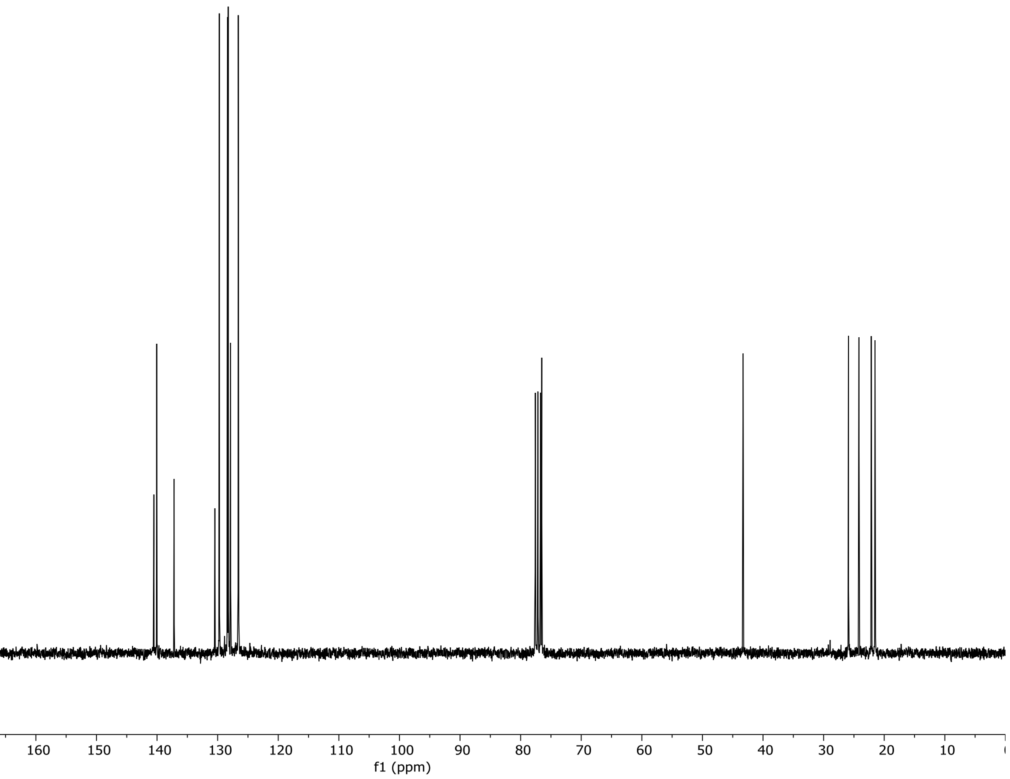

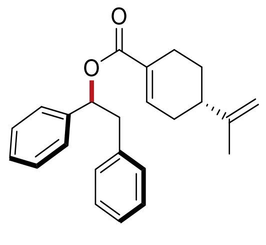

3r

^{1}H NMR

$CDCl_3$, 400 MHz

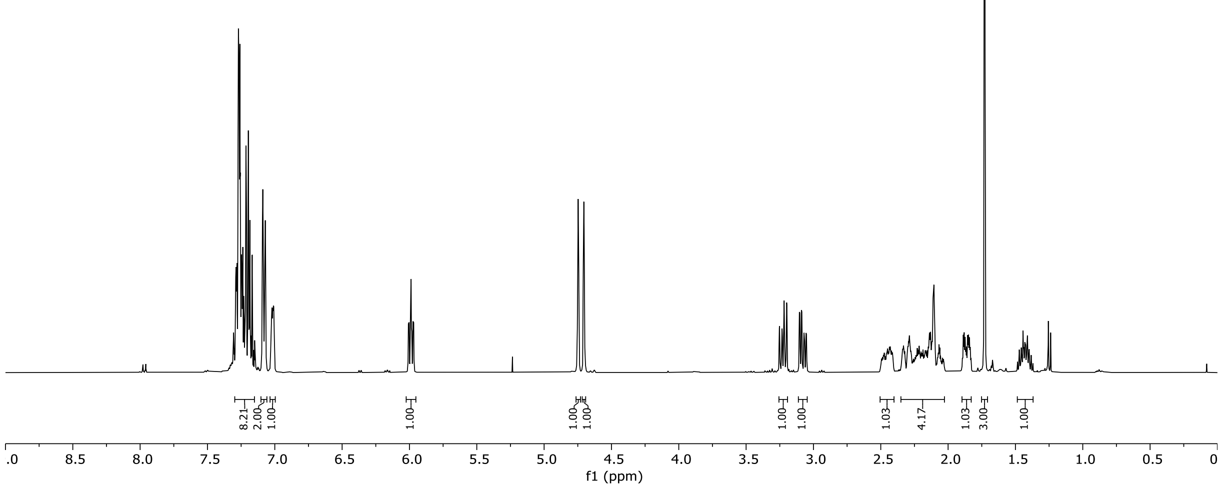

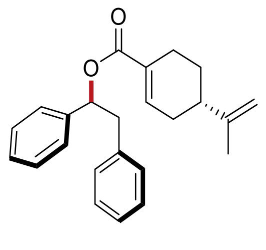

3r

^{13}C NMR

$CDCl_3$, 100 MHz

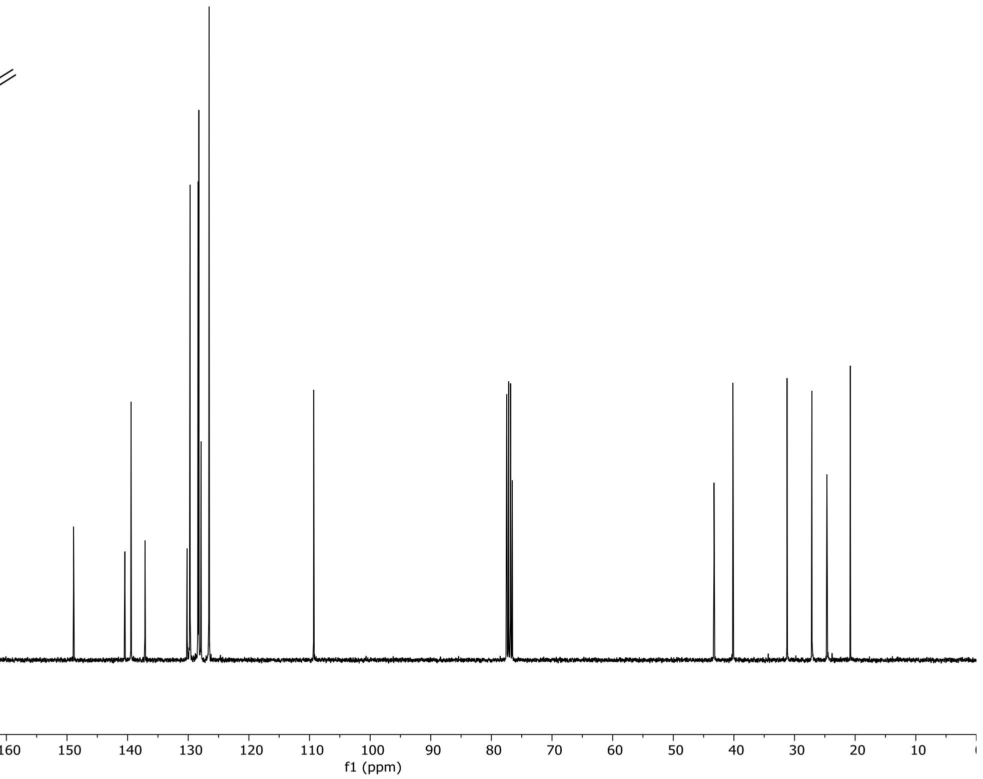

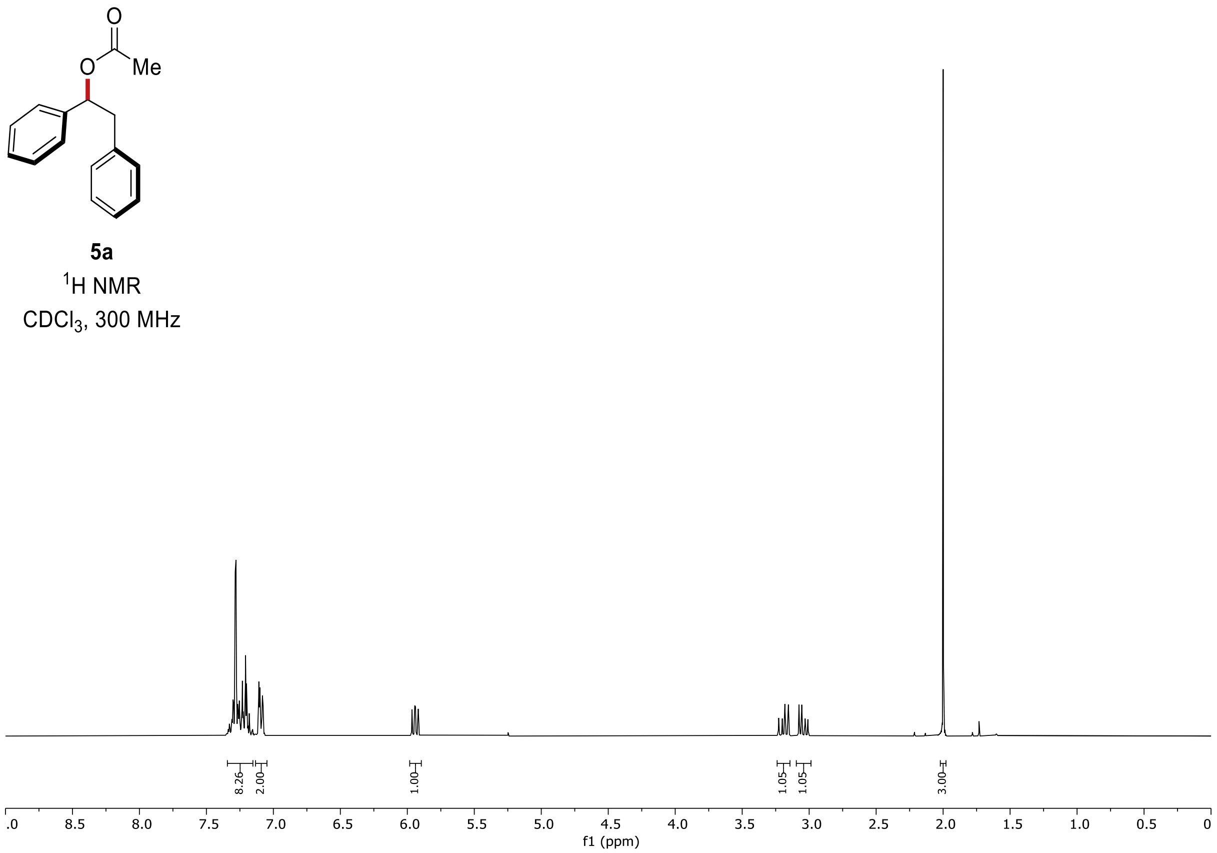
O
Me
5a
1H NMR
CDCl3, 300 MHz
8.26
2.00
1.00
1.05
1.05
3.00
f1 (ppm)

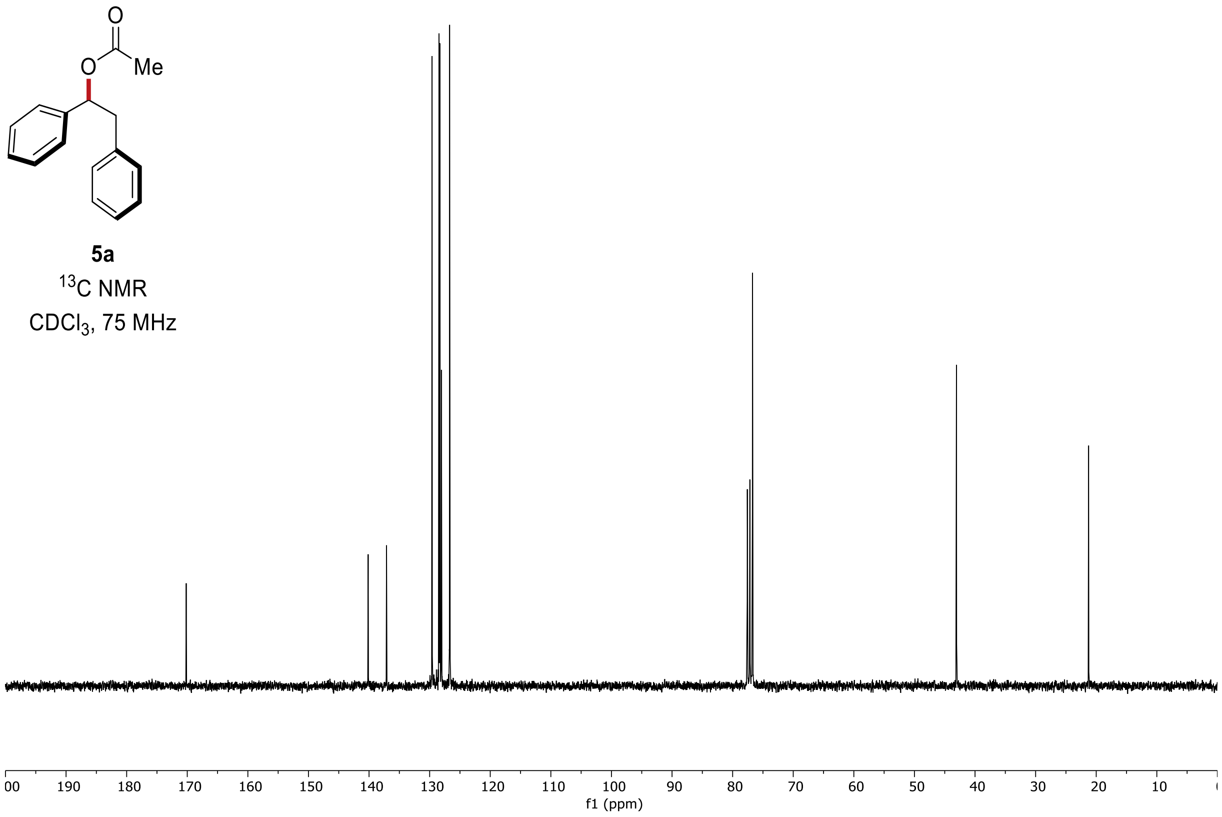
O
Me
5a
13C NMR
CDCl3, 75 MHz
f1 (ppm)

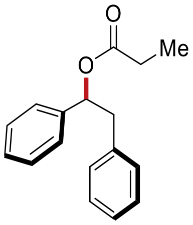

5b
^{1}H NMR
$CDCl_3$, 400 MHz

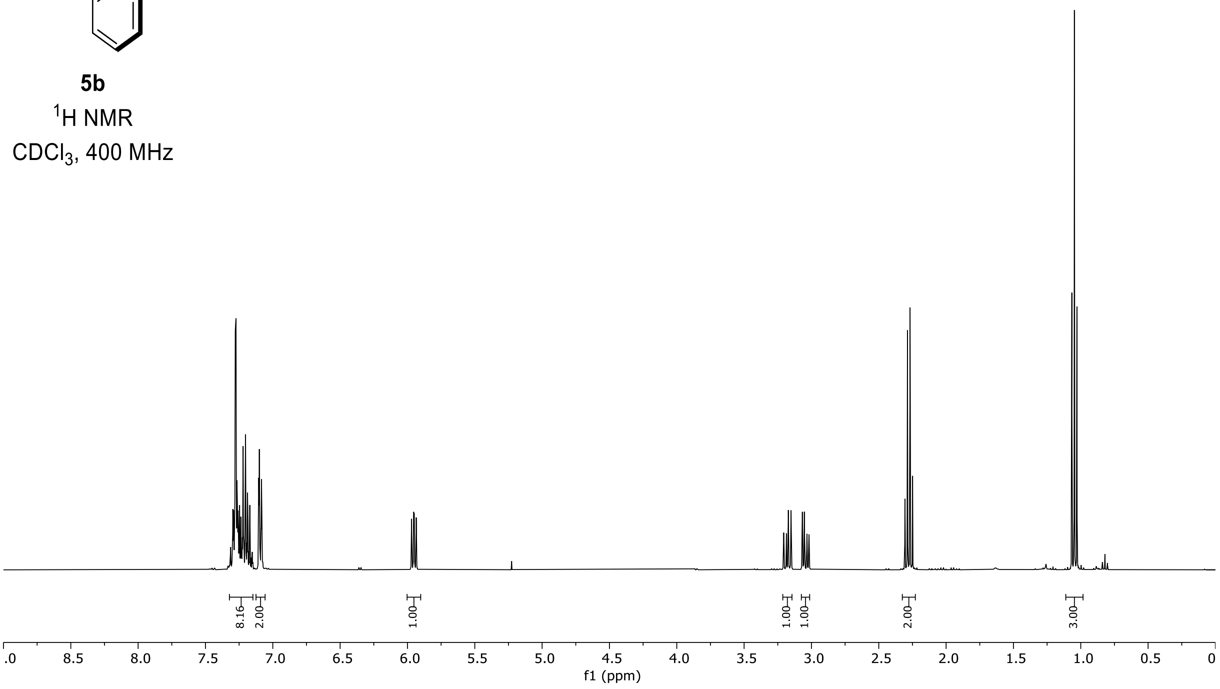

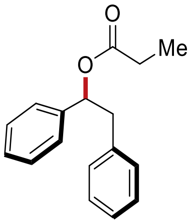

5b
^{13}C NMR
$CDCl_3$, 100 MHz

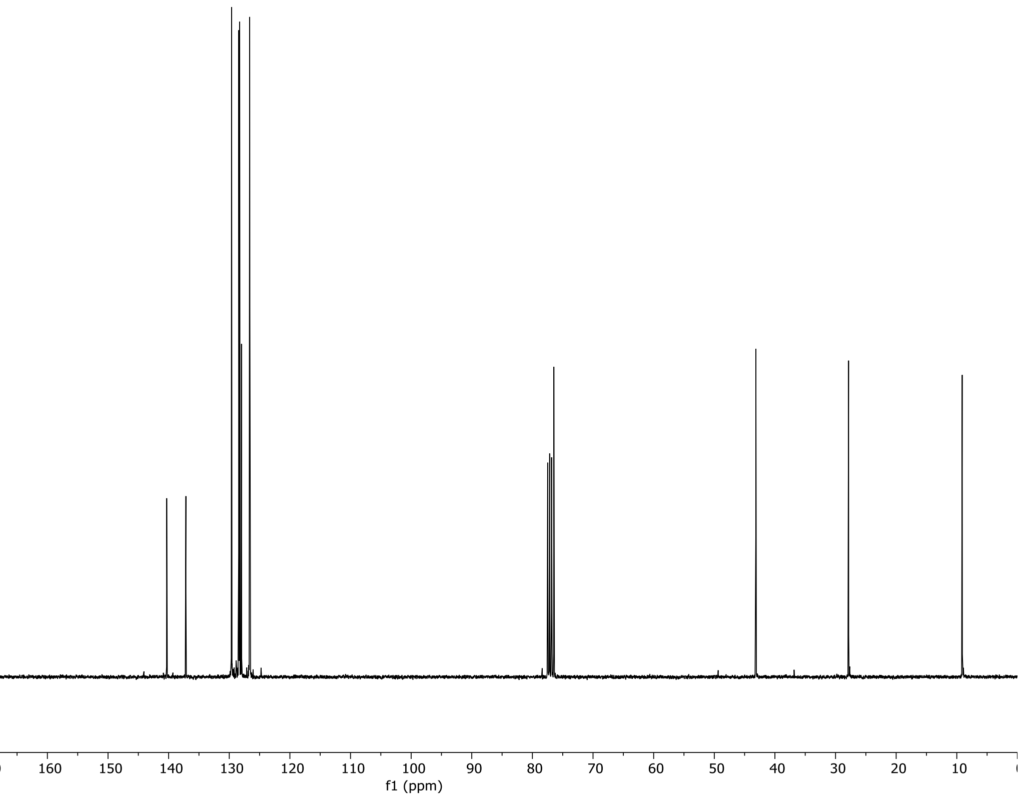

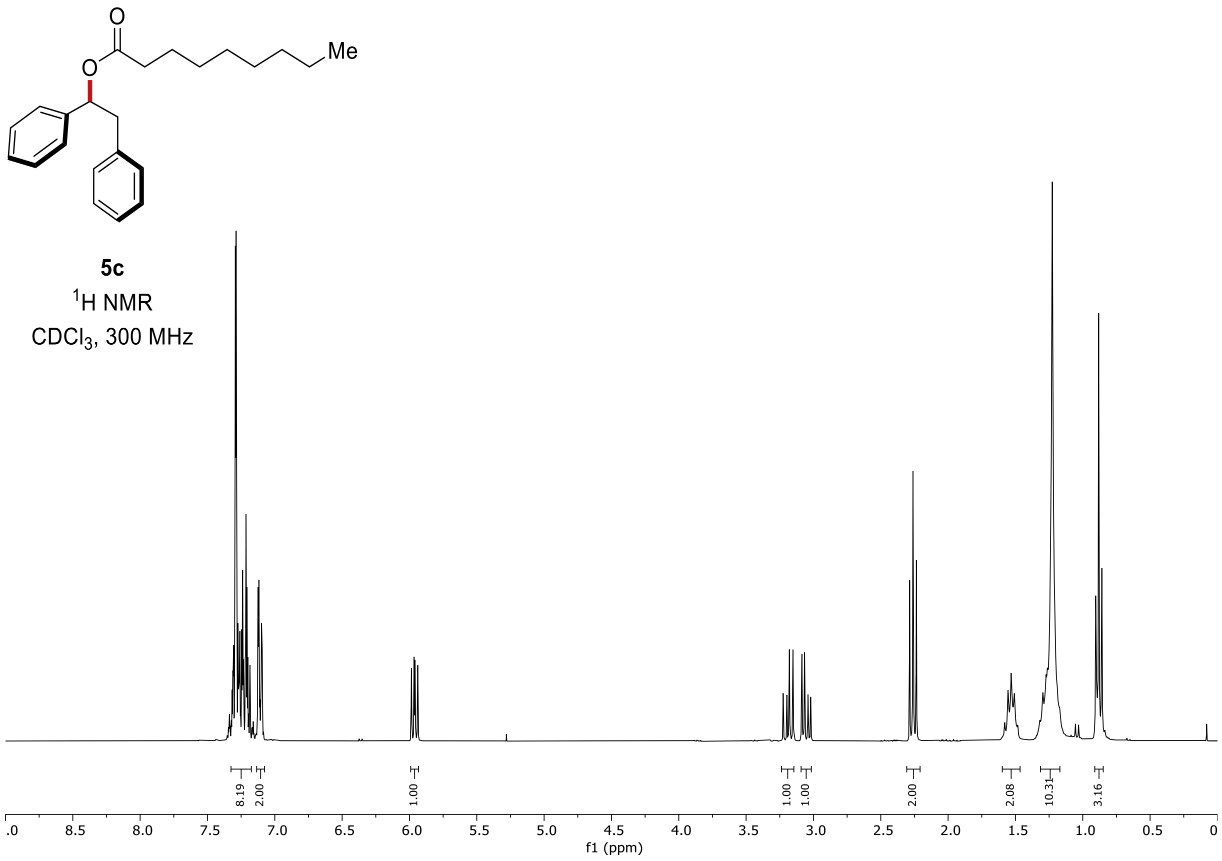

Me
O
O
5c
^{1}H NMR
CDCl3, 300 MHz
8.19
2.00
1.00
1.00
1.00
2.00
2.08
10.31
3.16
f1 (ppm)

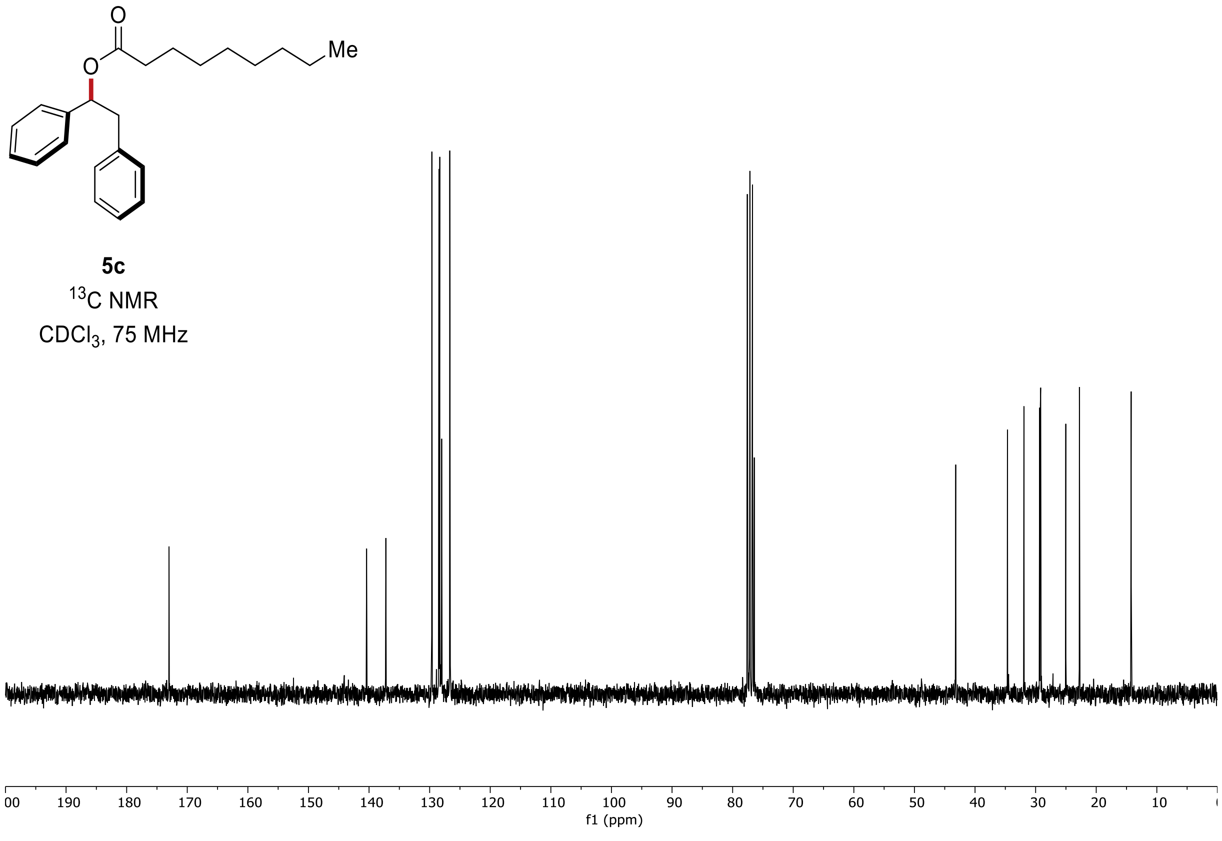

Me
O
O
5c
^{13}C NMR
CDCl3, 75 MHz
f1 (ppm)

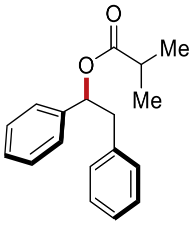

5d
^{1}H NMR
$CDCl_3$, 400 MHz

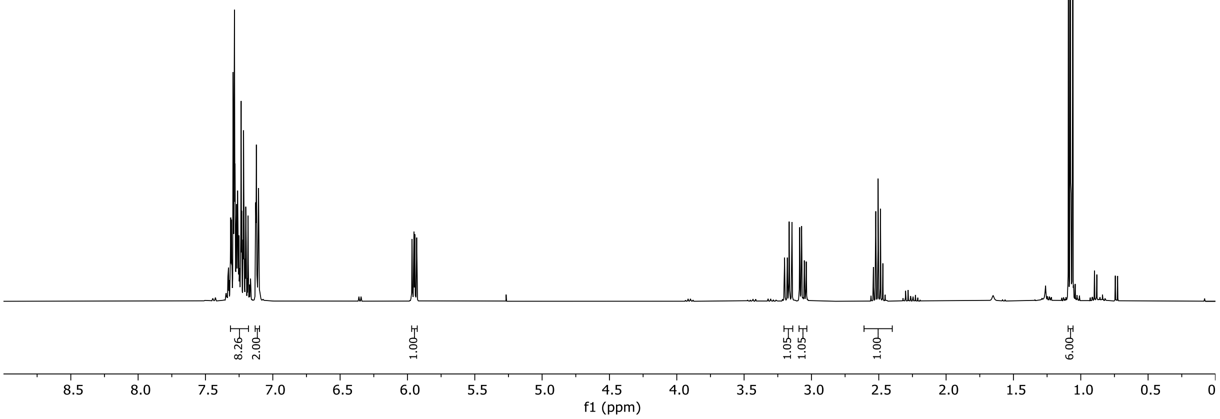

5d
^{13}C NMR
$CDCl_3$, 100 MHz

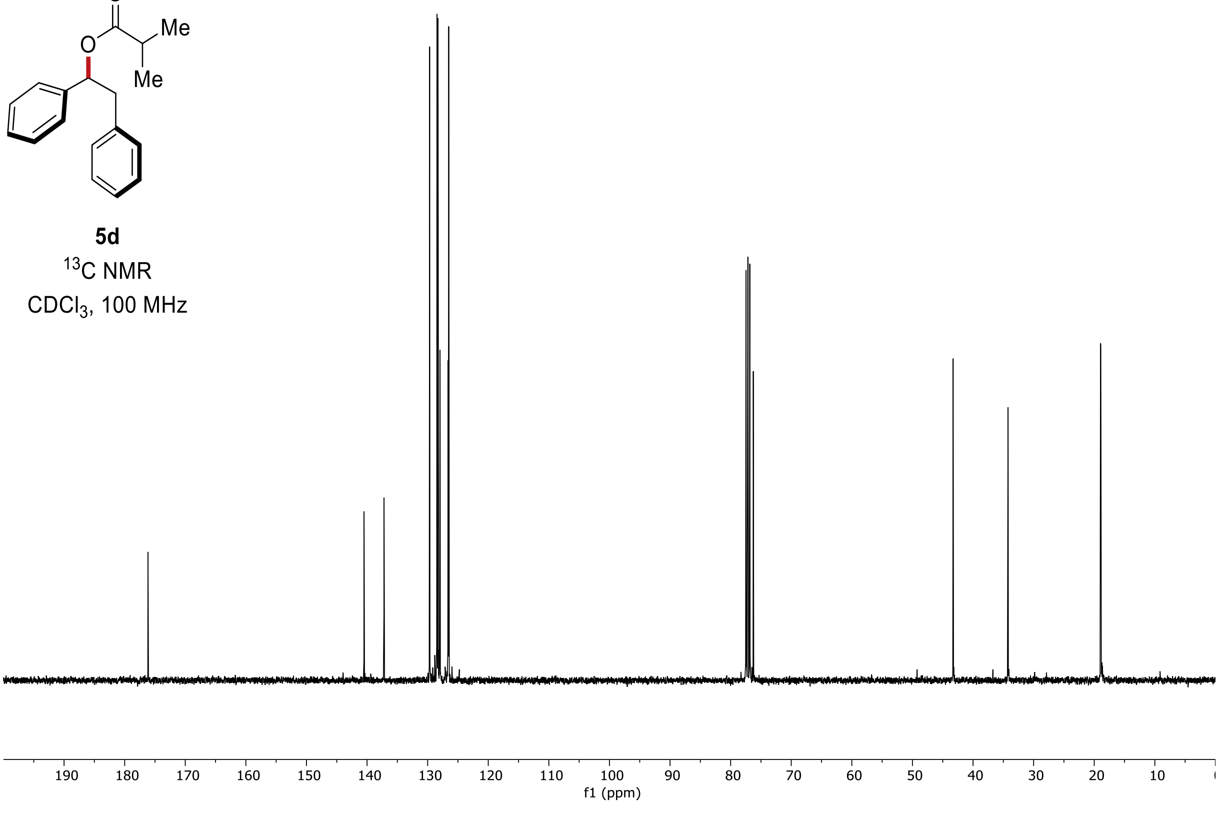

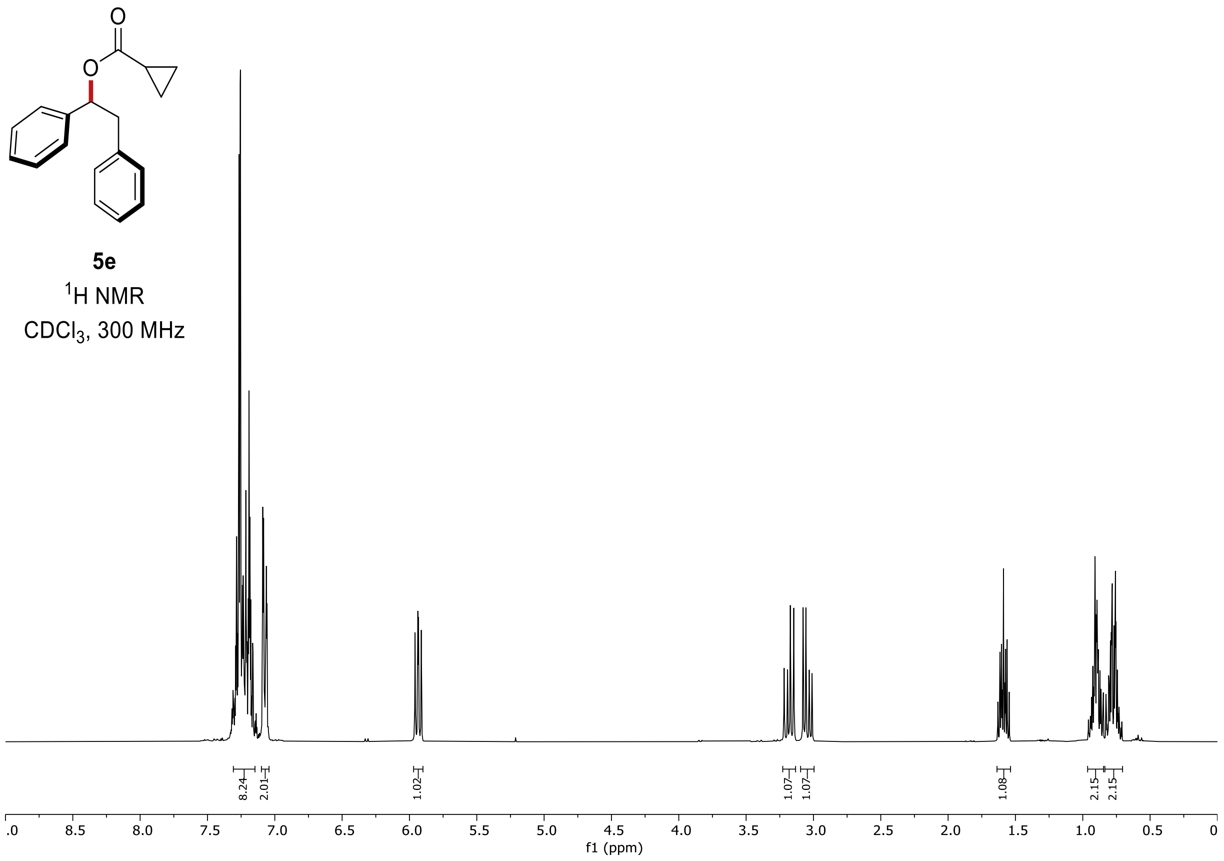
5e
^{1}H NMR
$CDCl_3$, 300 MHz
8.24
2.01
1.02
1.07
1.07
1.08
2.15
2.15
f1 (ppm)

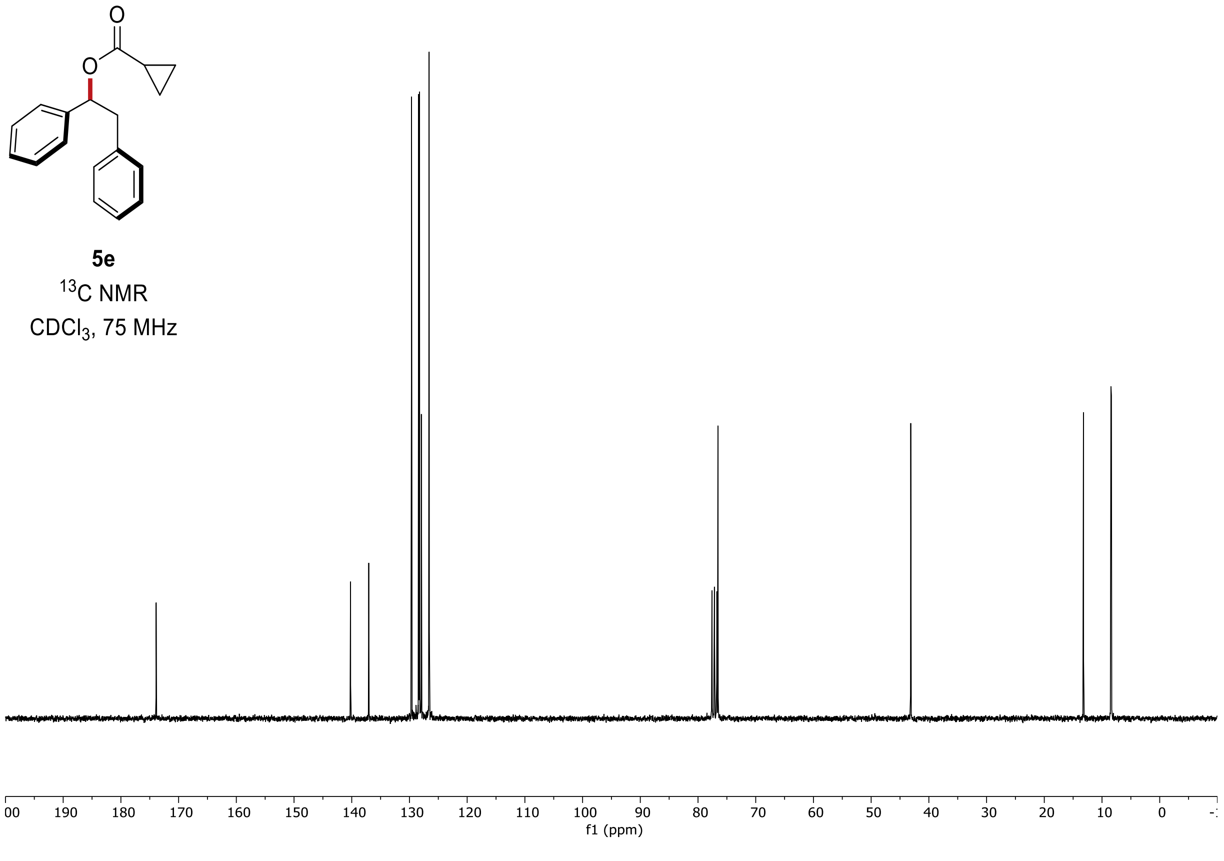
5e
^{13}C NMR
$CDCl_3$, 75 MHz
f1 (ppm)

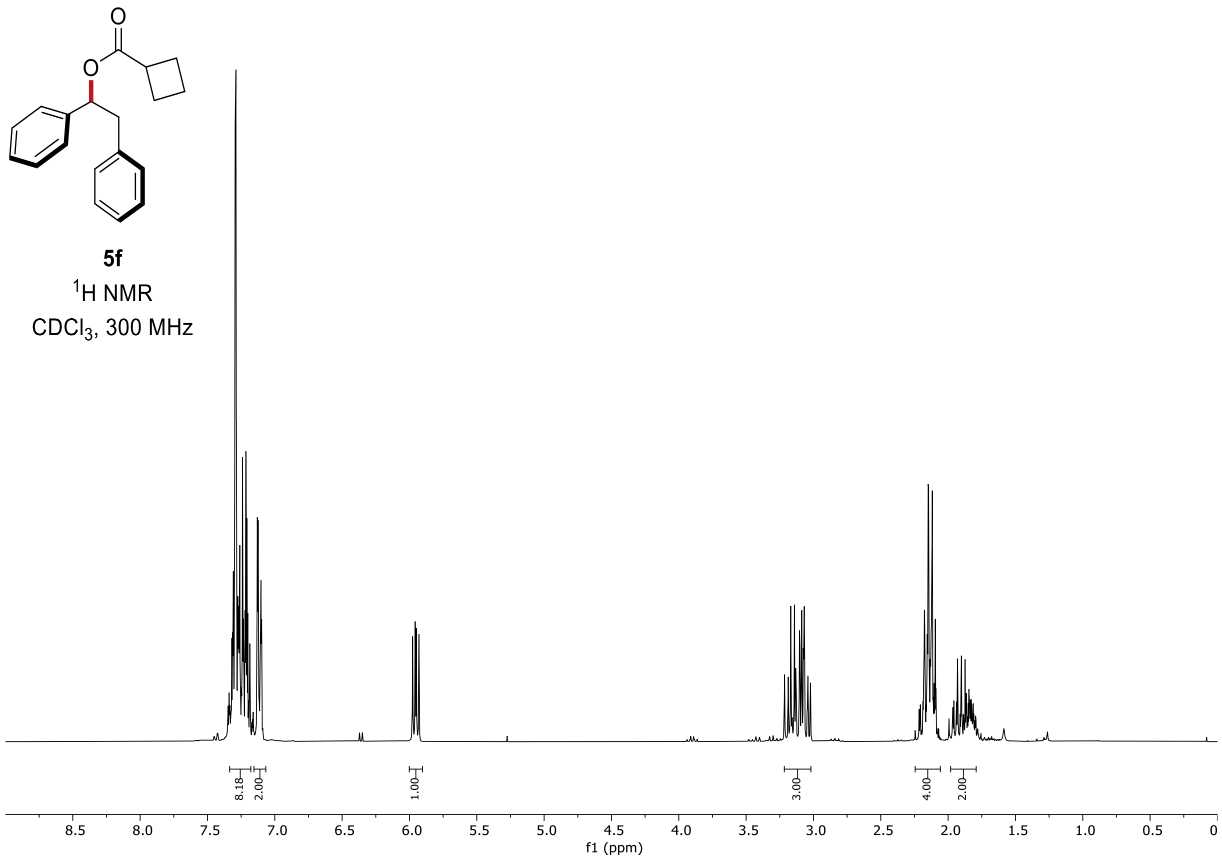
O
O
5f
1H NMR
CDCl3, 300 MHz
8.18
2.00
1.00
3.00
4.00
2.00
8.5 8.0 7.5 7.0 6.5 6.0 5.5 5.0 4.5 4.0 3.5 3.0 2.5 2.0 1.5 1.0 0.5 0
f1 (ppm)

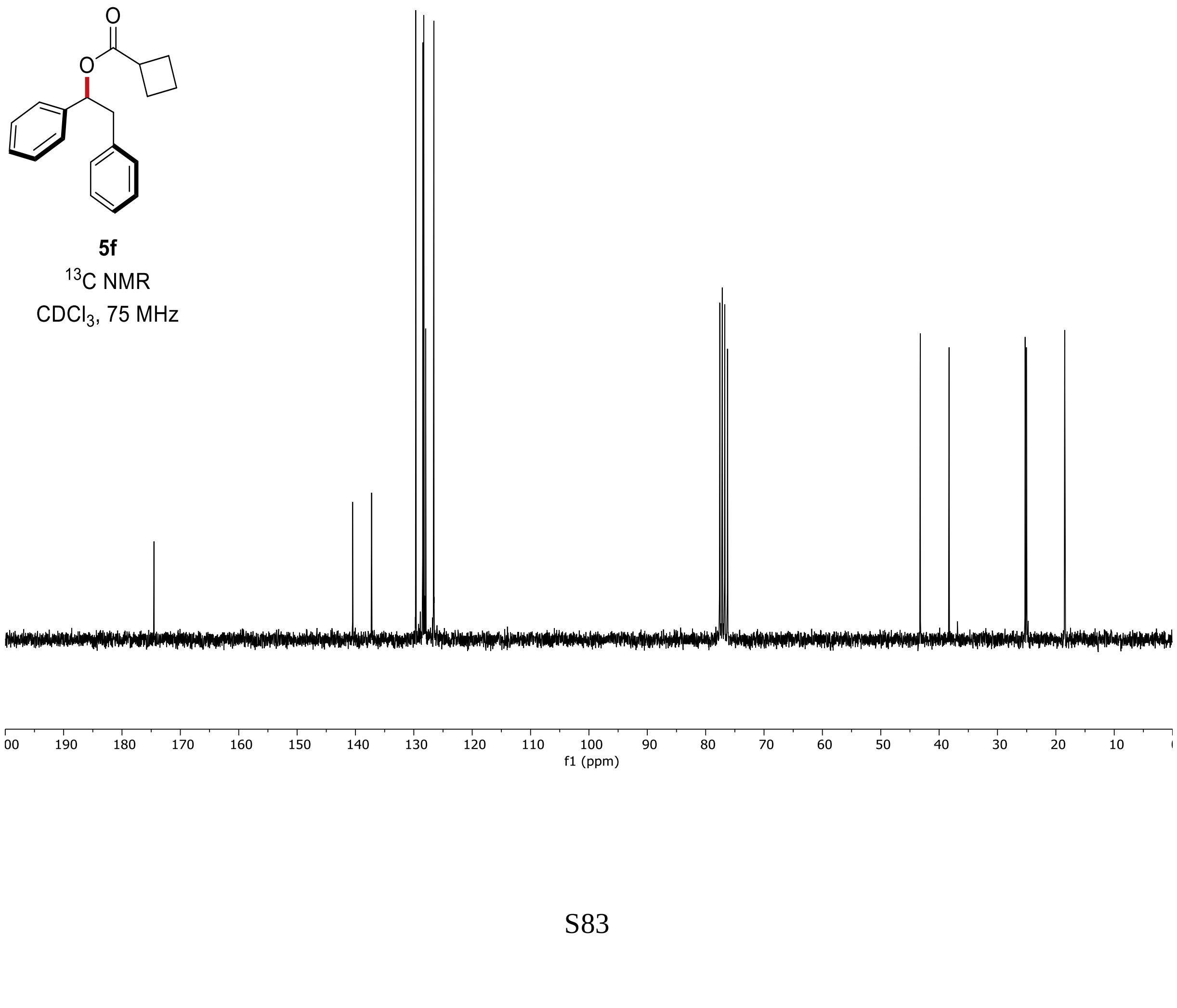
O
O
5f
13C NMR
CDCl3, 75 MHz
00 190 180 170 160 150 140 130 120 110 100 90 80 70 60 50 40 30 20 10
f1 (ppm)

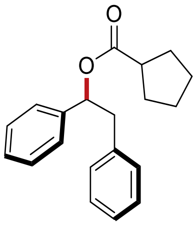

5g

^{1}H NMR

$CDCl_3$, 300 MHz

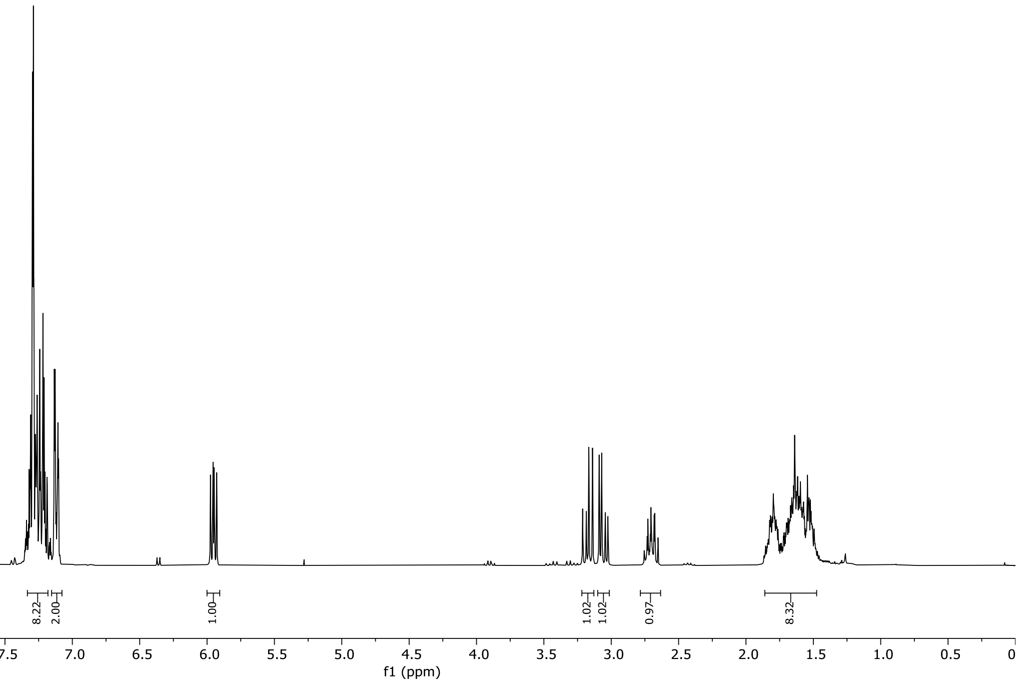

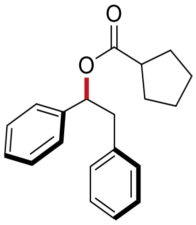

5g

^{13}C NMR

$CDCl_3$, 75 MHz

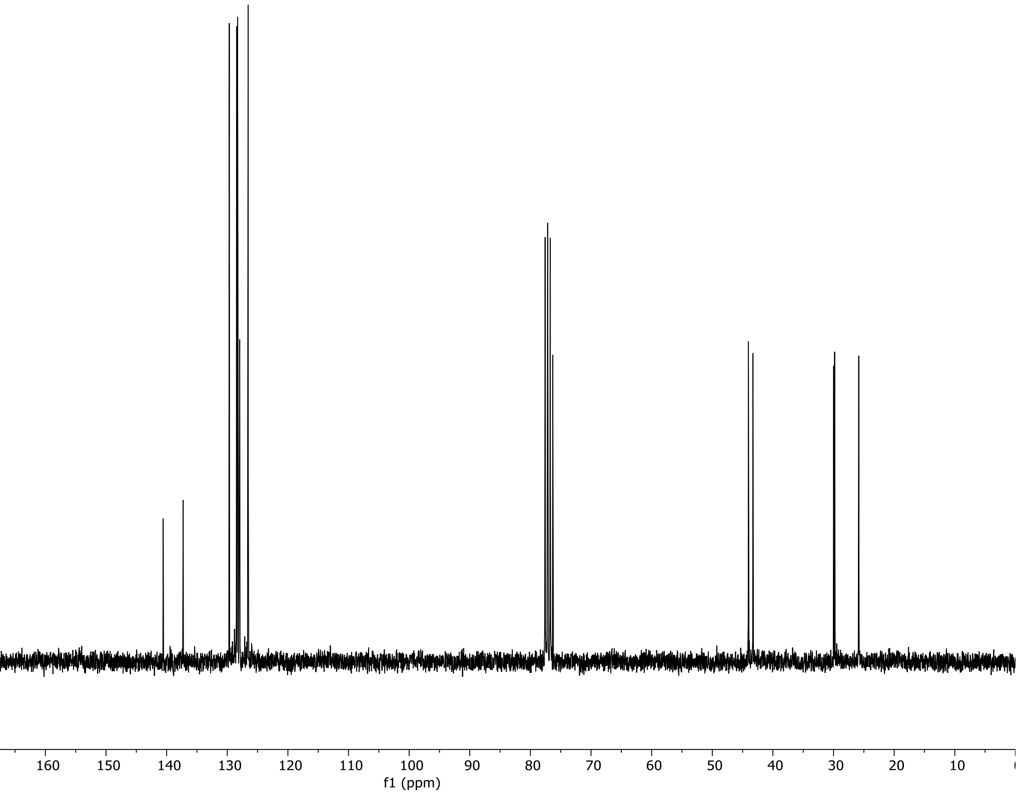

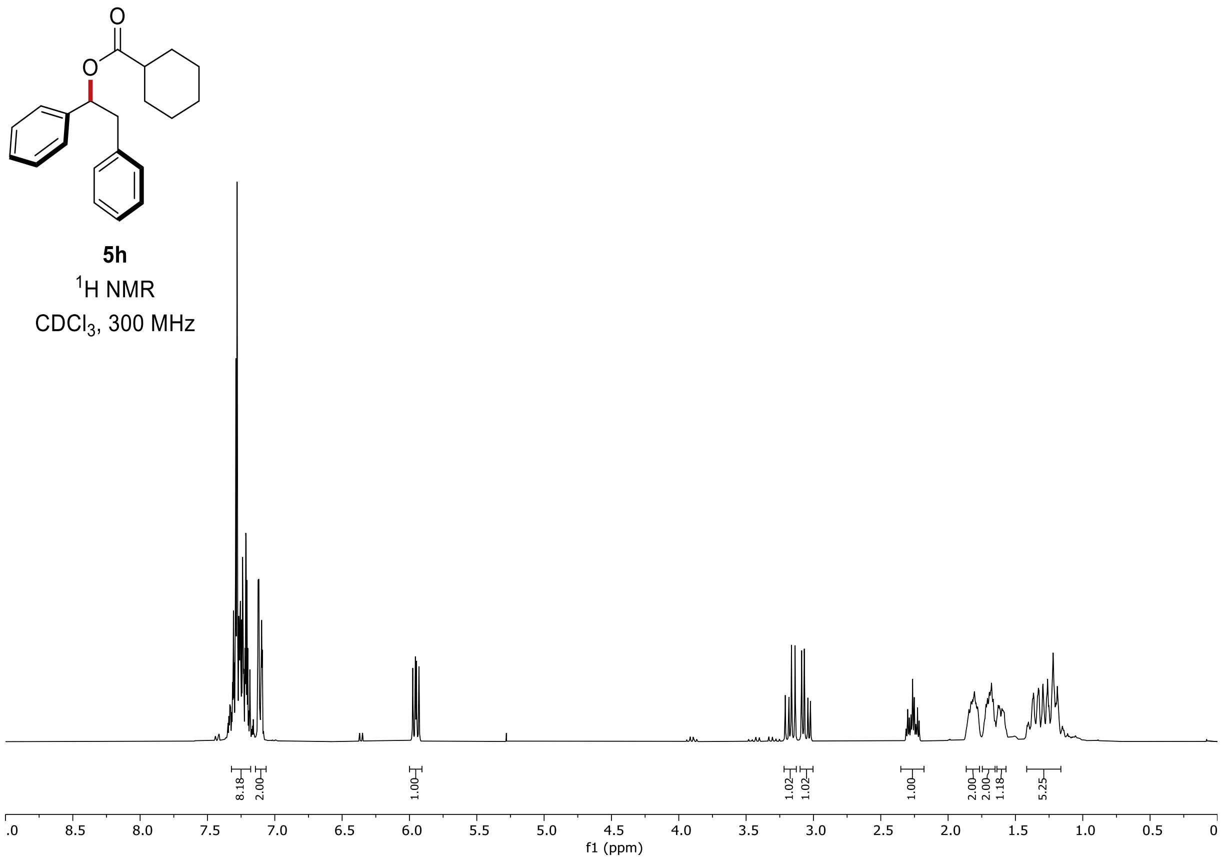
5h
1H NMR
CDCl3, 300 MHz
8.18
2.00
1.00
1.02
1.02
1.00
2.00
2.00
1.18
5.25
f1 (ppm)

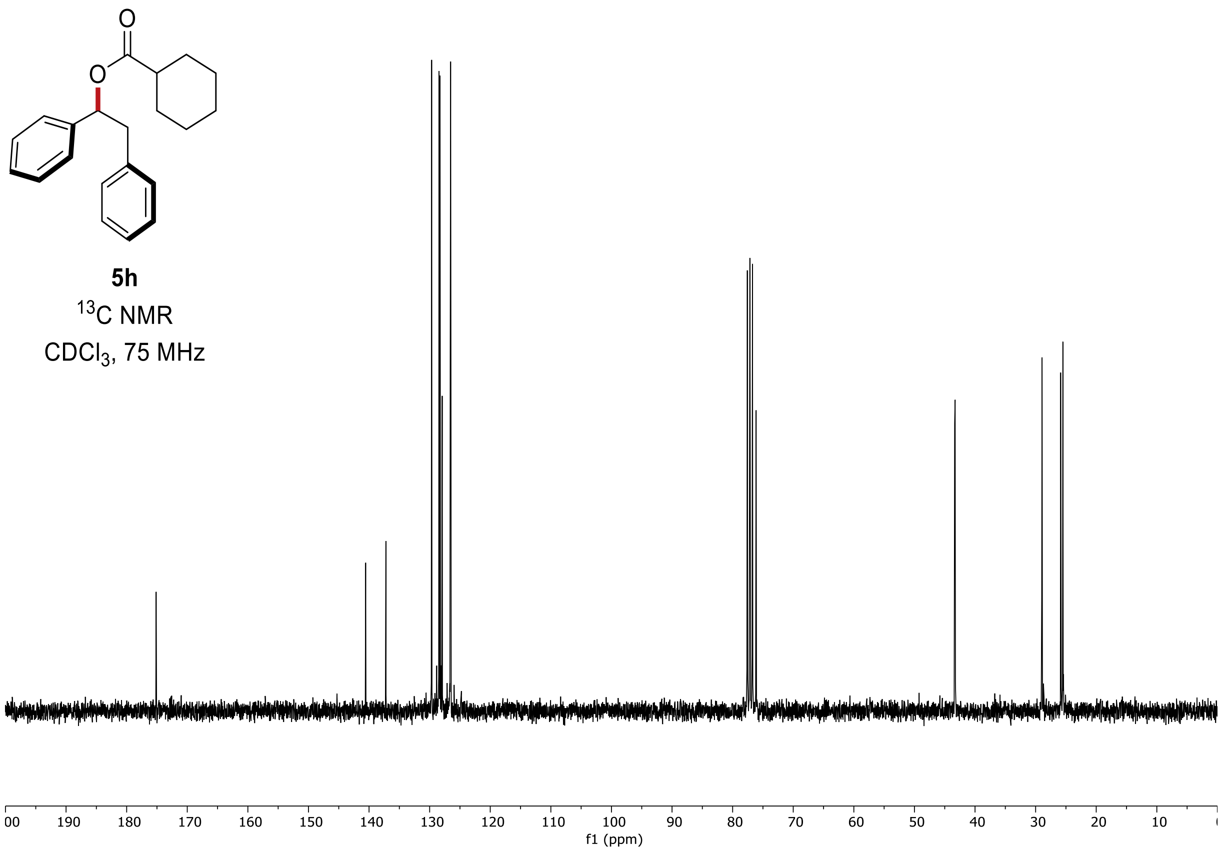
5h
13C NMR
CDCl3, 75 MHz
f1 (ppm)

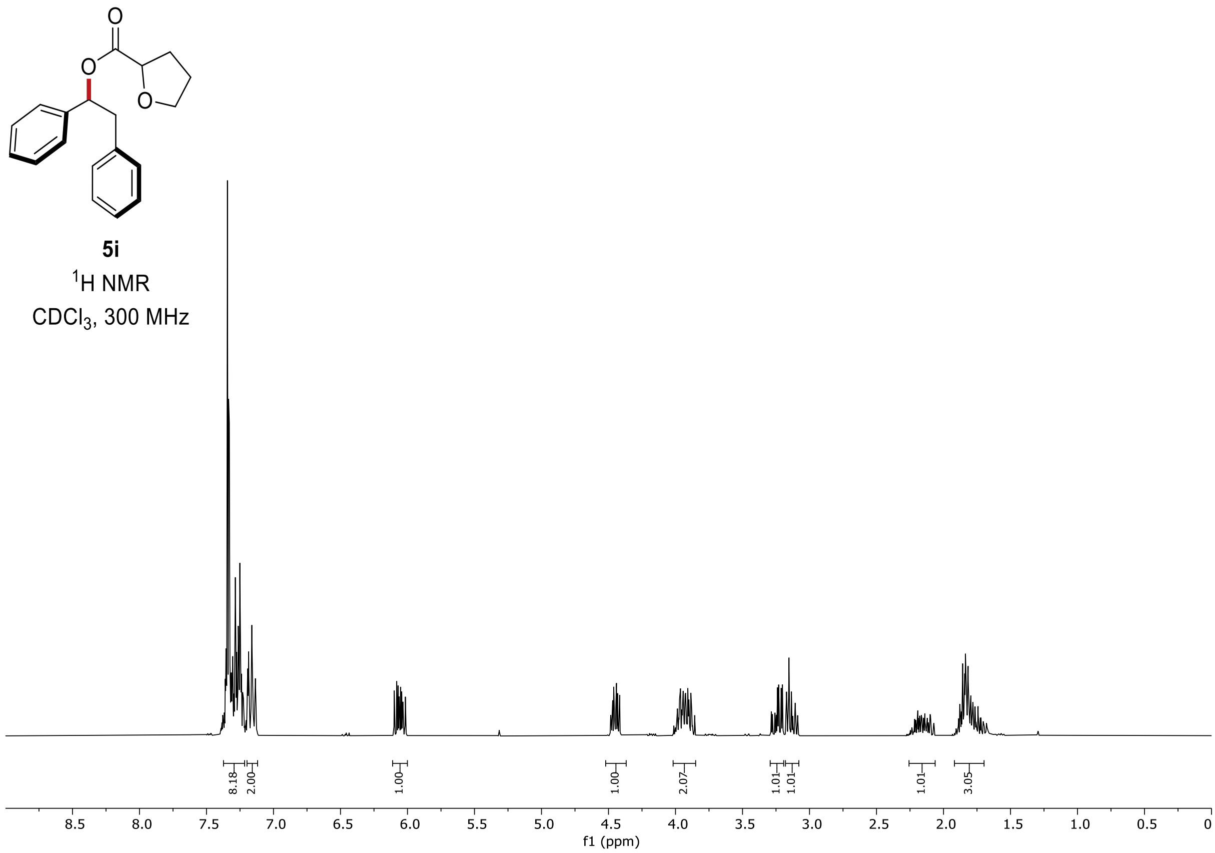

5i
^{1}H NMR
$CDCl_3$, 300 MHz

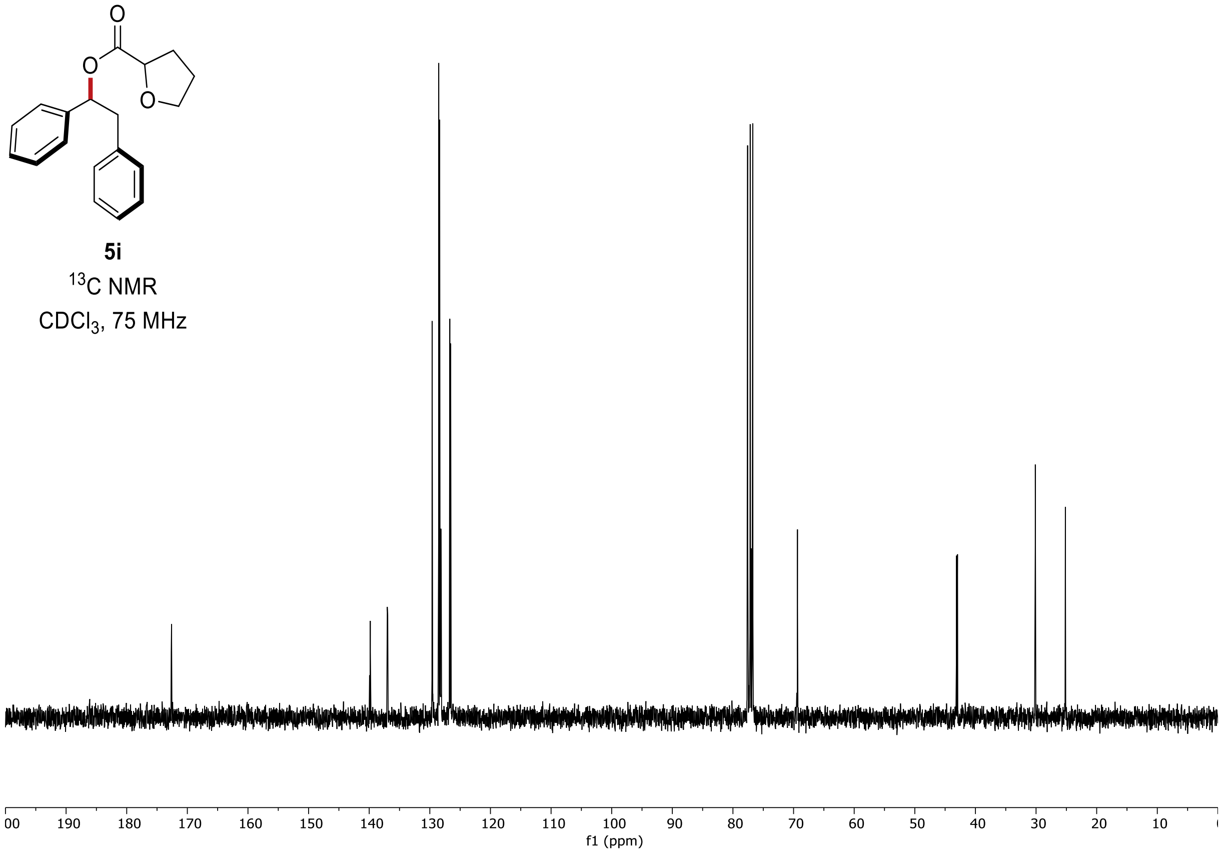

5i
^{13}C NMR
$CDCl_3$, 75 MHz

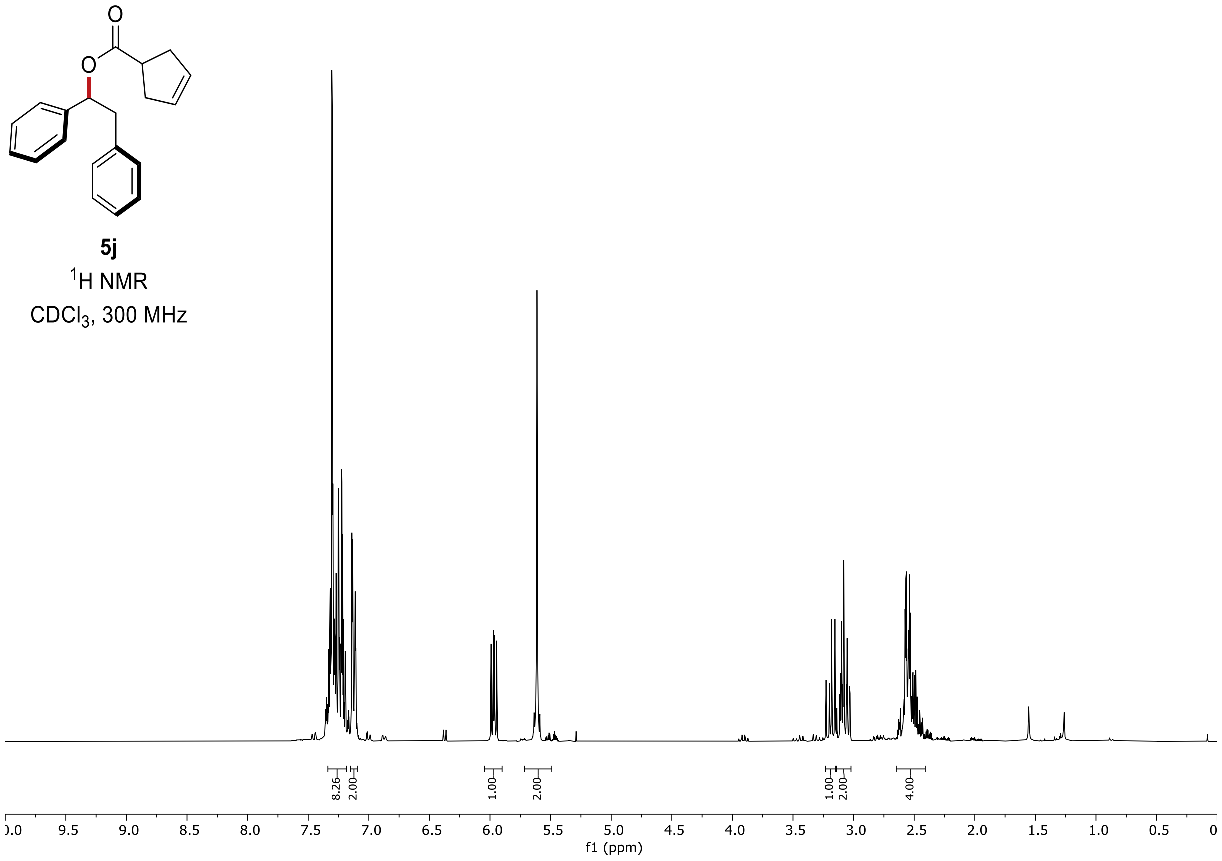

O
O
5j
^{1}H NMR
CDCl$_3$, 300 MHz
8.26
2.00
1.00
2.00
1.00
2.00
4.00
f1 (ppm)

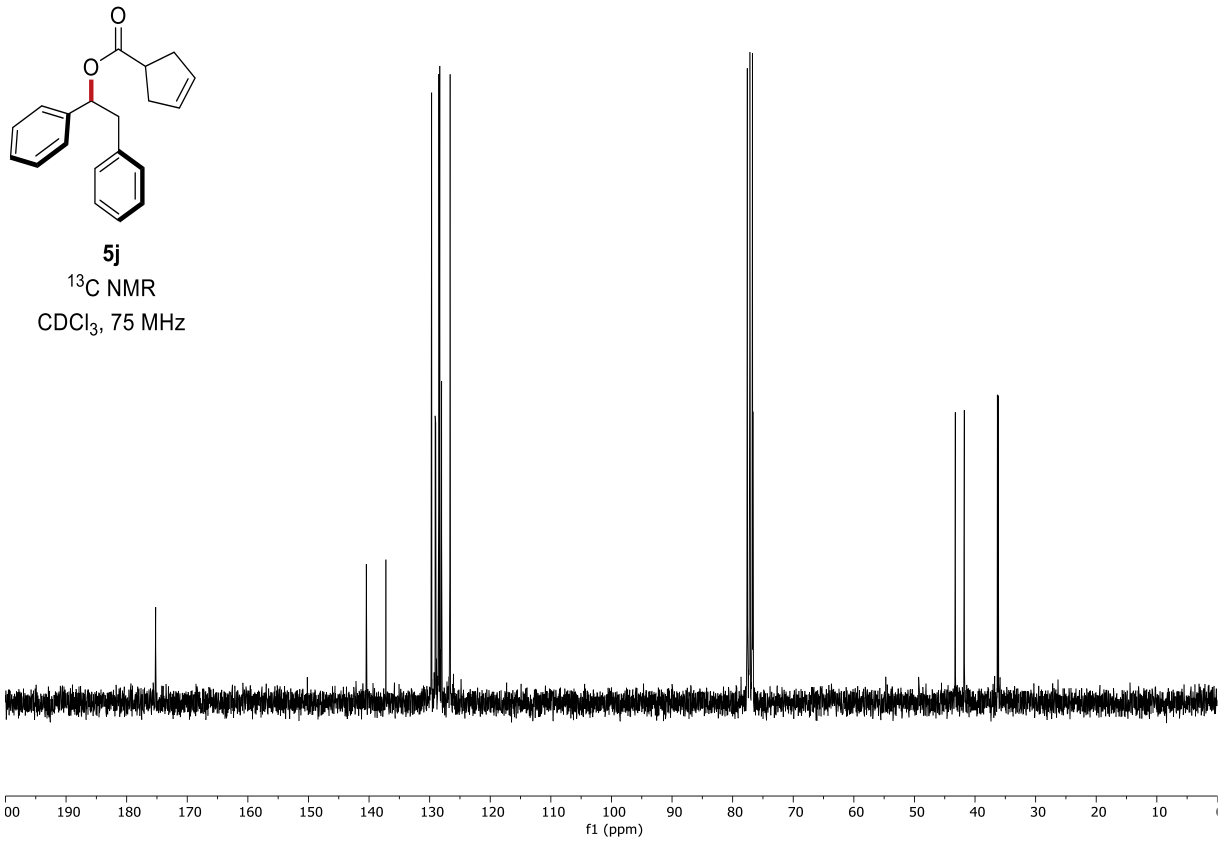

O
O
5j
^{13}C NMR
CDCl$_3$, 75 MHz
f1 (ppm)

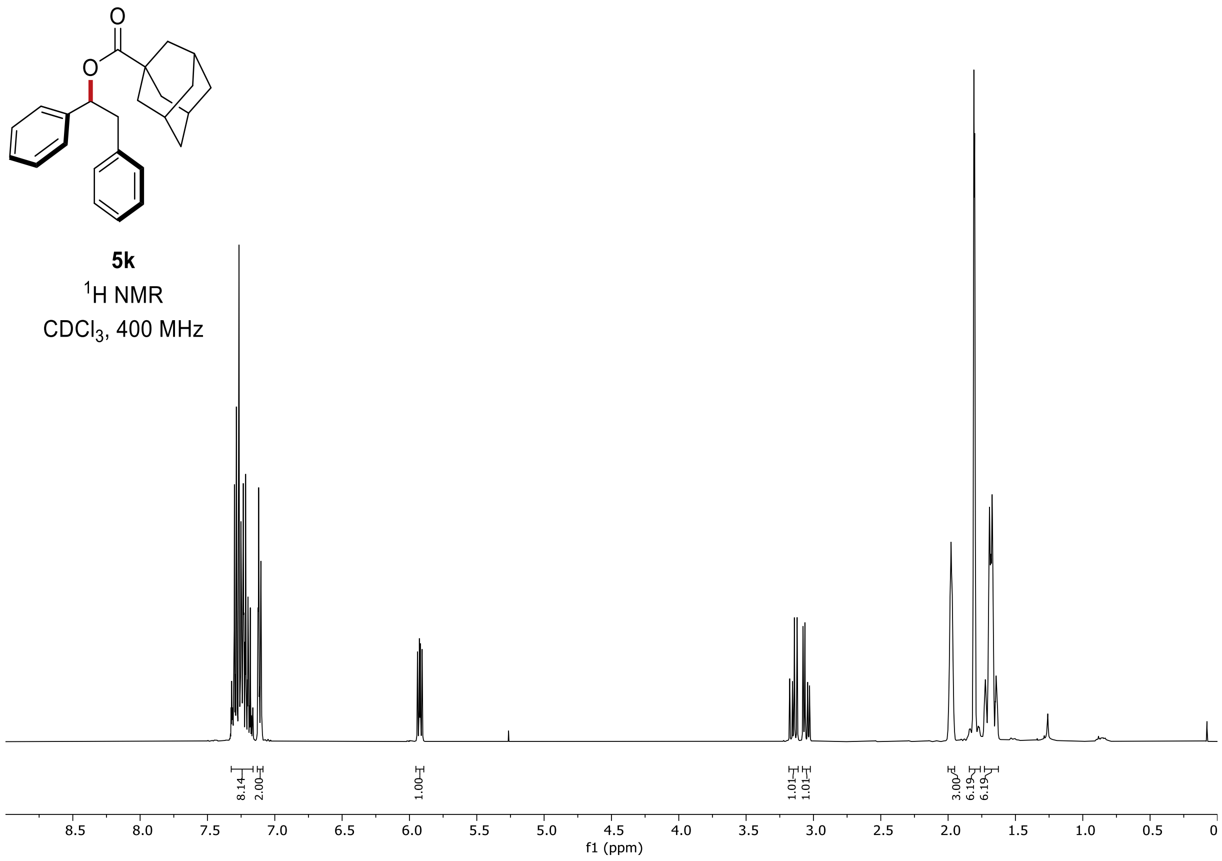

5k
^{1}H NMR
CDCl$_3$, 400 MHz
8.14
2.00
1.00
1.01
1.01
3.00
6.19
6.19
8.5 8.0 7.5 7.0 6.5 6.0 5.5 5.0 4.5 4.0 3.5 3.0 2.5 2.0 1.5 1.0 0.5 0
f1 (ppm)

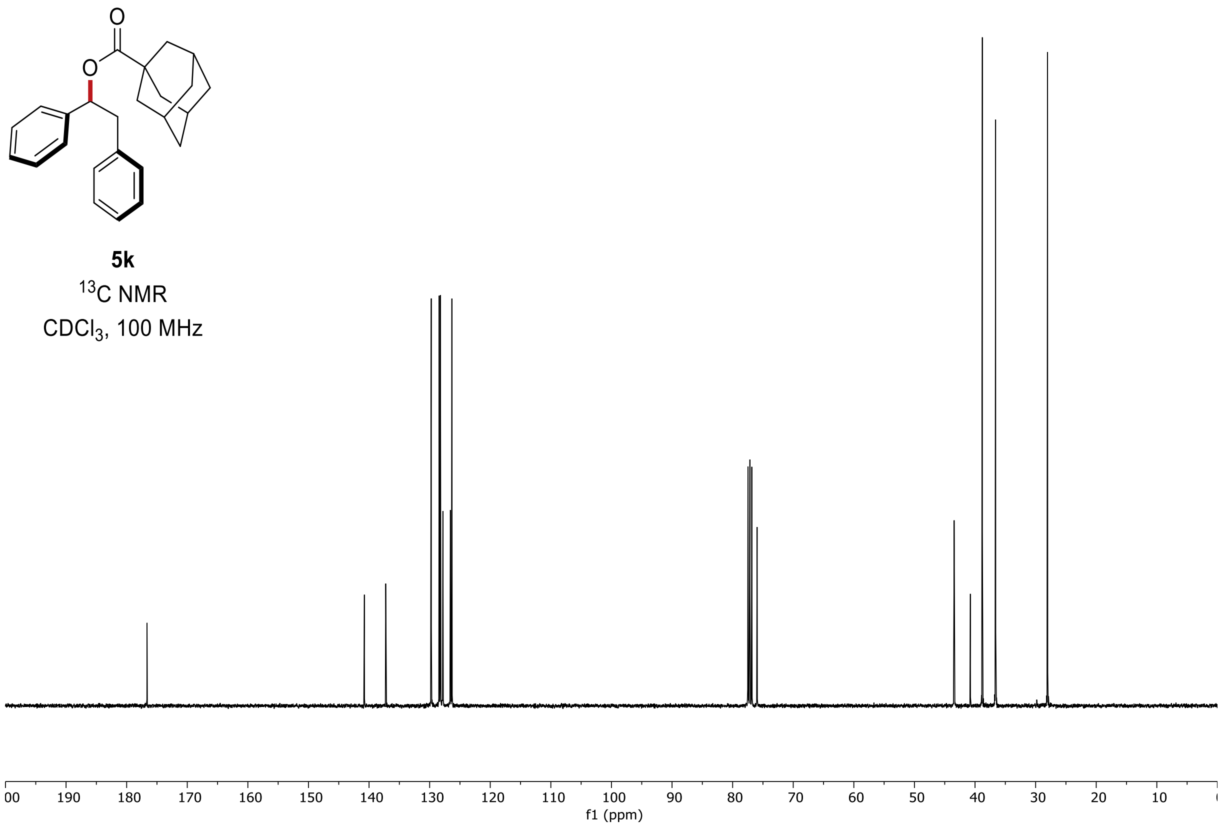

5k
^{13}C NMR
CDCl$_3$, 100 MHz
00 190 180 170 160 150 140 130 120 110 100 90 80 70 60 50 40 30 20 10
f1 (ppm)

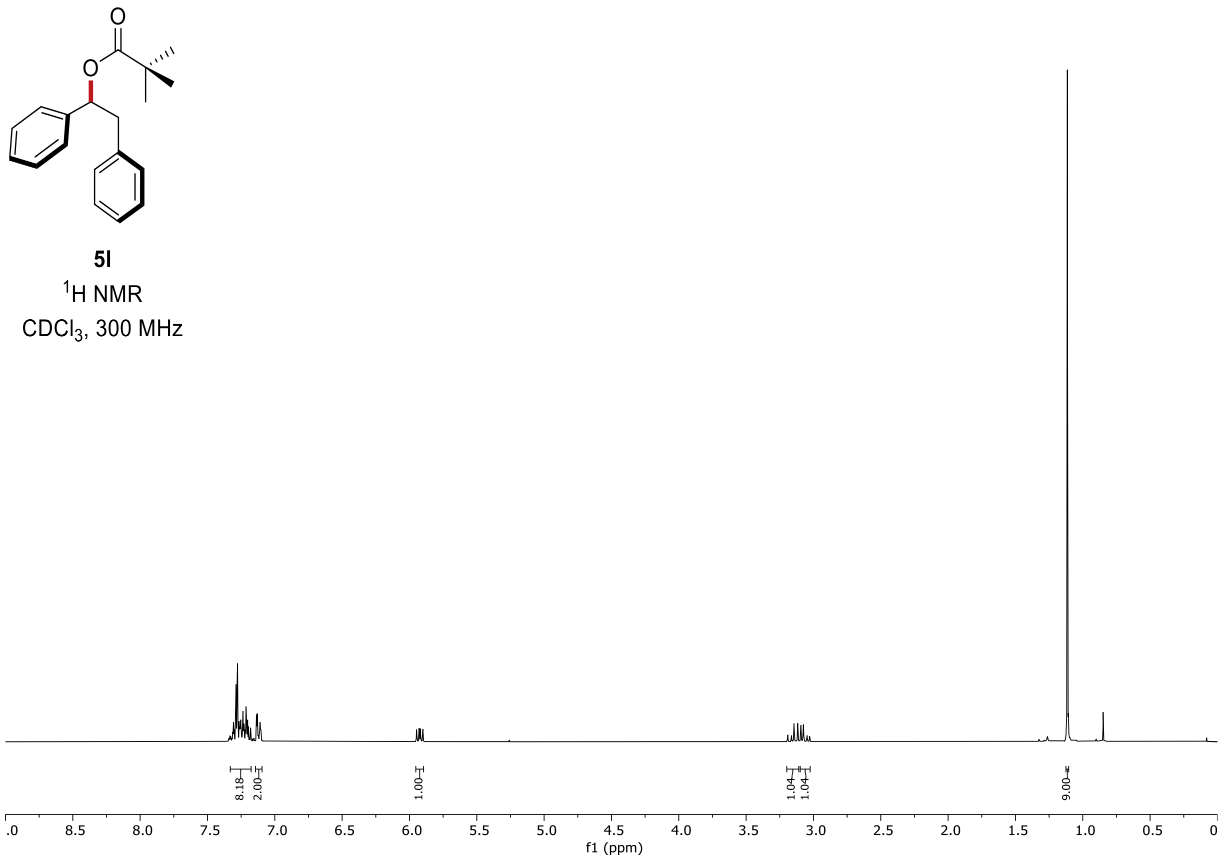
O
O
5l
^{1}H NMR
CDCl$_3$, 300 MHz
8.18
2.00
1.00
1.04
1.04
9.00
f1 (ppm)

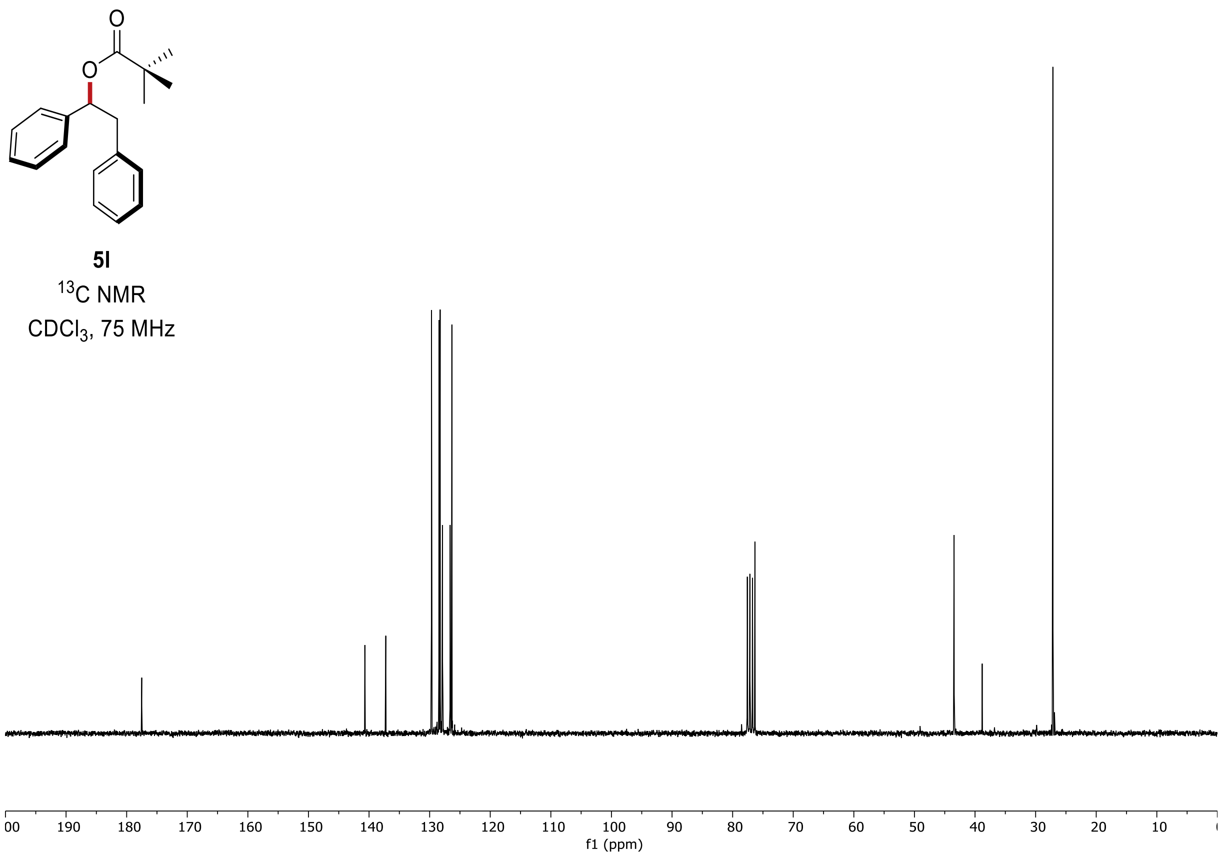
O
O
5l
^{13}C NMR
CDCl$_3$, 75 MHz
f1 (ppm)

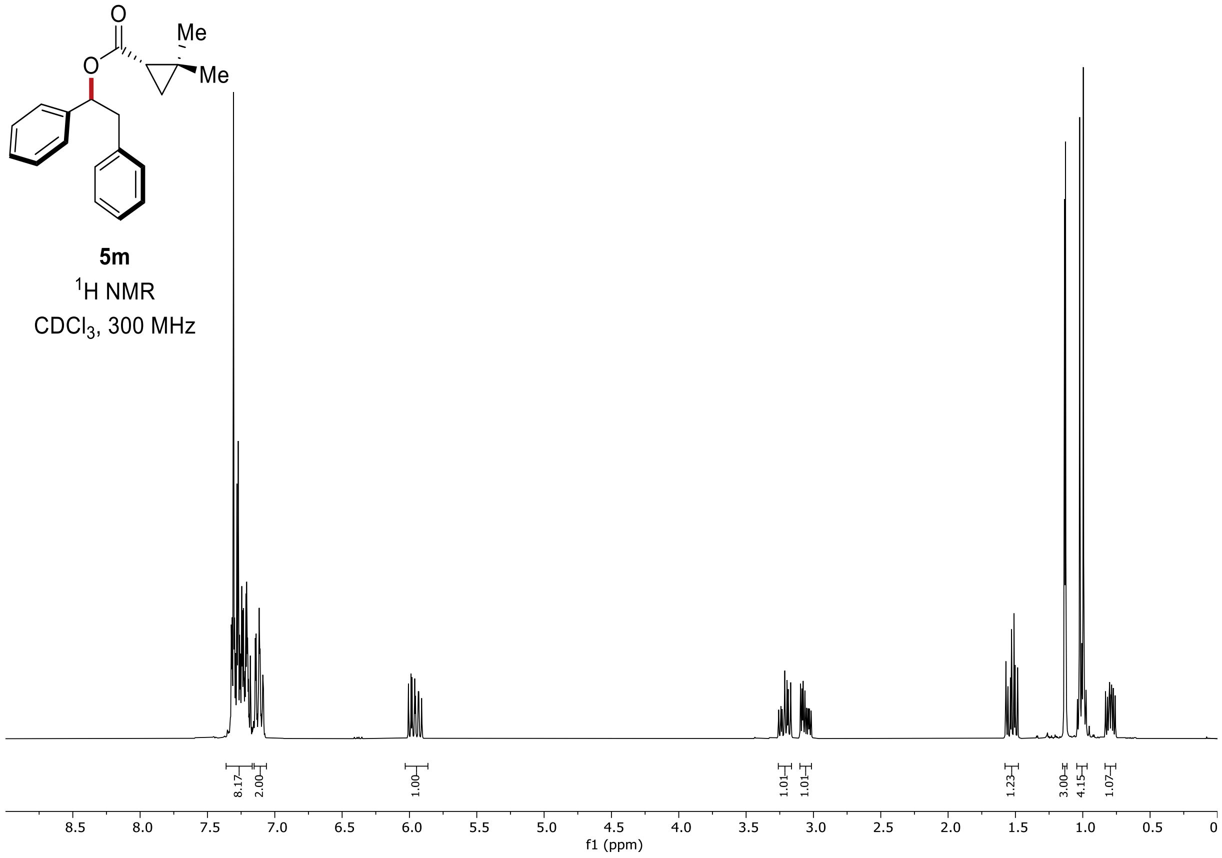

5m
^{1}H NMR
$CDCl_3$, 300 MHz

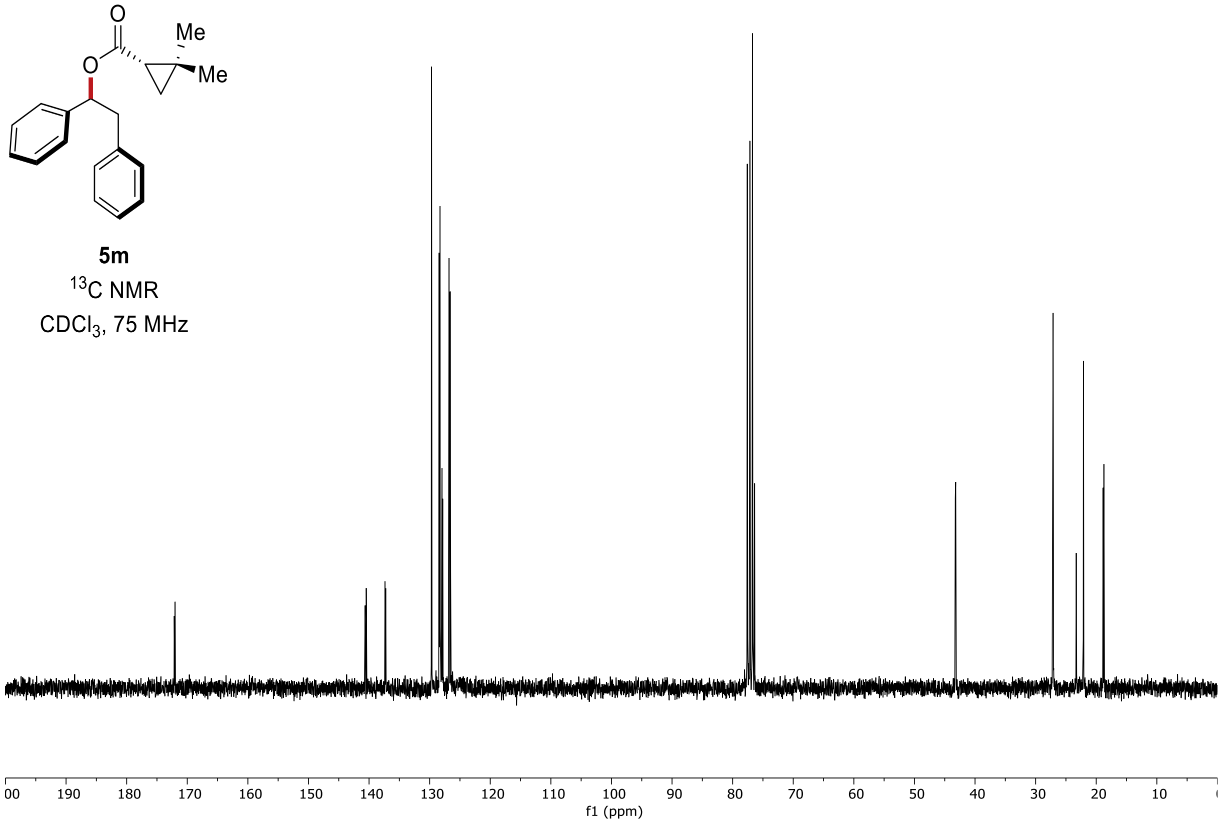

5m
^{13}C NMR
$CDCl_3$, 75 MHz

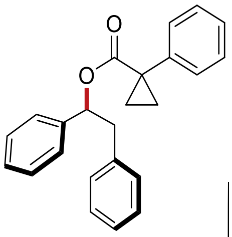

5n

^{1}H NMR

$CDCl_3$, 300 MHz

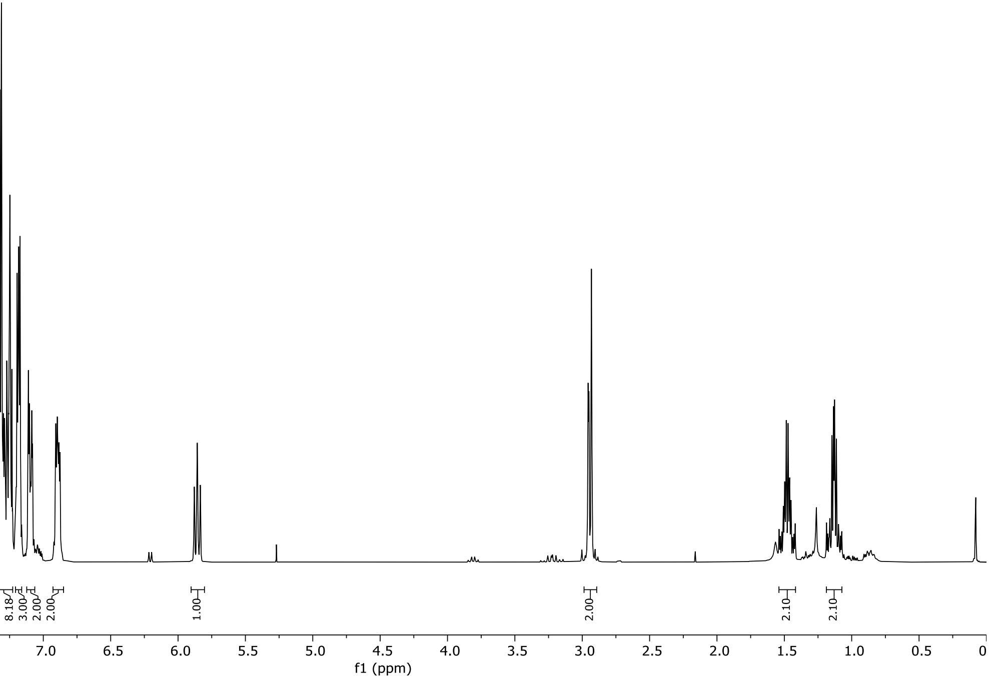

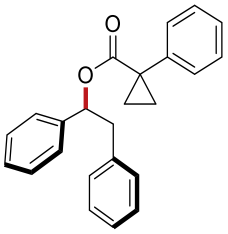

5n

^{13}C NMR

$CDCl_3$, 75 MHz

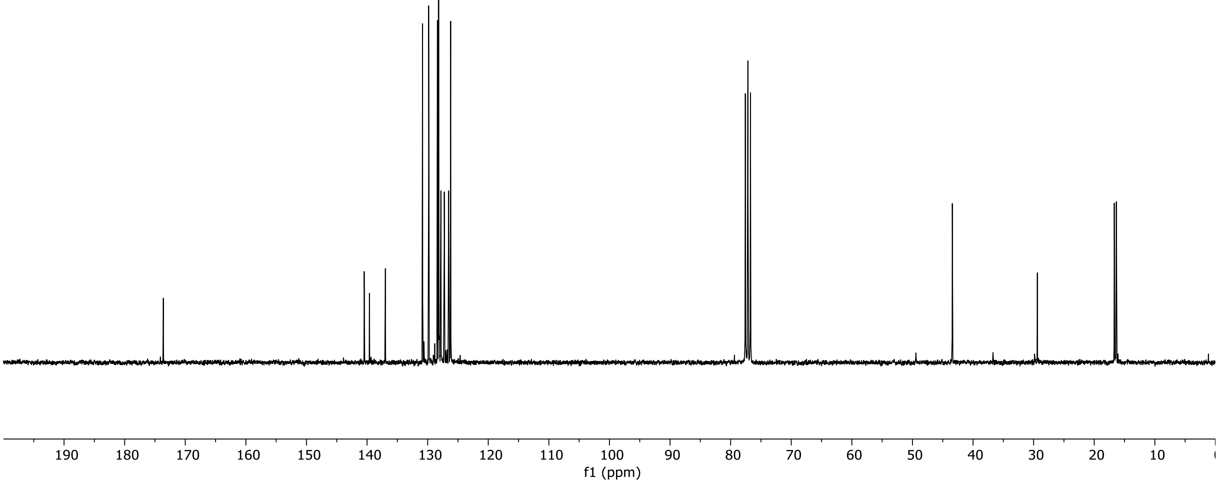

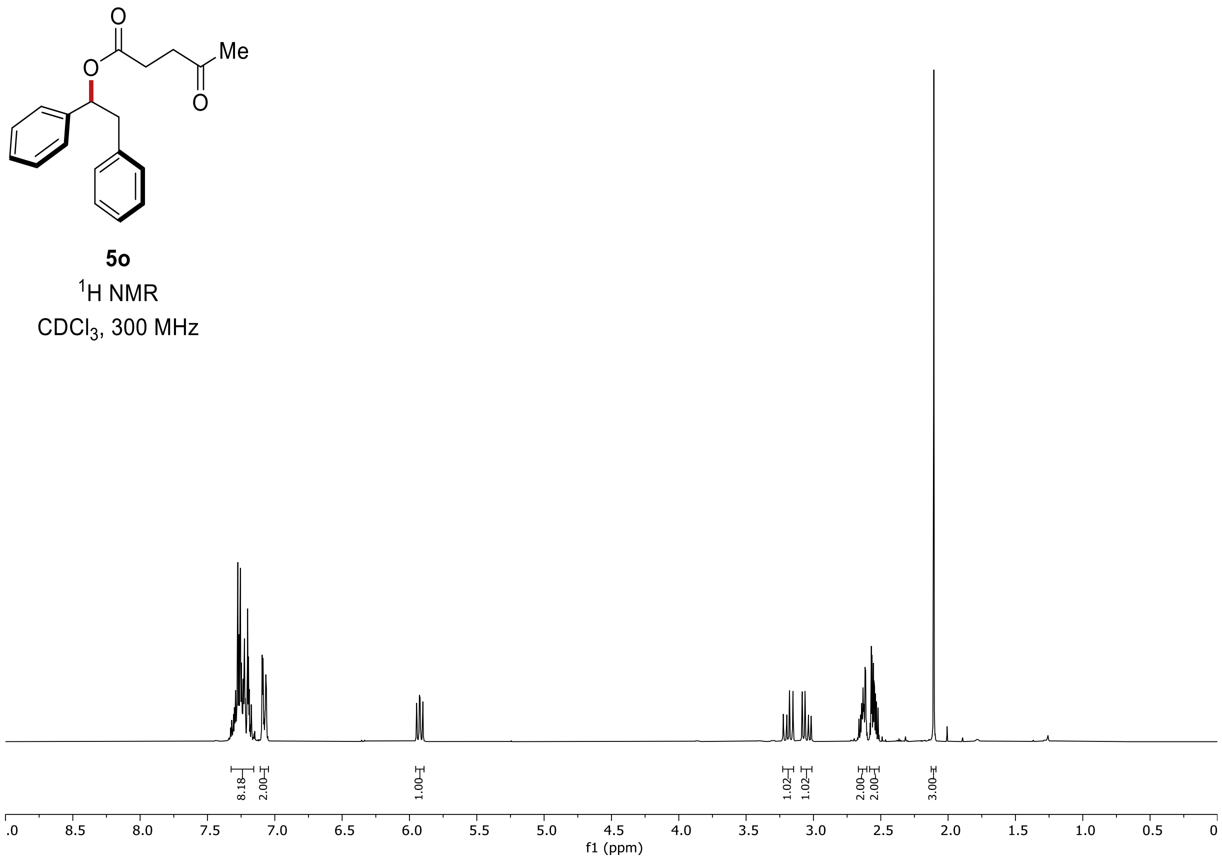
O
O
Me
O
5o
^{1}H NMR
$CDCl_3$, 300 MHz
8.18
2.00
1.00
1.02
1.02
2.00
2.00
3.00
f1 (ppm)

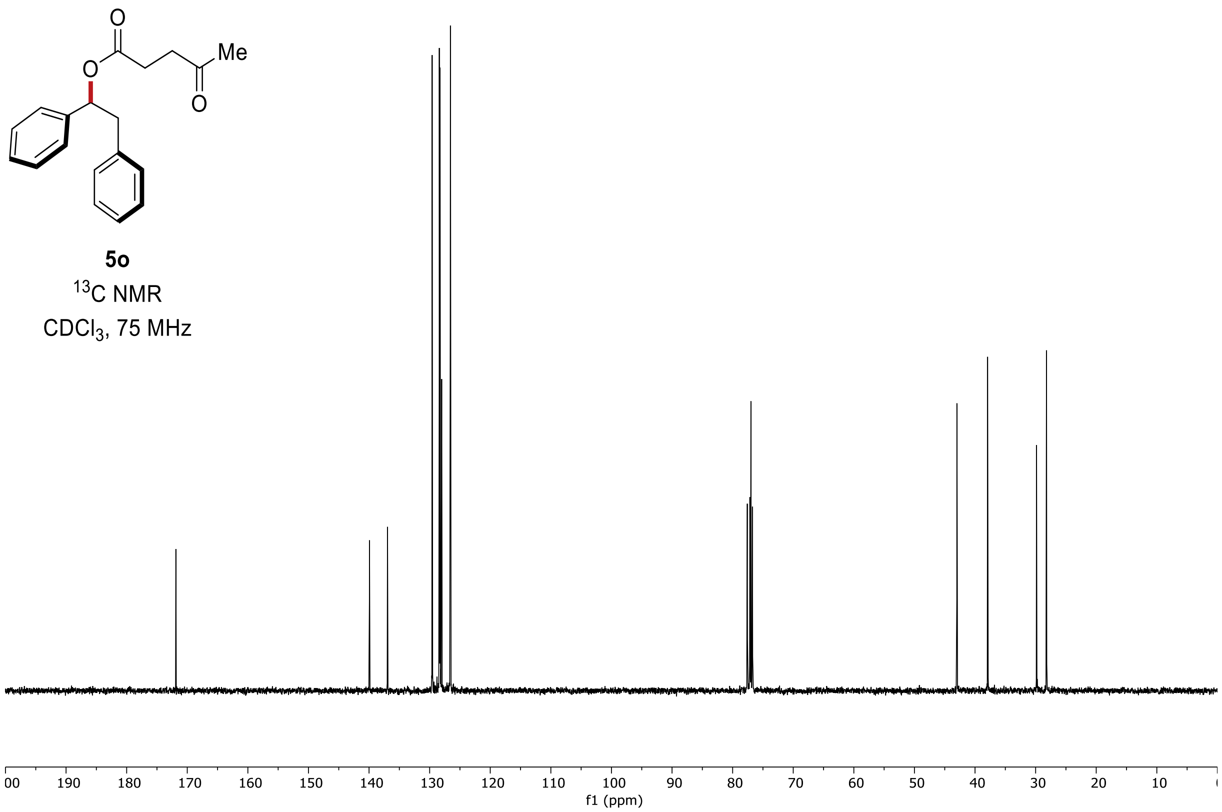
O
O
Me
O
5o
^{13}C NMR
$CDCl_3$, 75 MHz
f1 (ppm)

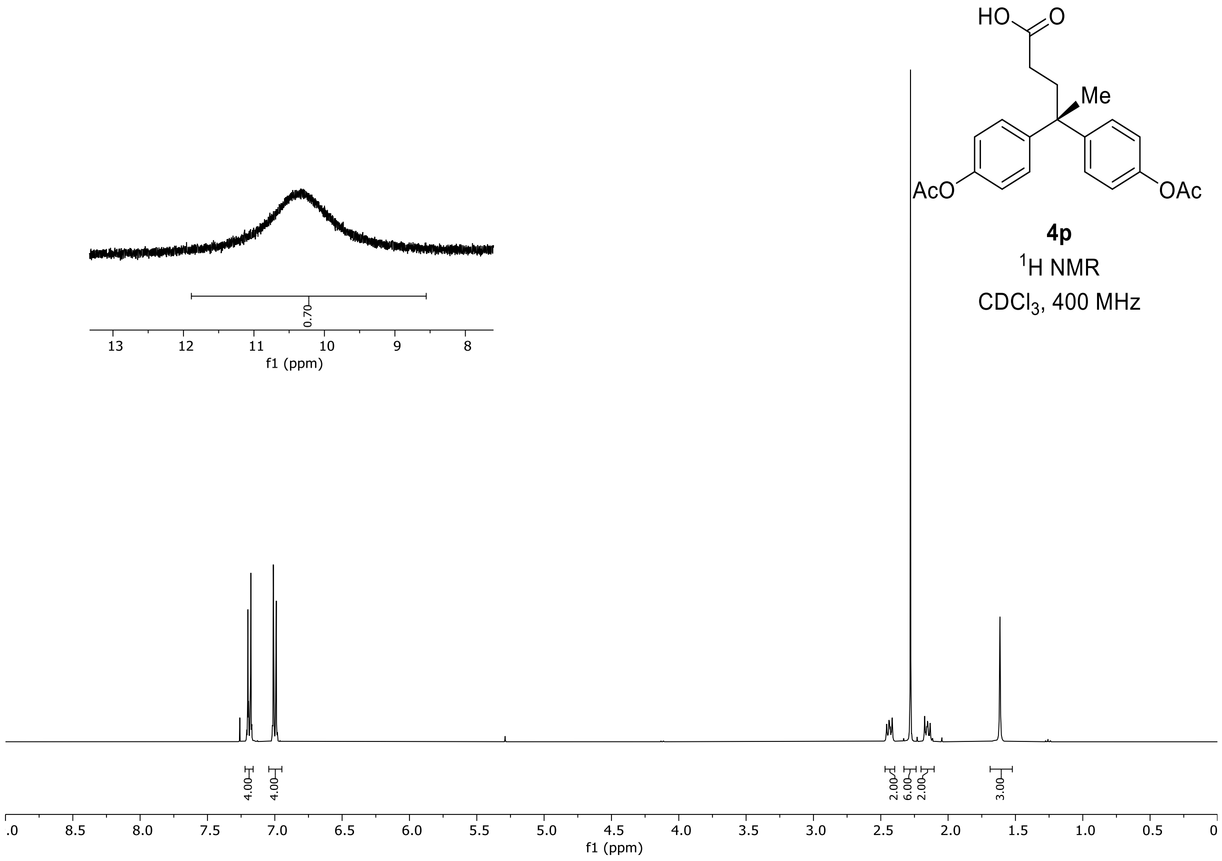
HO
O
Me
AcO
OAc
4p
^{1}H NMR
CDCl$_3$, 400 MHz
f1 (ppm)

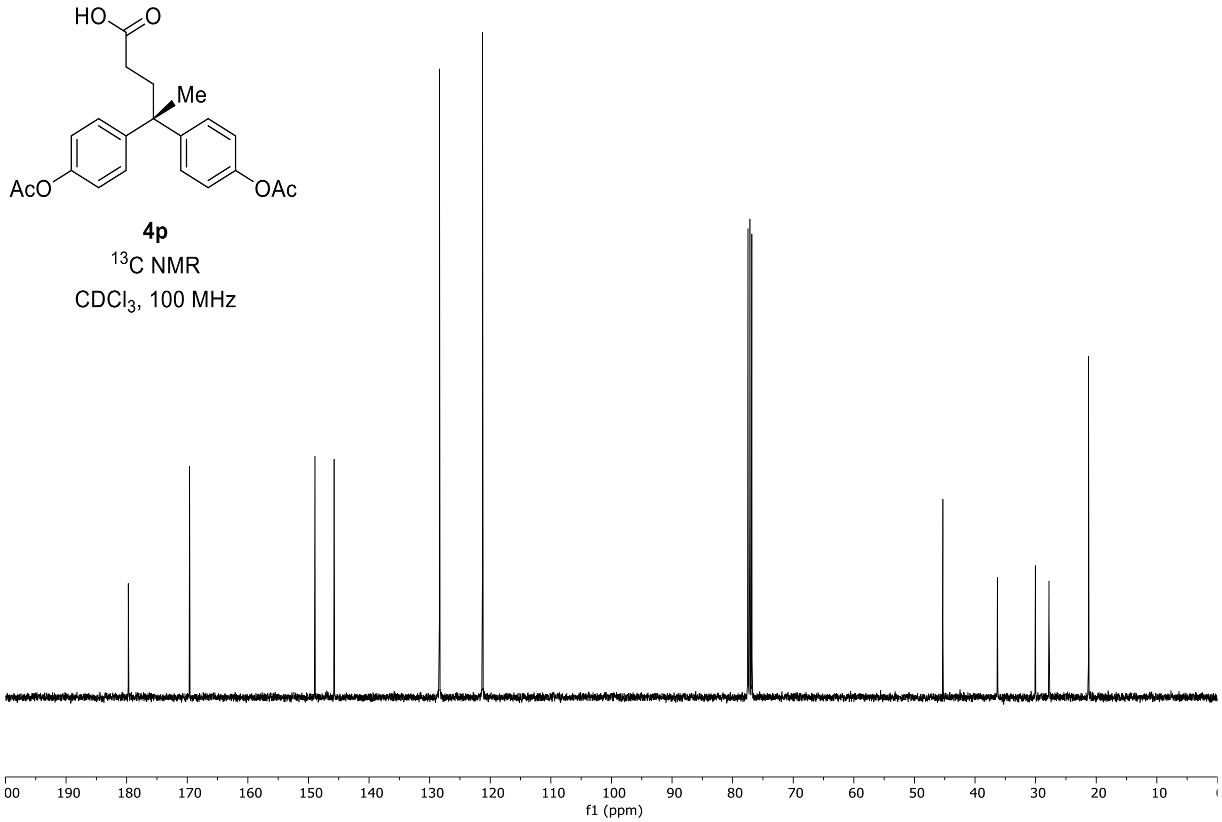
HO
O
Me
AcO
OAc
4p
^{13}C NMR
CDCl$_3$, 100 MHz
f1 (ppm)

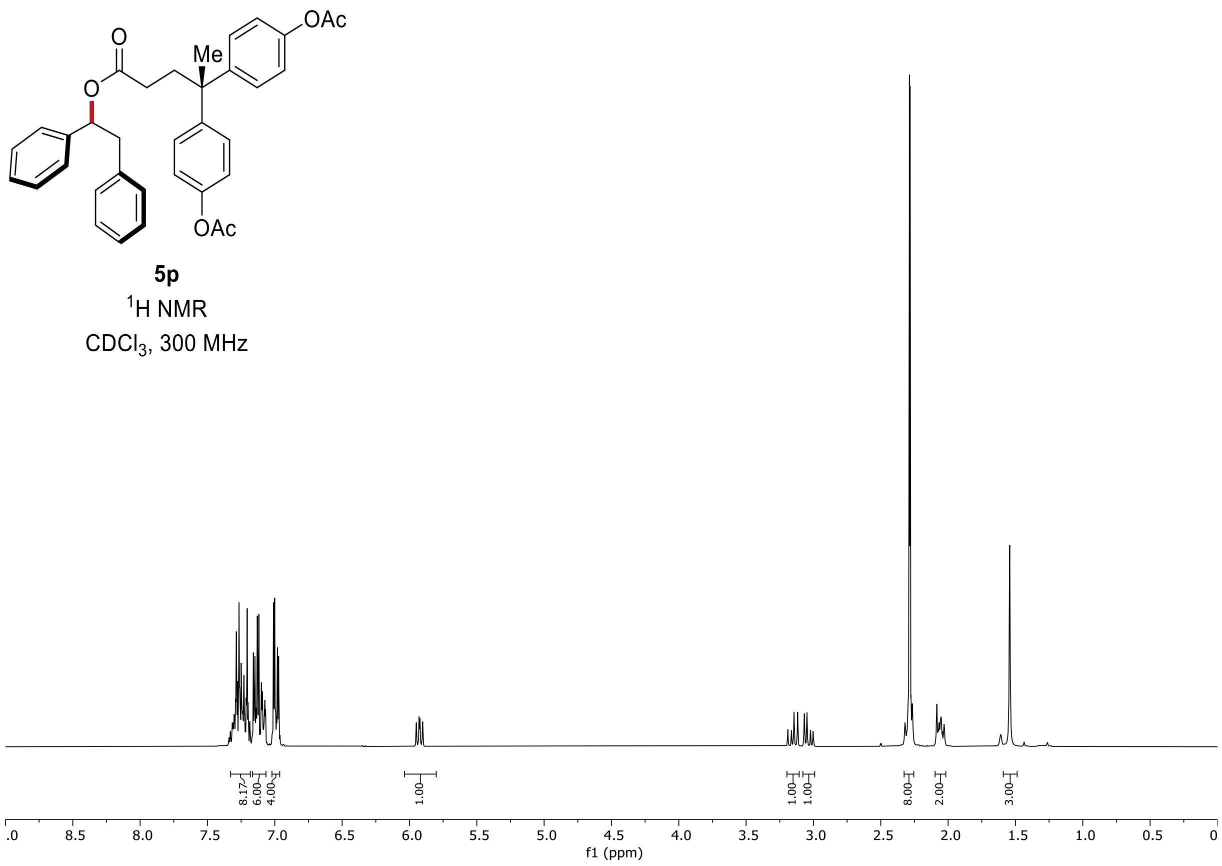
OAc
O
Me
O
OAc
5p
^{1}H NMR
$CDCl_3$, 300 MHz
8.17
6.00
4.00
1.00
1.00
1.00
8.00
2.00
3.00
f1 (ppm)

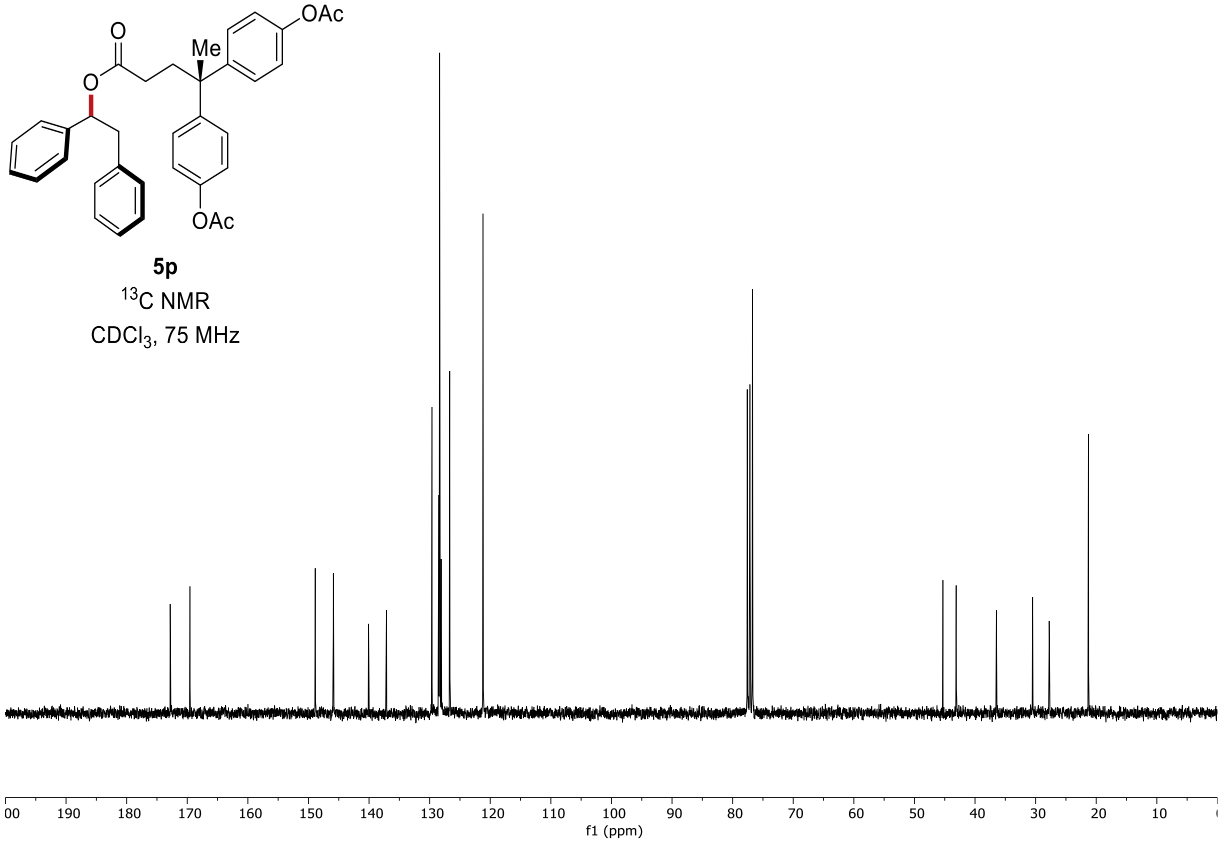
OAc
O
Me
O
OAc
5p
^{13}C NMR
$CDCl_3$, 75 MHz
f1 (ppm)

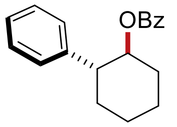

7b
^{1}H NMR
$CDCl_3$, 300 MHz

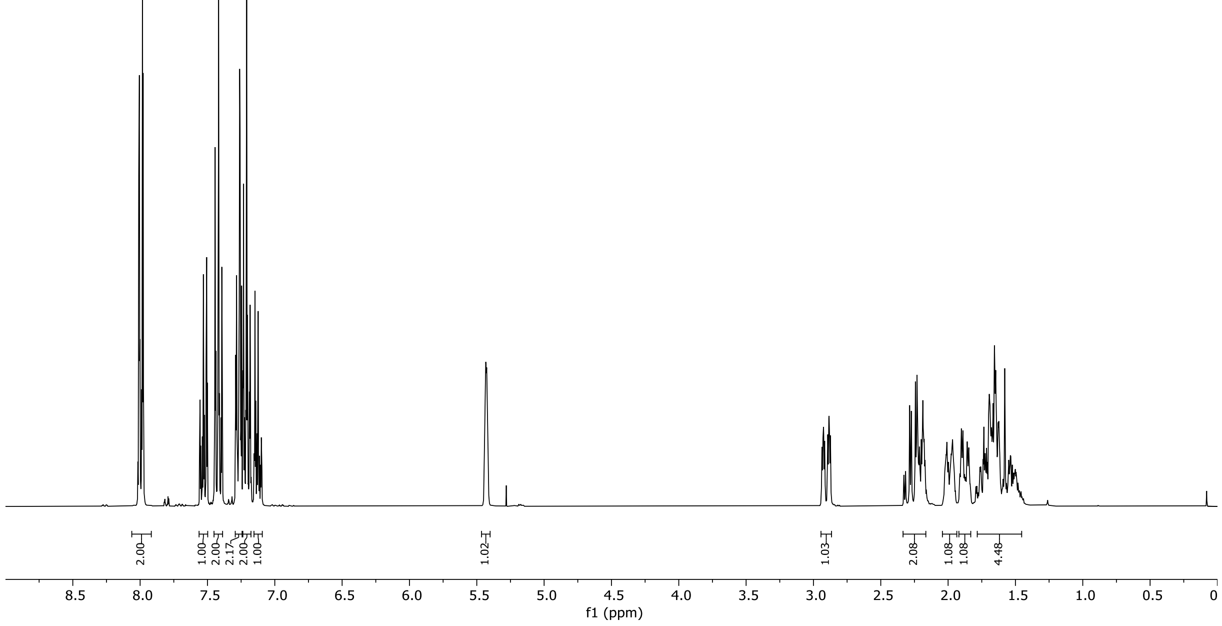

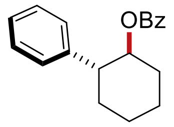

7b
^{13}C NMR
$CDCl_3$, 75 MHz

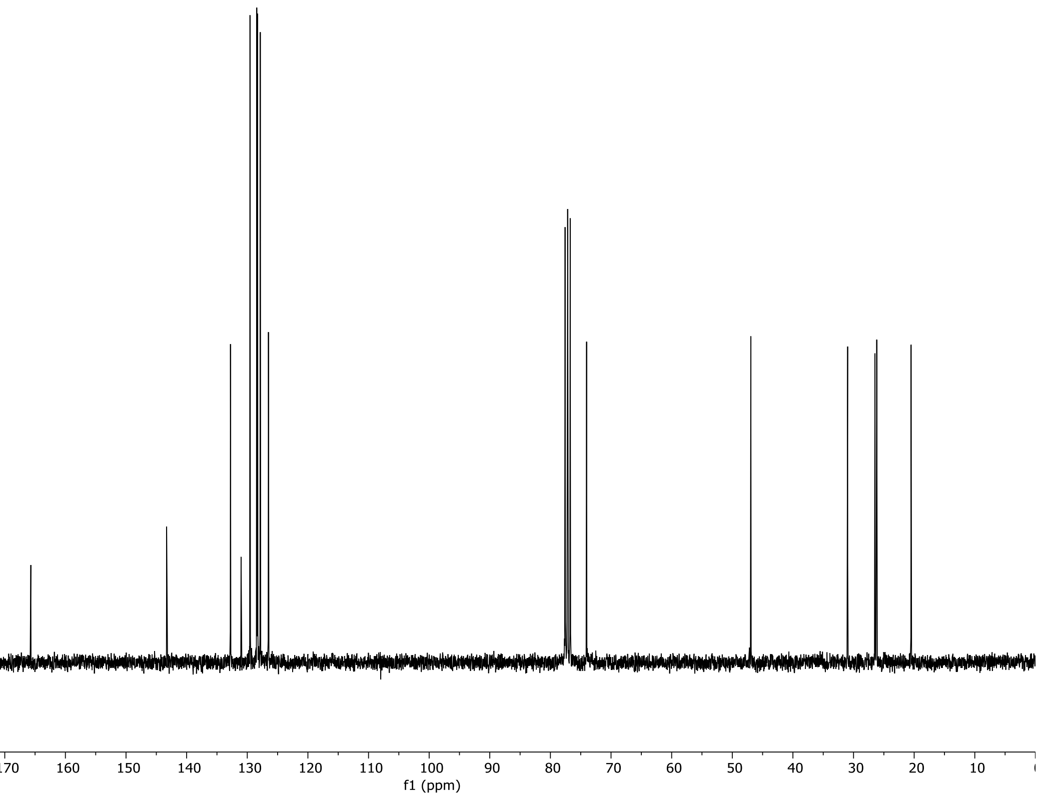

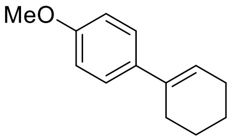

6c
^{1}H NMR
$CDCl_3$, 300 MHz

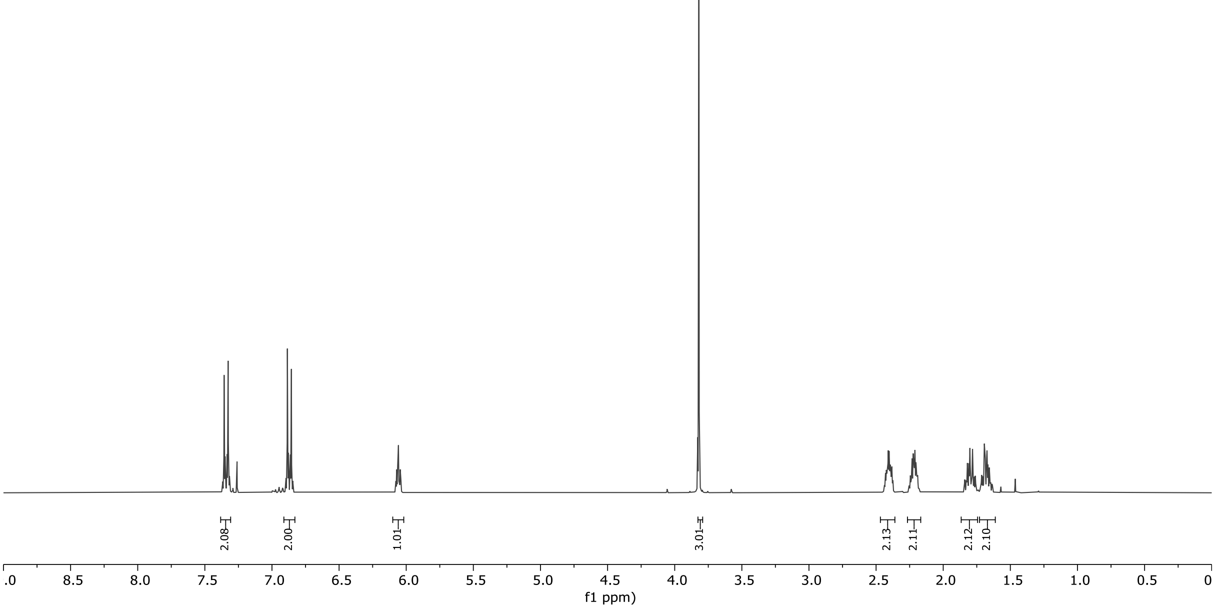

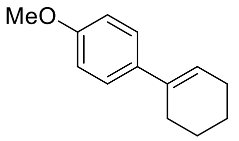

6c
^{13}C NMR
$CDCl_3$, 75 MHz

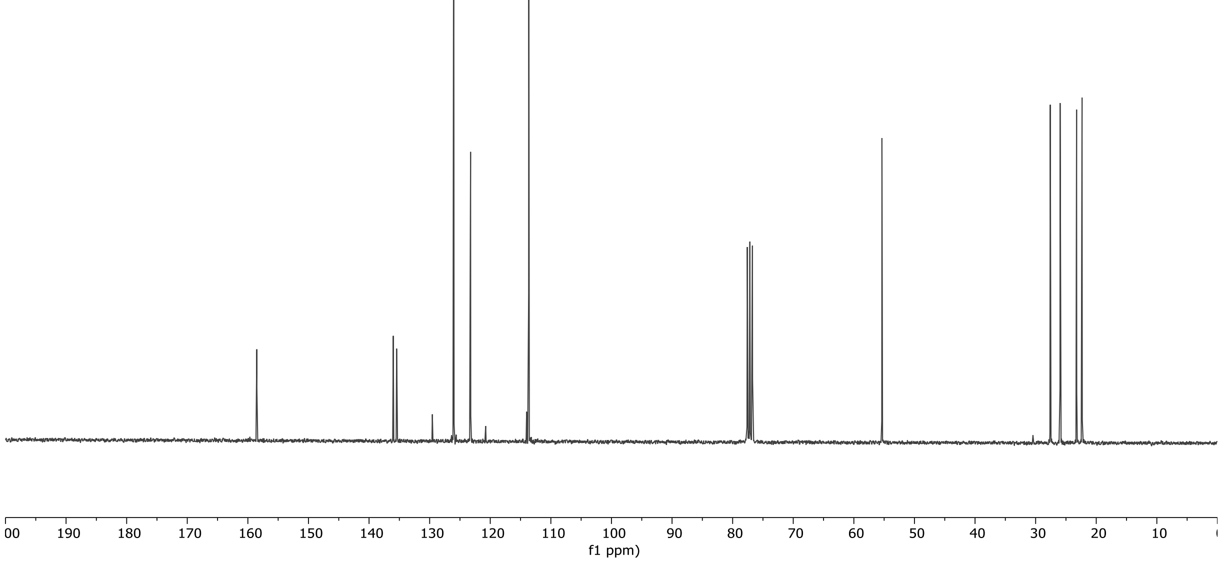

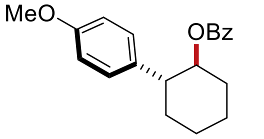

7c
^{1}H NMR
$CDCl_3$, 400 MHz

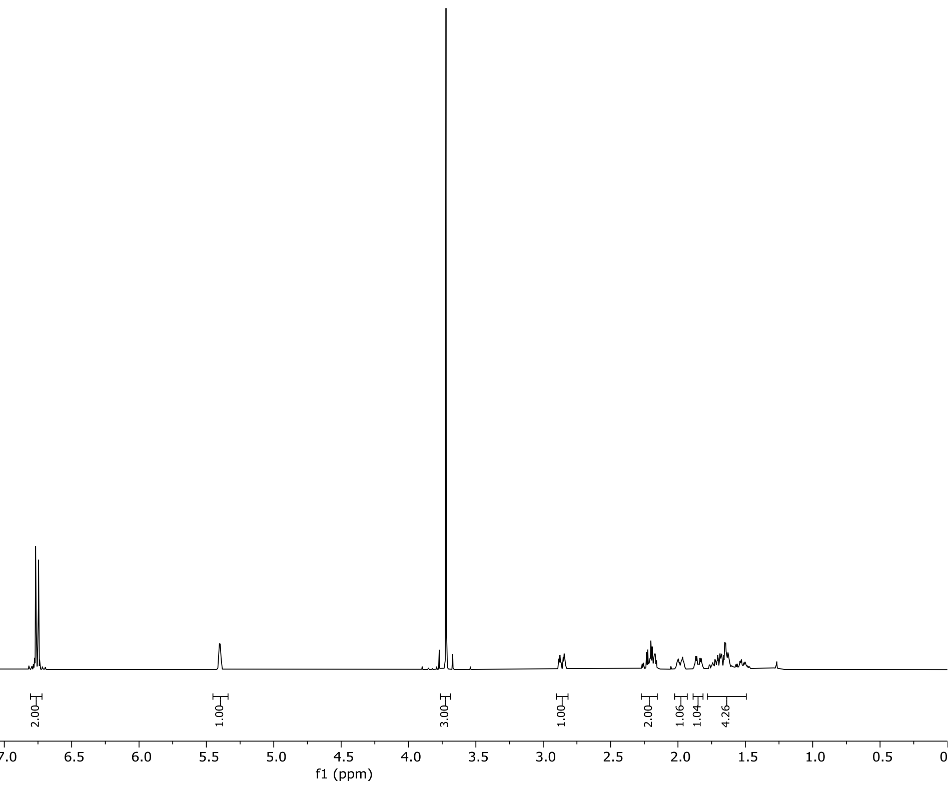

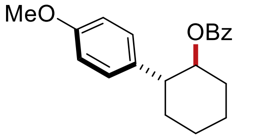

7c
^{13}C NMR
$CDCl_3$, 101 MHz

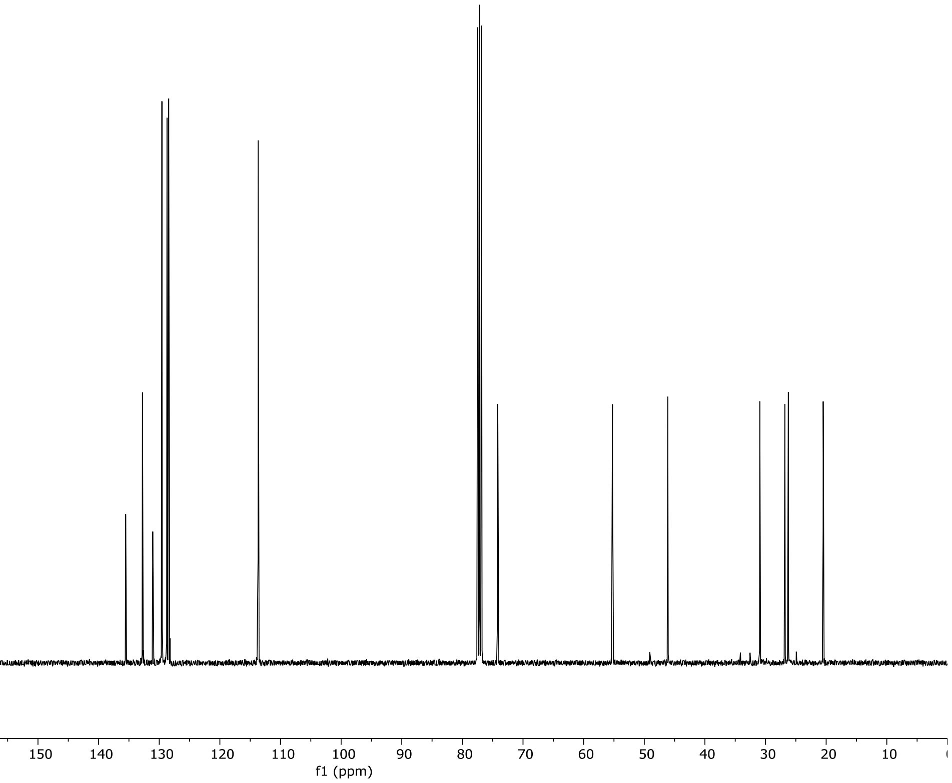

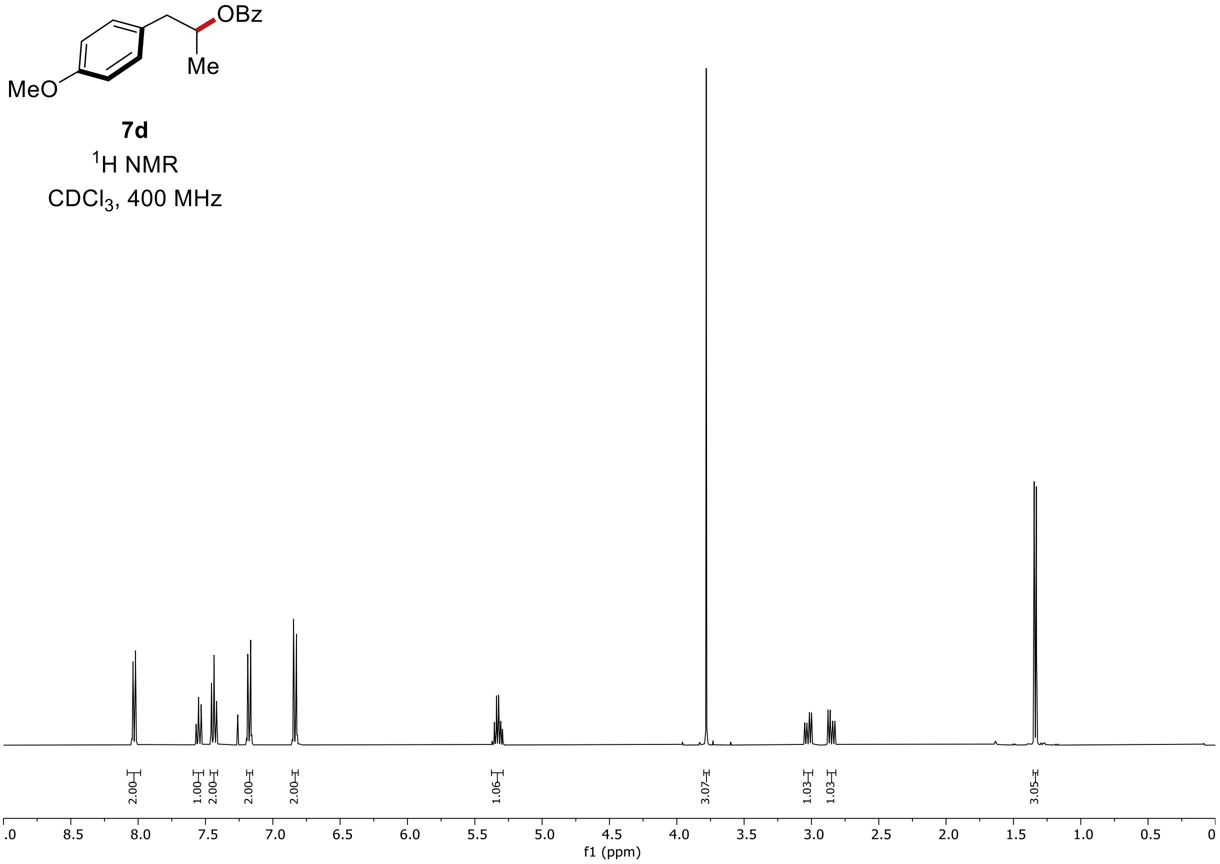
OBz
Me
MeO
7d
^{1}H NMR
$CDCl_3$, 400 MHz
2.00
1.00
2.00
2.00
2.00
1.06
3.07
1.03
1.03
3.05
f1 (ppm)

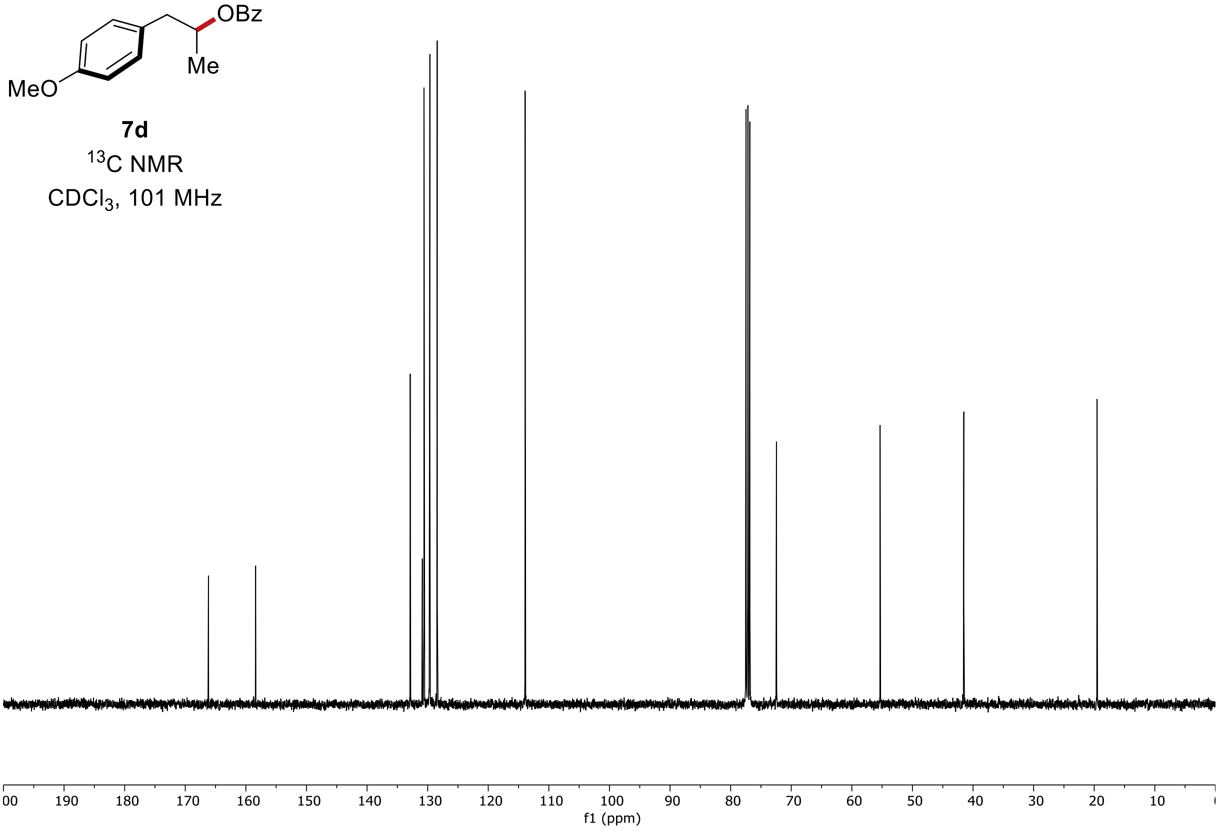
OBz
Me
MeO
7d
^{13}C NMR
$CDCl_3$, 101 MHz
f1 (ppm)

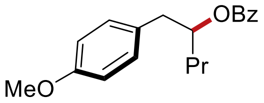

7e
^{1}H NMR
CDCl$_3$, 400 MHz

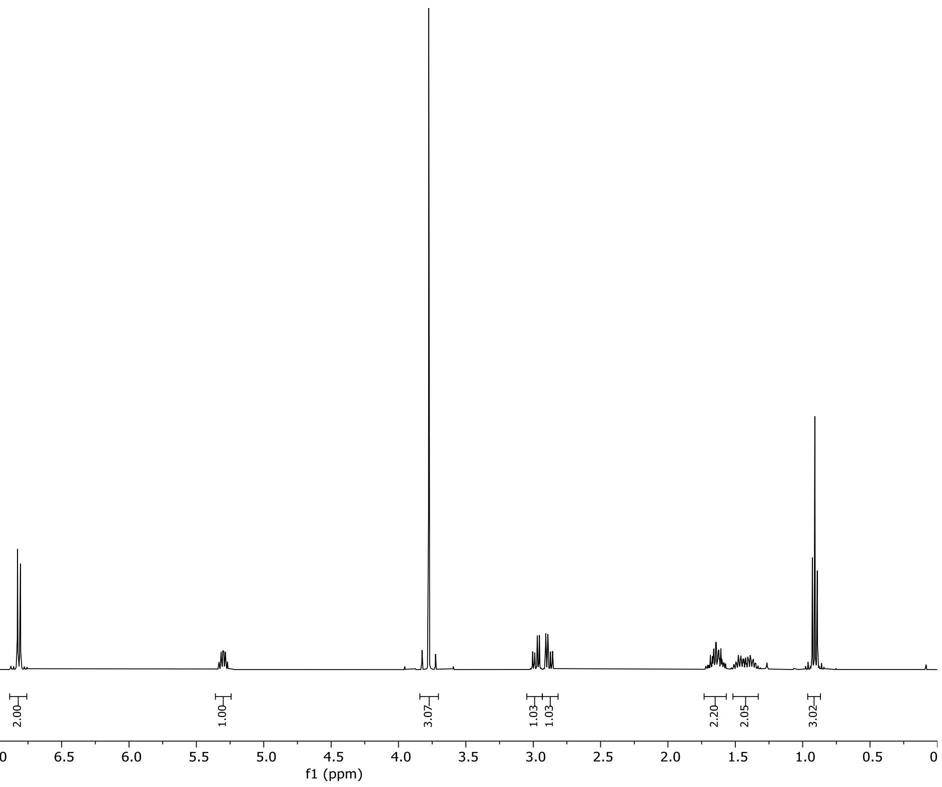

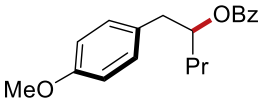

7e
^{13}C NMR
CDCl$_3$, 101 MHz

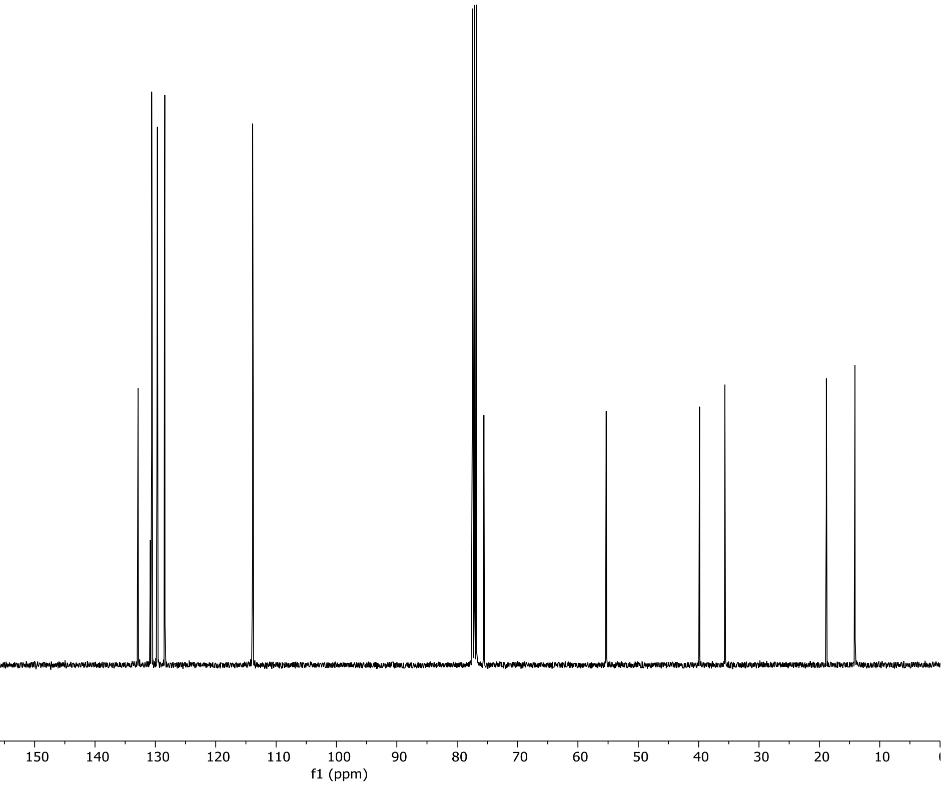

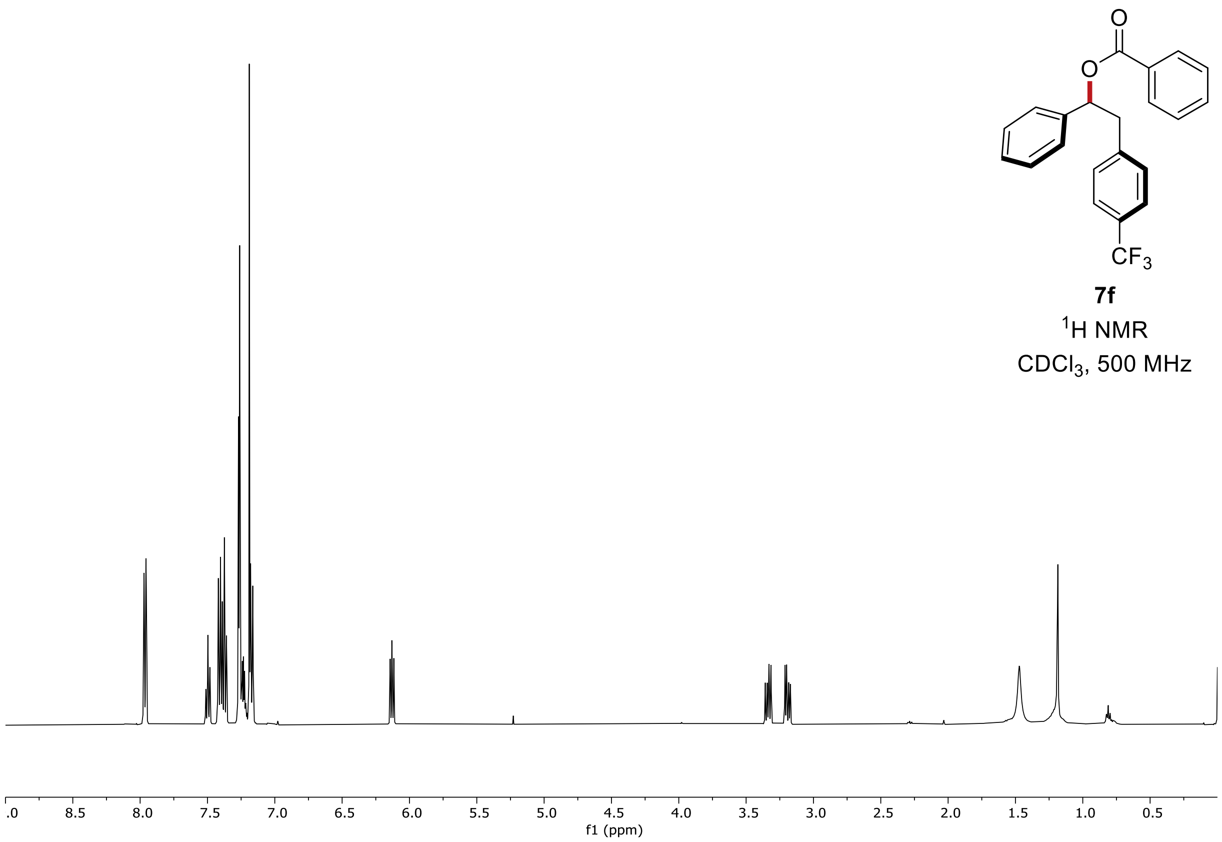

7f
^{1}H NMR
$CDCl_3$, 500 MHz

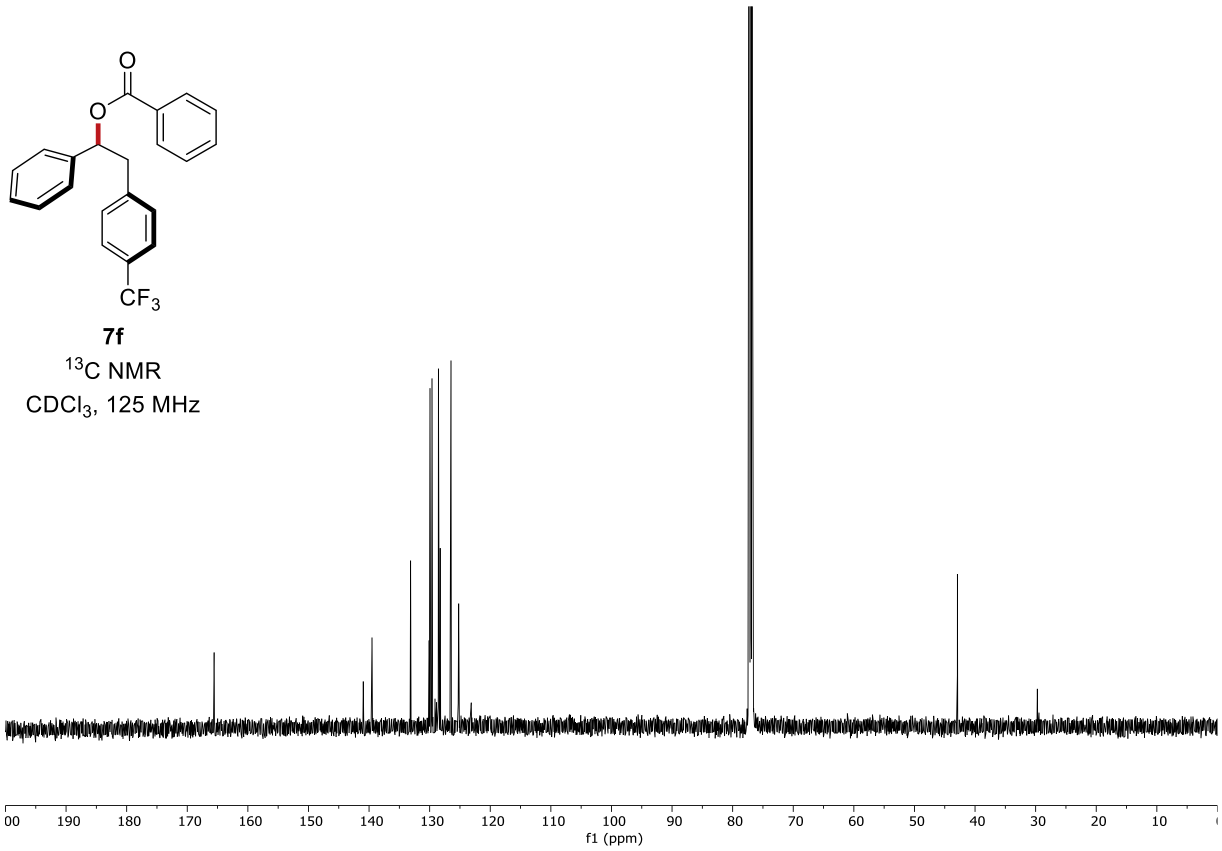

7f
^{13}C NMR
$CDCl_3$, 125 MHz

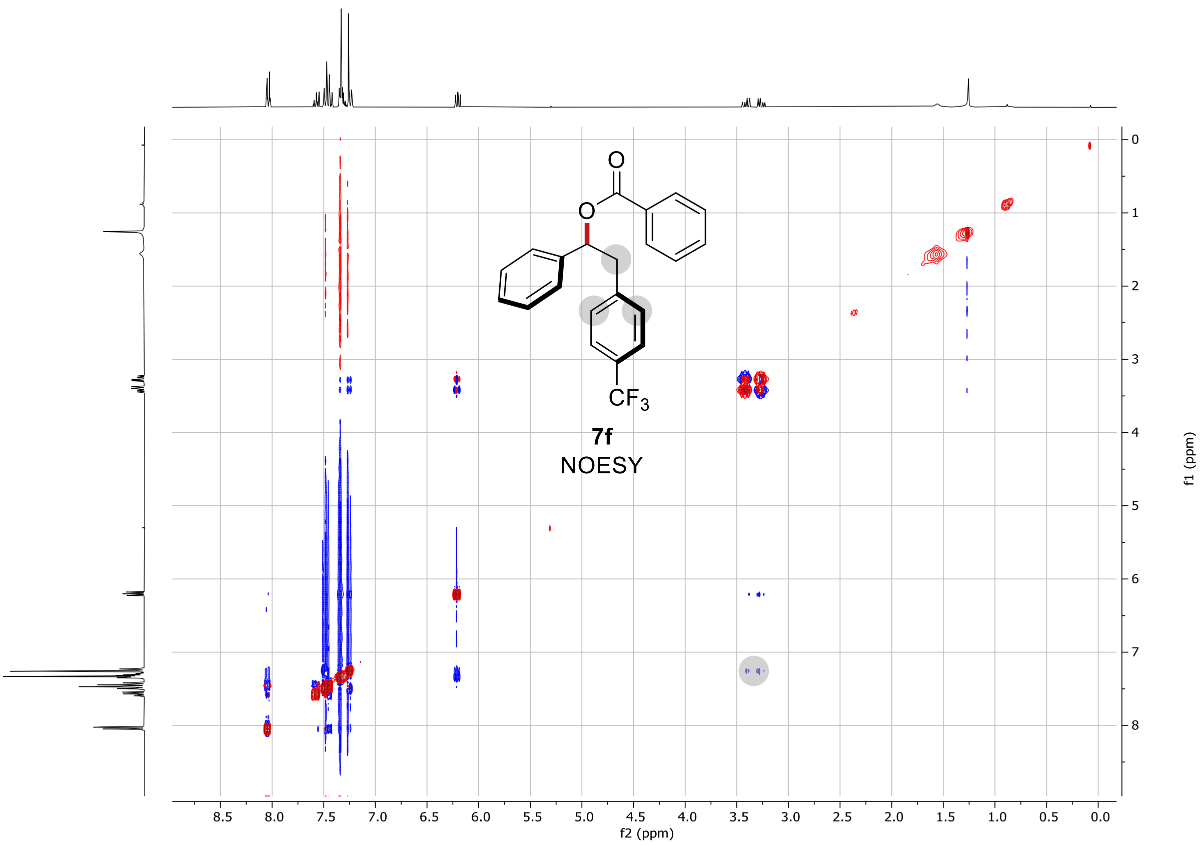

O
O
CF3
7f
NOESY
f2 (ppm)
f1 (ppm)

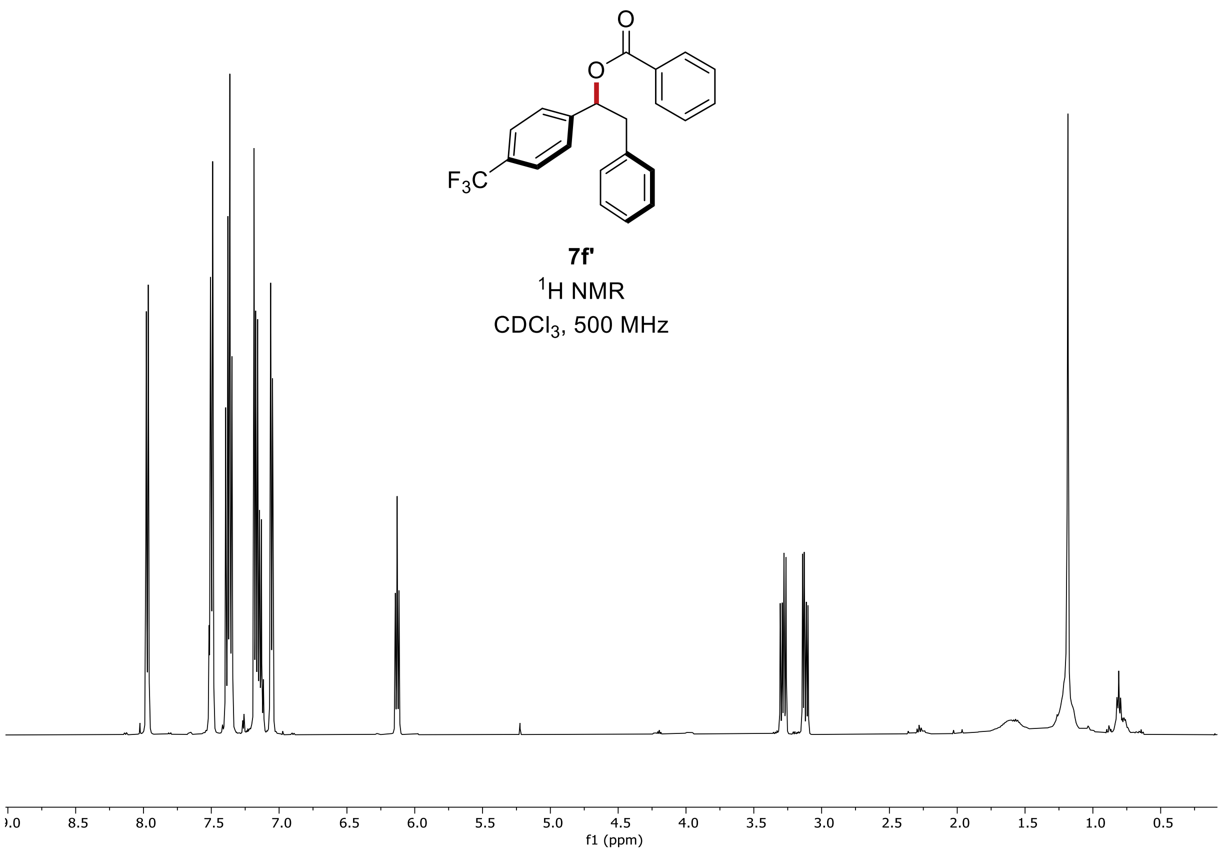
O
O
F3C
7f'
1H NMR
CDCl3, 500 MHz
f1 (ppm)

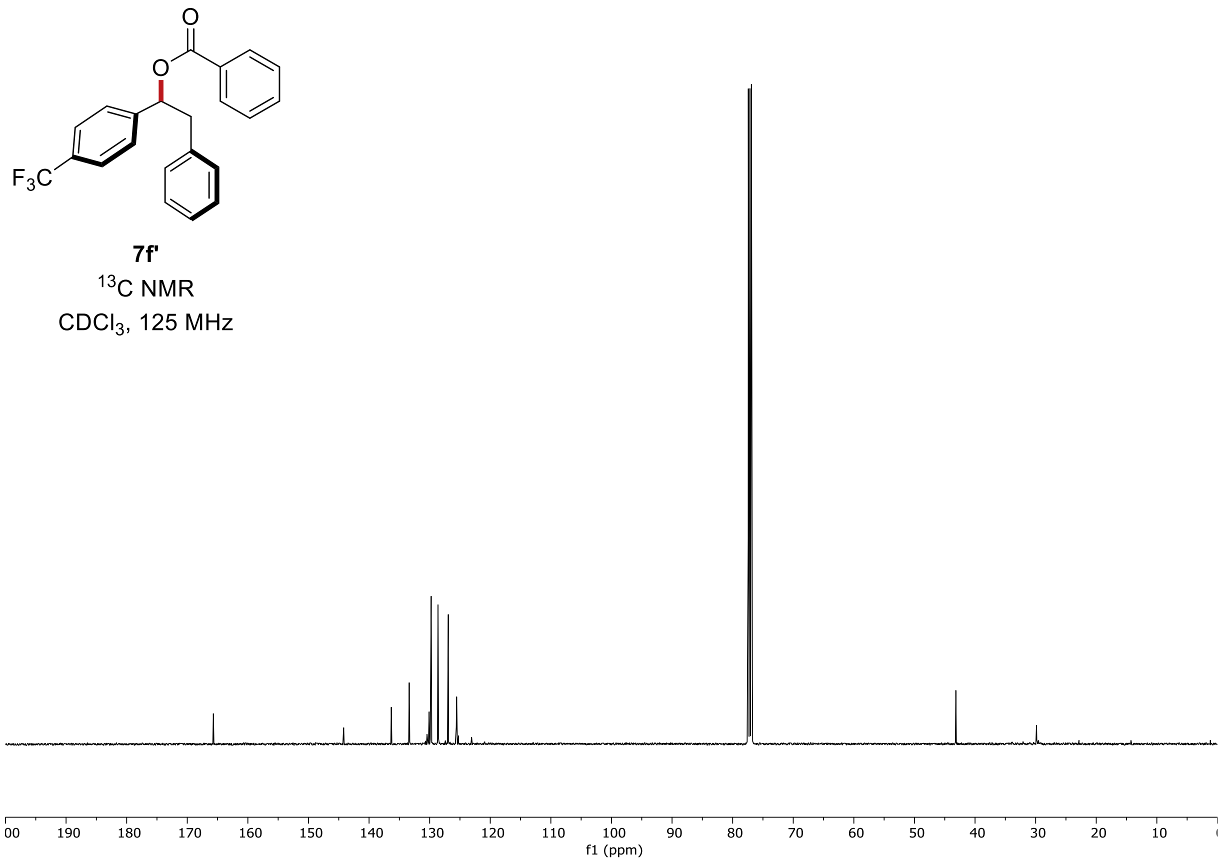
O
O
F3C
7f'
13C NMR
CDCl3, 125 MHz
f1 (ppm)

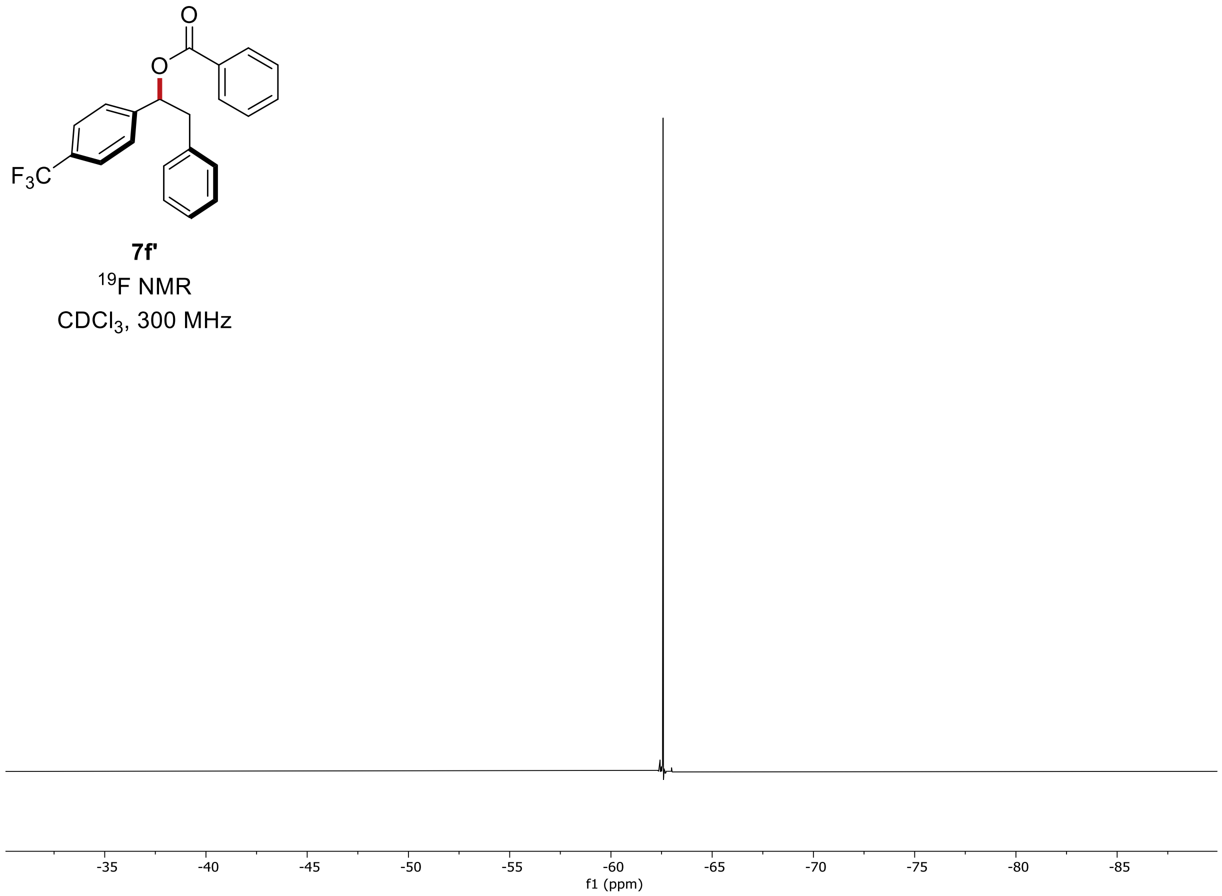
O
O
F3C
7f'
19F NMR
CDCl3, 300 MHz
-35
-40
-45
-50
-55
-60
-65
-70
-75
-80
-85
f1 (ppm)

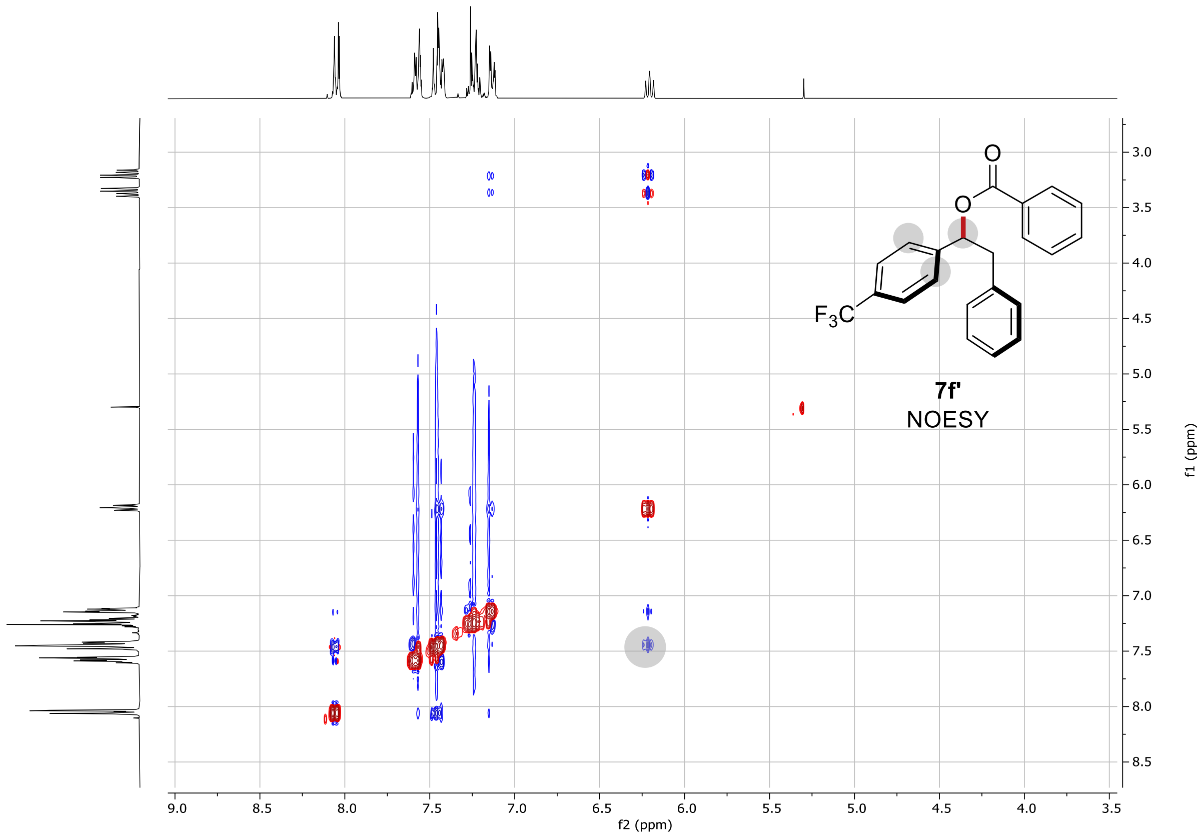
O
O
F3C
7f'
NOESY
9.0
8.5
8.0
7.5
7.0
6.5
6.0
5.5
5.0
4.5
4.0
3.5
f2 (ppm)
3.0
3.5
4.0
4.5
5.0
5.5
6.0
6.5
7.0
7.5
8.0
8.5
f1 (ppm)

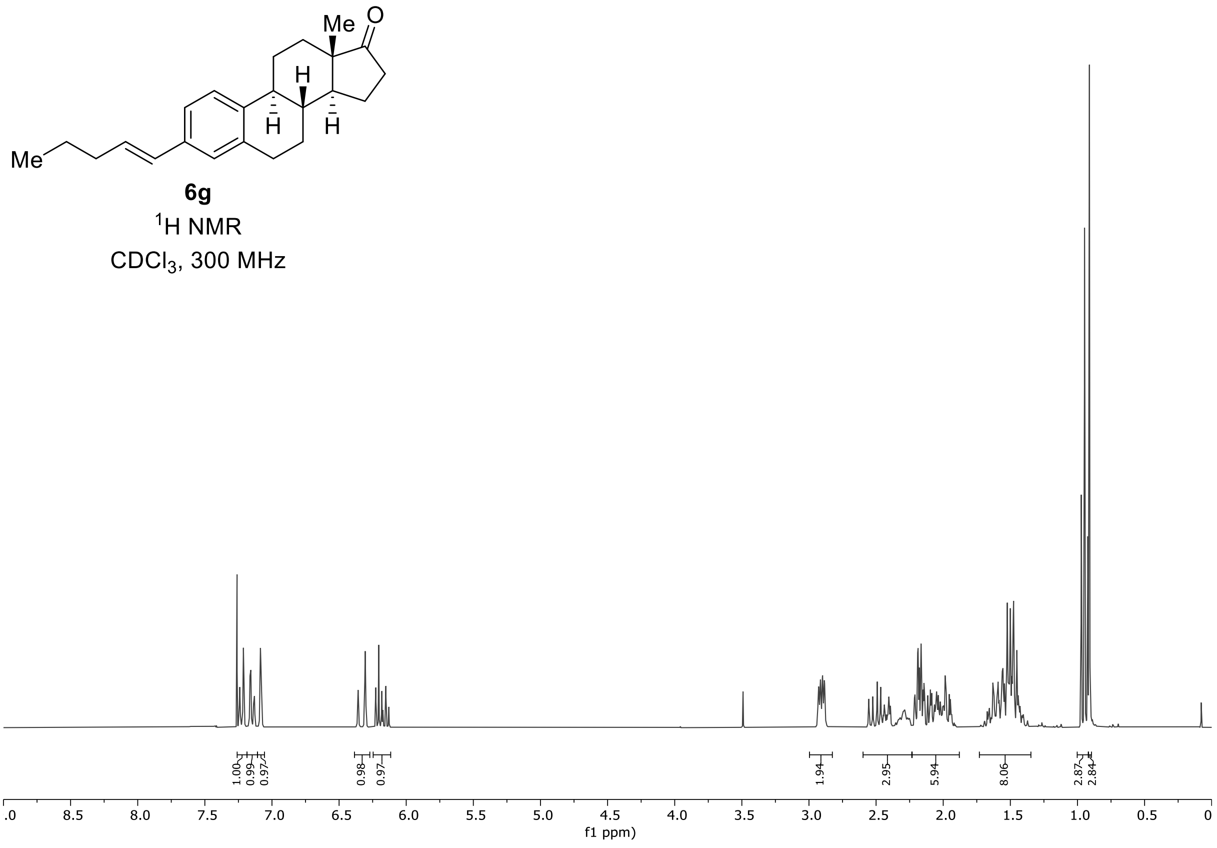

6g

^{1}H NMR

$CDCl_3$, 300 MHz

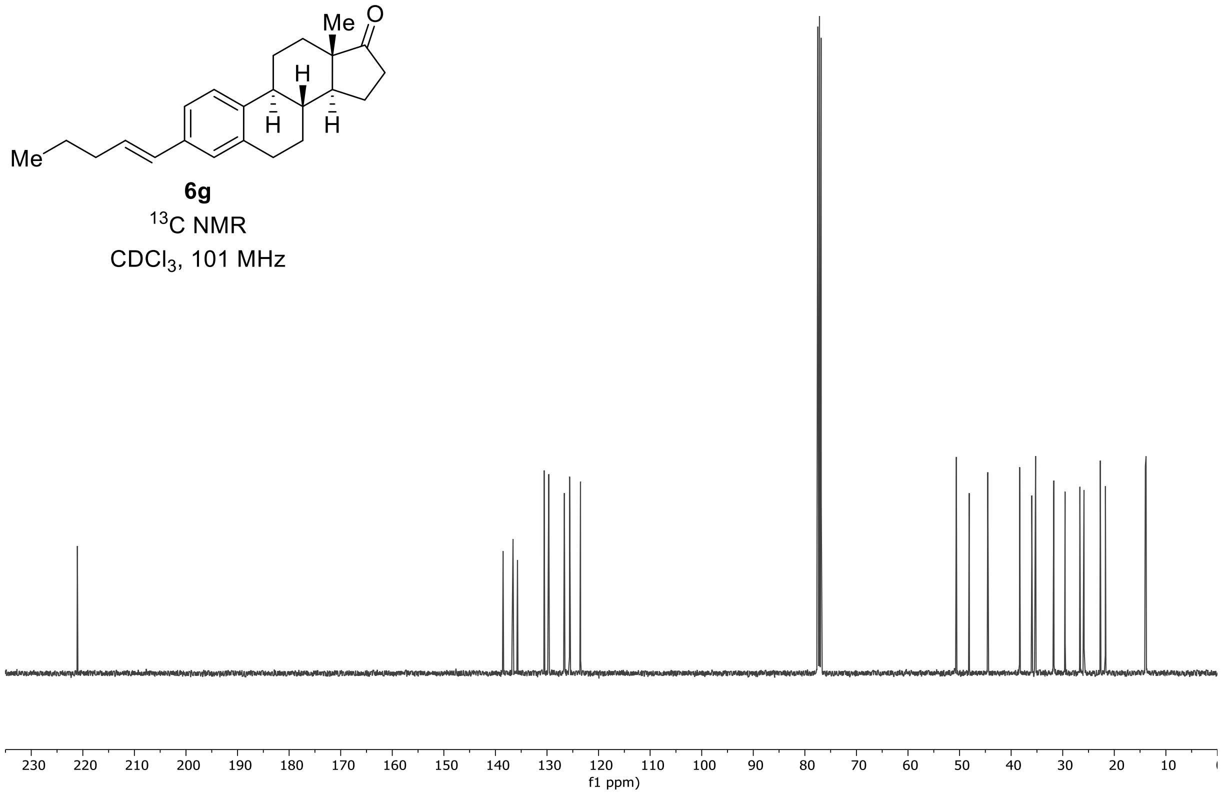

6g

^{13}C NMR

$CDCl_3$, 101 MHz

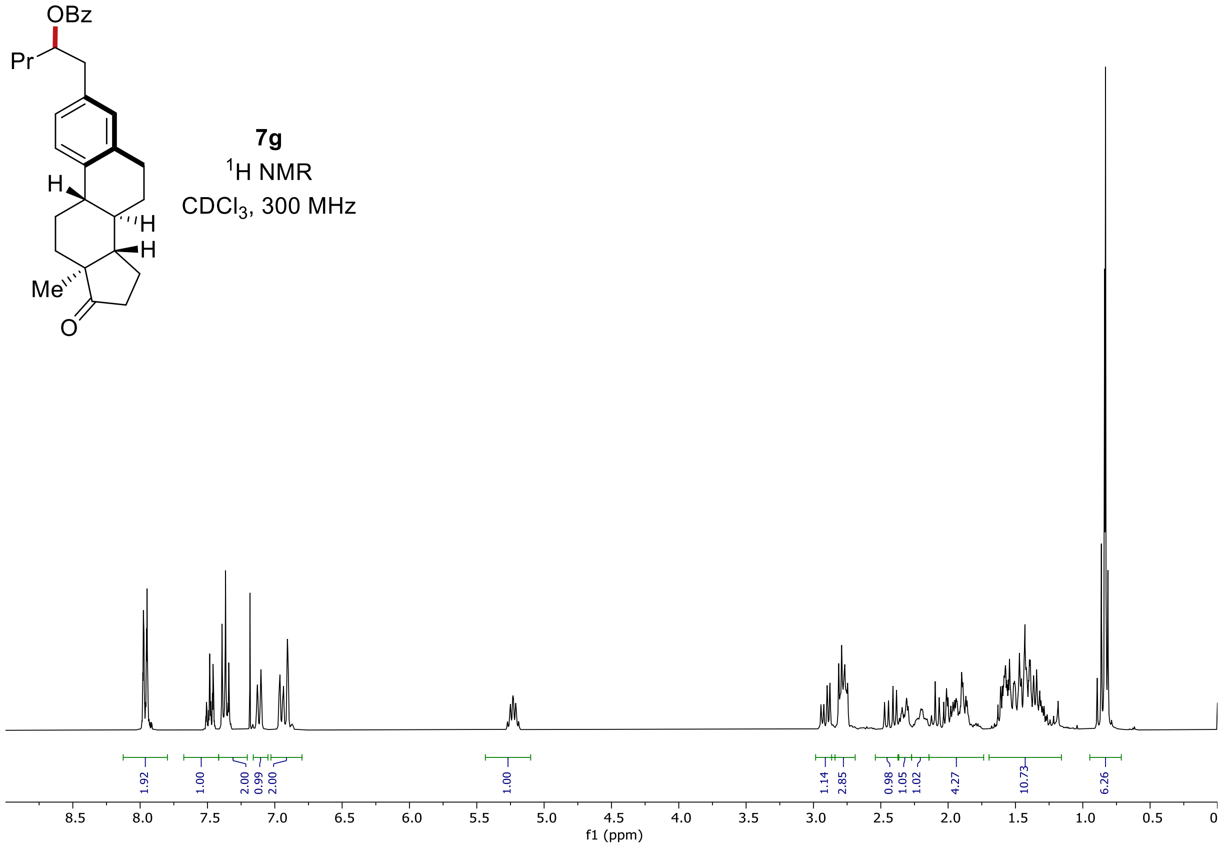
OBz
Pr
H
H
H
Me
O
7g
^{1}H NMR
$CDCl_3$, 300 MHz
1.92
1.00
2.00
0.99
2.00
1.00
1.14
2.85
0.98
1.05
1.02
4.27
10.73
6.26
8.5 8.0 7.5 7.0 6.5 6.0 5.5 5.0 4.5 4.0 3.5 3.0 2.5 2.0 1.5 1.0 0.5 0
f1 (ppm)

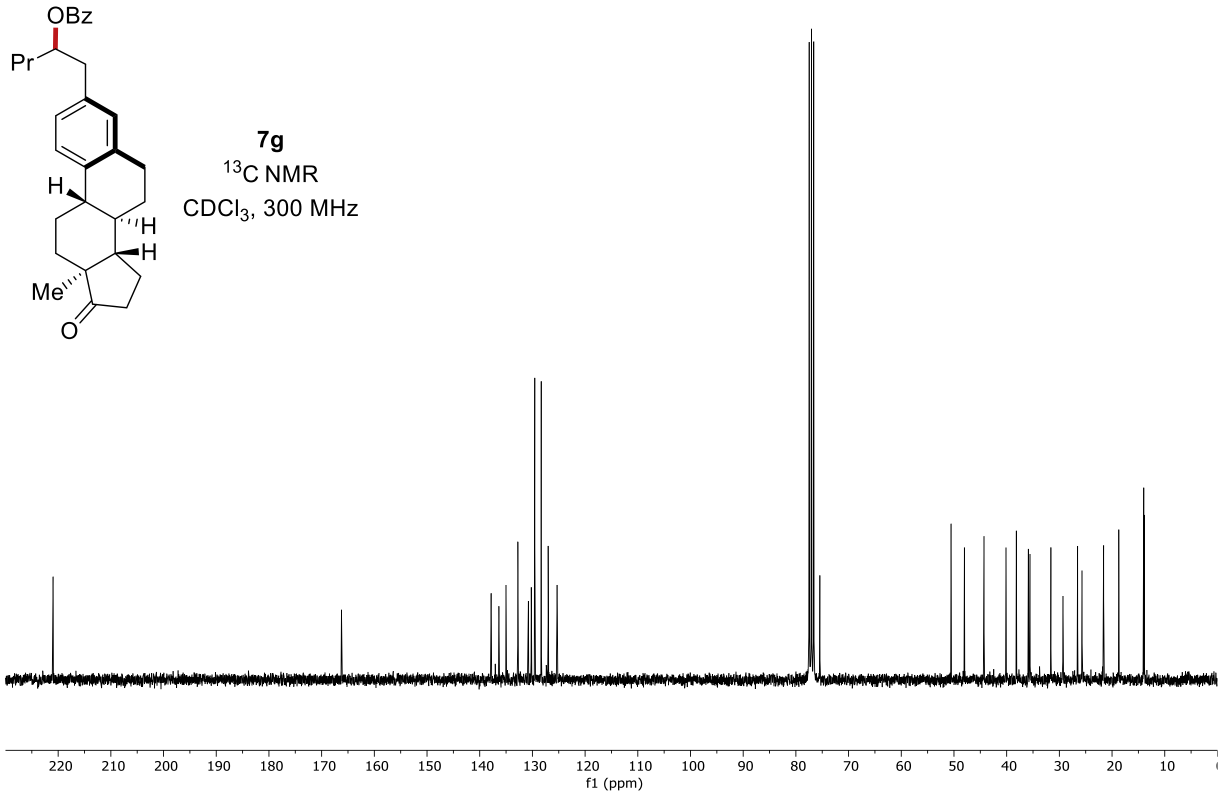
OBz
Pr
H
H
H
Me
O
7g
^{13}C NMR
$CDCl_3$, 300 MHz
220 210 200 190 180 170 160 150 140 130 120 110 100 90 80 70 60 50 40 30 20 10
f1 (ppm)

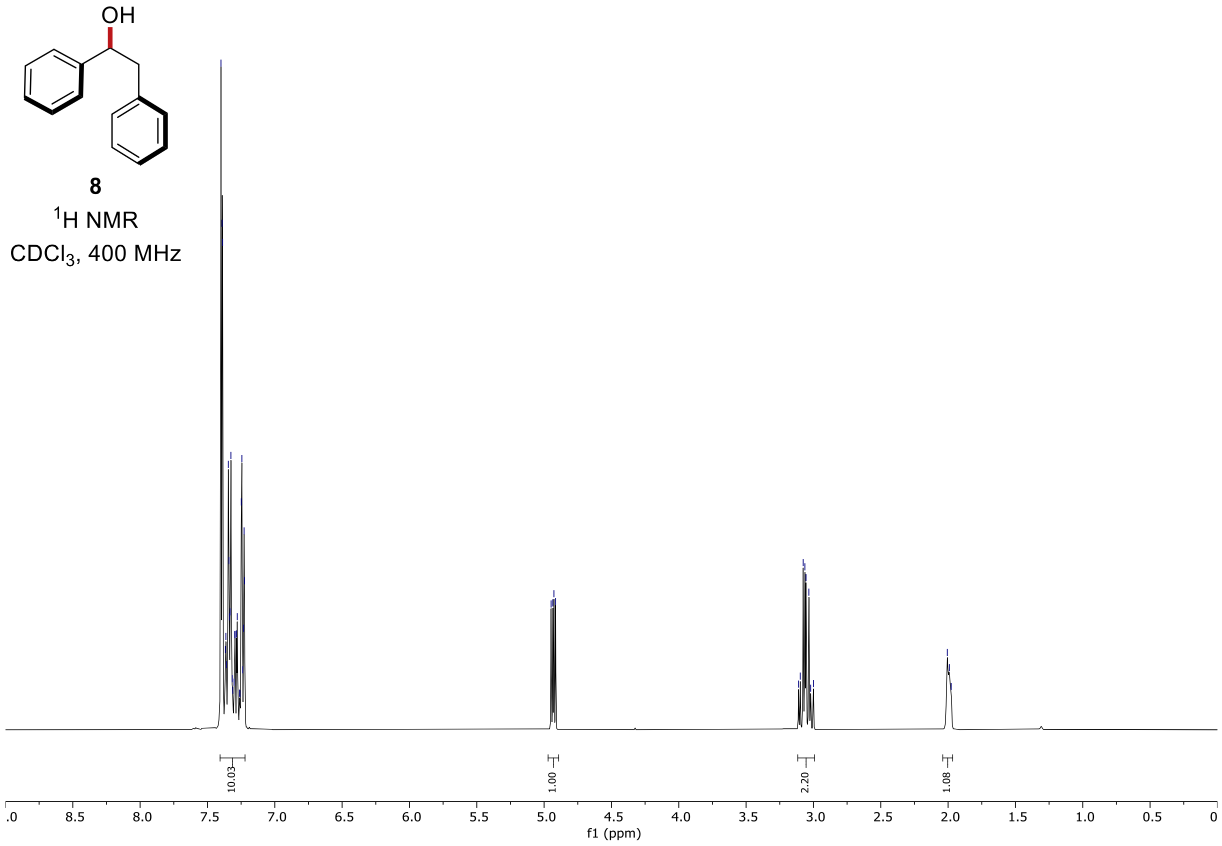
OH
8
^{1}H NMR
$CDCl_3$, 400 MHz
10.03
1.00
2.20
1.08
f1 (ppm)

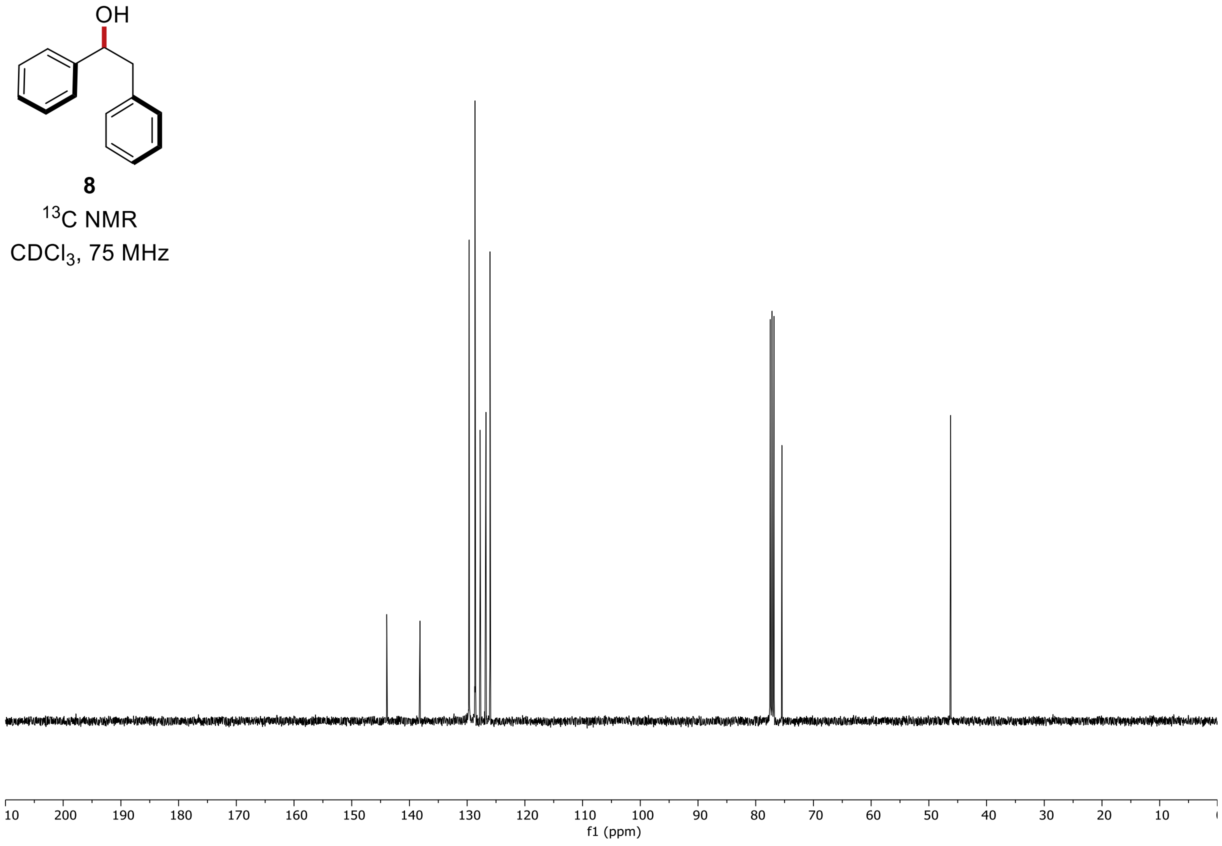
OH
8
^{13}C NMR
$CDCl_3$, 75 MHz
f1 (ppm)

References

1. Taeufer, T.; Argüello Cordero, M. A.; Petrosyan, A.; Surkus, A.-E.; Lochbrunner, S.; Pospech, J., Photophysical and Electrochemical Properties of Pyrimidopteridine-Based Organic Photoredox Catalysts. *ChemPhotoChem* **2021,** *5,* 999-1003.
2. Hauptmann, R.; Petrosyan, A.; Fennel, F.; Argüello Cordero, M. A.; Surkus, A.-E.; Pospech, J., Pyrimidopteridine N-Oxide Organic Photoredox Catalysts: Characterization, Application and Non-Covalent Interaction in Solid State. *Chem. Eur. J.* **2019,** *25*, 4325-4329.
3. Li, L.; Hilt, G., Regiodivergent DH or HD Addition to Alkenes: Deuterohydrogenation versus Hydrodeuterogenation. *Org. Lett.* **2020,** *22,* 1628-1632.
4. Mato, M.; Herlé, B.; Echavarren, A. M., Cyclopropanation by Gold- or Zinc-Catalyzed Retro-Buchner Reaction at Room Temperature. *Org. Lett.* **2018,** *20,* 4341-4345.
5. Zhao, Y.; Weix, D. J., Nickel-Catalyzed Regiodivergent Opening of Epoxides with Aryl Halides: Co-Catalysis Controls Regioselectivity. *J. Am. Chem. Soc.* **2014,** *136,* 48-51.
6. Stoll, S.; Schweiger, A., EasySpin, a comprehensive software package for spectral simulation and analysis in EPR. *J. Magn. Reson.* **2006,** *178,* 42-55.
7. Taeufer, T.; Hauptmann, R.; El-Hage, F.; Mayer, T. S.; Jiao, H.; Rabeah, J.; Pospech, J., Pyrimidopteridine-Catalyzed Hydroamination of Stilbenes with Primary Amines: A Dual Photoredox and Hydrogen Atom Transfer Catalyst. *ACS Catal.* **2021,** *11*, 4862-4869.
